# Identification and MS-assisted interpretation of genetically influenced NMR signals in human plasma

**DOI:** 10.1186/gm417

**Published:** 2013-02-15

**Authors:** Johannes Raffler, Werner Römisch-Margl, Ann-Kristin Petersen, Philipp Pagel, Florian Blöchl, Christian Hengstenberg, Thomas Illig, Christa Meisinger, Klaus Stark, H-Erich Wichmann, Jerzy Adamski, Christian Gieger, Gabi Kastenmüller, Karsten Suhre

**Affiliations:** 1 Institute of Bioinformatics and Systems Biology, Helmholtz Zentrum München, German Research Center for Environmental Health, Ingolstädter Landstraße 1, 85764 Neuherberg, Germany; 2 Faculty of Biology, Ludwig-Maximilians-Universität, Großhaderner Straße 2, 82152 Planegg-Martinsried, Germany; 3 Institute of Genetic Epidemiology, Helmholtz Zentrum München, German Research Center for Environmental Health, Ingolstädter Landstraße 1, 85764 Neuherberg, Germany; 4 numares GmbH, Josef-Engert-Str. 9, 93053 Regensburg, Germany; 5 Klinik und Poliklinik für Innere Medizin II, University of Regensburg, Franz-Josef-Strauss-Allee 11, 93053 Germany; 6 Research Unit of Molecular Epidemiology, Helmholtz Zentrum München, German Research Center for Environmental Health, Ingolstädter Landstraße 1, 85764 Neuherberg, Germany; 7 Hannover Unified Biobank, Hannover Medical School, Carl-Neuberg-Straße 1, 30625 Hannover, Germany; 8 Institute of Epidemiology II, Helmholtz Zentrum München, German Research Center for Environmental Health, Ingolstädter Landstraße 1, 85764 Neuherberg, Germany; 9 Department of Epidemiology and Preventive Medicine, University of Regensburg, Franz-Josef-Strauss-Allee 11, 93053 Germany; 10 Institute of Epidemiology I, Helmholtz Zentrum München, German Research Center for Environmental Health, Ingolstädter Landstraße 1, 85764 Neuherberg, Germany; 11 Institute of Experimental Genetics, Genome Analysis Center, Helmholtz Zentrum München, German Research Center for Environmental Health, Ingolstädter Landstraße 1, 85764 Neuherberg, Germany; 12 Department of Physiology and Biophysics, Weill Cornell Medical College in Qatar, Education City, Qatar Foundation, P.O. BOX 24144, Doha, Qatar

## Abstract

Nuclear magnetic resonance spectroscopy (NMR) provides robust readouts of many metabolic parameters in one experiment. However, identification of clinically relevant markers in ^1^H NMR spectra is a major challenge. Association of NMR-derived quantities with genetic variants can uncover biologically relevant metabolic traits. Using NMR data of plasma samples from 1,757 individuals from the KORA study together with 655,658 genetic variants, we show that ratios between NMR intensities at two chemical shift positions can provide informative and robust biomarkers. We report seven loci of genetic association with NMR-derived traits (APOA1, CETP, CPS1, GCKR, FADS1, LIPC, PYROXD2) and characterize these traits biochemically using mass spectrometry. These ratios may now be used in clinical studies.

## Background

Identification of the genetic and environmental determinants of human metabolism is key to the understanding of complex disorders. Metabolic profiling of blood samples from cohort studies is a major tool in the discovery of new disease relevant biomarkers and the metabolic individuality in the general population. Two major techniques are generally used: mass spectrometry (MS) and nuclear magnetic resonance spectroscopy (NMR). At the basis, both platforms are complementary in many regards. For instance, MS is more sensitive and allows obtaining more detailed information while using small sample volume, but is also quite sensitive to changing experimental conditions, and sample preparation is more demanding. NMR-based measurements, on the other hand, require higher sample volumes and the interpretation of the raw spectra is not always straightforward. Still, the strongest advantages of NMR are simplicity of sample preparation and excellent reproducibility of quantitative metabolic readouts. It has previously been shown that the stability of NMR measurements is to some extend independent of the NMR platforms and laboratory practices [1,2]. Therefore, NMR spectrometry is predestined for routine clinical applications as it generates datasets that are comparable between clinics and over time.

Previously, we and others have shown the potential of MS-based and NMR-based metabolomics in discovery studies such as the KORA study [3-7]. Using a genome-wide association approach with metabolite concentrations, so-called 'genetically influenced metabotypes' (GIM) have been discovered. These GIMs, which define the genetic basis of human metabolic individuality, have, in many cases, been linked to clinically relevant endpoints. For instance, polymorphisms in the fatty acid desaturase 1 (FADS1) locus are associated with Crohn's disease [8] and are furthermore suspected to play a role in cardiovascular disorders through association with cholesterol and triglycerides [9]. As further examples, the glucokinase regulator (GCKR) gene is a risk locus for several diabetes-relevant traits [10], and genetic variants at the carbamoyl-phosphatase 1 (CPS1) locus are associated with risk factors for chronic kidney disease [11].

Up to now, GWAS using metabolic traits mainly focused on metabolites that were identified by the annotation of MS and NMR spectra using reference spectra in existing databases.

Here, we address the question of whether known clinically relevant GIMs can be derived from raw NMR spectra in blood plasma without prior annotation. We take a hypothesis-free approach to identify genetically influenced NMR features and investigate whether specific ratios between NMR intensities at two chemical shifts can provide informative and robust biomarkers. Finally, we interpret our findings using correlations to MS determined metabolite concentrations.

With the KORA F4 study cohort, we have unique access to a large number of samples of the general population, which are metabolically deeply characterized both using MS and NMR methods. In previous studies based on these data, we showed that using ratios between metabolite concentrations can strengthen genotype-metabotype associations. In such cases, the ratios often reflect educt-product pairs of enzymatic reactions [12]. Based on this idea, we here conduct a genome-wide association study (GWAS) on the binned NMR spectra, in combination with testing ratios between pairs of intensity values at different chemical shift positions. We then confront our results with findings from previous GWAS with MS based metabolomics [4,5] and NMR derived lipid subclasses [13]. Finally, we interpret the results in the light of the existing MS determined metabolite concentrations and data originating from clinical biochemistry measurements.

## Methods

### Study population

The KORA study is an independent population-based survey from the general population living in the region of Augsburg, Germany. The KORA S4 study was conducted in 1999-2001 and comprises a total of 4,261 participants [14]. Between 2006 and 2008, a total of 3,080 subjects participated in a follow-up examination, KORA F4, which is the basis for the results presented here. All participants gave their signed informed consent and the local ethics committee approved the studies.

### Blood sampling

Blood samples for metabolic analysis and DNA extraction were collected as part of the KORA F4 follow-up study. To avoid variation due to circadian rhythm, blood was drawn in the morning between 08:00 and 10:30 after overnight fasting. A part of the blood was drawn into serum gel tubes, gently inverted twice, and then allowed to rest for 30 min at room temperature (18-25°C) to obtain complete coagulation. The material was then centrifuged for 10 min (2,750 g at 15°C). Serum was divided into aliquots and kept for a maximum of 6 h at 4°C, after which it was frozen at -80°C until final analysis. Another part of the blood was drawn into ethylene diaminetetraacetic acid (EDTA) tubes, gently inverted twice, and left on the Sarstedt Universal mixer <5 min to avoid mechanical hemolysis, followed by centrifugation at 15°C for 10 min at 2,750 g. Thereafter, plasma was separated, divided into 200 mL aliquots and kept at 4°C, after which it was deep-frozen to -80°C. Within 2 weeks, plasma was stored in the gaseous phase of liquid nitrogen at -196°C.

### Genotyping

For all individuals profiled from the KORA study, genome-wide single nucleotide polymorphism (SNP) data were already available. These data have been used and described extensively in the past in the context of several GWAS (for example, [4,5]). Therefore, we summarize only the essential details here. For genotyping, 1,814 randomly selected participants of KORA F4 were included. These samples were genotyped using the Affymetrix Human SNP Array 6.0 (sample call rate >93%). Genotypes were determined using the Birdseed2 clustering algorithm. For quality assurance, the criteria of SNP call rate >95%, minor allele frequency >1%, and P(Hardy-Weinberg) >10^-6 ^were applied as filters. In total, 655,658 autosomal SNPs satisfied these criteria.

### Metabolomics measurements

Metabolic analyses were conducted using clinical biochemistry methods, two distinct MS-based platforms (targeted and non-targeted), and NMR spectrometry (lipid classes and binned spectral data). For the joint analysis, metabolomics and genotype data were available for a total of 1,757 individuals. All metabolite measurements have been reported before. We therefore only summarize here the points that are essential for the present study.

#### Clinical biochemistry

The following serum lipids were measured on fresh samples using the Dimension RxL (Dade Behring). TC was determined by cholesterol esterase method (CHOL Flex, Dade-Behring, CHOD-PAP method), HDL-C using the AHDL Flex (Dade-Behring, CHOD-PAP method after selective release of HDL-C), LDL-C using the ALDL Flex (Dade Behring, CHOD-PAP method after colorless usage of all non-LDL-C), and TG was measured using a TGL Flex (Dade Behring, enzymatic colorimetric test, GPO-PAP method).

#### Targeted metabolomics

The Biocrates AbsoluteIDQ p150 kit was used for absolute quantification of a defined set of serum metabolites. Sample analyses were done on an API 4000 Q TRAP LC/MS/MS system (Applied Biosystems) equipped with a Schimadzu Prominence LC20AD pump and a SIL-20AC autosampler. The complete analytical process was performed using the MetIQ software package, which is an integral part of the AbsoluteIDQ kit. In total, we detected 163 different metabolites. The metabolomics dataset contains 14 amino acids, hexose (H1), free carnitine (C0), 40 acylcarnitines (Cx:y), hydroxylacylcarnitines (C(OH)x:y), and dicarboxylacylcarnitines (Cx:y-DC), 15 sphingomyelins (SM Cx:y) and N-hydroxylacyloylsphingosylphosphocholine (SM(OH) Cx:y), 77 phosphatidylcholines (PC, aa = diacyl, ae = acyl-alkyl) and 15 lyso-phosphatidylcholines. Lipid side chain composition is abbreviated as Cx:y, where x denotes the number of carbons in the side chain and y the number of double bonds. For further details on this dataset and the coefficients of variation (CVs) of metabolite quantification see [4].

#### Non-targeted metabolomics

Metabolon, a commercial supplier of metabolic analyses, developed a platform that integrates the chemical analysis, including identification and relative quantification, data-reduction, and quality-assurance components of the process. The analytical platform incorporates two separate ultrahigh-performance liquid chromatography/tandem mass spectrometry (UHPLC/MS/MS2) injections and one gas chromatography/mass spectrometry (GC/MS) injection per sample. The UHPLC injections were optimized for basic and acidic species. The LC/MS portion of the platform was based on a Waters ACQUITY UPLC and a Thermo-Finnigan LTQ mass spectrometer, which consisted of an electrospray ionization (ESI) source and a linear ion-trap (LIT) mass analyzer. For GC/MS, the samples were analyzed on a Thermo-Finnigan Trace DSQ fast-scanning single-quadrupole mass spectrometer using electron impact ionization. A total of 295 serum metabolites were measured, spanning several relevant classes (amino acids, acylcarnitines, sphingomyelins, glycerophospholipids, carbohydrates, vitamins, lipids, nucleotides, peptides, xenobiotics, and steroids). The detection of the entire panel was carried out with 24 min of instrument analysis time (two injections at 12-min each), while maintaining low median process variability (<12% across all compounds). The resulting MS/MS^2 ^data were searched against a standard library generated by Metabolon that included retention time, molecular mass to charge ratio (m/z), preferred adducts and in-source fragments as well as their associated MS/MS spectra for all molecules in the library. The library allowed for the identification of the experimentally detected molecules on the basis of a multiparameter match without the need for additional analyses. For further details on this dataset and the coefficients of variation (CVs) of the metabolite measurements see [5].

#### NMR-derived lipid classes

NMR spectra measurements were carried out at numares (formerly LipoFIT), Regensburg. The blood plasma spectra were recorded on a Bruker 600 MHz Avance IIplus spectrometer. The spectra were phased and baseline corrected. Since no reference compound was added, all spectra were horizontally aligned to the prominent lactate signals at 1.36 and 1.37 ppm. Based on a proprietary approach that uses complex deconvolution algorithms on the spectral regions between 0.6 and 1.5 ppm, a set of 15 lipoprotein subfractions was derived from the spectra. These fractions correspond to HDL (small = L1, medium = L2, large = L3, very large = L4), LDL (very small = L5, small = L6, medium = L7, large = L8, very large = L9), IDL (L10), VLDL (small = L11, large = L12), remnants (L13), and chylomicrons (small = L14, large = L15). For further details on this dataset see [13].

#### NMR binned data

For the purpose of this study, the spectra were limited to a range from 0 to 9 ppm and divided into 10,000 bins of equal width (0.001 ppm). Spectral binning is a standard procedure in NMR-based metabolomics studies that reduces the data complexity and compensates for slight jitter of the signals' chemical shifts [15]. The intensity values were log_10_-transformed prior to analysis. To diminish the effect of outliers, for each bin individual intensity values more than three times the standard deviation away from the mean were excluded. Also, the spectral region affected by the water peak (δ = 4.6-5 ppm) was excluded from further analysis.

### Statistical analysis

To test for associations between genetic polymorphisms and individual NMR signal bins, we created age- and gender-adjusted linear additive models using PLINK (Version 1.07) [16]. Testing all possible ratios between NMR signals all over the spectrum would not be feasible due to the huge amount of computational time that would be needed. Therefore, we performed a simple feature selection. In most cases SNPs do not associate with only one NMR signal bin but with a number of adjacent bins. Thus, we performed a search for local minima on the pseudo-spectra (chemical shift *vs*. strength of association) resulting from the first GWAS run to pick the positions on the spectra which show the strongest associations. We then used the 500 best-scoring NMR signal positions to compute pair wise ratios (124,750 in total). As before, we used age- and gender-adjusted linear additive models in PLINK to test for associations.

We applied a conservative Bonferroni correction to control for false-positive error rates resulting from multiple testing. We corrected for tests on 655,658 SNPs and 133,350 NMR features at a nominal significance level of 5%, thus obtaining an adjusted *P *value of 0.05/655,658/133,350 = 5.72 × 10^-13^.

Spearman correlations between biochemically and MS determined metabolite concentrations and NMR signal intensities were calculated using the statistical analysis system R (Version 2.15.1). To avoid false-positive associations due to small sample sizes, only metabolic traits with at least 300 non-missing values were included. Furthermore, metabolite concentrations that were more than three times the standard deviation away from the mean were excluded.

## Results

### Seven genetic associations identified

We adopted a two-stage approach to detect associations between genetic variants and NMR signals. In the first step, we performed a GWAS using the NMR intensity readouts of the ^1^H-NMR spectra from 1,757 plasma samples. To this end, we binned the spectral region ranging from 0 to 9 ppm at a high resolution of 0.001 ppm. The region surrounding the water peak (δ = 4.6-5 ppm) was discarded. NMR intensities were log_10_-scaled and extreme outliers were removed (see Methods). Usually, NMR-based GWAS either use a selection of chemical shifts or perform spectral annotation to reduce the NMR data to the underlying metabolite concentrations (for example, [6,7,17]). In contrast, our approach uses the signal intensities at almost all chemical shift positions of the NMR spectra.

In the second step, we selected the 500 bins in the NMR spectrum that exhibited the strongest signal of association. In cases where neighboring bins all displayed associations of comparable strength to the same single nucleotide polymorphism (SNP), the bin with the lowest *P *value was selected. In a second GWAS, we then tested all possible ratios between the intensities at these 500 chemical shift positions for associations with all genetic variants. To our knowledge, this is the first GWAS that uses a ratio-based, hypothesis-free approach with raw NMR spectral data as a phenotype. For both GWAS, age and gender were used as covariates. In the subsequent analysis, we considered only robust associations of frequent SNPs with minor allele frequencies (MAF) >10%.

In total, seven loci (LIPC, CETP, FADS1, GCKR, APOA1, CPS1, PYROXD2) displayed a signal of association that attained the genome-wide level of significance (*P *<5.72 × 10^-13^) after Bonferroni correction for 655,658 tested SNPs and 133,350 NMR traits (133,350 = 500*499/2 ratios + 8,600 chemical shifts). Table 1 lists the loci with significant associations to NMR features, the lead SNPs showing the strongest associations within the loci, respectively, and the strength of association. Additional file 1 provides regional association plots, boxplots, and quantile-quantile plots for all associations listed in Table 1. All loci were previously reported in the same KORA F4 dataset using MS [3-5], with the exception of the PYROXD2 locus, which has been reported by Nicholson *et al. *in 2011 [6].

**Table 1:** Genetic associations with NMR intensities and ratios between NMR intensities.

**Locus**	**SNP**	**CHR**^a^	**POS**^**a**^	**A**^**a**^	**B**^**a**^	**MAF**	**Chemical shift(ppm)**	***n***	**Beta'**^**b**^	***P *** **value**	**Chemical shifts for ratios (ppm)**	***n***	**Beta' ratios**^**b**^	***P *** **value ratios**	***P*** **gain**^c^
GCKR	rs780094	2	27,594,741	T	C	41.4%	1.370	1,688	0.0413	1.2x10^-10^	3.286/1.370	1,667	-0.0407	2.8x10^-15^	4.3x10^4^
CPS1	rs2216405	2	211,325,139	C	T	19.3%	3.599	1,719	0.0456	4.5x10^-14^	3.599/2.475	1,718	0.0459	1.8x10^-19^	2.5x10^5^
PYROXD2	rs4488133	10	100,149,126	A	T	34.3%	2.757	1,735	-0.0190	7.3x10^-12^	2.757/2.755	1,734	-0.0123	2.9x10^-94^	2.5x10^82^
FADS1	rs174547	11	61,327,359	C	T	30.0%	2.801	1,733	-0.0408	4.0x10^-35^	2.801/2.017	1,724	-0.0449	1.1x10^-94^	3.8x10^59^
APOA1	rs3741298	11	116,162,771	C	T	21.4%	2.038	1,724	0.0363	8.4x10^-11^	4.162/4.082	1,715	-0.0138	1.8x10^-14^	4.6x10^3^
LIPC	rs4775041	15	56,461,987	C	G	28.5%	1.283	1,671	0.0320	1.4x10^-10^	1.068/1.029	1,664	-0.0056	3.6x10^-21^	4.0x10^10^
CETP	rs247617	16	55,548,217	A	C	31.3%	3.259	1,705	0.0529	7.6x10^-15^	2.211/2.011	1,695	-0.0124	1.1x10^-18^	6.8x10^3^

The locus name indicates the gene that most likely hosts the causative SNP.

A/B: minor/major allele; MAF: minor allele frequency; *n*: number of samples where genetic and NMR data are jointly available; ppm: parts per million.

^a^Chromosomal location (CHR, POS), minor (effect, A) and major (B) alleles are reported with respect to the positive strand of the human genome (NCBI build 36.1).

^b^Beta': relative effect size, defined as beta' = (10^beta^- 1), where beta is the slope in the linear model (using log_10_-scaled traits) and genotype is coded as 0-1-2 (major-hetero-minor genotype). Beta' represents the relative difference per copy of the minor allele for the unscaled metabolic trait (NMR bin or NMR ratio) compared to the estimated mean of the major allele homozygote group (intercept of the linear model).

^c^The *P *gain is defined as min(p(S_1_)/p(S_1_/S_2_), p(S_2_)/p(S_1_/S_2_)), where p(S_1_), p(S_2_), and p(S_1_/S_2_) denote the *P *values of association of two log-scaled NMR intensities at chemical shift S_1 _and S_2_, and of their ratio S_1_/S_2_, respectively.

### Overlap with NMR-derived lipid subclasses

Recently, Petersen *et al. *reported an association study with NMR-derived lipoprotein subclasses [13]. Their analysis is based on the same NMR data as used in this study. In contrast to our non-targeted approach, they used a targeted approach that derives 15 different lipoprotein subclasses from the spectral region between 0.6 ppm and 1.5 ppm. The authors tested 101 SNPs in known lipid loci for associations with these lipoprotein subclasses. As a result, they identified eight loci that associated specifically with one or more of these subclasses. Of the eight loci reported by Petersen *et al*., we identified five (LIPC, CETP, FADS1, GCKR, APOA1) in association with NMR intensities at single chemical shift positions (NMR bins) in our genome-wide approach. Note that in comparison, the strength of association is generally weaker when testing NMR bins instead of lipid subclasses, as one would expect when using a less aggregated parameter. However, in the GWAS with ratios of chemical shift pairs (NMR ratios), we observe a strong increase in the strength of association for four loci (LIPC, CETP, FADS1, GCKR), resulting in *P *values that are between 9 and 89 orders of magnitude lower than the values reported by Petersen *et al. *for the associations with lipid subclasses (Table 2). In these four cases, the ratios represent stronger readouts of metabolic phenotypes that are modified by the genetic variant than do either the binned NMR intensities or the NMR-derived lipid subclasses. These ratios are thus worthy of further investigation (see below).

**Table 2:** Comparison with association data from previous studies on the same specimen.

**Locus**		**Mass spectrometry Targeted, quantitative **[3,4]	**Mass spectrometry Non-targeted, semi-quantitative **[5]	**NMR Lipid subclasses **[13]	**NMR Ratios between chemical shifts(this study)**
GCKR	Trait	Pc ae C34:2/Pc aa C32:2	Glucose/Mannose	L10	3.286 ppm/1.370 ppm
	SNP	rs1260326, LD r^2 ^= 0.93	rs780094	rs1260326, LD r^2 ^= 0.93	rs780094
	*P *value	*P *= 3.8x10^-8^	*P *= 4.9x10^-32^	*P *= 3.7x10^-6^	*P *= 2.8x10^-15^
CPS1	Trait	Glycine/PC aa C38:2	Asparagine/Glycine		3.599 ppm/2.475 ppm
	SNP	rs2216405	rs2216405	(locus not reported)	rs2216405
	*P *value	*P *= 1.9x10^-30^	*P *= 9.8x10^-21^		*P *= 1.8x10^-19^
PYROXD2	Trait		Saccharin/Threonine		2.757 ppm/2.755 ppm
	SNP	(locus not reported)	rs4488133	(locus not reported)	rs4488133
	*P *value		*P *= 0.00021		*P *= 2.9x10^-94^
FADS1	Trait	PC aa C36:3/PC aa C36:4	1-arachidonoylglycero-phosphoethanolamine/1-linoleoylglycero-phosphoethanolamine	L4	2.801 ppm/2.017 ppm
	SNP	rs174547	rs174547	rs174546, LD r^2 ^= 1.0	rs174547
	*P *value	*P *= 6.5x10^-179^	*P *= 1.2x10^-80^	*P *= 1.4x10^-5^	*P *= 1.1x10^-94^
APOA1	Trait	PC aa C36:2/PC aa C38:1	1-oleoglycerol/Oleamide	L8	4.162 ppm/4.082 ppm
	SNP	rs964184, LD r^2 ^= 0.61	rs3741298	rs964184, LD r^2 ^= 0.61	rs3741298
	*P *value	*P *= 1.8x10^-10^	*P *= 4.3x10^-7^	*P *= 4.8x10^-12^	*P *= 1.8x10^-14^
LIPC	Trait	PCaa C38:6	1-palmitoylglycero-phosphoethanolamine	L5	1.068 ppm/1.029 ppm
	SNP	rs4775041	rs4775041	rs1532085, LD r^2 ^= 0.55	rs4775041
	*P *value	*P *= 9.7x10^-8^	*P *= 8.7x10^-7^	*P *= 5.3x10^-11^	*P *= 3.6x10^-21^
CETP	Trait		Guanosine/Phenylacetylglutamine	L3	2.211 ppm/2.011 ppm
	SNP	(locus not reported)	rs247617	rs3764261, LD r^2 ^= 1.0	rs247617
	*P *value		*P *= 0.00039	*P *= 3.6x10^-7^	*P *= 1.1x10^-18^

Association data from previous GWAS for the loci reported in this study are reported either on the same SNP, or, if a different SNP was reported, the correlation with the SNP in linkage disequilibrium (r^2 ^LD) is given (based on HapMap, release #27, phases I, II, III, CEU population).

### Strong correlations of NMR signals to MS-based and further metabolite concentrations

**Figure 1:**
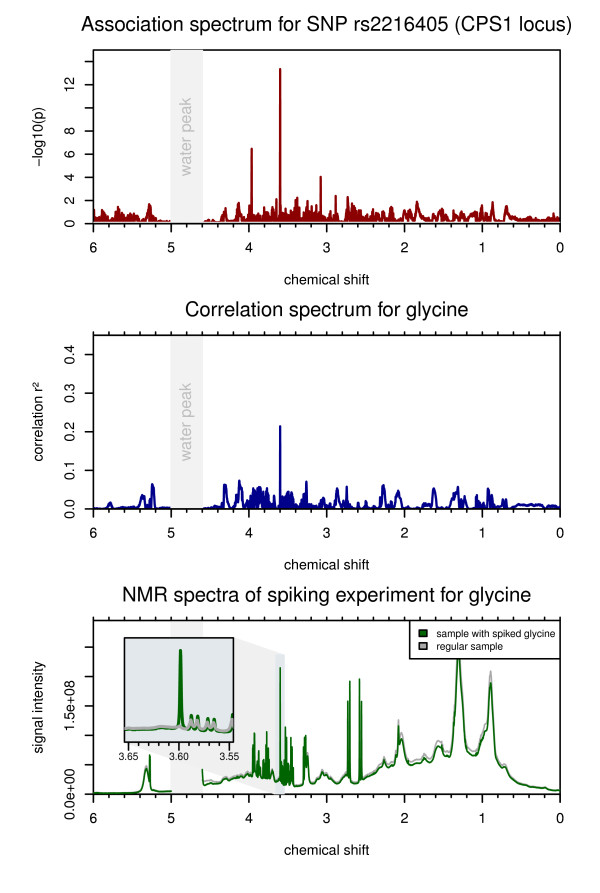
**Association at the CPS1 locus**. Top: The red line indicates the strength of association (-log_10_(*P *value)) of SNP rs2216405to the chemical shifts. The strongest association can be observed with the NMR signals at δ = 3.599 ppm (*P *<4.5 × 10^-14^). Middle: The blue line gives the squared Spearman correlation coefficient (r_s_^2^) for MS determined glycine and the NMR signals. The highest correlation (r_s_^2 ^= 0.21) can be observed as a sharp peak at δ = 3.599 ppm. Bottom: The plot shows the NMR spectra of two samples, where one was spiked with glycine (green spectral line). The inset shows a magnification of the spectral region around δ = 3.6 ppm. This experiment confirms that the signal intensities at δ = 3.599 ppm are indeed driven by glycine.

**Figure 2:**
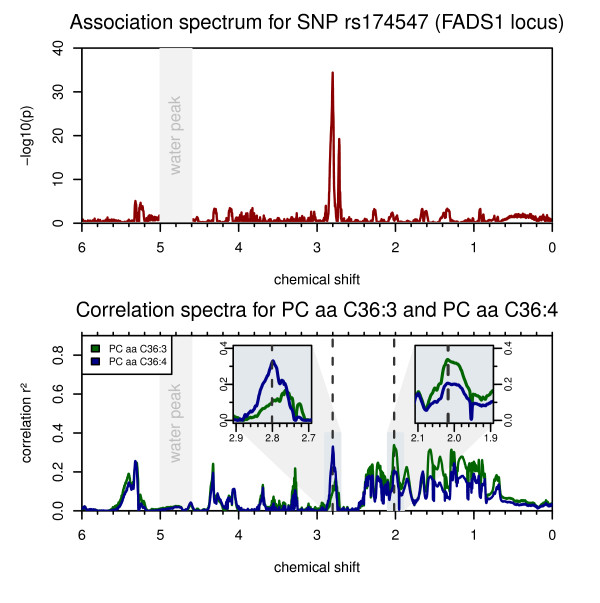
**Association at the FADS1 locus**. SNPrs174547 associates most strongly with the NMR signal at chemical shift δ = 2.801 ppm (*P *= 3.7 × 10^-35^) (top). When ratios between NMR intensities are tested, the strength of association increases by 59 orders of magnitude for the ratio between NMR intensities at 2.801 ppm and 2.017 ppm (*P *= 1.1×10^-94^) (Table 1). A similar increase in the strength of association has been observed in previous GWAS for this locus with ratios between phospholipids containing C20:3 and C20:4 fatty acids, such as PC aa C36:3 and PC aa C36:4 [3]. The FADS1 codes for a delta-5 fatty acid desaturase; C20:3 and C20:4 fatty acids are their substrate-product pair. The correlation plot between these lipid species and the NMR intensities (bottom) indicates that the region around δ = 2.801 ppm correlates more strongly with C20:4 fatty acid-containing lipids, while the region around δ = 2.017 ppm more with C20:3 lipids (bottom insets). The ratio between intensities at δ = 2.801 ppm and δ = 2.017 ppm is therefore a likely proxy for the ratio between three- and four-fold desaturated log chain fatty acids.

**Figure 3:**
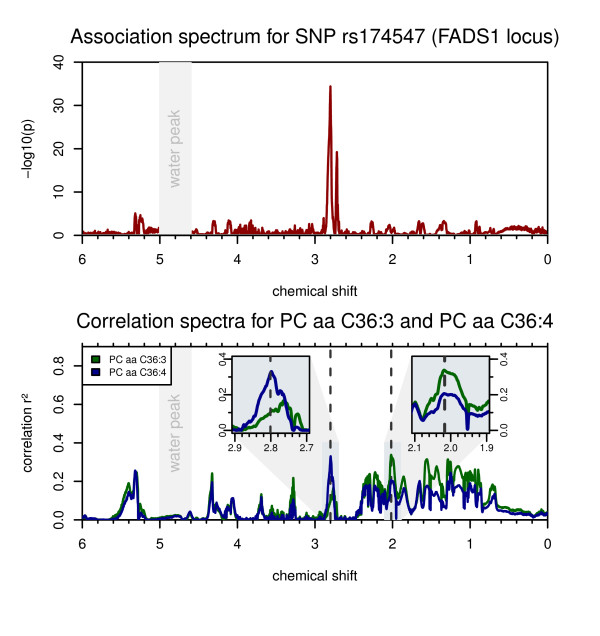
**Association at the GCKR locus**. SNP rs780094 (and SNPs in LD) have been associated with different diabetes-relevant traits, including fasting glucose and triglyceride levels (TG). We observe a genome-wide significant association (*P *= 2.8x10^-15^) at this locus with the ratio of intensities at δ = 3.286 ppm and δ = 1.370 ppm (dashed lines) (Table 1). The correlation spectra for glucose and TG (both parameters were determined by clinical biochemistry) show that the association signal at δ = 3.286 ppm is mainly driven by glucose and that at δ = 1.370 ppm by TG. The ratio of these two NMR intensities is thus a likely proxy for a diabetes-relevant composite readout on the GCKR pathway.

A ^1^H-NMR spectrum of a biological sample is the superposition of the resonance spectra of all individual metabolites measured in that sample. The signals of different metabolites thus overlap in the NMR spectrum. Identification and annotation of individual metabolites is a challenging task. Here we compute 'correlation spectra' to visualize the degree of correlation between NMR bins and MS-derived or biochemically determined metabolite concentrations. Note that all measurements (NMR, MS, clinical biochemistry) were performed on blood samples from the same draw and subject. Correlation spectra thus link the chemical identity of the metabolites detected by other platforms to chemical shifts in the NMR spectra, where the chemical shifts may correspond to the same metabolite, but also to a metabolite on a related biochemical pathway. As an example, Figure 1 shows the correlation spectrum for MS determined glycine. We also observed a number of correlations with phosphatidylcholines (PCs) (Figure 2), and with parameters that were determined independently using standard clinical biochemistry methods, namely triglycerides (TG), high-density lipoproteins (HDL), low-density lipoproteins (LDL), total cholesterol (TC), and glucose (Figure 3), and a number of other metabolites. Most of the correlations we observed are positive, meaning that a stronger NMR signal at the given chemical shift goes with a higher concentration of the correlated metabolite, as one would expect in cases where MS and NMR are targeting identical or closely-related metabolites. In a few cases, we also observe negative correlations between NMR bins and MS-determined metabolites. This may indicate cases where the NMR signal is indirectly related to a MS-measured metabolite. It could, for instance, correspond to the product or substrate of a metabolite quantified on the MS platform.

In the case of correlation with lipid-related parameters, the correlation spectra generally show wider areas of the NMR spectrum corresponding to these metabolites (for example, PCs in Figure 2, triglyceride levels in Figure 3). In contrast, correlations with non-lipid metabolites are visible as individual, sharp peaks (for example, glycine in Figure 1 and glucose in Additional file 2). These peaks correlate, among others, with the concentrations of glucose, lactate, proline, and glycine with squared Spearman correlation coefficients (r_s_^2^) up to 0.61. In most cases, we can verify the validity of the correlations using experimental spectra of pure compounds from HMDB [18]. For glycine, we conducted an additional spiking experiment to confirm that the NMR signal at δ = 3.599 ppm indeed is driven by glycine. However, since many metabolites are by nature interlinked in metabolic networks, some signal correlations do not reflect just a single compound but a mixture of co-regulated (and therefore auto-correlated) metabolites. Also, due to overlapping signals in NMR spectra, there may be multiple, non-related metabolites showing correlations at the same chemical shift position.

Additional file 3, Table S1 lists all metabolites that correlate with NMR signals at r_s_^2 ^= 0.20 (or above) and the chemical shift where the correlation coefficient is highest. The corresponding correlation spectra are provided in Additional file 2.

For all associations between NMR bins and genetic loci, we report the best correlating biochemically or MS-determined metabolite at the given chemical shift position (Table 3). In case of NMR ratios, we report either the correlations with single metabolite concentrations or, if stronger, the correlations with ratios of metabolite concentrations. The rationale behind this procedure is that two different chemical shifts might be representative for two specific pairs of metabolites and a strong correlation would indicate a potential biochemical match between NMR chemical shifts and the biochemically or MS-derived metabolites.

**Table 3:** Spearmancorrelations between NMR intensities and metabolic traits determined by MS and clinical biochemistry.

**Locus**	**SNP**	**Chemical shift (ppm)**	**Metabolite**	**r_s_^2^**	**Chemical shifts for ratios (ppm)**	**Metabolite or Metabolite ratios**	**r_s_^2^**
GCKR	rs780094	1.370	Triglyceride	0.62	3.286/1.370	Glucose/Triglyceride	0.42
CPS1	rs2216405	3.599	Glycine	0.22	3.599/2.475	Glycine/Tryptophan	0.29
PYROXD2	rs4488133	2.757	Total cholesterol	0.64	2.757/2.755	Total cholesterol/Triglyceride	0.26
FADS1	rs174547	2.801	PC aa C38:5	0.48	2.801/2.017	PC aa C38:5/ PC aa C36:3	0.45
APOA1	rs3741298	2.038	Triglyceride	0.73	4.162/4.082	Lactate/Triglyceride	0.60
LIPC	rs4775041	1.283	PC aa C36:1	0.42	1.068/1.029	4-methyl-2-oxopentanoate/Total cholesterol	0.21
CETP	rs247617	3.259	HDL cholesterol	0.55	2.211/2.011	HDL cholesterol	0.68

The 'Metabolite' and 'Metabolite ratios' columns list the MS and biochemically determined metabolites (or ratios thereof) that correlate best at the chemical shift positions (or ratios) identified in the GWAS.

ppm: parts per million.

## Discussion

In this study, we perform a GWAS with binned NMR spectra and ratios between pairs of chemical shifts in a hypothesis-free approach without prior annotation of NMR features. In total, we identify seven genetic loci. Four of these associations (LIPC, PYROXD2, GCKR, APOA1) only reach a genome-wide significance level when ratios between the signal intensities at two different chemical shift positions are tested. Five of the seven loci (LIPC, CETP, FADS1, GCKR, APOA1) are also known lipid risk loci, and all have previously been identified in GWAS with metabolic traits. There is thus no doubt about the functional role of these genetic loci in inducing a metabolic phenotype. Moreover, correlating NMR features to known metabolic phenotypes determined on other platforms can help in the interpretation of the observed genetic associations for various loci and the characterization of the associated NMR trait, as shown in this study for the CPS1, FADS1, and GCKR loci.

Given the fact that these loci have already been largely studied for their interest from a biomedical point of view, we focus here on aspects that highlight NMR-specific features of the associations. The features presented in this work may now be used in future studies with clinical disease endpoints or may potentially be established as biomarkers. In the following, we focus on four of the loci, each of them highlighting a different situation.

### Singular NMR intensities (peaks) correlate with MS-based glycine measurements and provide comparable genetic associations (CPS1 locus)

The carbamoyl-phosphate synthase 1 (CPS1) controls the first step in the urea cycle. Klaus *et al. *report two mutations of the CPS1 gene that contribute to the onset of CPS1 deficiency, an inborn error of metabolism that causes hyperammonemia [19]. In 2010, Illig *et al. *reported an association of SNP rs2216405 with glycine concentrations at a nominal *P *value of 2.59×10^-26 ^[4]. Glycine is metabolically related to carbamoyl phosphate, which in turn is the product of CPS1. Thus, Illig *et al. *presented a genetically influenced metabotype that might represent a mild form of CPS1 deficiency.

Here, we find an association of the same SNP with the NMR signals at δ = 3.599 ppm (*P *= 4.46×10^-14^). The correlation between MS determined glycine and the NMR signals at that chemical shift is r_s_^2 ^= 0.22. Although this correlation is modest, the correlation spectrum for glycine shows a distinct peak at δ = 3.599 ppm (Figure 1). A reference spectrum for glycine taken from HMDB [18] shows a single peak at δ = 3.54 ppm. While the spectra used in this study were referenced to lactate and the sample pH was 7.4, the HMDB spectrum was referenced to 4,4-dimethyl-4-silapentane-1-sulfonic acid (DSS) and the sample pH was 7.0. Thus, the 0.06 ppm distance between the putative glycine peak in this study and the HMDB glycine peak can be explained by different experimental conditions. In a separate spiking experiment comparing two spectra with and without addition of glycine, we confirmed that the signal at δ = 3.599 ppm indeed corresponds with glycine.

Thus, in this case we find that information obtained from a GWAS with NMR data provides comparable results to what was obtained with MS, albeit the strength of the association observed with NMR data is weaker in this case. The NMR chemical shift δ = 3.599 ppm could potentially be used as a marker for mild forms of perturbations in the ammonia metabolism caused by genetic variations in the CPS1 locus.

### Associations with ratios between NMR intensities indicate spectral regions which are representative of triple and quadruple fatty acid desaturation (FADS1 locus)

The fatty-acid desaturase 1 (FADS1) gene product catalyzes the desaturation reaction of 8,11,14-eicosatrienoyl-CoA to arachidonoyl-CoA (C20:3 → C20:4). Genetic variants in this locus have been linked to Crohn's disease [8] and risk factors for cardiovascular disorders, namely cholesterol and triglyceride levels [9]. Recently, two independent studies showed that dietary intake of long-chain polyunsaturated fatty acids (for example, C20:3 and C20:4) modulates the association between genetic variation in FADS1 and serum lipid levels, and thereby potentially also modifies the risk of cardiovascular disease [20,21]. In their previous GWAS, Gieger *et al. *[3] and Illig *et al. *[4] identified strong associations of SNP rs174547 (intronic region of FADS1) with a number of glycerophospholipids, many containing lipid side chains with C20:3 and C20:4 polyunsaturated omega-3 and omega-6 fatty acids (PUFAs). The authors observed an exceptionally large increase in the strength of the association when ratios between phospholipids containing PUFAs with <4 double bonds and ≥4 double bonds were testedin pairs [4]. This observation can be explained by the fact that ratios between substrate: product pairs approximate the underlying enzymatic reaction rate and also because ratios between related metabolites reduce the overall variance that is observed between individuals with different levels of overall blood PUFA concentrations [12].

In our data, the correlation spectra for glycerophospholipid levels show distinct differences depending on whether the degree of saturation of the side chains is below four double bonds (precursor of substrates of FADS1 enzymatic reaction) or equal or above four double bonds (products downstream of FADS1) (Figure 2). When testing binned NMR intensities for association, SNP rs174547 displays associations at a wider range of chemical shifts, with the strongest signal at δ = 2.801 ppm (*P *= 3.96×10^-35^). Interestingly, when using ratios between the NMR intensities at δ = 2.801 ppm and 2.017 ppm, the strength of association increases by nearly 60 orders of magnitude (*P *= 1.1×10^-94^), similar to the MS-based case. The NMR ratio δ = 2.801 ppm/δ = 2.017 ppm is therefore a likely proxy for the ratio between three- and four-fold desaturated long chain fatty acids. As a metabolic marker, this ratio might be a readout for the efficacy of the FADS1 enzymatic reaction in the context of dietary intake of PUFAs.

### A ratio between NMR intensities in the triglyceride and in the glucose range of the spectrum constitutes an integrated pleiotropic diabetes risk marker (GCKR locus)

The product of the glucokinase regulator (GCKR) gene both transports and regulates glucokinase, a key enzyme of glucose metabolism [22]. GCKR is a genetic risk locus for diabetes-related traits [10].

In our study, SNP rs780094 (intronic region of GCKR) associates with NMR intensities at δ = 1.370 ppm. At this spectral position, we observe a high correlation between NMR signals and TG levels (r_s_^2 ^= 0.61) (Figure 3). However, this association is not of genome-wide significance (*P *= 1.2×10^-10^). When testing ratios for association, the strength of association increases by five orders of magnitude, with a *P *value of 2.8×10^-15 ^for the association of SNP rs780094 to the NMR ratio δ = 1.370 ppm/δ = 3.286 ppm. The correlation spectrum with glucose shows a distinct signal at δ = 3.286 ppm (r_s_^2 ^= 0.28). This chemical shift is indeed located within a known glucose-related spectral region. Furthermore, this NMR ratio correlates with the triglyceride/glucose ratio (r_s_^2 ^= 0.42). Interestingly, GCKR is a diabetes risk locus that inversely modulates triglyceride and fasting glucose levels [4,23-26].

Thus, the combination of these two chemical shifts likely provides a combined measure of two main diabetes-risk readouts that have been independently associated with the GCKR locus before. We suggest that this ratio therefore may constitute an integrated biomarker for the pleiotropic biological processes related to perturbations in the GCKR pathway.

### Ratios between neighboring NMR intensities act as a local baseline correction and strengthen the genetic association (PYROXD2 locus)

PYROXD2 is a probable pyridine nucleotide-disulphide oxidoreductase gene. Nicholson *et al. *recently found this locus to be associated with dimethylamine concentrations in plasma, also using NMR spectrometry [6]. At the time when our study was conducted, metabolomics information was only accessible using NMR methods. Meanwhile, an association with an MS determined, biochemically non-identified metabolite has been found, thus replicating this GIM on a different metabolomics platform [27]. However, a link of this metabotype to a phenotype of clinical relevance has not been reported so far.

**Figure 4:**
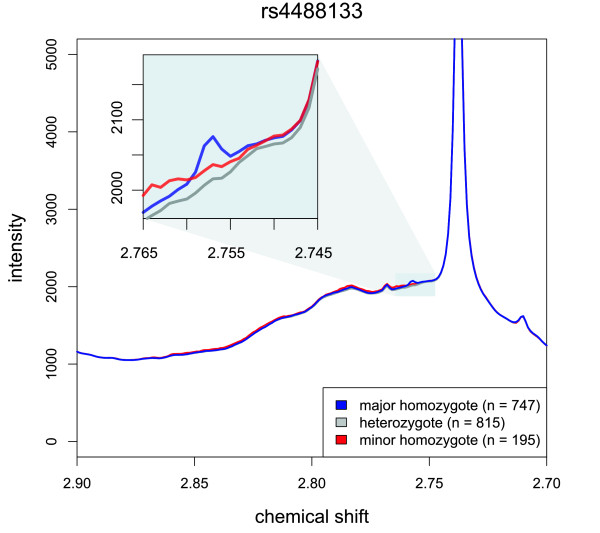
**Association at the PYROXD2 locus**. SNP rs4488133 associates with the ratio between two neighboring chemical shifts (δ = 2.757 ppm and δ = 2.755 ppm; *P *= 2.9x10^-94^), displaying an exceptionally strong increase in the strength of association when using ratios of over 82 orders of magnitude. This figure presents the median NMR signal intensities, differentiated by the genotype of SNP rs4488133 (color-coded). A peak at δ = 2.757 ppm is only present in major allele homozygotes (inset). This peak is located on the shoulder of a much larger underlying NMR signal. We argue that in this case the ratio between signals at two neighboring chemical shifts, one of them in a localized peak, is equivalent to applying a local baseline correction, effectively elevating the peak out of the background noise.

We find an association between rs4488133 in PYROXD2 and the signal intensities at δ = 2.757 ppm. With a *P *value of 7.3×10^-12^, this association only slightly misses the threshold for genome-wide significance. At the given chemical shift, we do not find any noteworthy correlation to one of the known MS determined metabolites. Most interestingly, similar to the FADS1 case, this locus displays an exceptionally increase in strength of associations when using ratios (>80 orders of magnitude). However, as opposed to the FADS1 locus, the ratio here is between two chemical shift positions in direct neighborhood (δ = 2.757 ppm and 2.755 ppm). An inspection of the detailed spectrum differentiated by genotype reveals that only the signal at δ = 2.757 ppm actually shows clear genotype dependence, with a peak only detected in the major allele homozygote group (Figure 4). This peak is located on the shoulder of a much larger underlying NMR signal that correlates with cholesterol levels. We argue that in this case the ratio between signals at two neighboring chemical shifts, one of them in a localized peak, is equivalent to applying a local baseline correction, thus elevating the peak out of the background noise. Wei *et al. *have described this effect in a recent publication where they present a ratio-based approach that effectively raises the signal-to-noise ratio in NMR spectra [28].

Since PYROXD2 is a relatively uncharacterized genetic locus, combining the NMR ratio presented here with findings from other studies and platforms could be used to further investigate its biological function.

## Conclusions

In this study, we identify seven ratios of NMR signal intensities at different chemical shift positions that associate with genetic loci at a genome-wide significance level. Compared to the genetic associations to signal intensities at individual chemical shift positions, the strength of association increased in the case of CEPT, GCKR, CPS1, and APOA1 by at least three orders of magnitude. In the case of FADS1 and PYROXD2 it even increased by 60 and 80 orders of magnitude (Table 1). There are different reasons for this increase in the strength of association: In the case of the FADS1 and GCKR loci, the signal ratios most likely represent the ratio between two distinct metabolites or metabolite classes, while in the case of PYROXD2, ratios may compensate for overlapping NMR signals originating from other metabolites, thereby acting as a local baseline correction.

More generally, we have shown that the use of signal ratios is a simple method to derive genetically validated bi-variate measures or biomarkers from NMR spectra. This approach could potentially be generalized to the multi-variate case, again using genetic association as a criterion for feature selection.

**Figure 5:**
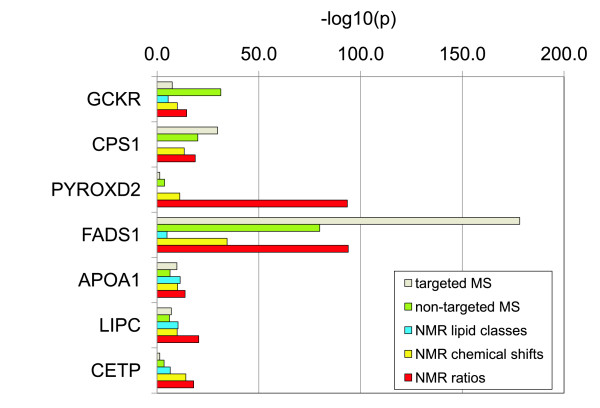
**Strength of association with metabolic traits obtained using different methods and technologies**. Samples from identical blood draws were analyzed using different methods. Identical genotype data were used and the number of samples is the same (except for minor differences due to missing data); height of the colored bars represents the strength of association for the different methods as reported in Table 2 (grey: targeted MS [3,4], green: non-targeted MS [5], blue: NMR lipid classes [13], yellow: NMR chemical shifts (bins), and red: NMR ratios (this work)).

When we compare our findings to association data that could be obtained by other techniques on the same samples, an increased strength of association was found in four out of seven cases (Table 2 and Figure 5). In the other three cases (FADS1, GCKR, and CPS1), the MS-based techniques clearly outperform our approach, showing again the complementary character of both platforms. However, since NMR is in general more adapted to clinical application, and also since the computation of ratios from NMR spectra is easy and straightforward, the ratios we identify here have the potential of representing easy-to-use disease biomarkers for tests in future clinical studies. Also, our approach to annotate NMR signals using correlations with MS determined metabolite concentrations could be used to transfer knowledge between different platforms.

## Abbreviations

APOA1: Apolipoprotein A1; C(OH)x:y: Hydroxylacylcarnitine (x = number of carbons in fatty acid side chain, y = number of double bonds in fatty acid side chain); C0: Free carnitine; CETP: Cholesterylester transfer protein; CEU: Utah Residents with Northern and Western European Ancestry; CHR: Chromosome; CPS1: Carbamoyl-phosphate synthetase 1; CV: Coefficient of variation; Cx:y: Acylcarnitine (x = number of carbons in fatty acid side chain, y = number of double bonds in fatty acid side chain); Cx:y-DC: Dicarboxylacylcarnitine (x = number of carbons in fatty acid side chain, y = number of double bonds in fatty acid side chain); DNA: Deoxyribonucleic acid; DSS: 4,4-dimethyl-4-silapentane-1-sulfonic acid; EDTA: Ethylene diaminetetraacetic acid; ESI: Electro spray ionization; FADS1: Delta-5 fatty acid desaturase 1; FID: Free induction decay; GC: Gas chromatography; GCKR: Glucokinase regulator; GIM: Genetically influenced metabotype; GWAS: Genome-wide association study; H1: Hexose; HLD-C: High-density lipoprotein cholesterol; HMDB: Human Metabolome Database; IDL: Intermediate-density lipoprotein; KORA: Kooperative Gesundheitsforschung in der Region Augsburg (Cooperative Health Research in the Region of Augsburg); LC: Liquid chromatography; LD: Linkage disequilibrium; LDL-C: Low-density lipoprotein cholesterol; LIPC: Hepatic lipase; LIT: Linear ion trap; m/z: Mass-to-charge ratio; MAF: Minor allele frequency; MS: Mass spectrometry; NCBI: National Center for Biotechnology Information; NMR: Nuclar magnetic resonance spectroscopy; PC aa Cx:y: Diacyl phosphatidylcholine (x = number of carbons in fatty acid side chain, y = number of double bonds in fatty acid side chain); PC ae Cx:y: Acyl-alkyl phosphatidylcholine(x = number of carbons in fatty acid side chain, y = number of double bonds in fatty acid side chain); POS: Position; ppm: Parts per million; PUFA: Polyunsaturated fatty acid; PYROXD2: Pyridine nucleotide-disulphide oxidoreductase domain 2; Q-Q: Quantile-quantile; SM(OH) Cx:y: N-hydroxylacyloylsphingosylphosphocholine (x = number of carbons in fatty acid side chain, y = number of double bonds in fatty acid side chain); SM Cx:y: Sphingomyelin (x = number of carbons in fatty acid side chain, y = number of double bonds in fatty acid side chain); SNP: Single nucleotide polymorphism; TC: Total cholesterol; TG: Triglyceride; UHPLC: Ultrahigh performance liquid chromatography; VLDL: Very low-density lipoprotein.

## Competing interests

FB and PP are employed by numares GmbH. They contributed only to logistics, optimization of NMR spectroscopy, and to NMR data interpretation. numares was not involved in the design of the study, statistical analyses, or interpretation of the results. All other authors declare that they have no competing interests.

## Authors' contributions

Designed the study and wrote the paper: GK, JR, WR, KSu Contributed to data analysis and/or interpretation: JA, FB, CG, CH, TI, CM, AP, PP, KSt, HW Provided material and/or data: JA, CG, CH, TI, CM, HW All authors read and approved the final manuscript.

## Supplementary Material

Additional file 1 **Regional association plots, box plots, histograms, and quantile-quantile plots for the genetic associations and NMR traits reported in Table 1**. Top: Regional association plots based on the SNPs that were used in our GWAS. Gene annotations and SNP positions are based on human genome hg18 (NCBI 36.1); linkage equilibrium correlation coefficients (r^2^) are based on Hapmap, release 21. Bottom left: Box plots of NMR signal intensities or NMR ratios for each genotype (in order major allele homozygotes, heterozygotes, minor allele homozygotes). The number of samples per group is indicated below the plot. Data are presented on a log_10_-normal scale. Bottom center: Histograms for NMR signal intensities or NMR ratios. The blue line and blue boxes indicate the distribution of the log_10_-scaled data, the red line indicates a normal distribution with the same mean and standard deviation as found in the log_10_-scaled data. Bottom right: Q-Q plots showing the observed *versus *the theoretically expected distribution of the associations' *P *values for all tested SNPs to the given NMR bin or NMR ratio. Click here for file

Additional file 2 **Spearman correlation plots between NMR chemical shifts and metabolite concentrations**. The plots correspond to the correlations between metabolites and chemical shifts reported in Additional file 3, Table S1. For some metabolites, reference NMR spectra of the pure compound wereavailable as Free Induction Decay (FID) files from HMDB [18]. In these cases, the reference spectra are plotted below the corresponding correlation spectra. Note that in comparison with the correlation spectra, the peaksin the HMDB spectra may be shifted due to different experimental conditions such as sample pH and calibration to a different reference compound. Click here for file

Additional file 3 **Table S1. Spearman correlations between NMR intensities and metabolite concentrations measured on different platforms**. The 'Chemical Shift' column lists the position of the signal intensities that show the best correlation (for r_s_^2^≥0.20) with the chemical compound noted in the 'Metabolite' column. '▲' 'indicates positive correlation, '▼' anticorrelation. 'N' is the number of samples used for calculating the correlations where valid NMR data points and metabolite concentrations were jointly available.'CV' gives the coefficient of variation in the quality control samples, where available. Chemical shifts are reported in parts per million (ppm).**Table S2. Non-parametric tests for genetic associations with NMR bins and NMR ratios**. All associations listed in Table 1 were tested separately using a non-parametric test. To test for associations between a SNP and an NMR trait (individual chemical shift or ratio between intensities at two different chemical shifts), Spearman's rho statistic was used; the resulting *P *values are given as p_s_. For comparison, the *P *values of the age- and gender-corrected linear models are given as p_lm_. Chemical shifts are reported in parts per million (ppm). See Table 1 for details about the SNPs and the number of tested traits for each variant. Click here for file
