# Supplementary material for: Identification and MS-assisted interpretation of genetically influenced NMR signals in human plasma
Source: Genome Med. 2013 Feb 15;5(2):13. doi: 10.1186/gm417 (PMC3706909; doi:10.1186/gm417)
Supplement: Additional file 1 — Regional association plots, box plots, histograms, and quantile-quantile plots for the genetic associations and NMR traits reported in Table 1. Top: Regional association plots based on the SNPs that were used in our GWAS. Gene annotations and SNP positions are based on human genome hg18 (NCBI 36.1); linkage equilibrium correlation coefficients (r2) are based on Hapmap, release 21. Bottom left: Box plots of NMR signal intensities or NMR ratios for each genotype (in order major allele homozygotes, heterozygotes, minor allele homozygotes). The number of samples per group is indicated below the plot. Data are presented on a log10-normal scale. Bottom center: Histograms for NMR signal intensities or NMR ratios. The blue line and blue boxes indicate the distribution of the log10-scaled data, the red line indicates a normal distribution with the same mean and standard deviation as found in the log10-scaled data. Bottom right: Q-Q plots showing the observed versus the theoretically expected distribution of the associations' P values for all tested SNPs to the given NMR bin or NMR ratio. [file gm417-S1.PDF]

KORA F4 Plasma NMR (GCKR locus)

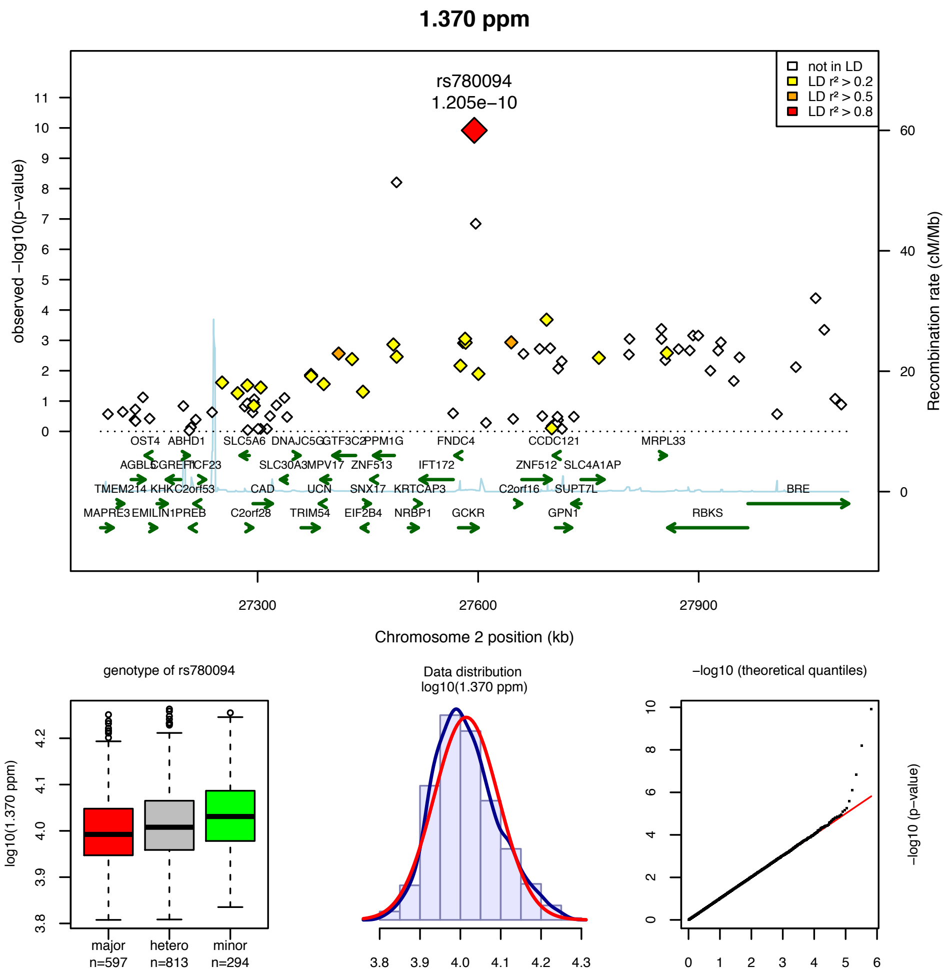

KORA F4 Plasma NMR (GCKR locus)

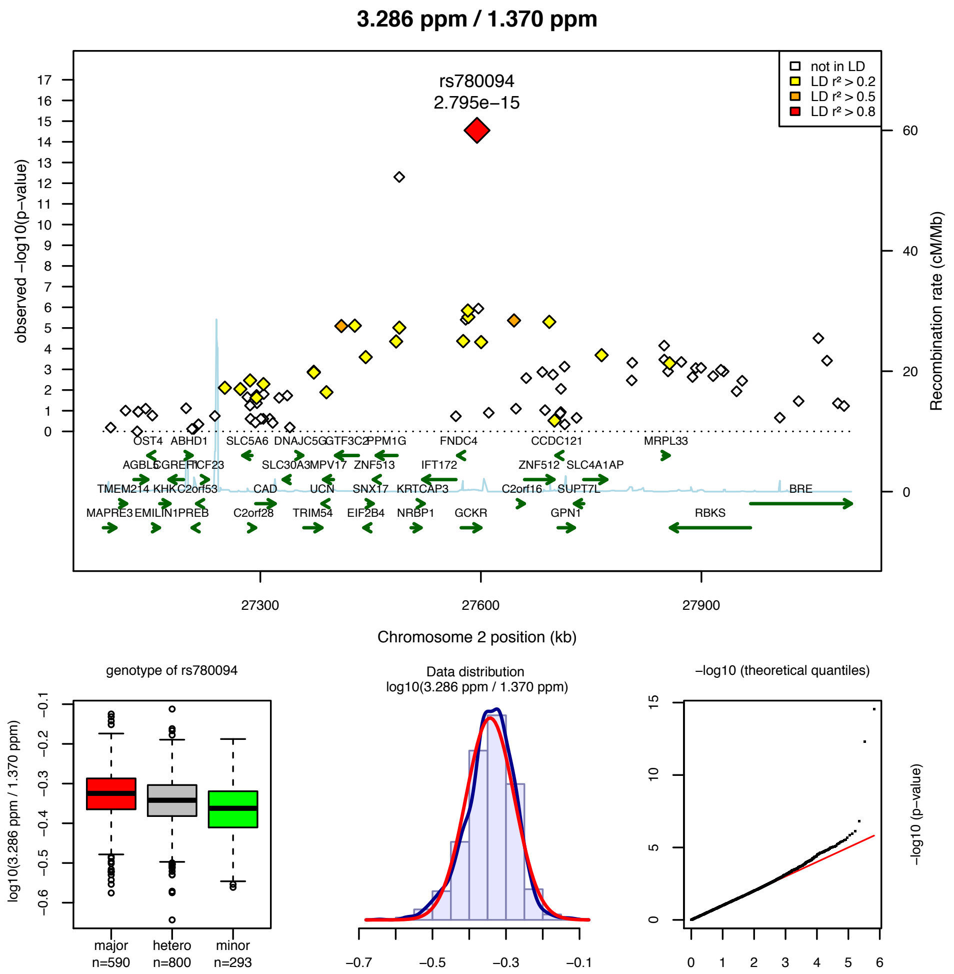

KORA F4 Plasma NMR (CPS1 locus)

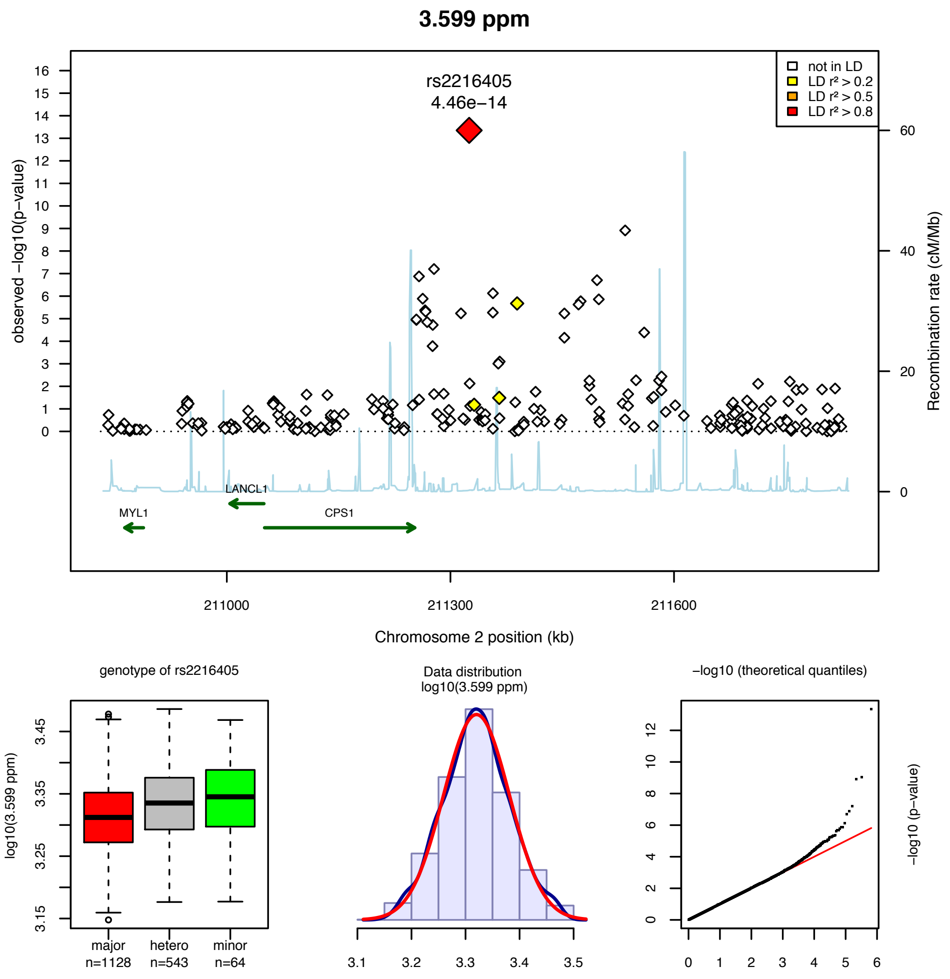

KORA F4 Plasma NMR (CPS1 locus)

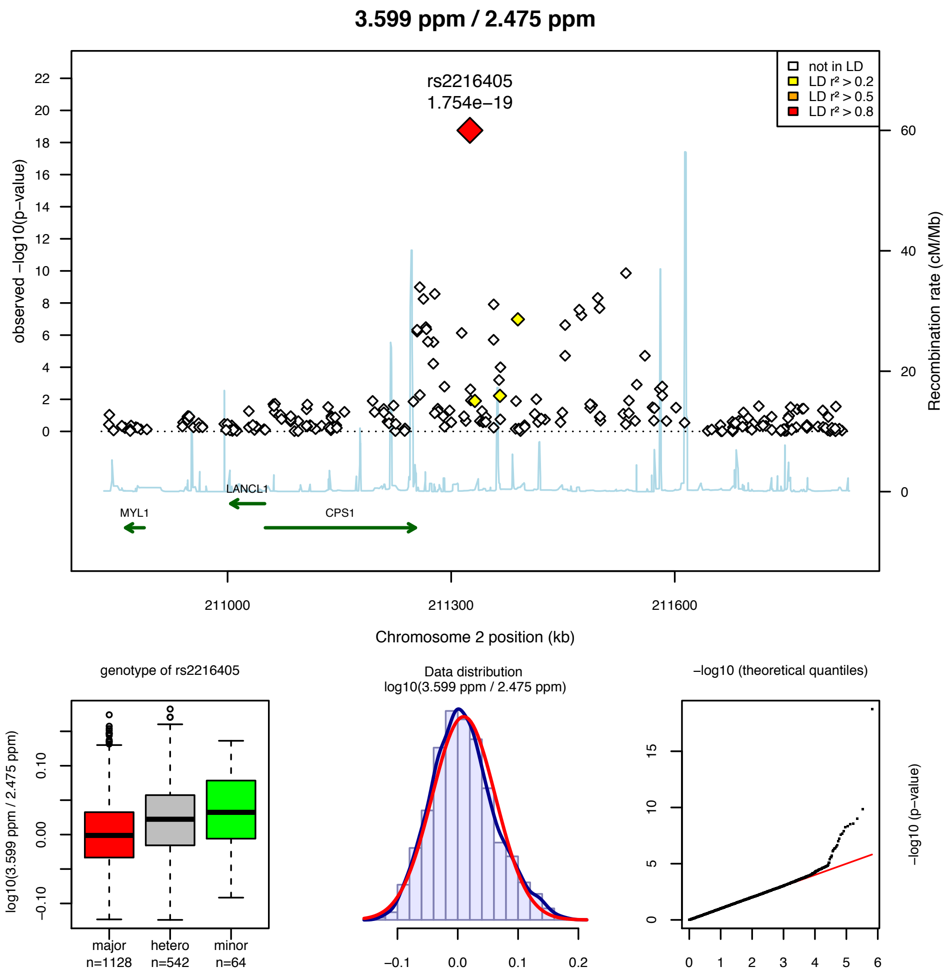

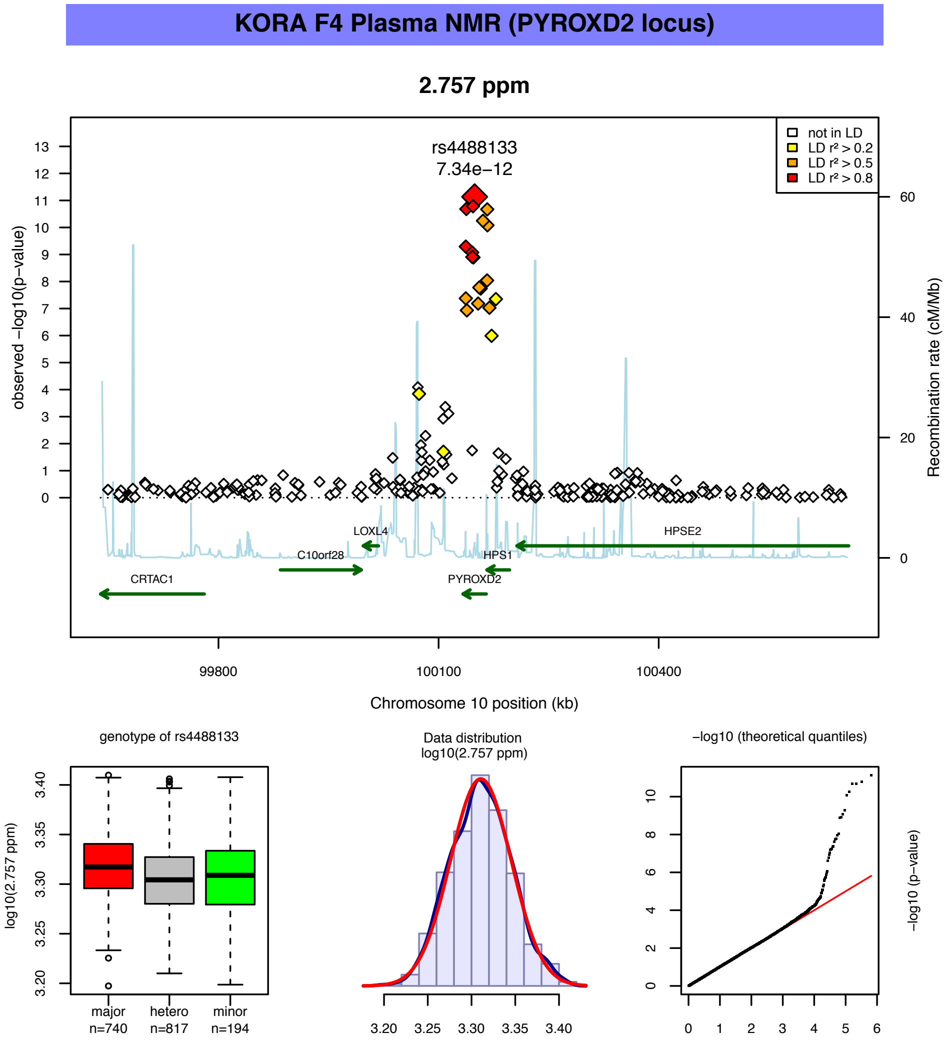

KORA F4 Plasma NMR (PYROXD2 locus)
2.757 ppm
rs4488133
7.34e−12
not in LD
LD r² > 0.2
LD r² > 0.5
LD r² > 0.8
observed −log10(p−value)
Recombination rate (cM/Mb)
LOXL4
C10orf28
HPS1
HPSE2
CRTAC1
PYROXD2
99800
100100
100400
Chromosome 10 position (kb)
genotype of rs4488133
log10(2.757 ppm)
major n=740
hetero n=817
minor n=194
Data distribution
log10(2.757 ppm)
−log10 (theoretical quantiles)
−log10 (p−value)

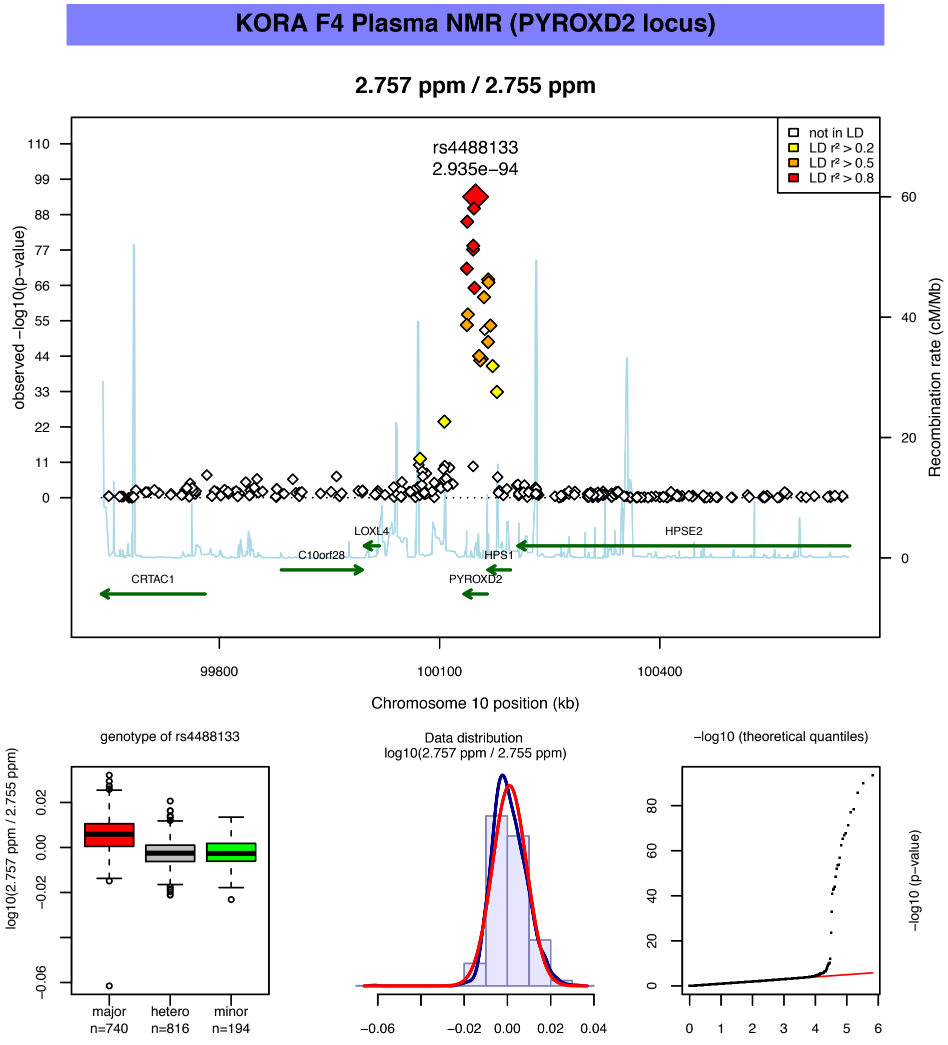

KORA F4 Plasma NMR (PYROXD2 locus)
2.757 ppm / 2.755 ppm
rs4488133
2.935e−94
not in LD
LD r² > 0.2
LD r² > 0.5
LD r² > 0.8
observed −log10(p−value)
Recombination rate (cM/Mb)
LOXL4
C10orf28
HPS1
HPSE2
CRTAC1
PYROXD2
99800
100100
100400
Chromosome 10 position (kb)
genotype of rs4488133
log10(2.757 ppm / 2.755 ppm)
major n=740
hetero n=816
minor n=194
Data distribution
log10(2.757 ppm / 2.755 ppm)
−log10 (theoretical quantiles)
−log10 (p−value)

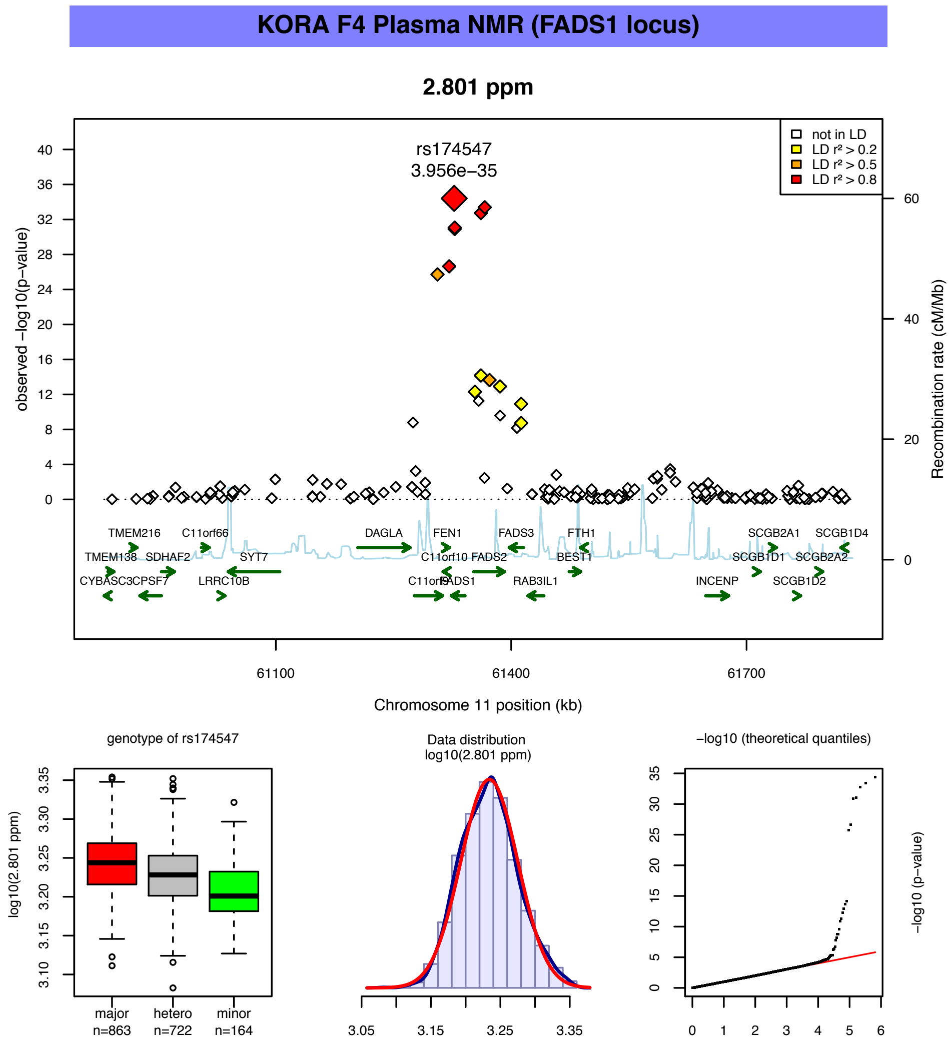
KORA F4 Plasma NMR (FADS1 locus)
2.801 ppm
rs174547
3.956e−35
not in LD
LD r² > 0.2
LD r² > 0.5
LD r² > 0.8
observed −log10(p−value)
Recombination rate (cM/Mb)
Chromosome 11 position (kb)
TMEM216
C11orf66
DAGLA
FEN1
FADS3
FTH1
SCGB2A1
SCGB1D4
TMEM138
SDHAF2
SYT7
C11orf10 FADS2
BEST1
SCGB1D1
SCGB2A2
CYBASC3CPSF7
LRRC10B
C11orf9ADS1
RAB3IL1
INCENP
SCGB1D2
genotype of rs174547
log10(2.801 ppm)
major
n=863
hetero
n=722
minor
n=164
Data distribution
log10(2.801 ppm)
−log10 (theoretical quantiles)
−log10 (p−value)

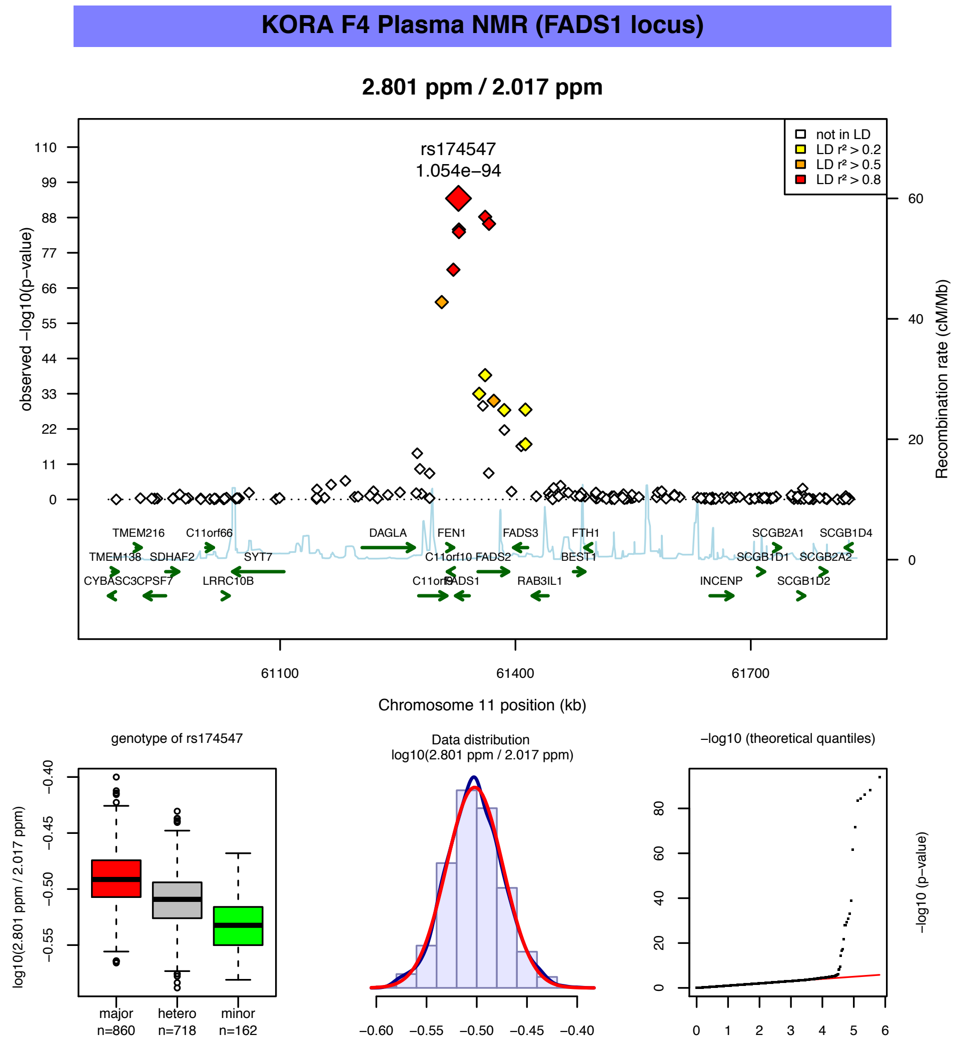
KORA F4 Plasma NMR (FADS1 locus)
2.801 ppm / 2.017 ppm
rs174547
1.054e−94
not in LD
LD r² > 0.2
LD r² > 0.5
LD r² > 0.8
observed −log10(p−value)
Recombination rate (cM/Mb)
Chromosome 11 position (kb)
TMEM216
C11orf66
DAGLA
FEN1
FADS3
FTH1
SCGB2A1
SCGB1D4
TMEM138
SDHAF2
SYT7
C11orf10 FADS2
BEST1
SCGB1D1
SCGB2A2
CYBASC3CPSF7
LRRC10B
C11orf9ADS1
RAB3IL1
INCENP
SCGB1D2
genotype of rs174547
log10(2.801 ppm / 2.017 ppm)
major
n=860
hetero
n=718
minor
n=162
Data distribution
log10(2.801 ppm / 2.017 ppm)
−log10 (theoretical quantiles)
−log10 (p−value)

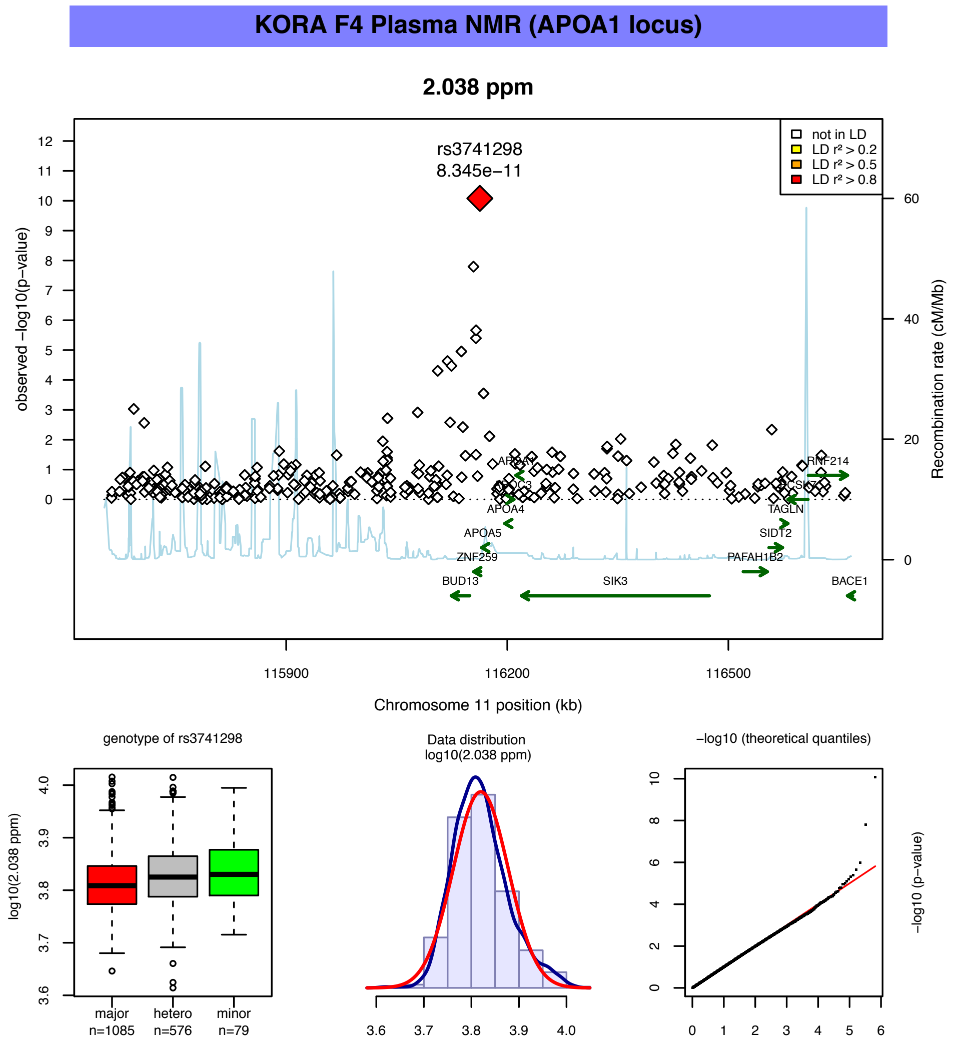
KORA F4 Plasma NMR (APOA1 locus)
2.038 ppm
rs3741298
8.345e−11
not in LD
LD r² > 0.2
LD r² > 0.5
LD r² > 0.8
observed −log10(p−value)
Recombination rate (cM/Mb)
APOA1
APOC3
APOA4
APOA5
ZNF259
BUD13
SIK3
RNF214
PCSK7
TAGLN
SIDT2
PAFAH1B2
BACE1
115900
116200
116500
Chromosome 11 position (kb)
genotype of rs3741298
log10(2.038 ppm)
major
n=1085
hetero
n=576
minor
n=79
Data distribution
log10(2.038 ppm)
−log10 (theoretical quantiles)
−log10 (p−value)

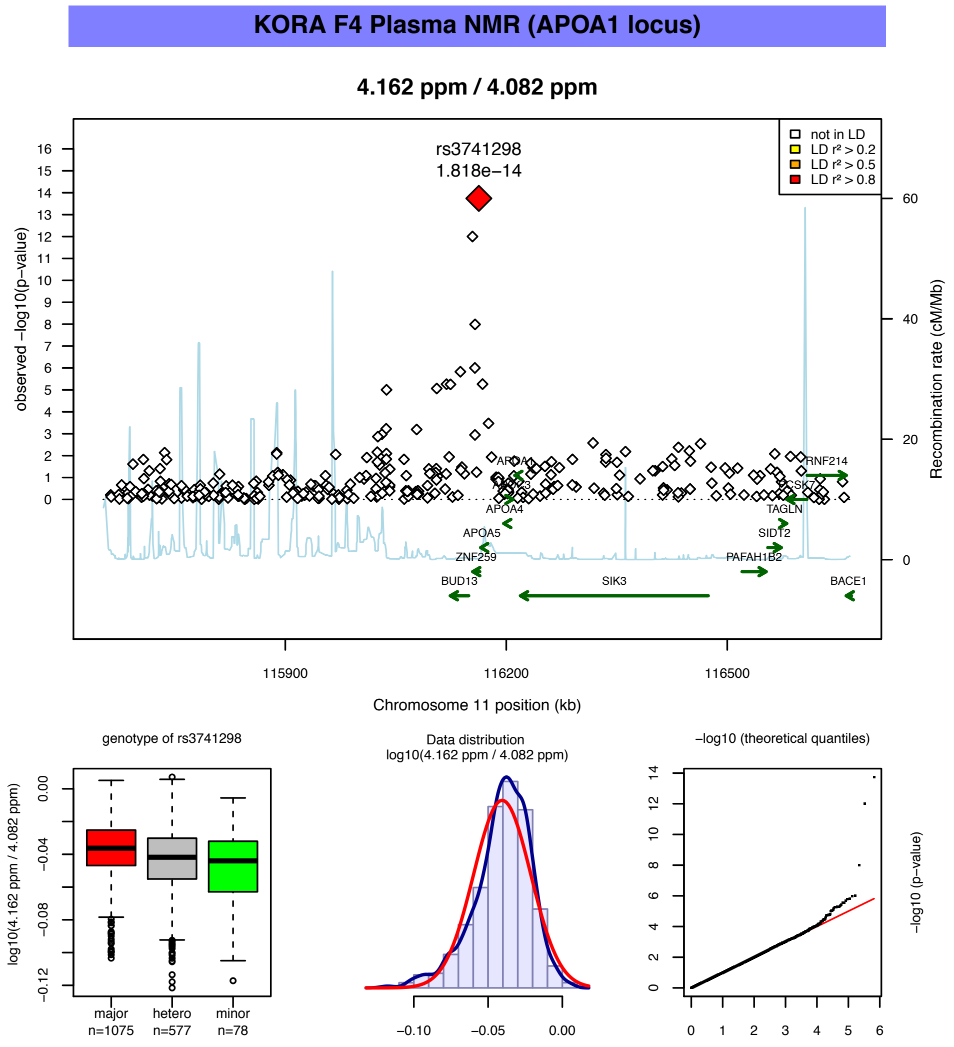
KORA F4 Plasma NMR (APOA1 locus)
4.162 ppm / 4.082 ppm
rs3741298
1.818e−14
not in LD
LD r² > 0.2
LD r² > 0.5
LD r² > 0.8
observed −log10(p−value)
Recombination rate (cM/Mb)
APOA1
APOC3
APOA4
APOA5
ZNF259
BUD13
SIK3
RNF214
PCSK7
TAGLN
SIDT2
PAFAH1B2
BACE1
115900
116200
116500
Chromosome 11 position (kb)
genotype of rs3741298
log10(4.162 ppm / 4.082 ppm)
major
n=1075
hetero
n=577
minor
n=78
Data distribution
log10(4.162 ppm / 4.082 ppm)
−log10 (theoretical quantiles)
−log10 (p−value)

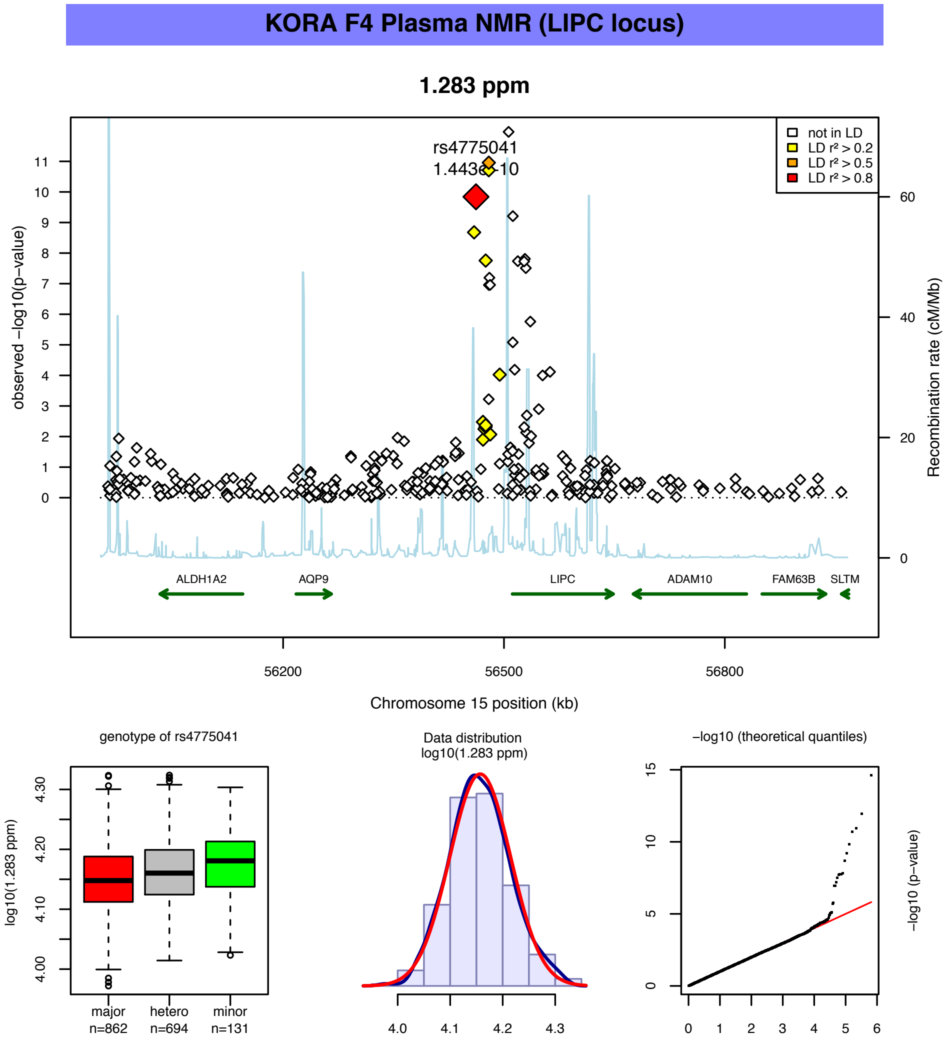
KORA F4 Plasma NMR (LIPC locus)
1.283 ppm
rs4775041
1.443e−10
not in LD
LD r² > 0.2
LD r² > 0.5
LD r² > 0.8
observed −log10(p−value)
Recombination rate (cM/Mb)
ALDH1A2
AQP9
LIPC
ADAM10
FAM63B
SLTM
56200
56500
56800
Chromosome 15 position (kb)
genotype of rs4775041
log10(1.283 ppm)
major
n=862
hetero
n=694
minor
n=131
Data distribution
log10(1.283 ppm)
−log10 (theoretical quantiles)
−log10 (p−value)

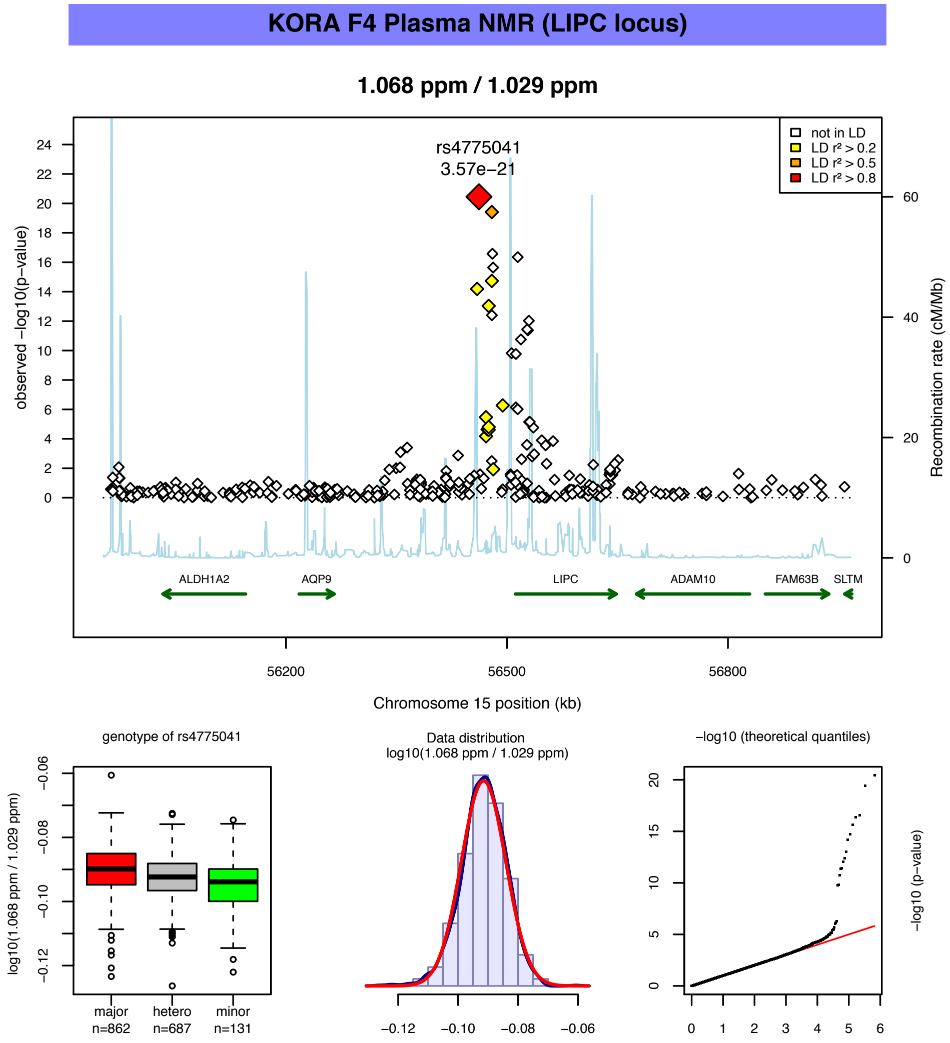
KORA F4 Plasma NMR (LIPC locus)
1.068 ppm / 1.029 ppm
rs4775041
3.57e−21
not in LD
LD r² > 0.2
LD r² > 0.5
LD r² > 0.8
observed −log10(p−value)
Recombination rate (cM/Mb)
ALDH1A2
AQP9
LIPC
ADAM10
FAM63B
SLTM
56200
56500
56800
Chromosome 15 position (kb)
genotype of rs4775041
log10(1.068 ppm / 1.029 ppm)
major
n=862
hetero
n=687
minor
n=131
Data distribution
log10(1.068 ppm / 1.029 ppm)
−log10 (theoretical quantiles)
−log10 (p−value)

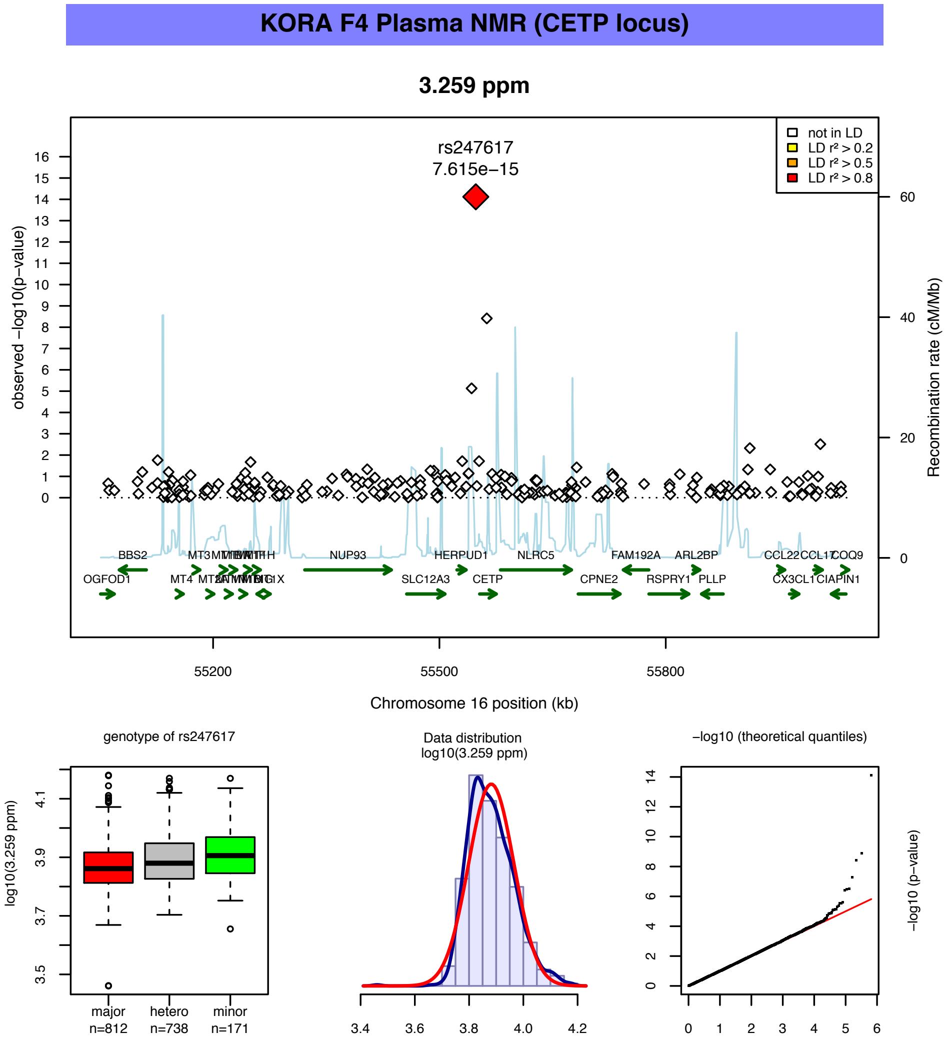
KORA F4 Plasma NMR (CETP locus)
3.259 ppm
rs247617
7.615e−15
not in LD
LD r² > 0.2
LD r² > 0.5
LD r² > 0.8
observed −log10(p-value)
Recombination rate (cM/Mb)
BBS2
OGFOD1
MT4
NUP93
HERPUD1
SLC12A3
CETP
NLRC5
CPNE2
FAM192A
RSPRY1
ARL2BP
PLLP
CCL22
CX3CL1
CIAPIN1
COQ9
55200
55500
55800
Chromosome 16 position (kb)
genotype of rs247617
log10(3.259 ppm)
major
n=812
hetero
n=738
minor
n=171
Data distribution
log10(3.259 ppm)
−log10 (theoretical quantiles)
−log10 (p−value)

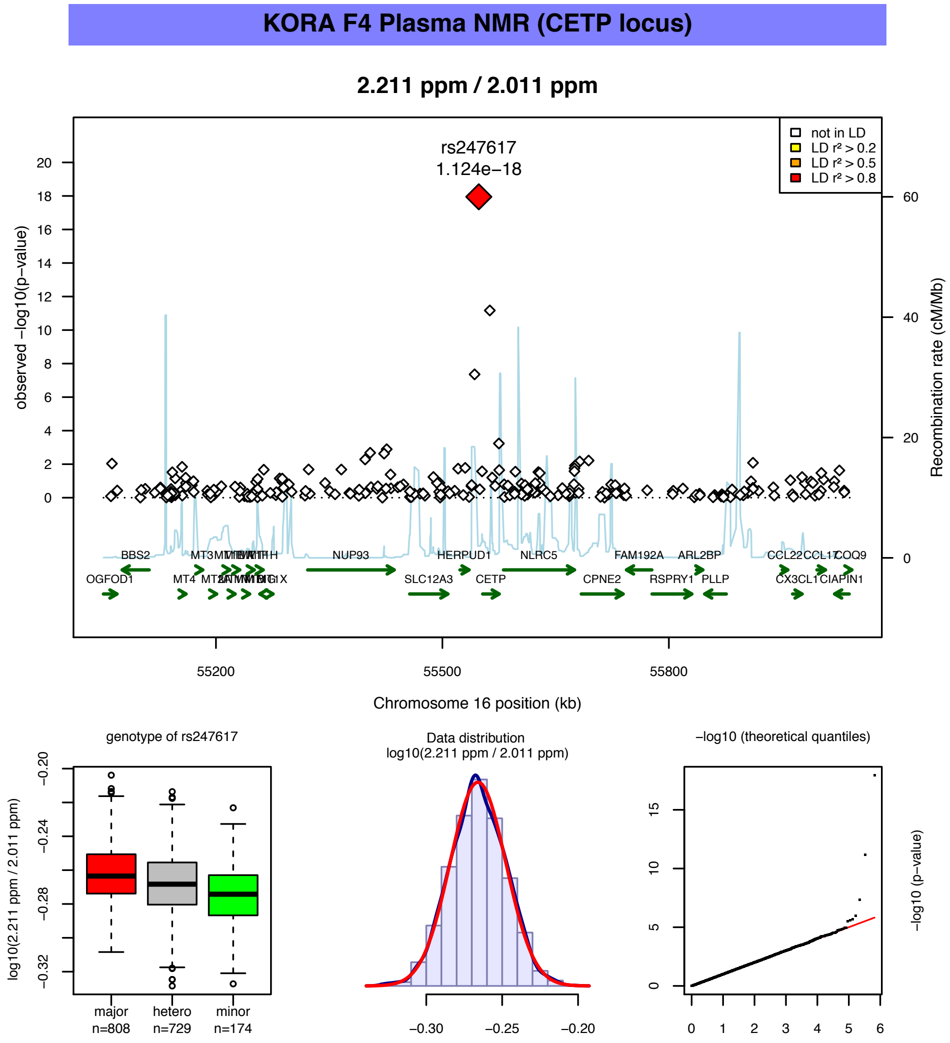
KORA F4 Plasma NMR (CETP locus)
2.211 ppm / 2.011 ppm
rs247617
1.124e−18
not in LD
LD r² > 0.2
LD r² > 0.5
LD r² > 0.8
observed −log10(p-value)
Recombination rate (cM/Mb)
BBS2
OGFOD1
MT4
NUP93
HERPUD1
SLC12A3
CETP
NLRC5
CPNE2
FAM192A
RSPRY1
ARL2BP
PLLP
CCL22
CX3CL1
CIAPIN1
COQ9
55200
55500
55800
Chromosome 16 position (kb)
genotype of rs247617
log10(2.211 ppm / 2.011 ppm)
major
n=808
hetero
n=729
minor
n=174
Data distribution
log10(2.211 ppm / 2.011 ppm)
−log10 (theoretical quantiles)
−log10 (p−value)
