# Supplementary material for: Identification and MS-assisted interpretation of genetically influenced NMR signals in human plasma
Source: Genome Med. 2013 Feb 15;5(2):13. doi: 10.1186/gm417 (PMC3706909; doi:10.1186/gm417)
Supplement: Additional file 2 — Spearman correlation plots between NMR chemical shifts and metabolite concentrations. The plots correspond to the correlations between metabolites and chemical shifts reported in Additional file 3, Table S1. For some metabolites, reference NMR spectra of the pure compound wereavailable as Free Induction Decay (FID) files from HMDB [18]. In these cases, the reference spectra are plotted below the corresponding correlation spectra. Note that in comparison with the correlation spectra, the peaksin the HMDB spectra may be shifted due to different experimental conditions such as sample pH and calibration to a different reference compound. [file gm417-S2.PDF]

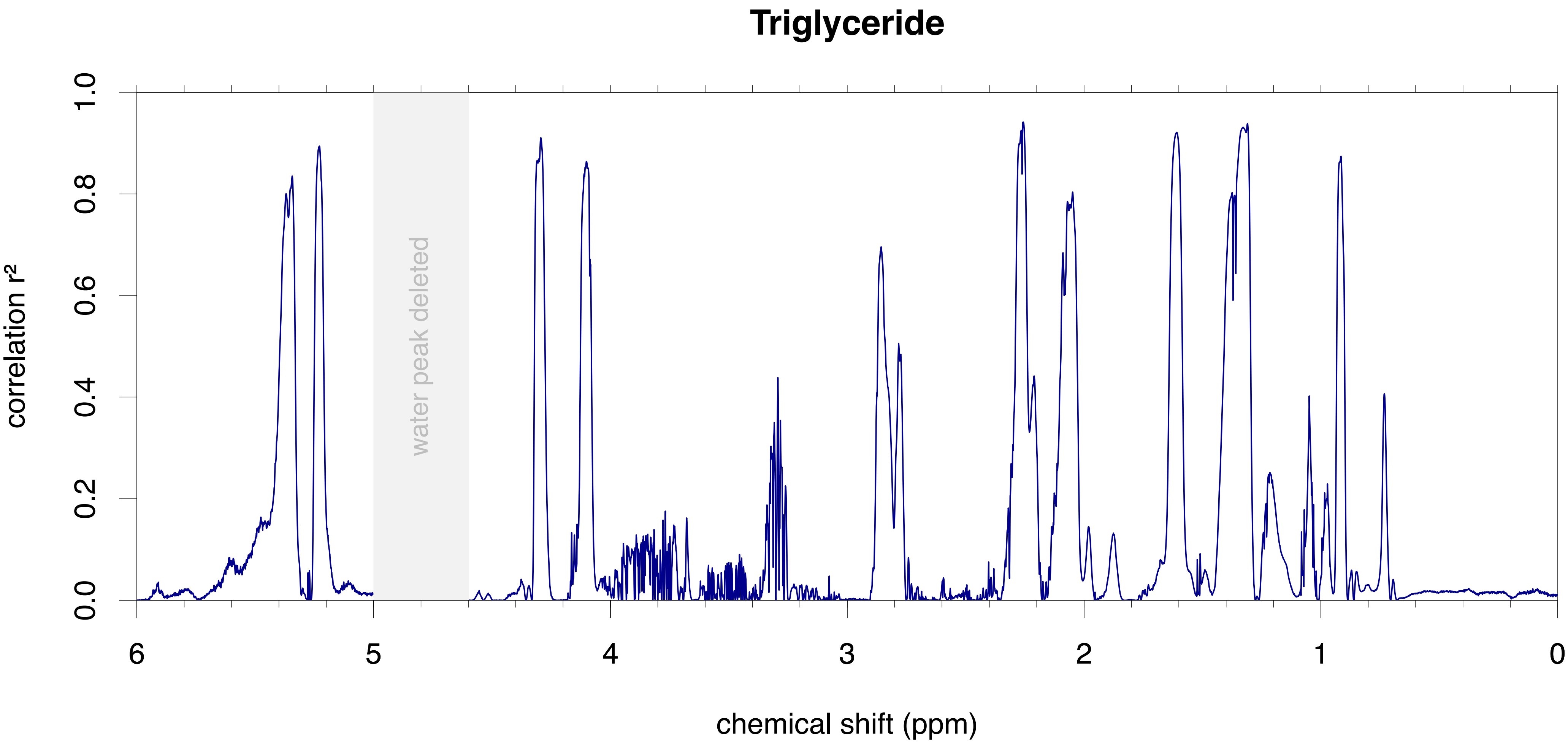

Triglyceride
correlation r²
0.0
0.2
0.4
0.6
0.8
1.0
water peak deleted
6
5
4
3
2
1
0
chemical shift (ppm)

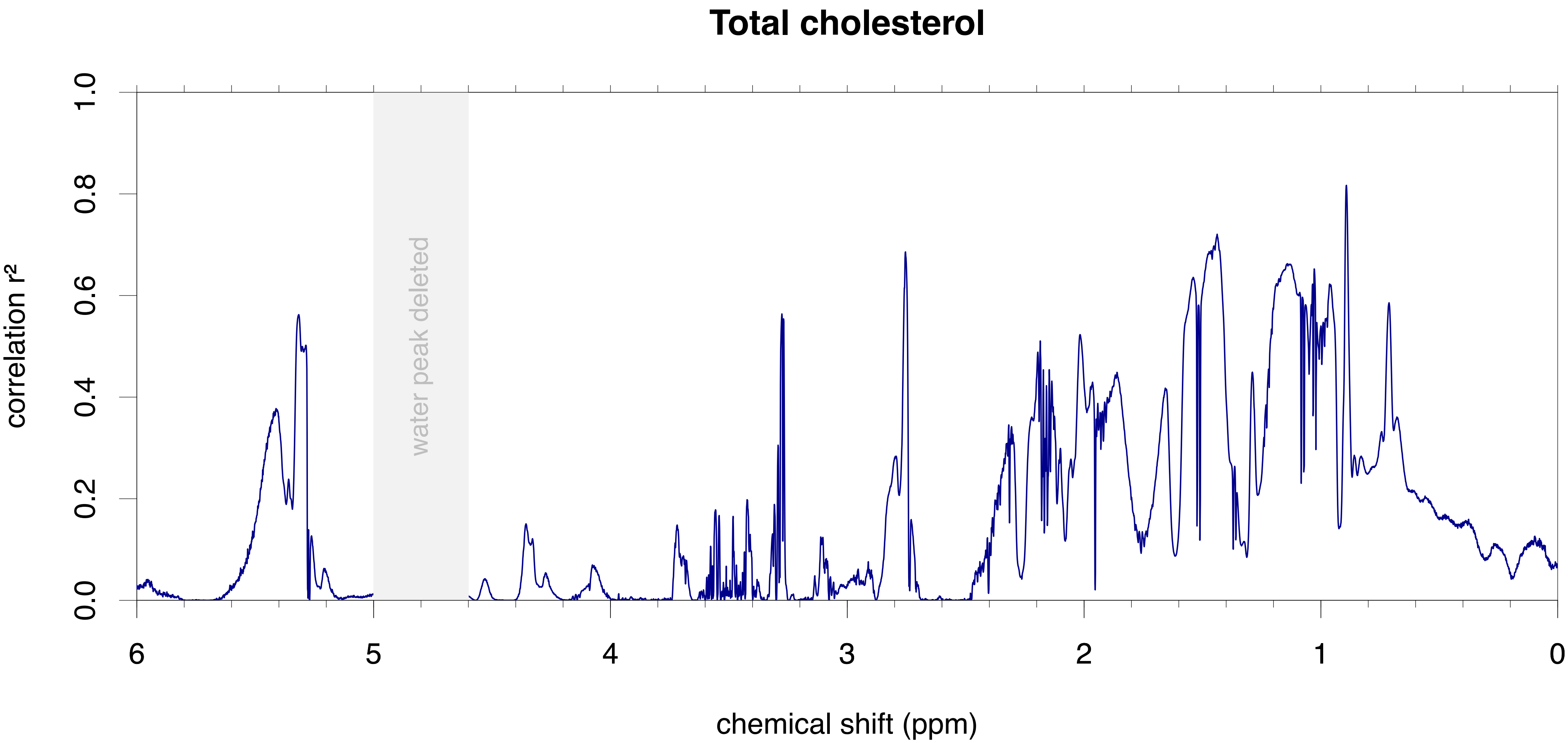
Total cholesterol
correlation r²
1.0
0.8
0.6
0.4
0.2
0.0
water peak deleted
6
5
4
3
2
1
0
chemical shift (ppm)

Cholesterol (HMDB)

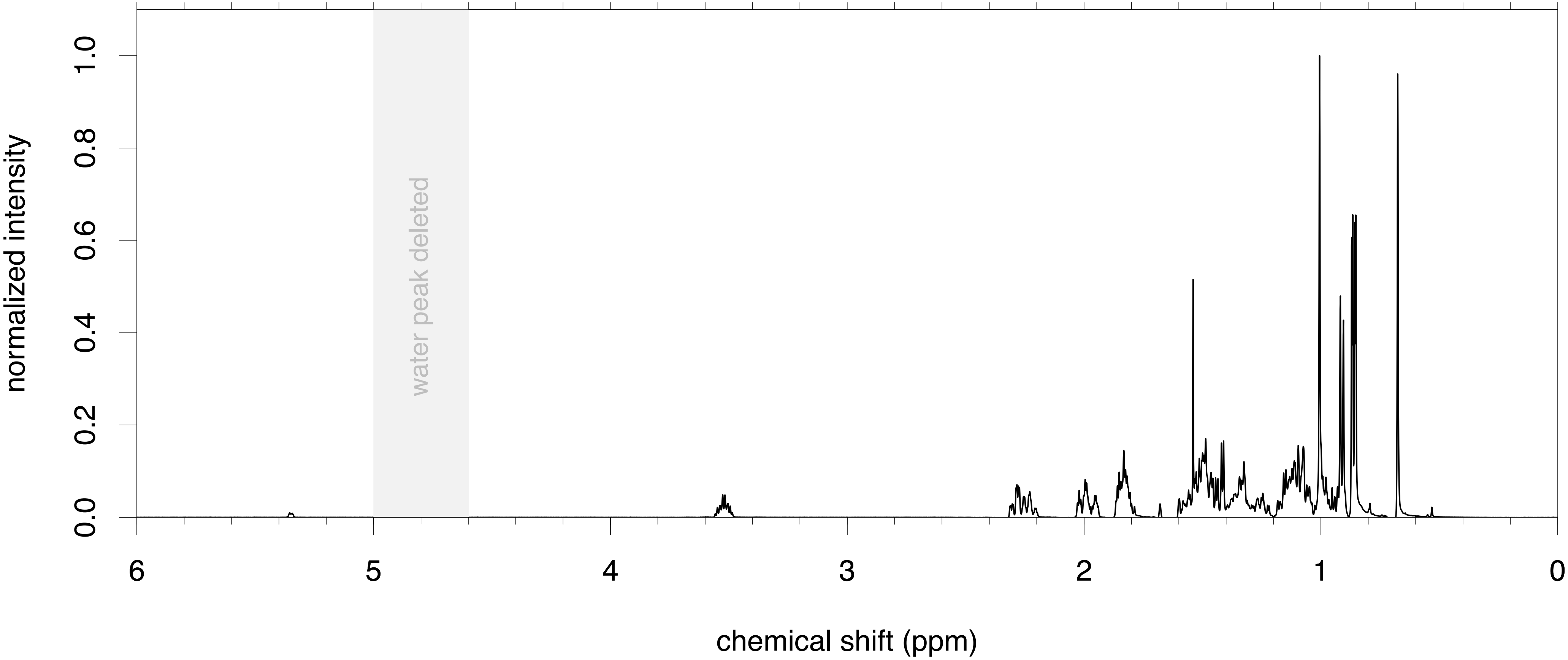

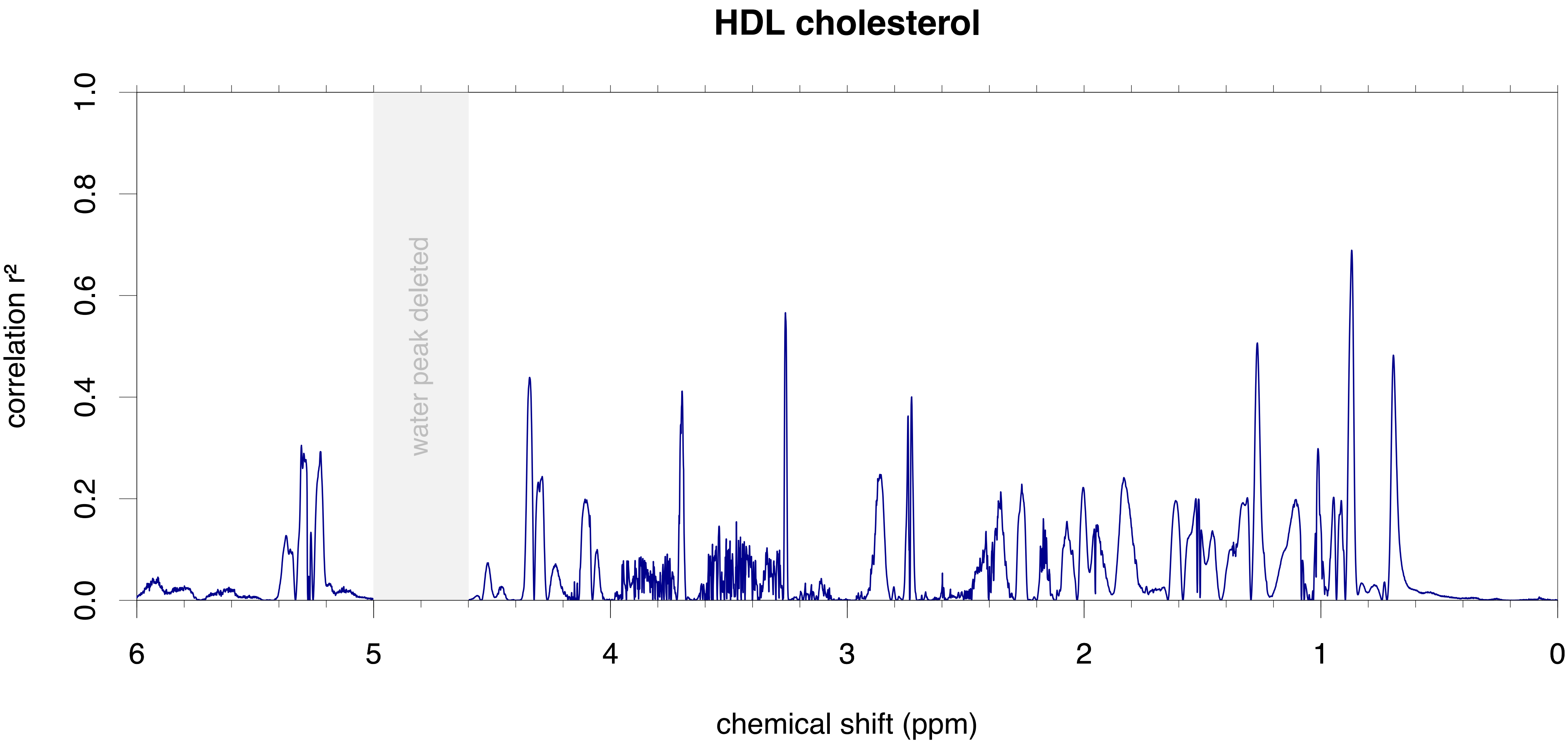
HDL cholesterol
correlation r²
1.0
0.8
0.6
0.4
0.2
0.0
water peak deleted
6
5
4
3
2
1
0
chemical shift (ppm)

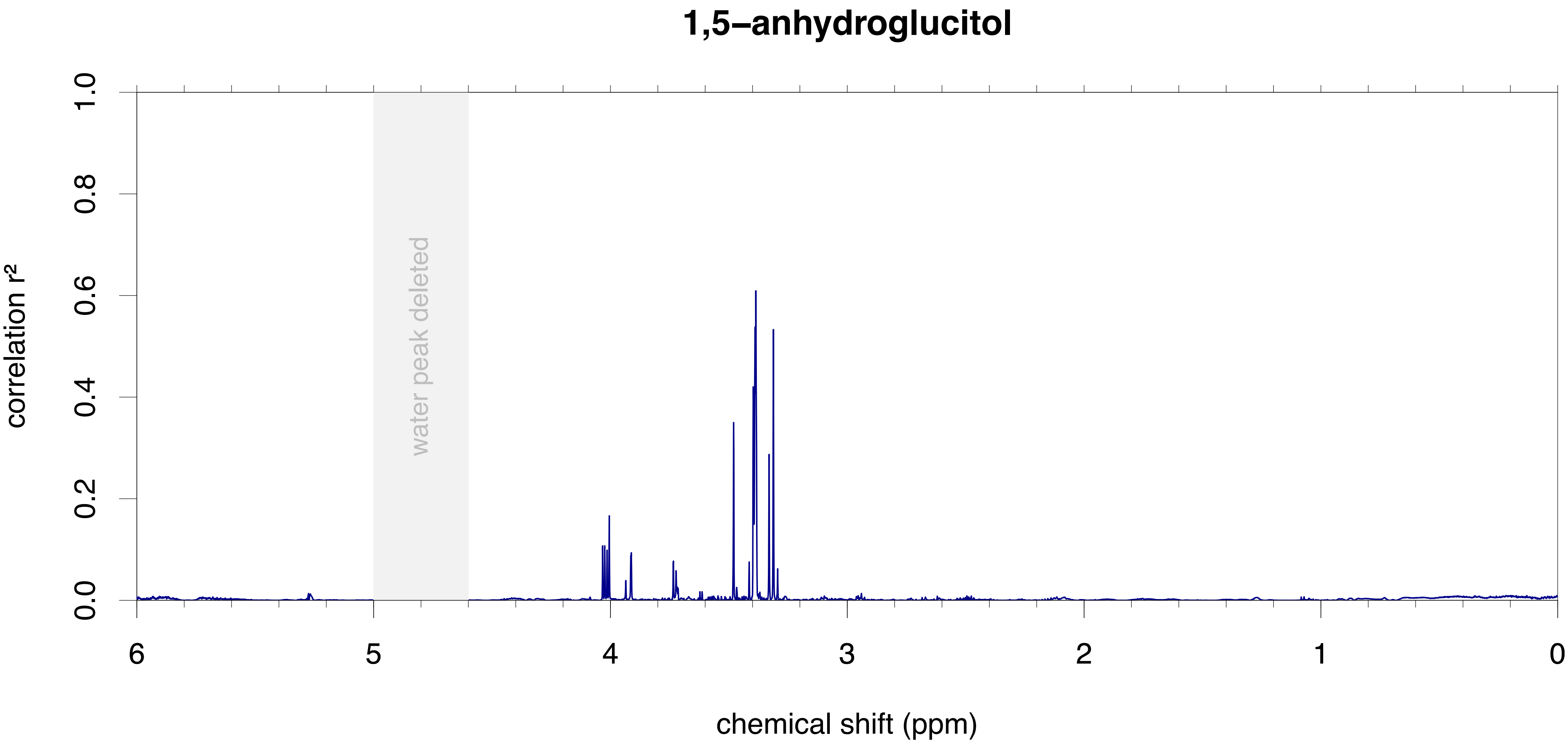

1,5−anhydroglucitol
correlation r²
0.0
0.2
0.4
0.6
0.8
1.0
water peak deleted
6
5
4
3
2
1
0
chemical shift (ppm)

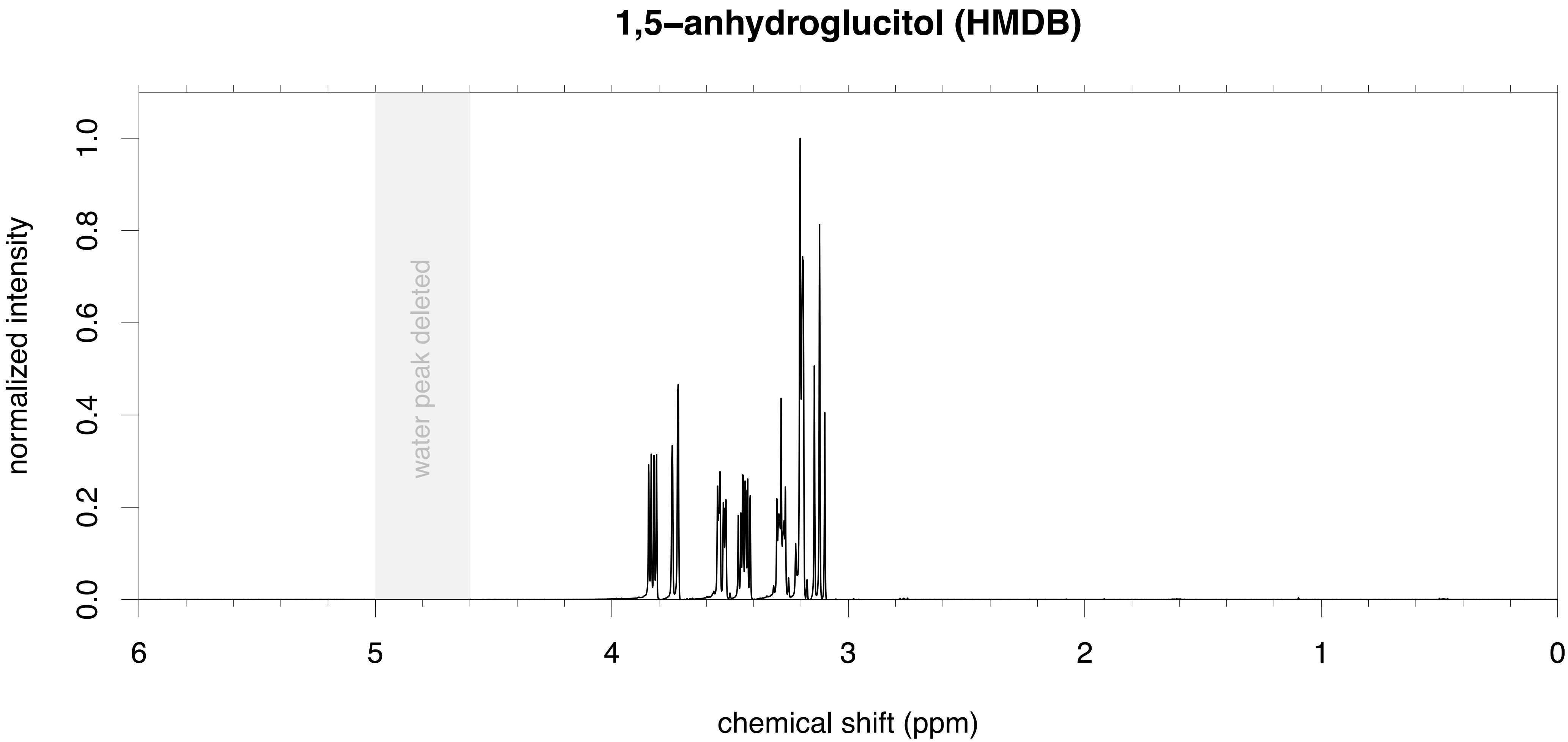

1,5–anhydroglucitol (HMDB)
normalized intensity
0.0
0.2
0.4
0.6
0.8
1.0
water peak deleted
6
5
4
3
2
1
0
chemical shift (ppm)

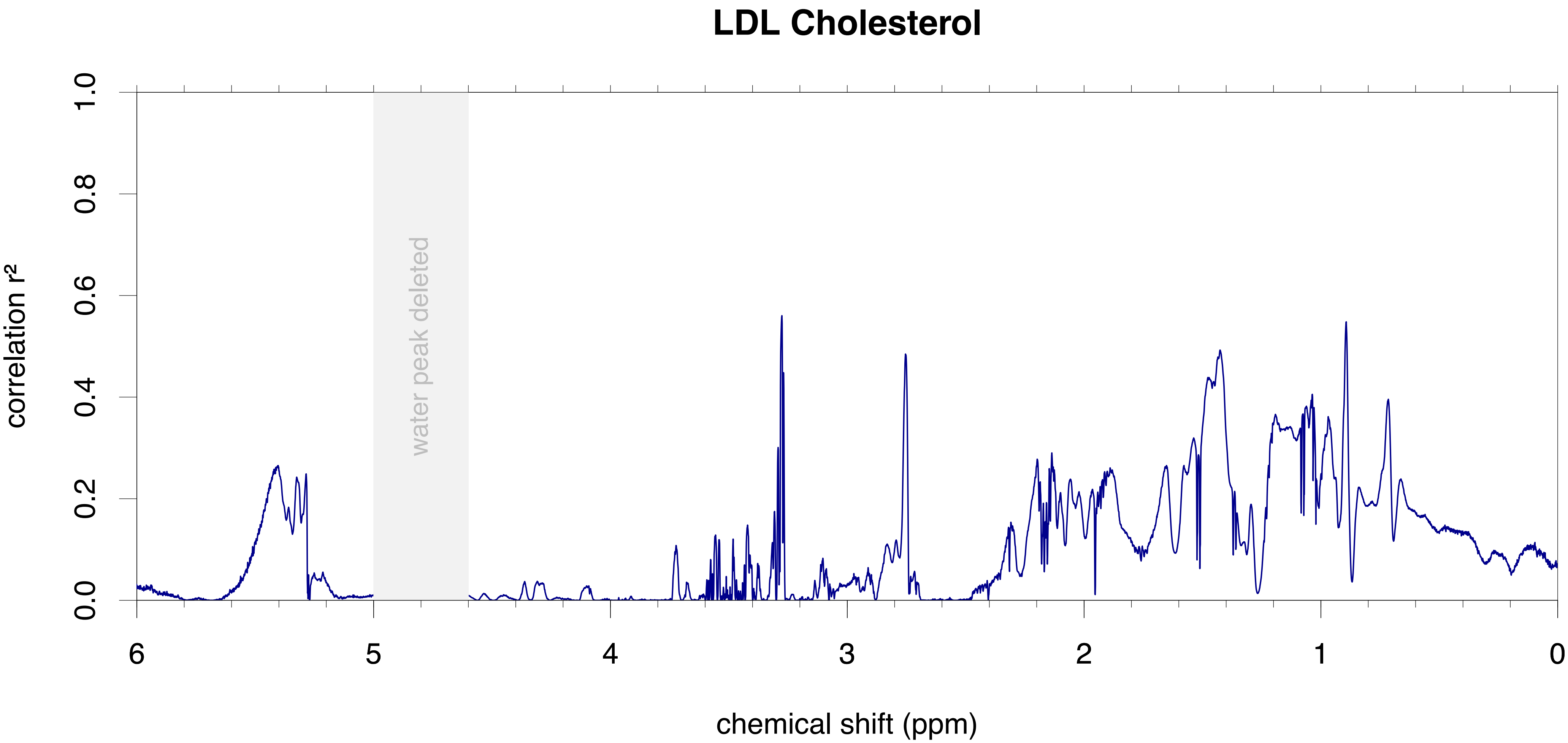

LDL Cholesterol
correlation r²
1.0
0.8
0.6
0.4
0.2
0.0
water peak deleted
6
5
4
3
2
1
0
chemical shift (ppm)

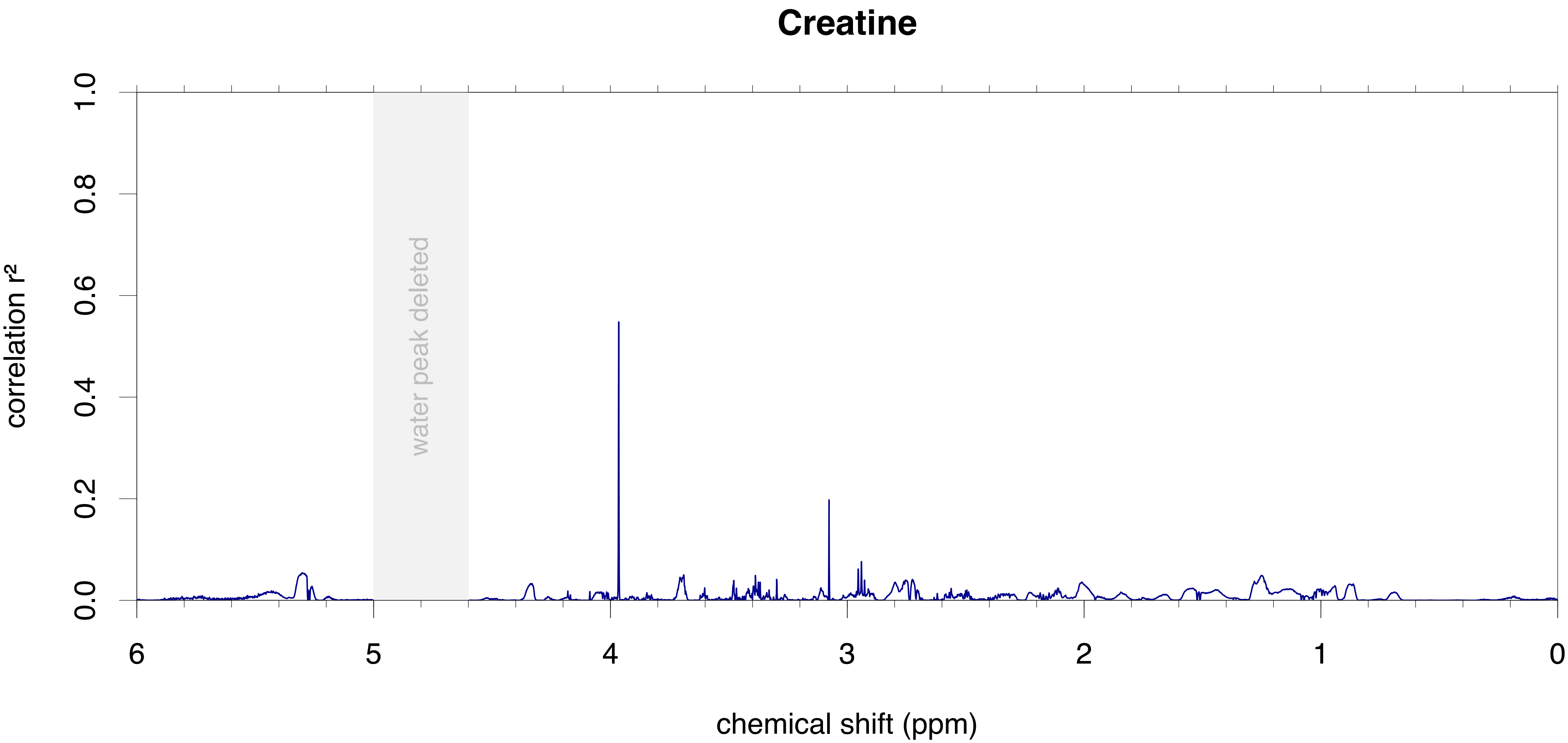

Creatine
water peak deleted
correlation r²
chemical shift (ppm)
0.0
0.2
0.4
0.6
0.8
1.0
6
5
4
3
2
1
0

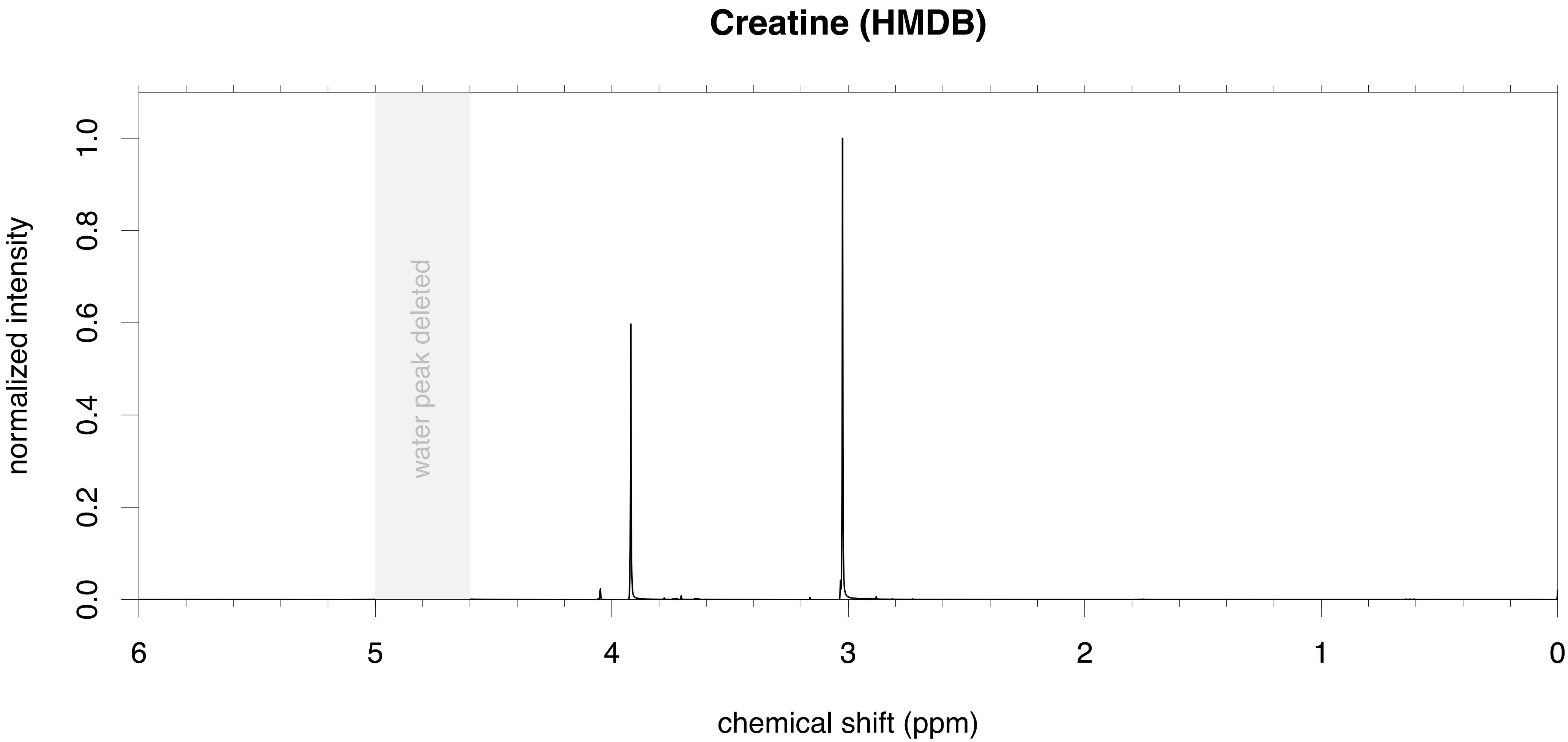
Creatine (HMDB)
normalized intensity
0.0
0.2
0.4
0.6
0.8
1.0
water peak deleted
6
5
4
3
2
1
0
chemical shift (ppm)

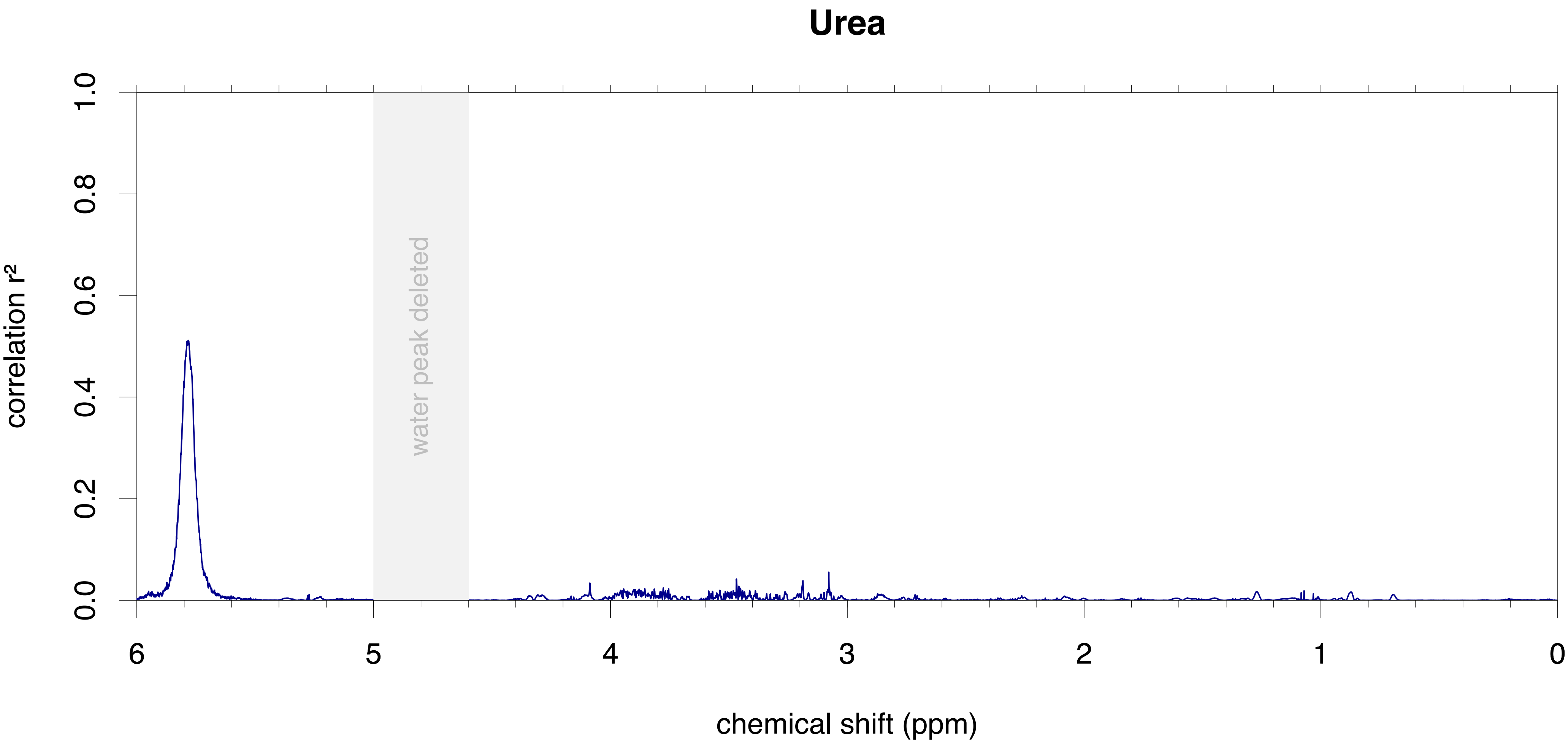

Urea
correlation r²
1.0
0.8
0.6
0.4
0.2
0.0
water peak deleted
6
5
4
3
2
1
0
chemical shift (ppm)

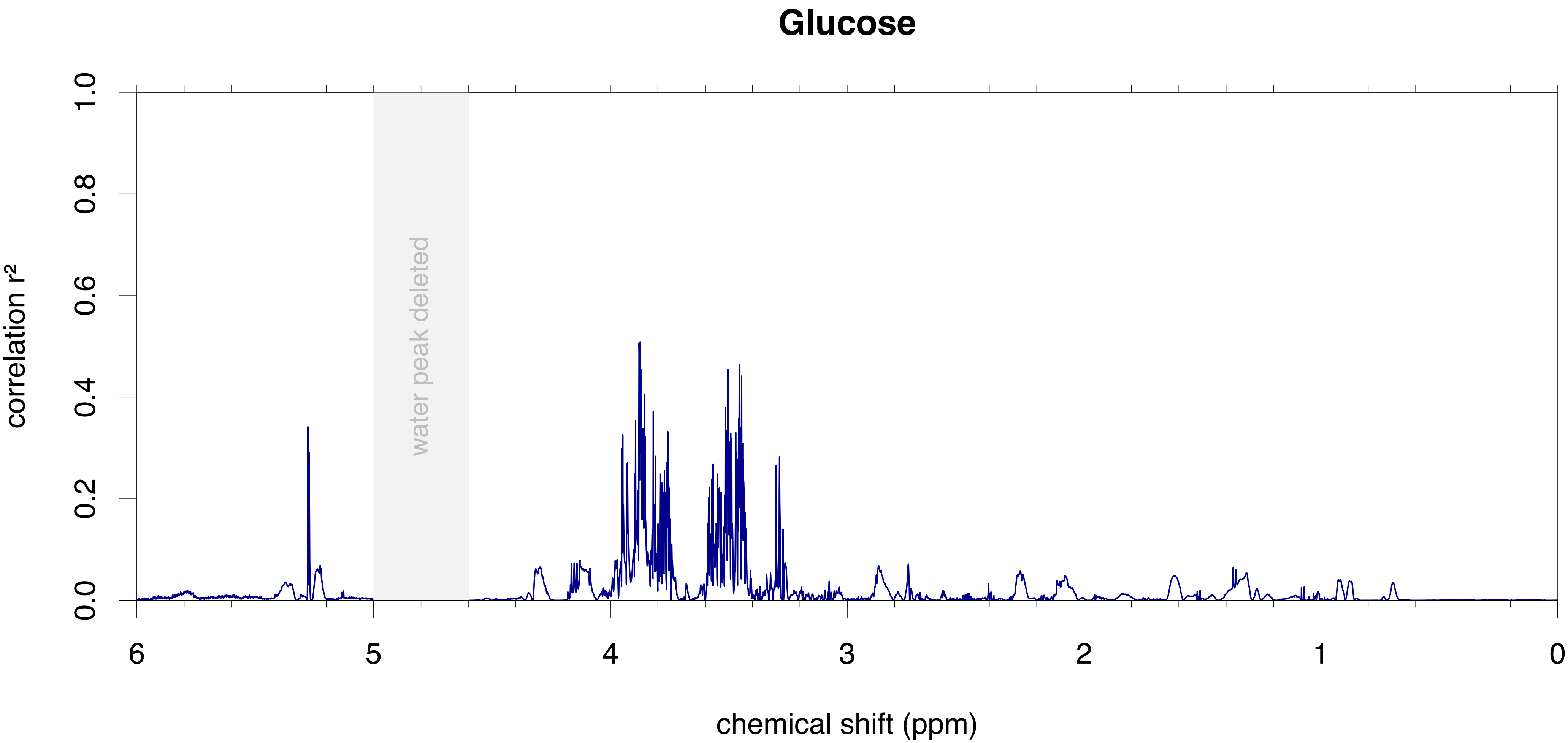

Glucose
correlation r²
1.0
0.8
0.6
0.4
0.2
0.0
water peak deleted
6
5
4
3
2
1
0
chemical shift (ppm)

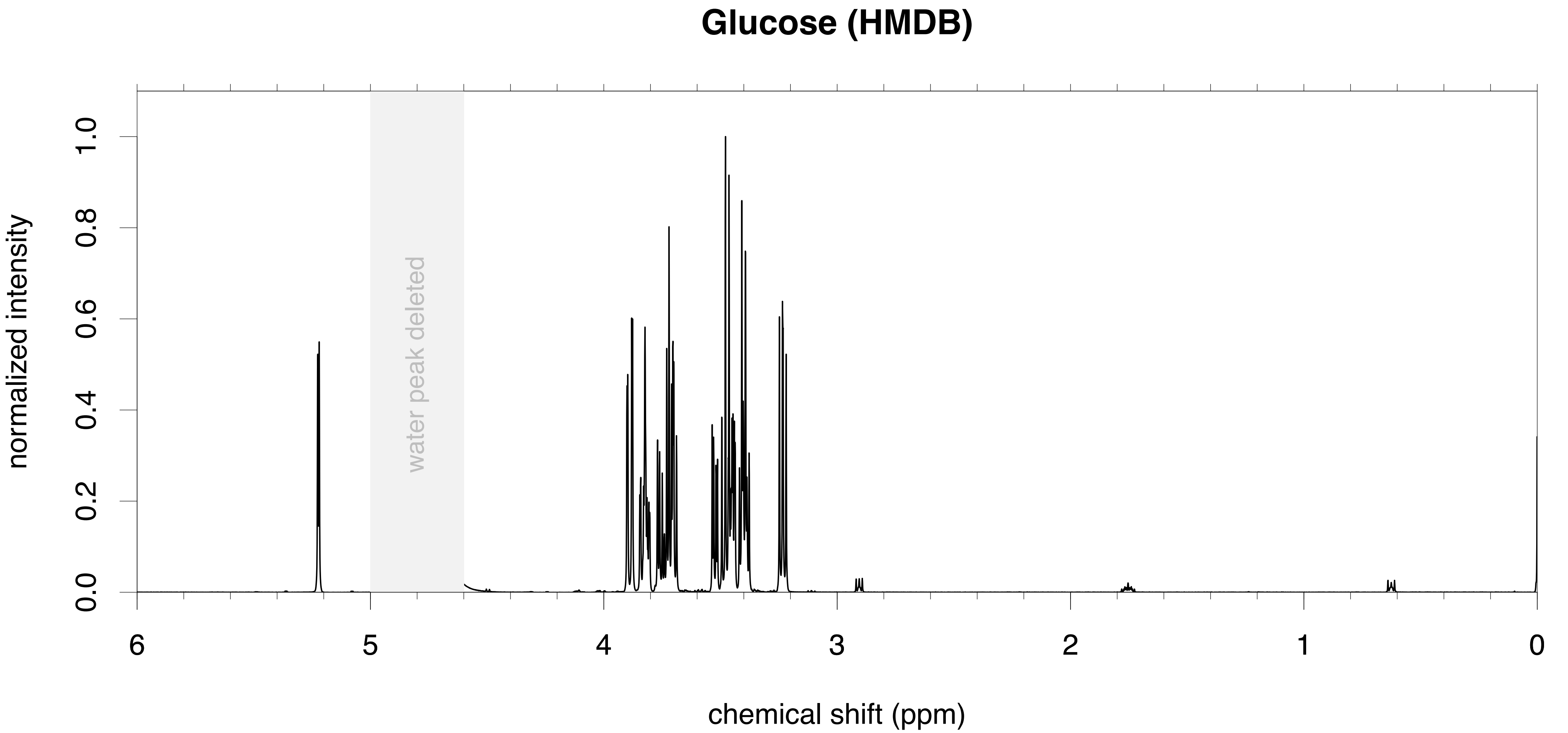

Glucose (HMDB)
normalized intensity
1.0
0.8
0.6
0.4
0.2
0.0
water peak deleted
6
5
4
3
2
1
0
chemical shift (ppm)

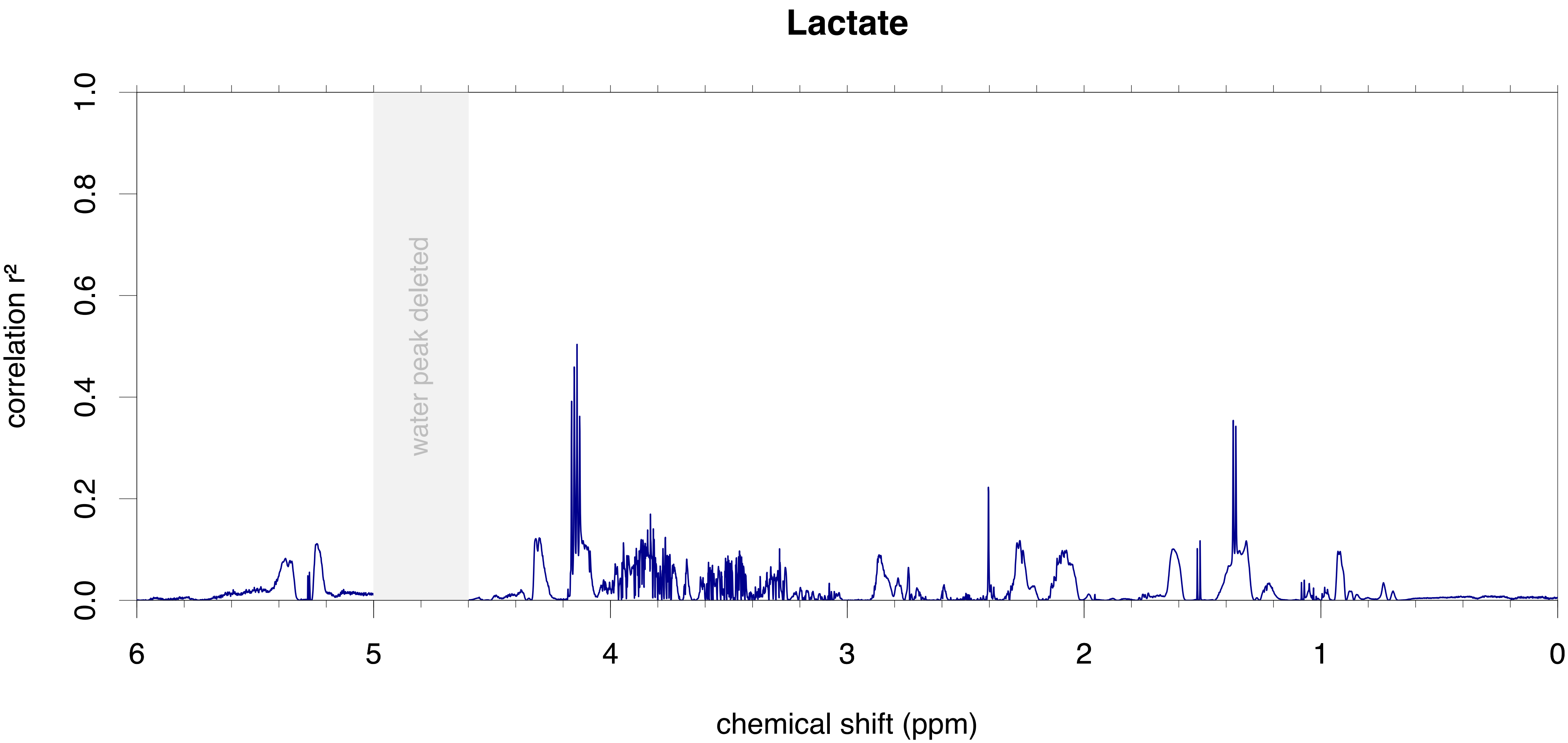

Lactate
correlation r²
water peak deleted
chemical shift (ppm)
0.0
0.2
0.4
0.6
0.8
1.0
6
5
4
3
2
1
0

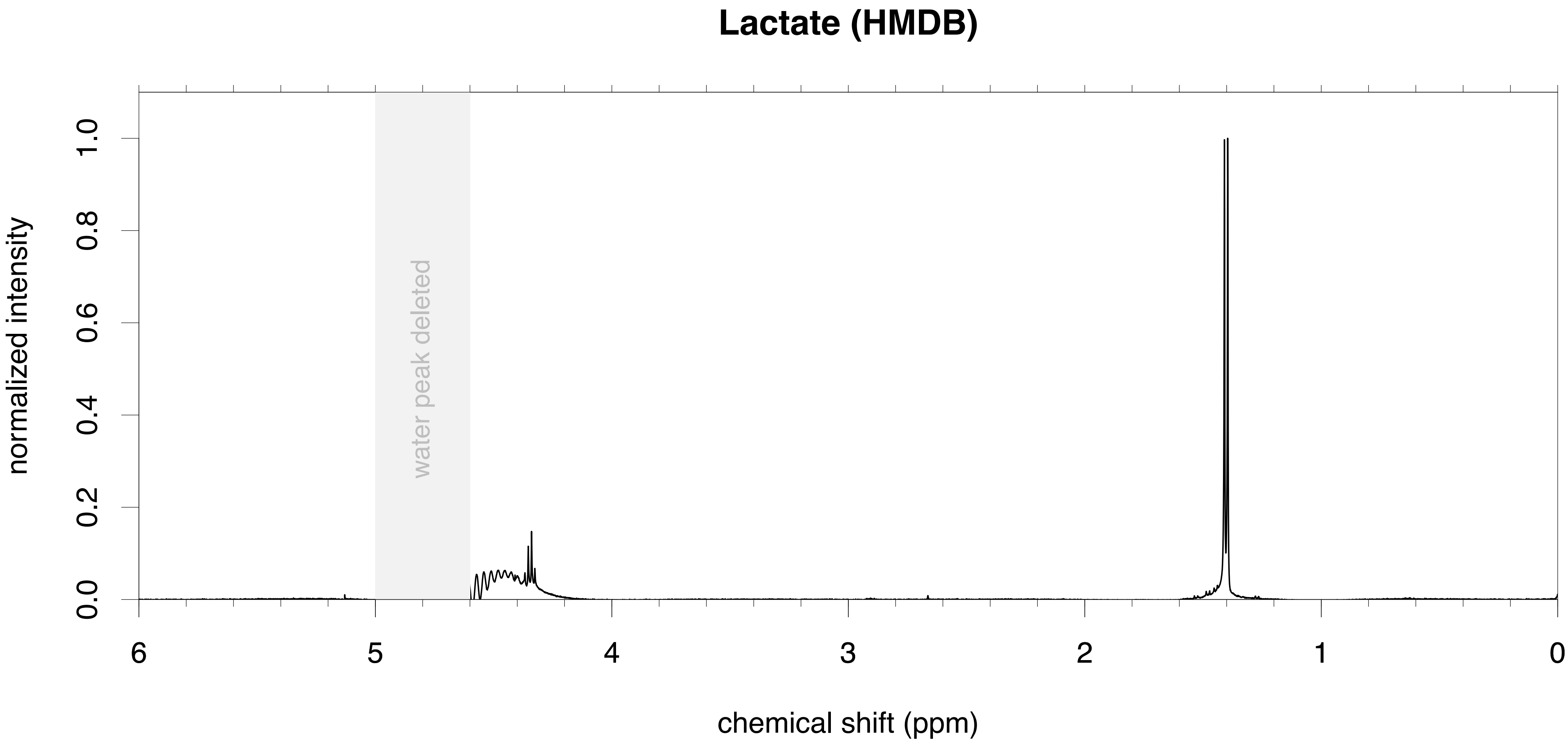
Lactate (HMDB)
normalized intensity
0.0
0.2
0.4
0.6
0.8
1.0
water peak deleted
6
5
4
3
2
1
0
chemical shift (ppm)

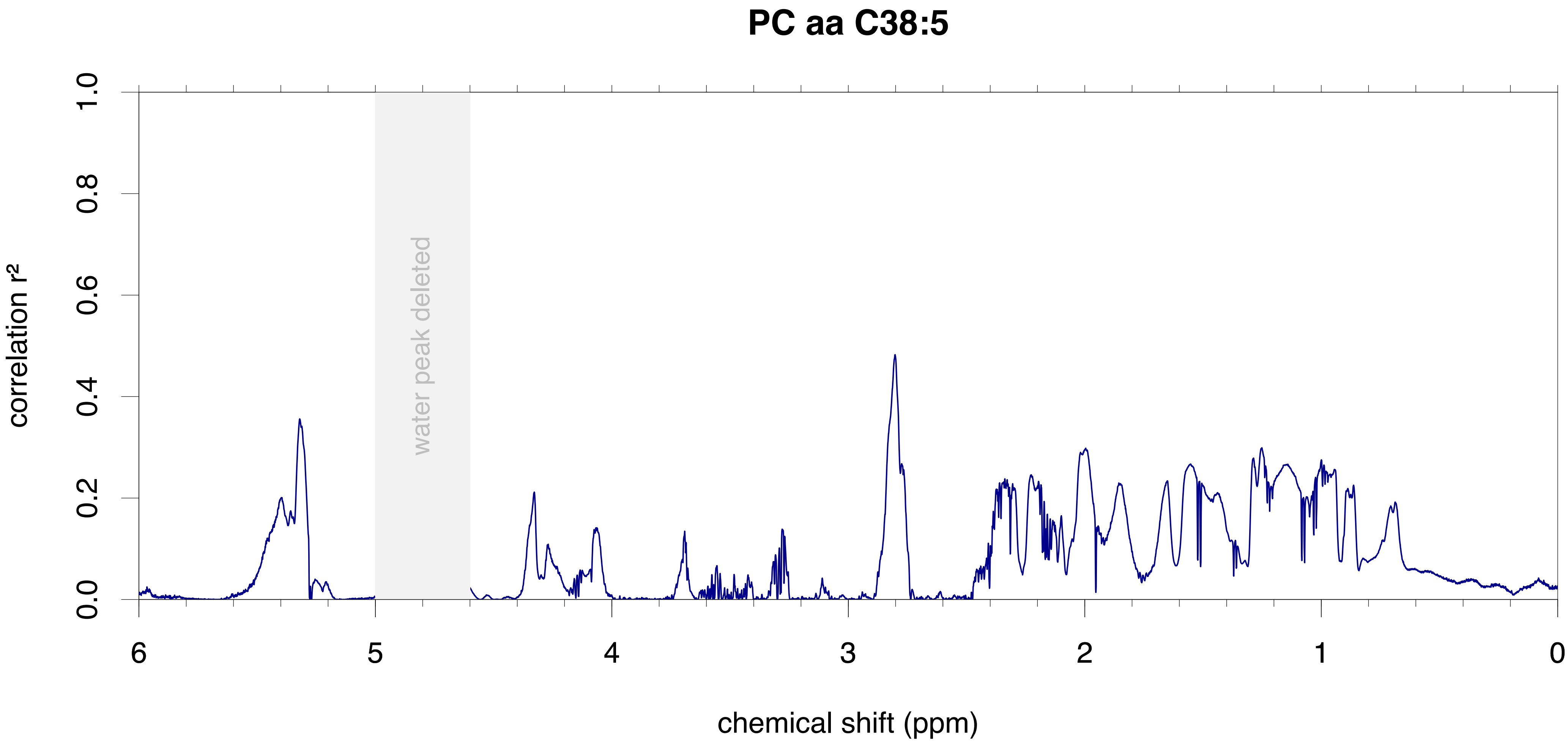

PC aa C38:5
correlation r²
0.0
0.2
0.4
0.6
0.8
1.0
water peak deleted
6
5
4
3
2
1
0
chemical shift (ppm)

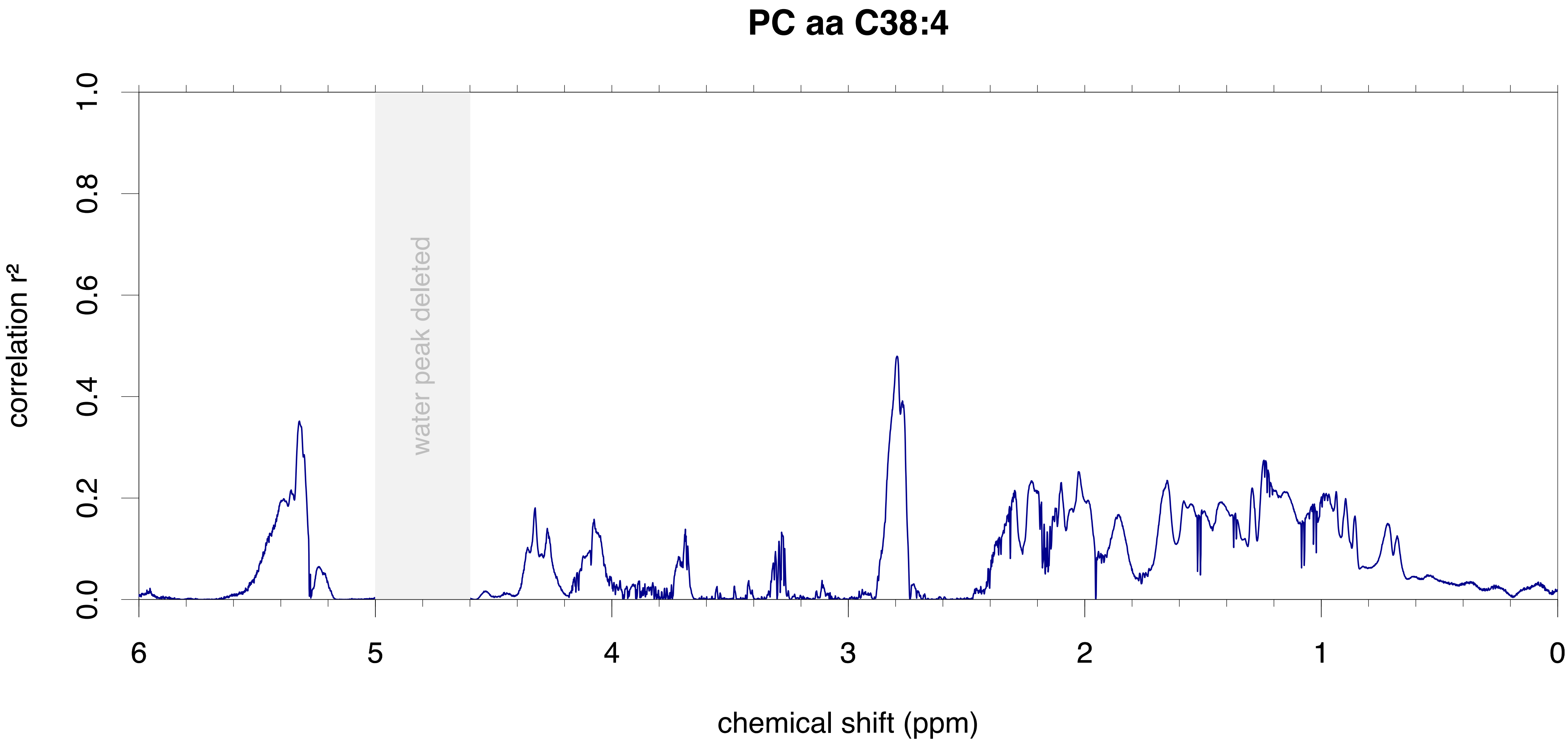

PC aa C38:4
correlation r²
1.0
0.8
0.6
0.4
0.2
0.0
water peak deleted
6
5
4
3
2
1
0
chemical shift (ppm)

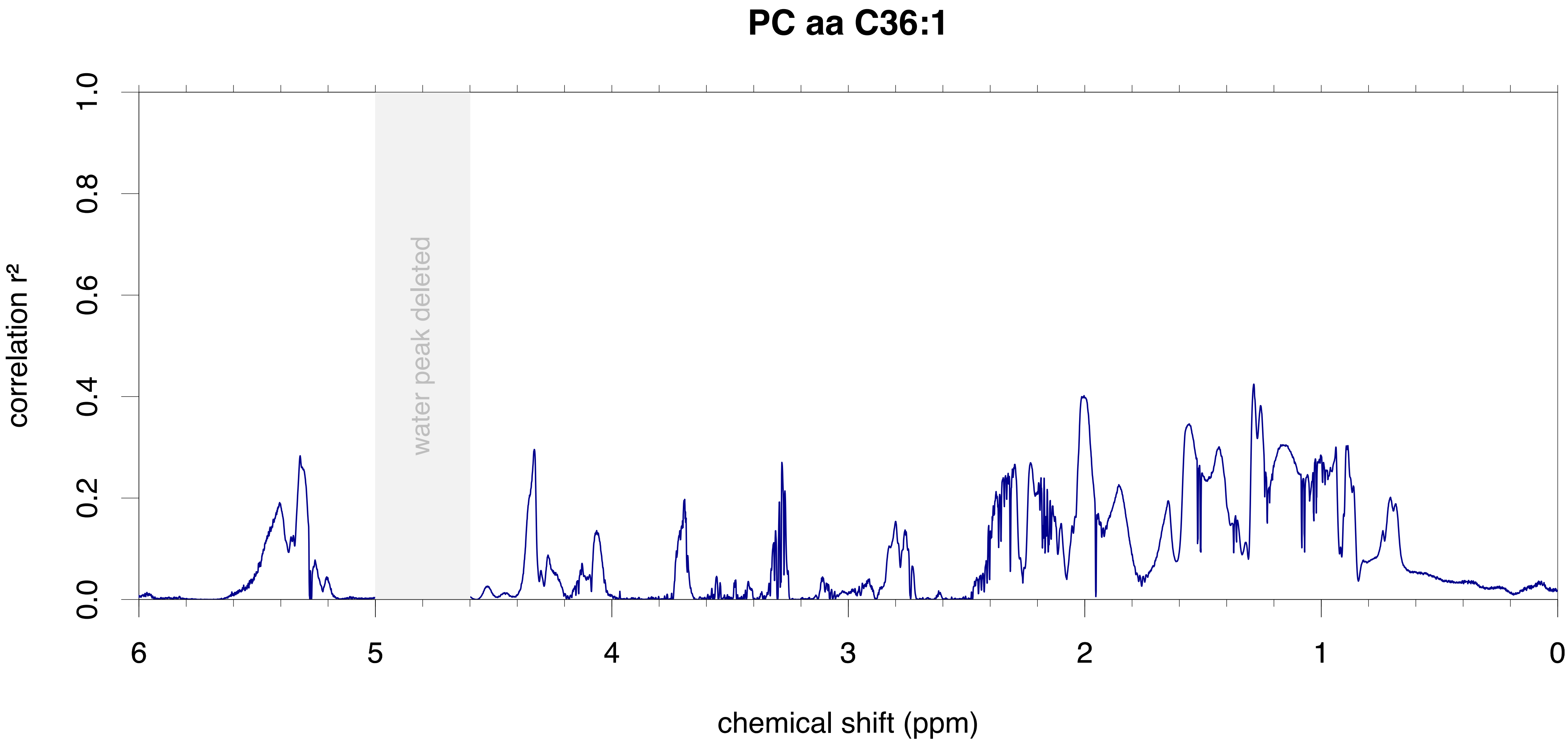

PC aa C36:1
correlation r²
1.0
0.8
0.6
0.4
0.2
0.0
water peak deleted
6
5
4
3
2
1
0
chemical shift (ppm)

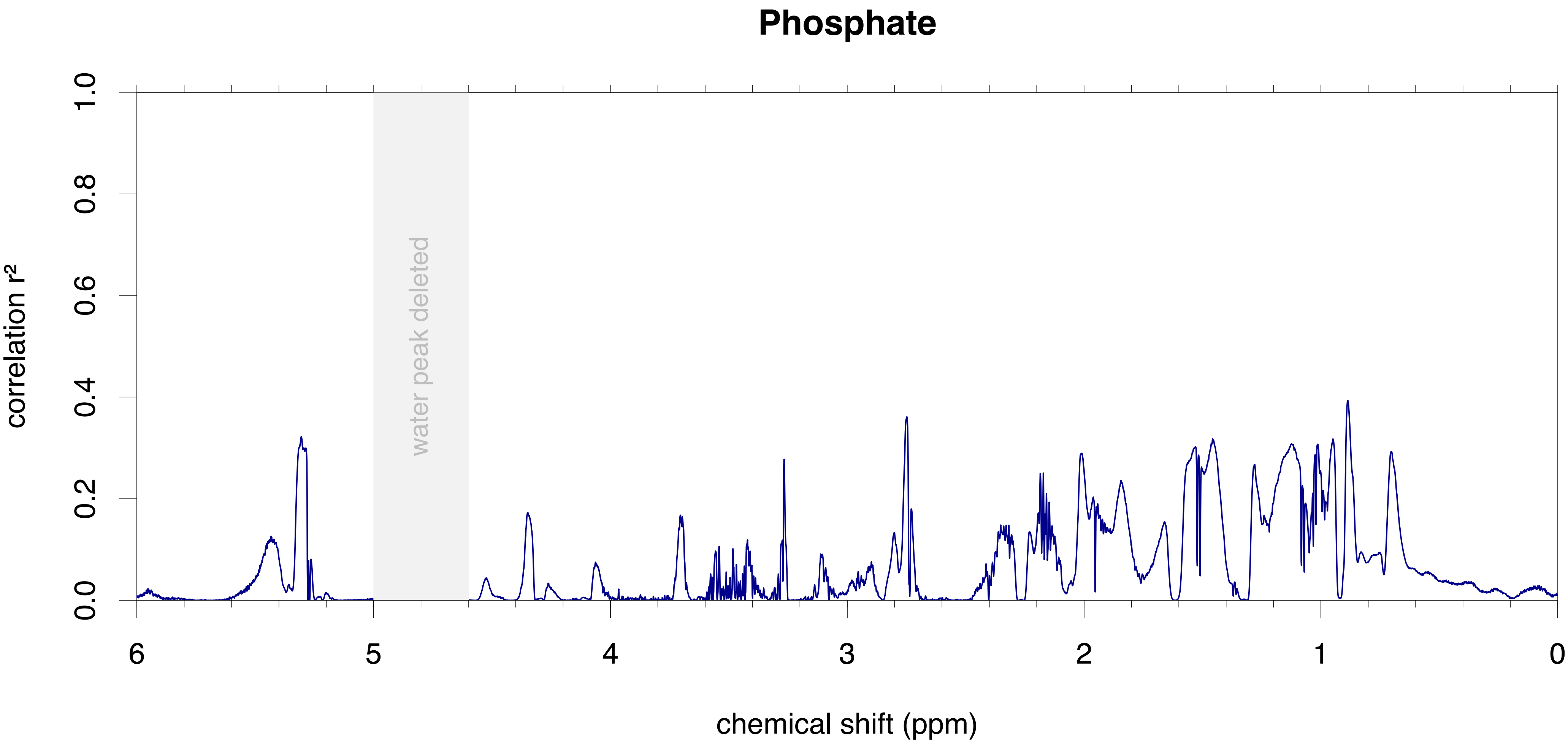

Phosphate
correlation r²
1.0
0.8
0.6
0.4
0.2
0.0
water peak deleted
6
5
4
3
2
1
0
chemical shift (ppm)

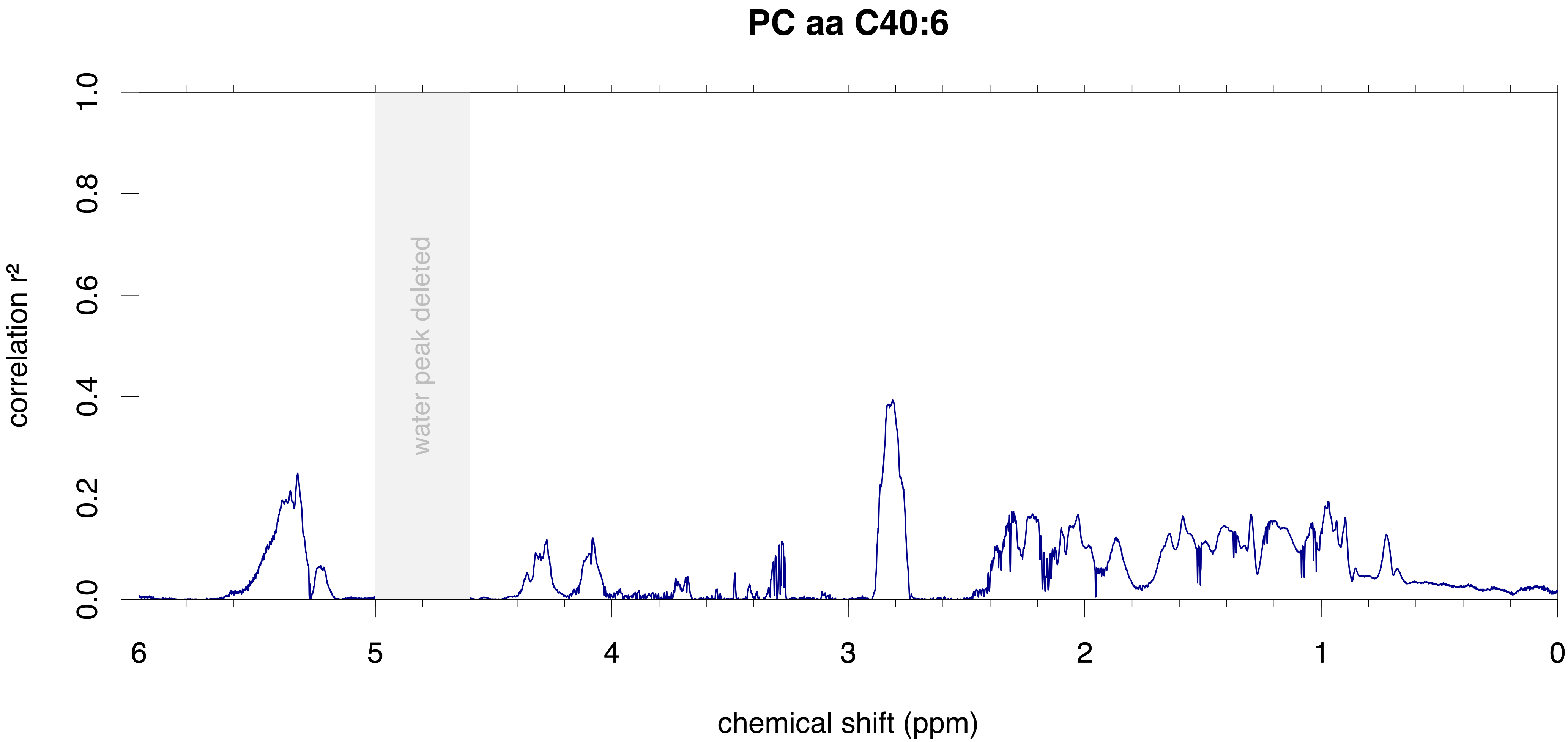

PC aa C40:6
correlation r²
0.0
0.2
0.4
0.6
0.8
1.0
water peak deleted
6
5
4
3
2
1
0
chemical shift (ppm)

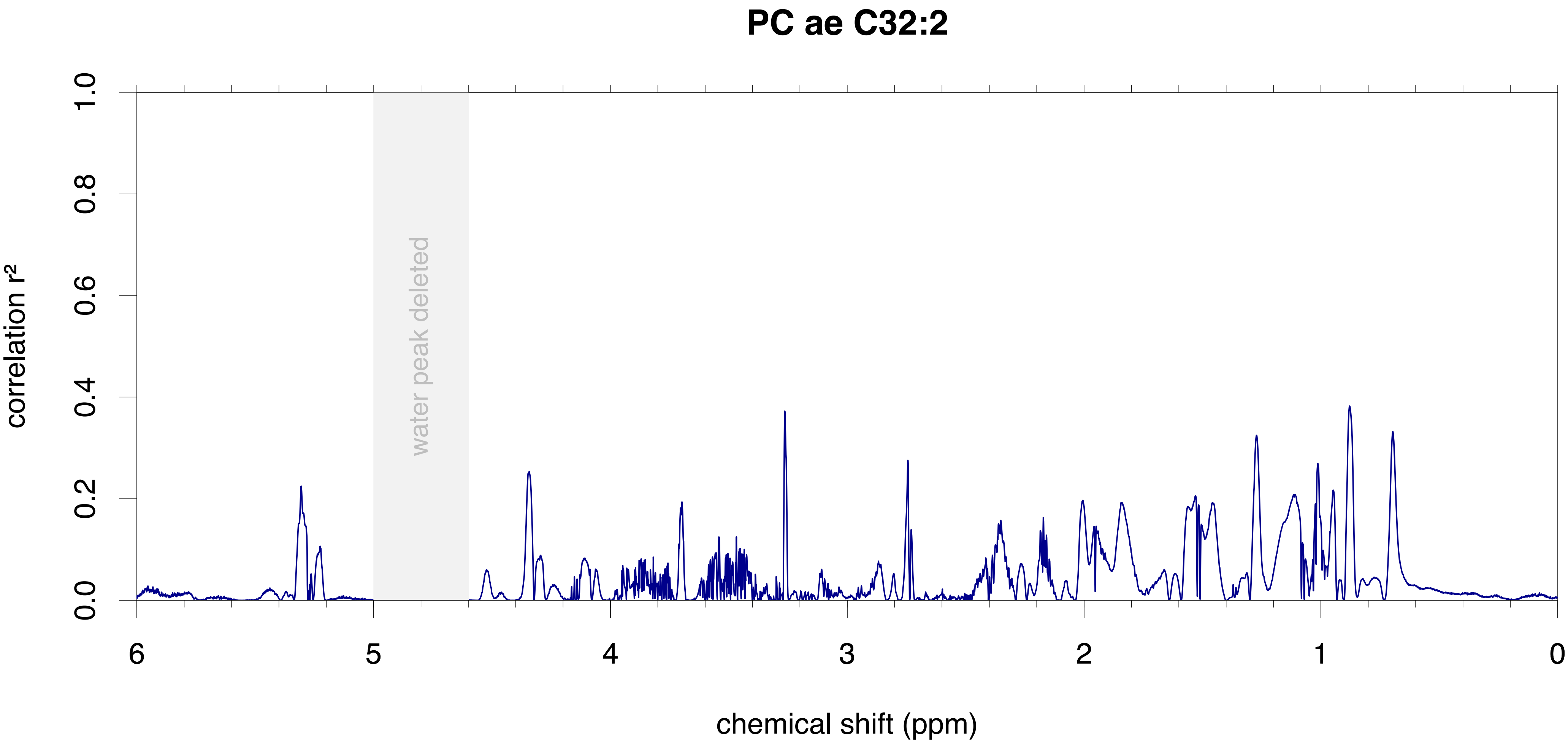

PC ae C32:2
correlation r²
0.0
0.2
0.4
0.6
0.8
1.0
water peak deleted
6
5
4
3
2
1
0
chemical shift (ppm)

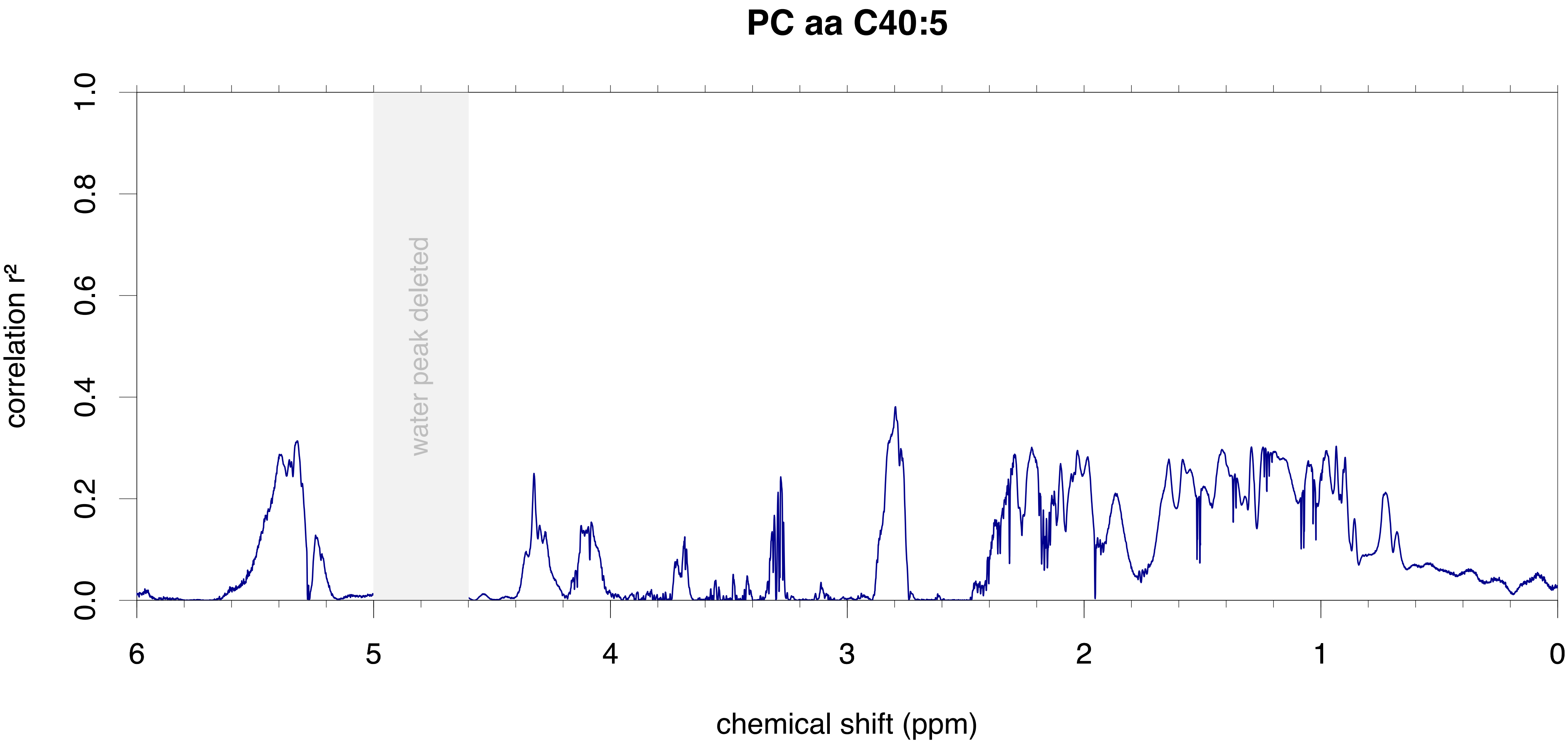
PC aa C40:5
correlation r²
1.0
0.8
0.6
0.4
0.2
0.0
water peak deleted
6
5
4
3
2
1
0
chemical shift (ppm)

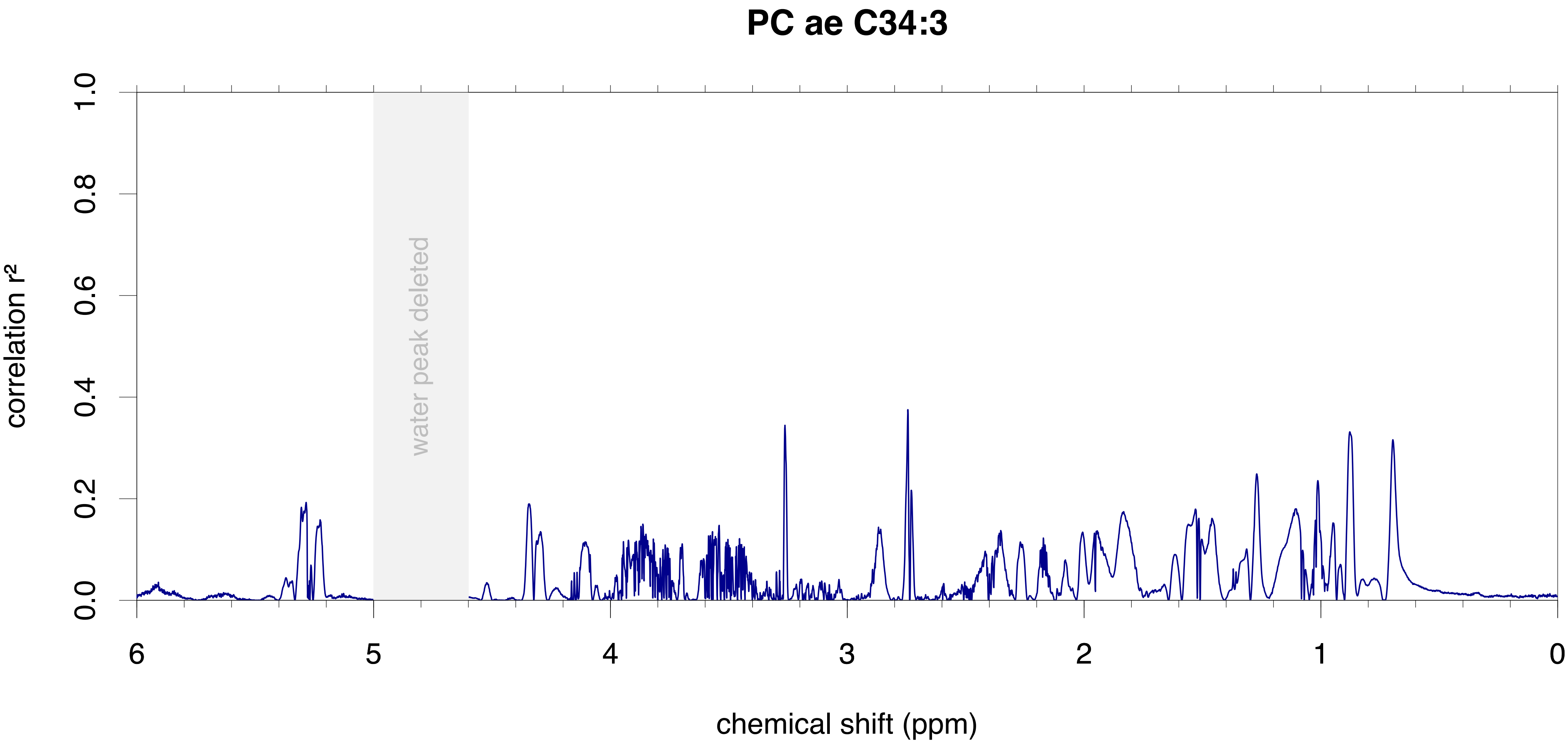

PC ae C34:3
correlation r²
1.0
0.8
0.6
0.4
0.2
0.0
water peak deleted
6
5
4
3
2
1
0
chemical shift (ppm)

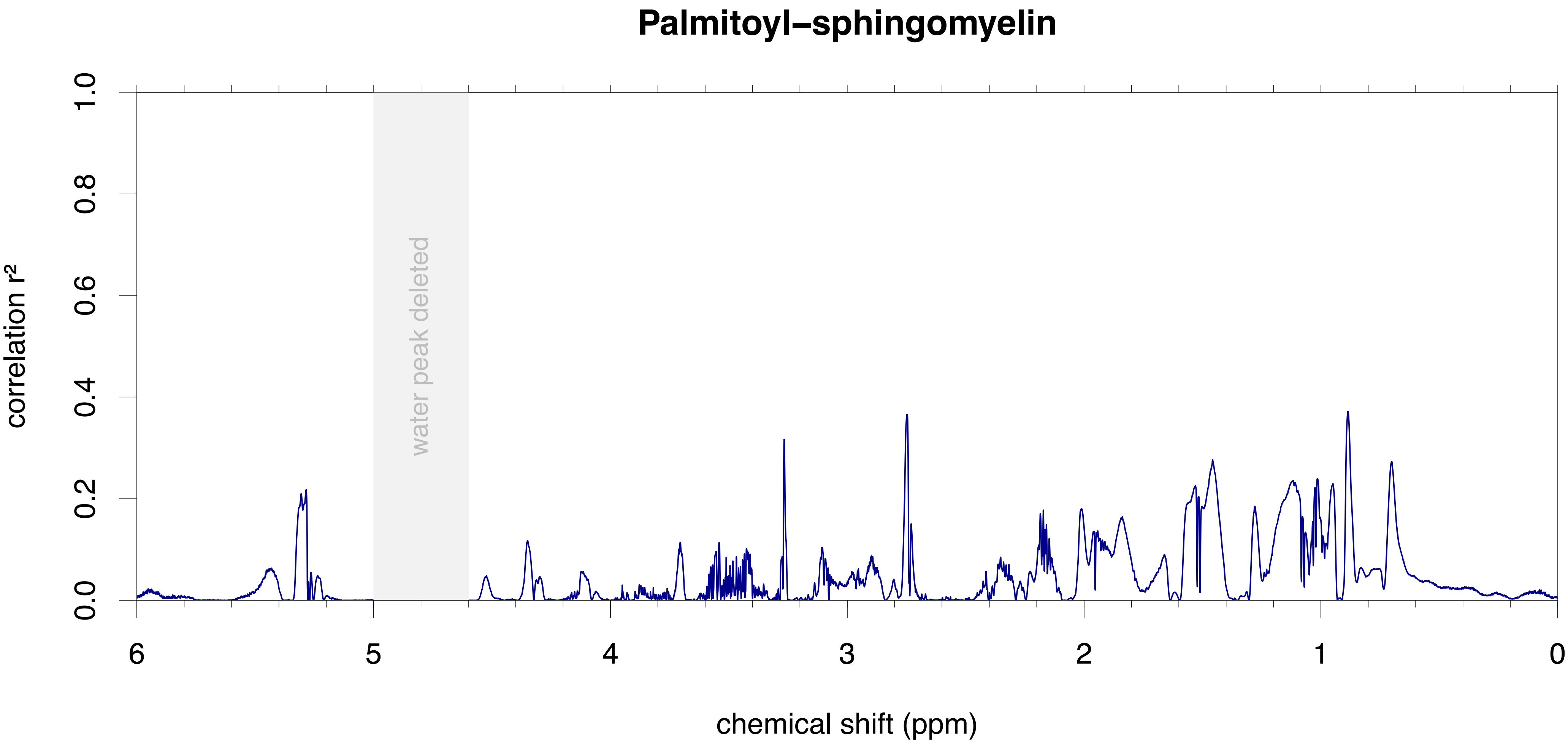

Palmitoyl–sphingomyelin
correlation r²
1.0
0.8
0.6
0.4
0.2
0.0
water peak deleted
6
5
4
3
2
1
0
chemical shift (ppm)

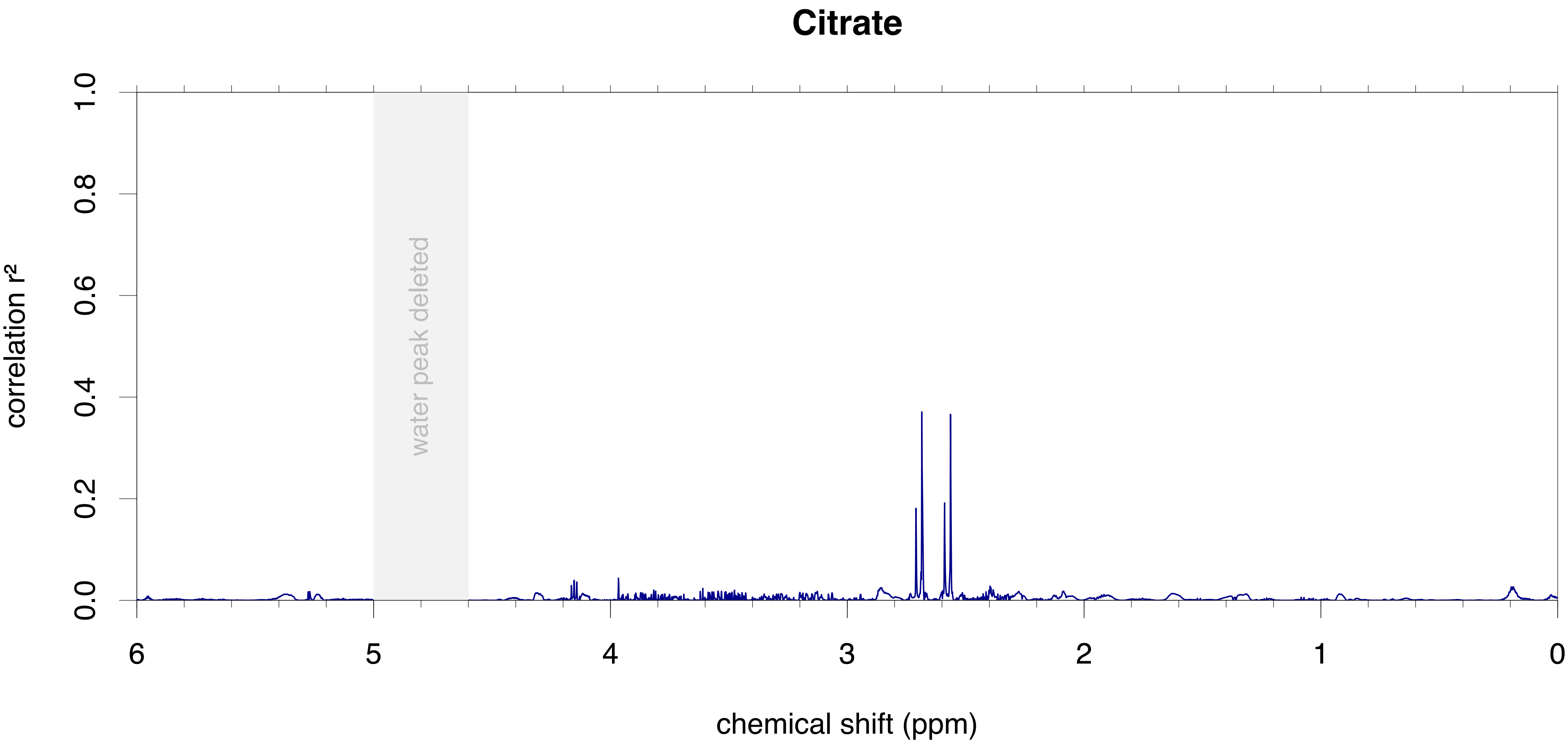

Citrate
correlation r²
1.0
0.8
0.6
0.4
0.2
0.0
water peak deleted
6
5
4
3
2
1
0
chemical shift (ppm)

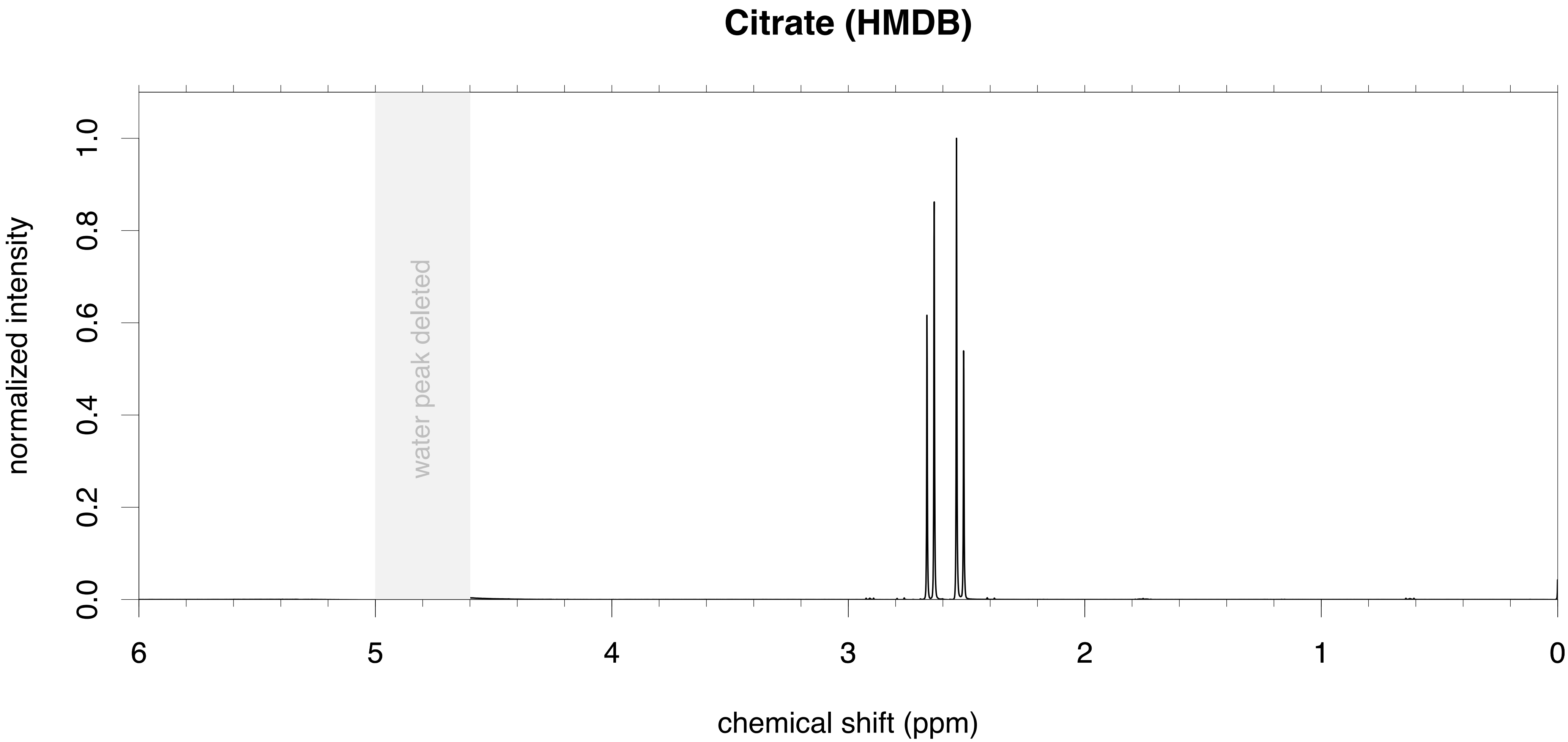

Citrate (HMDB)
normalized intensity
water peak deleted
0.0
0.2
0.4
0.6
0.8
1.0
6
5
4
3
2
1
0
chemical shift (ppm)

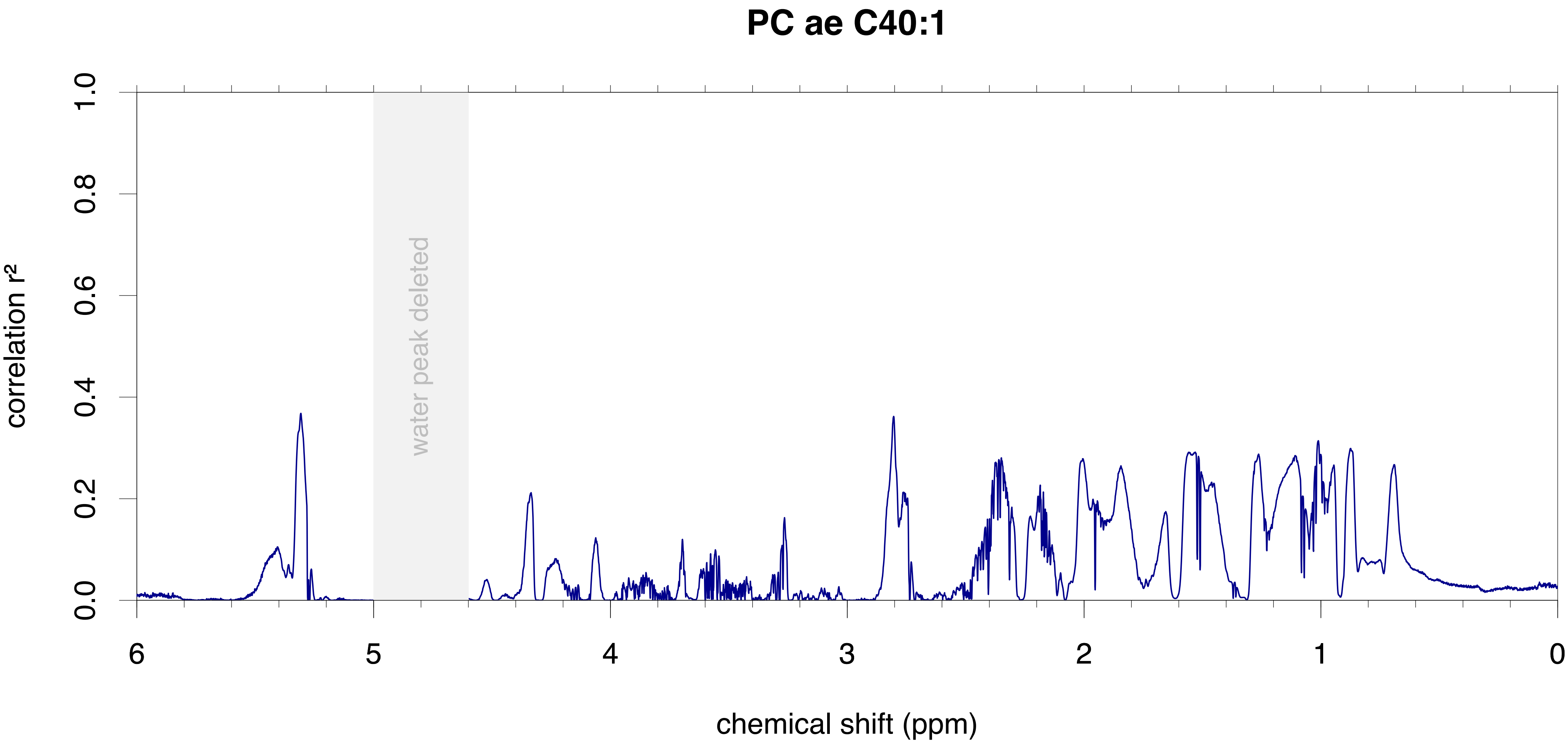
PC ae C40:1
correlation r²
chemical shift (ppm)
water peak deleted
0.0
0.2
0.4
0.6
0.8
1.0
6
5
4
3
2
1
0

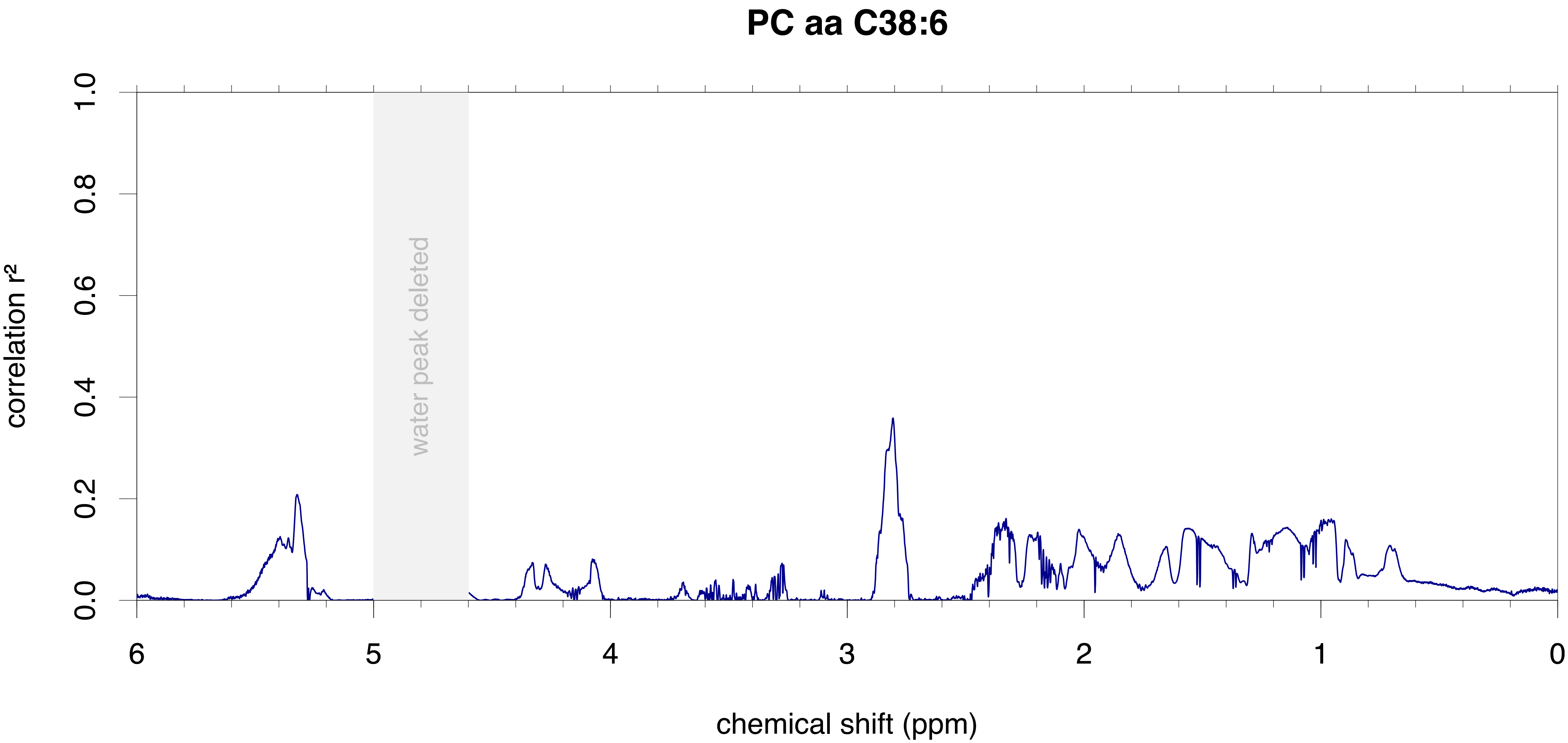

PC aa C38:6
correlation r²
1.0
0.8
0.6
0.4
0.2
0.0
water peak deleted
6
5
4
3
2
1
0
chemical shift (ppm)

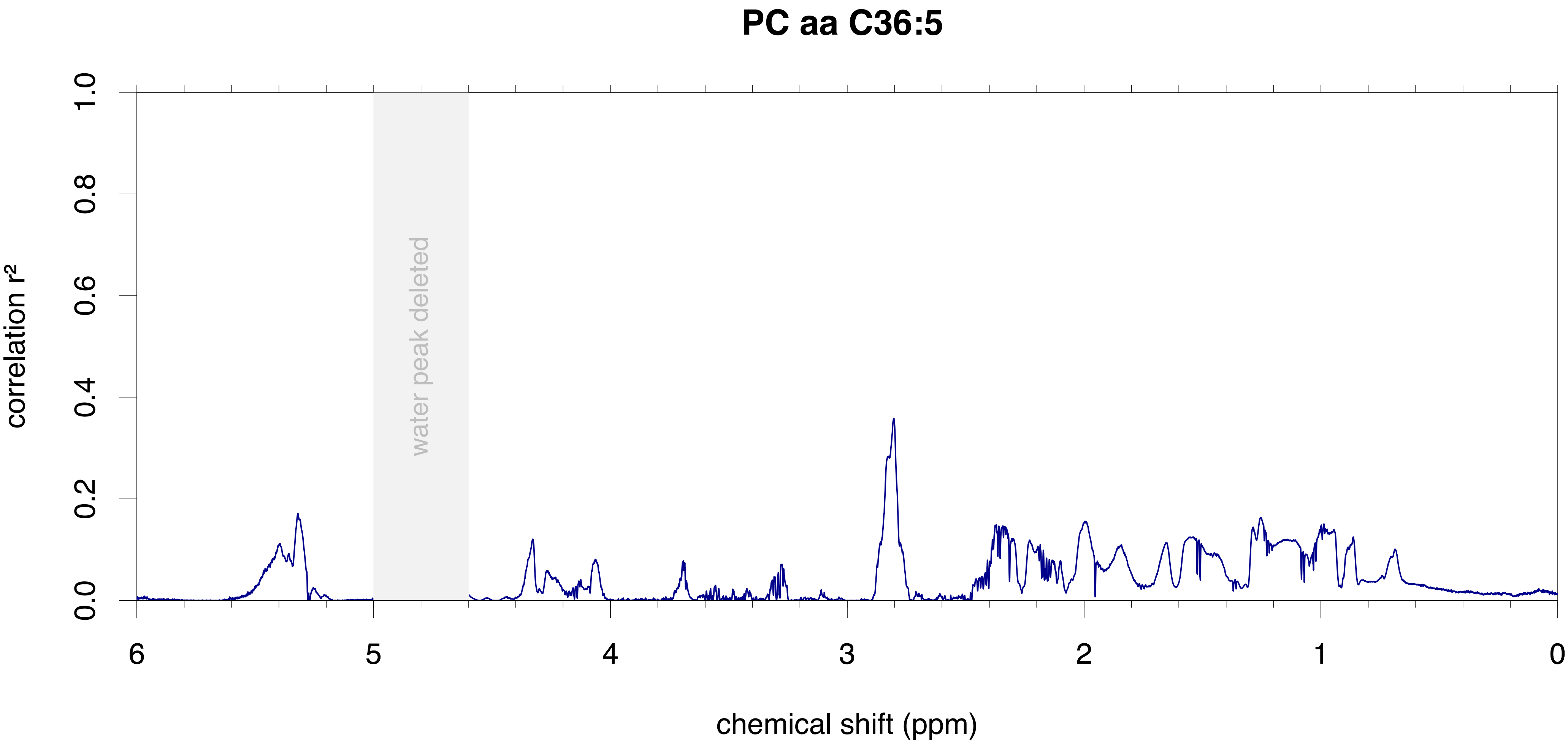

PC aa C36:5
correlation r²
chemical shift (ppm)
water peak deleted
0.0
0.2
0.4
0.6
0.8
1.0
6
5
4
3
2
1
0

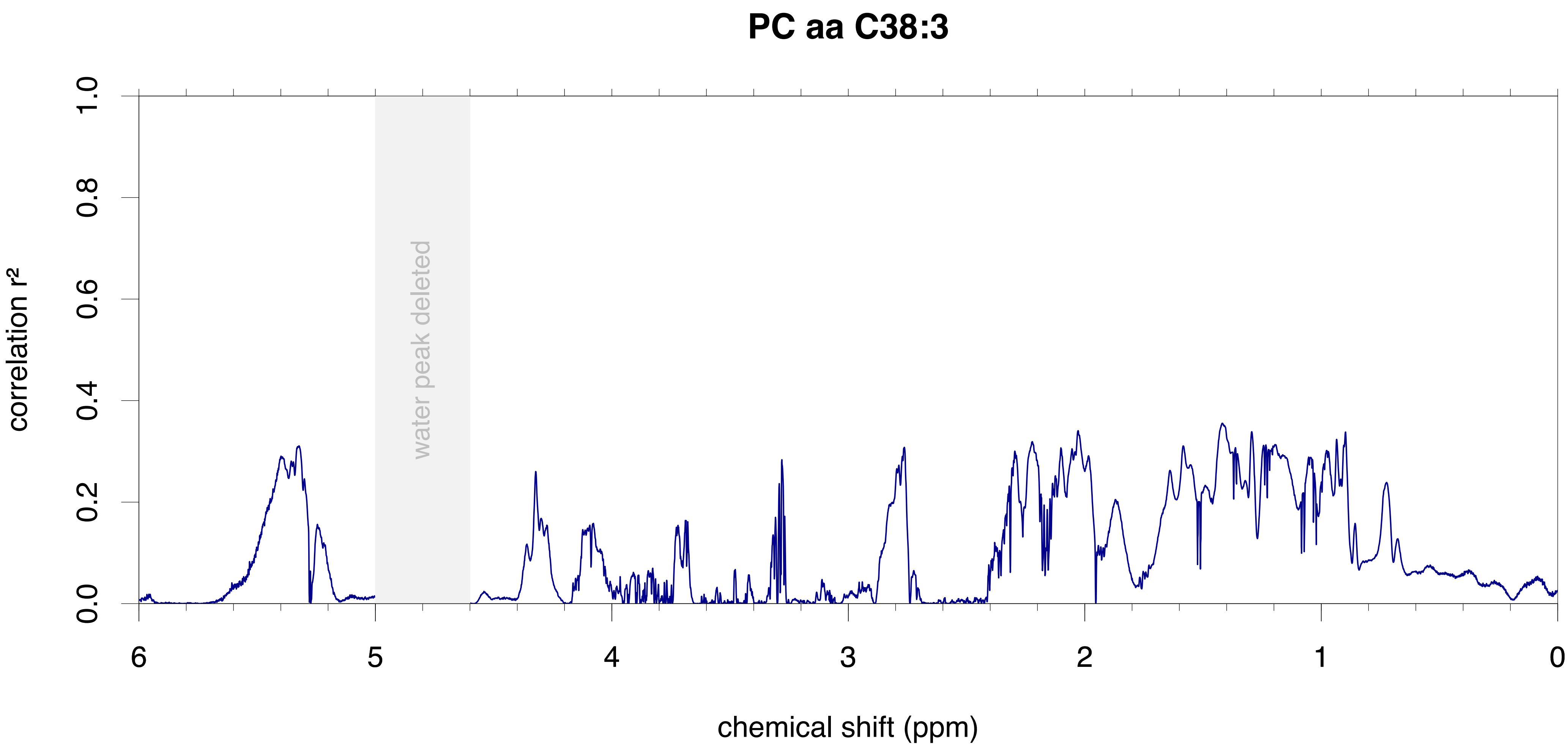
PC aa C38:3
correlation r²
1.0
0.8
0.6
0.4
0.2
0.0
water peak deleted
6
5
4
3
2
1
0
chemical shift (ppm)

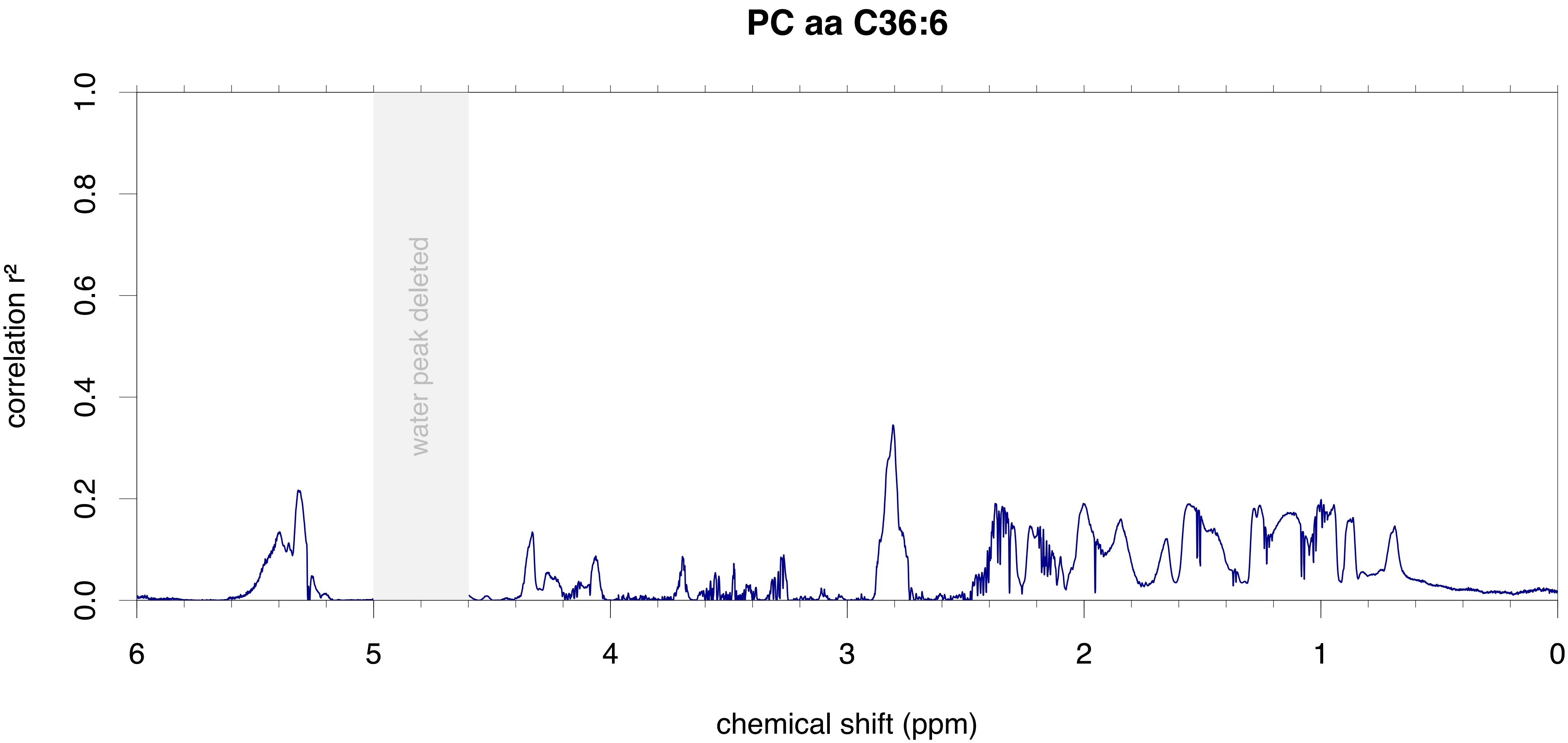
PC aa C36:6
correlation r²
1.0
0.8
0.6
0.4
0.2
0.0
water peak deleted
6
5
4
3
2
1
0
chemical shift (ppm)

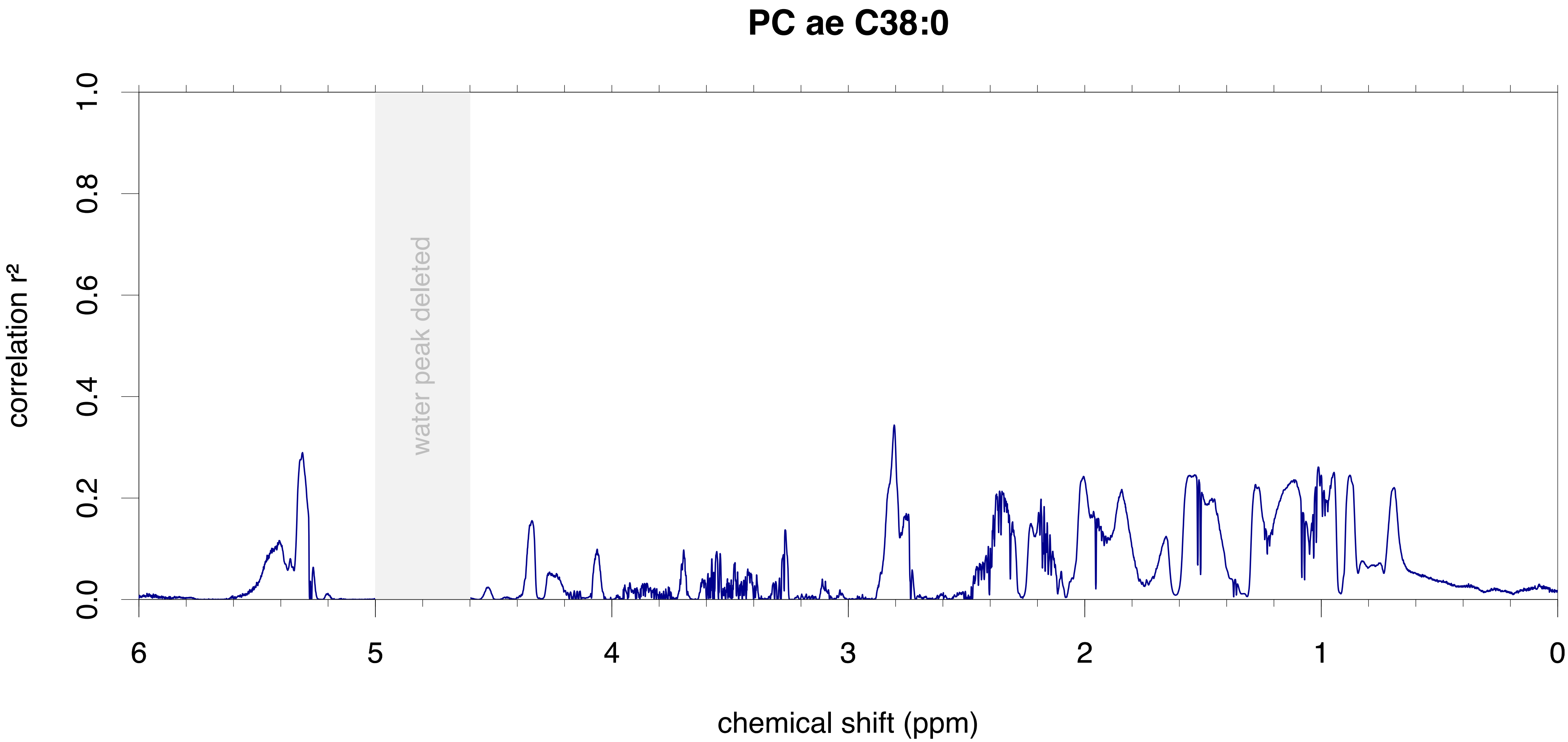

PC ae C38:0
correlation r²
1.0
0.8
0.6
0.4
0.2
0.0
water peak deleted
6
5
4
3
2
1
0
chemical shift (ppm)

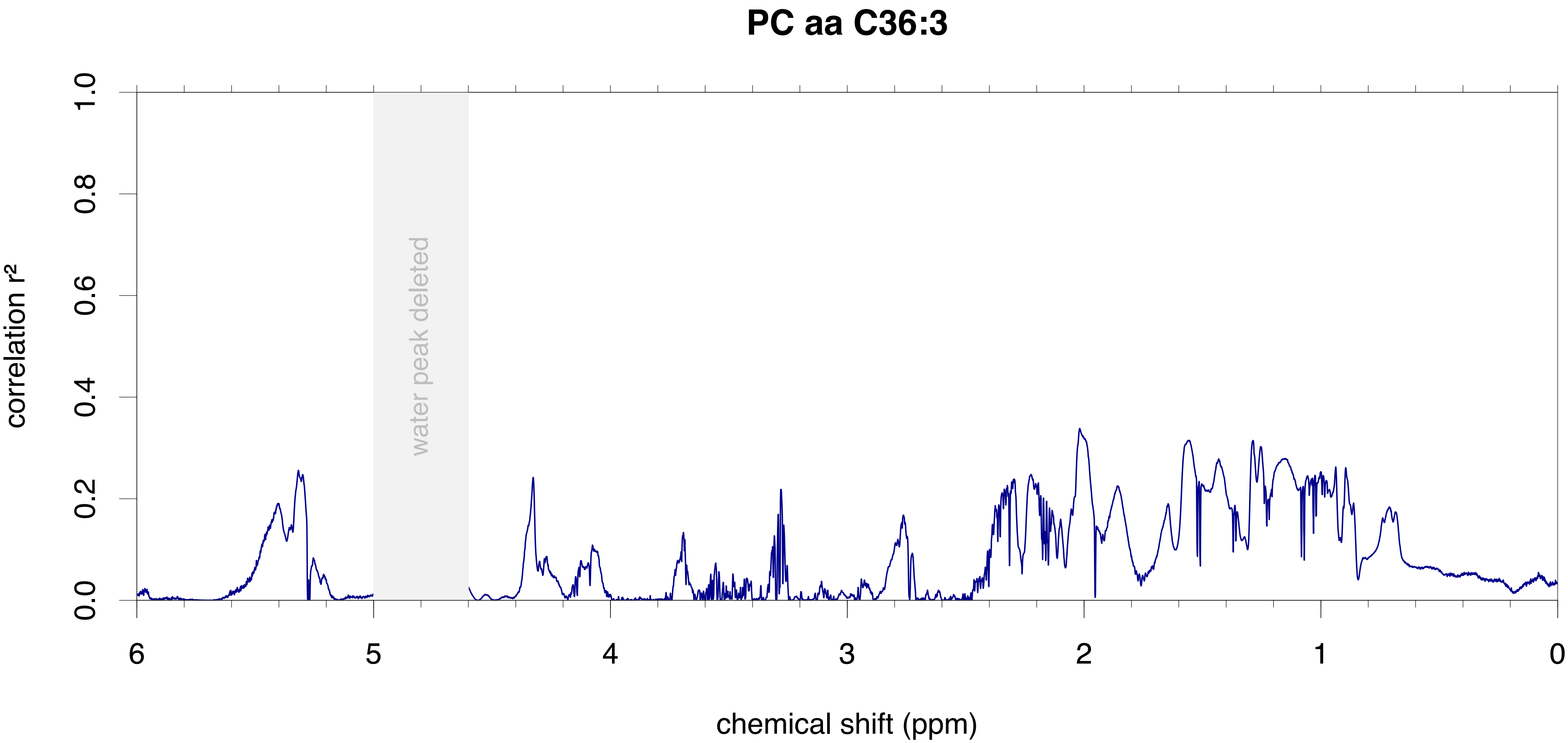

PC aa C36:3
correlation r²
0.0
0.2
0.4
0.6
0.8
1.0
water peak deleted
6
5
4
3
2
1
0
chemical shift (ppm)

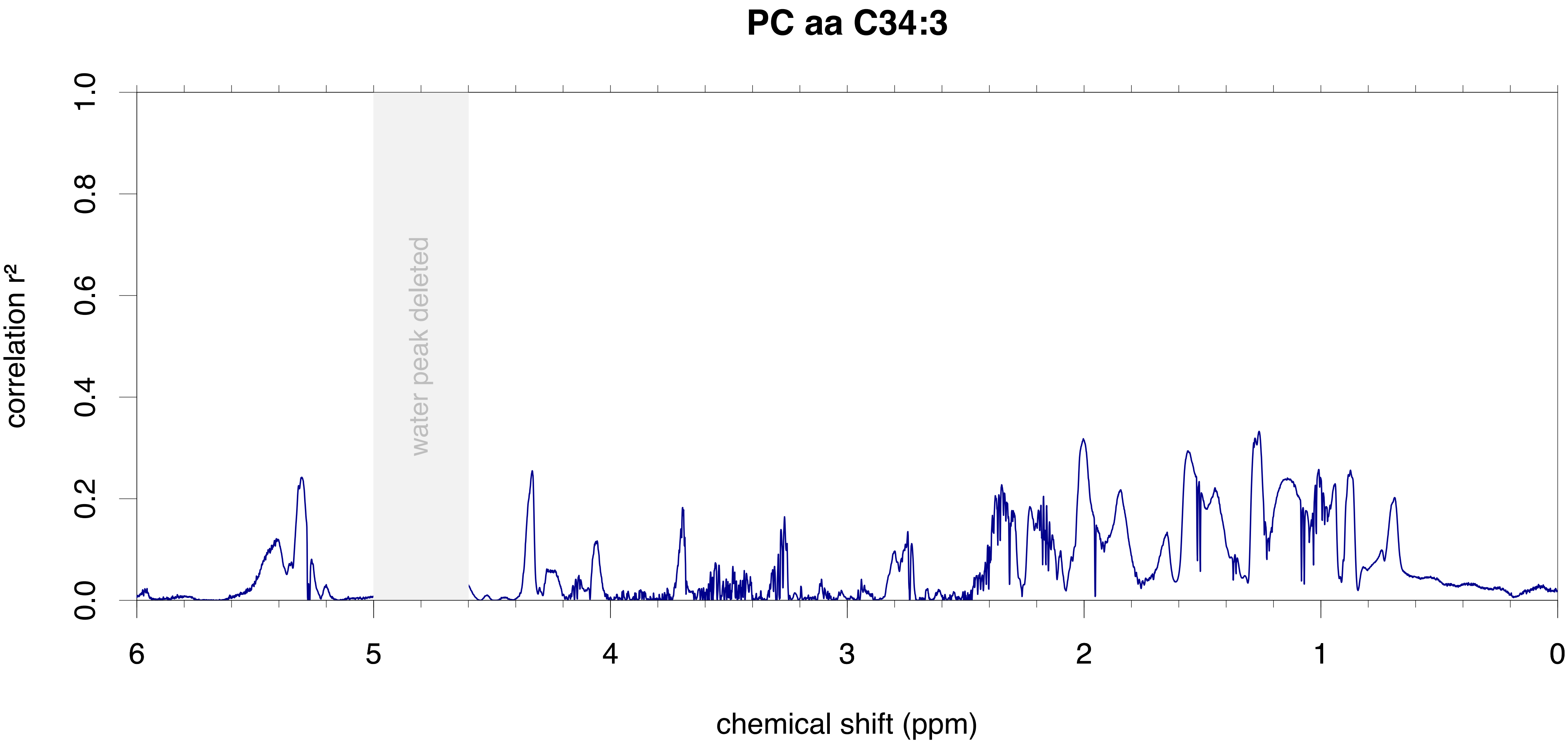

PC aa C34:3
correlation r²
1.0
0.8
0.6
0.4
0.2
0.0
water peak deleted
6
5
4
3
2
1
0
chemical shift (ppm)

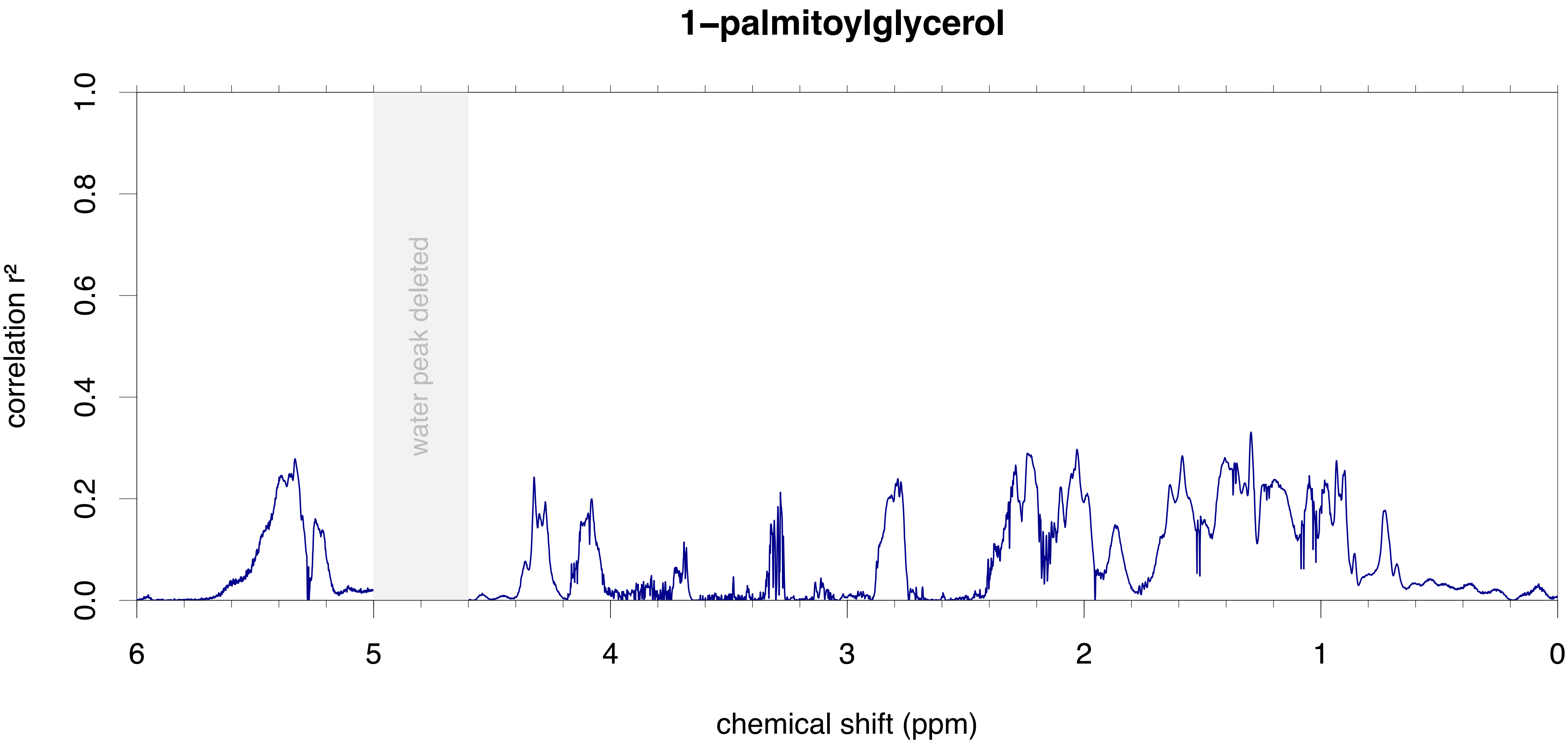

1−palmitoylglycerol
correlation r²
0.0
0.2
0.4
0.6
0.8
1.0
water peak deleted
6
5
4
3
2
1
0
chemical shift (ppm)

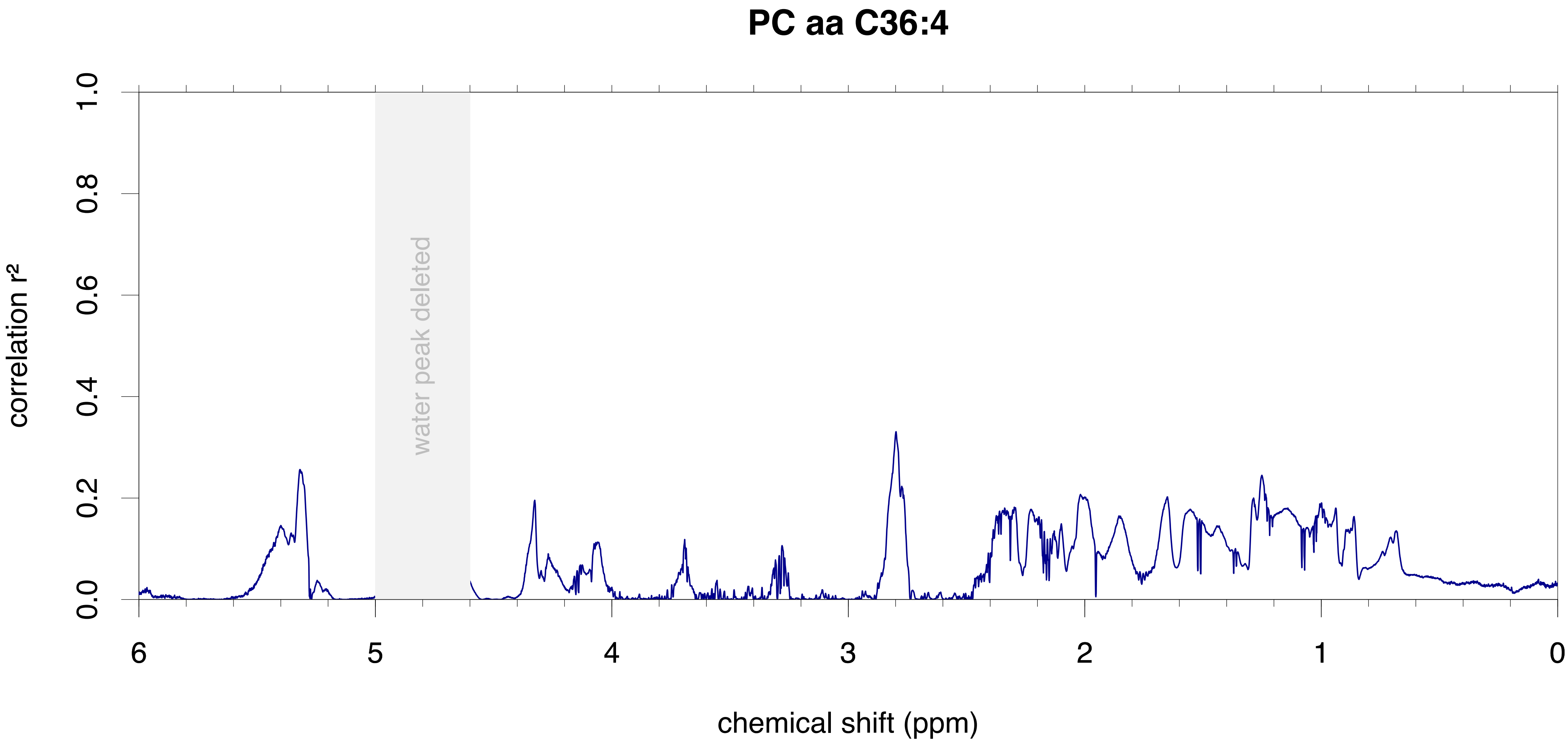
PC aa C36:4
correlation r²
1.0
0.8
0.6
0.4
0.2
0.0
water peak deleted
6
5
4
3
2
1
0
chemical shift (ppm)

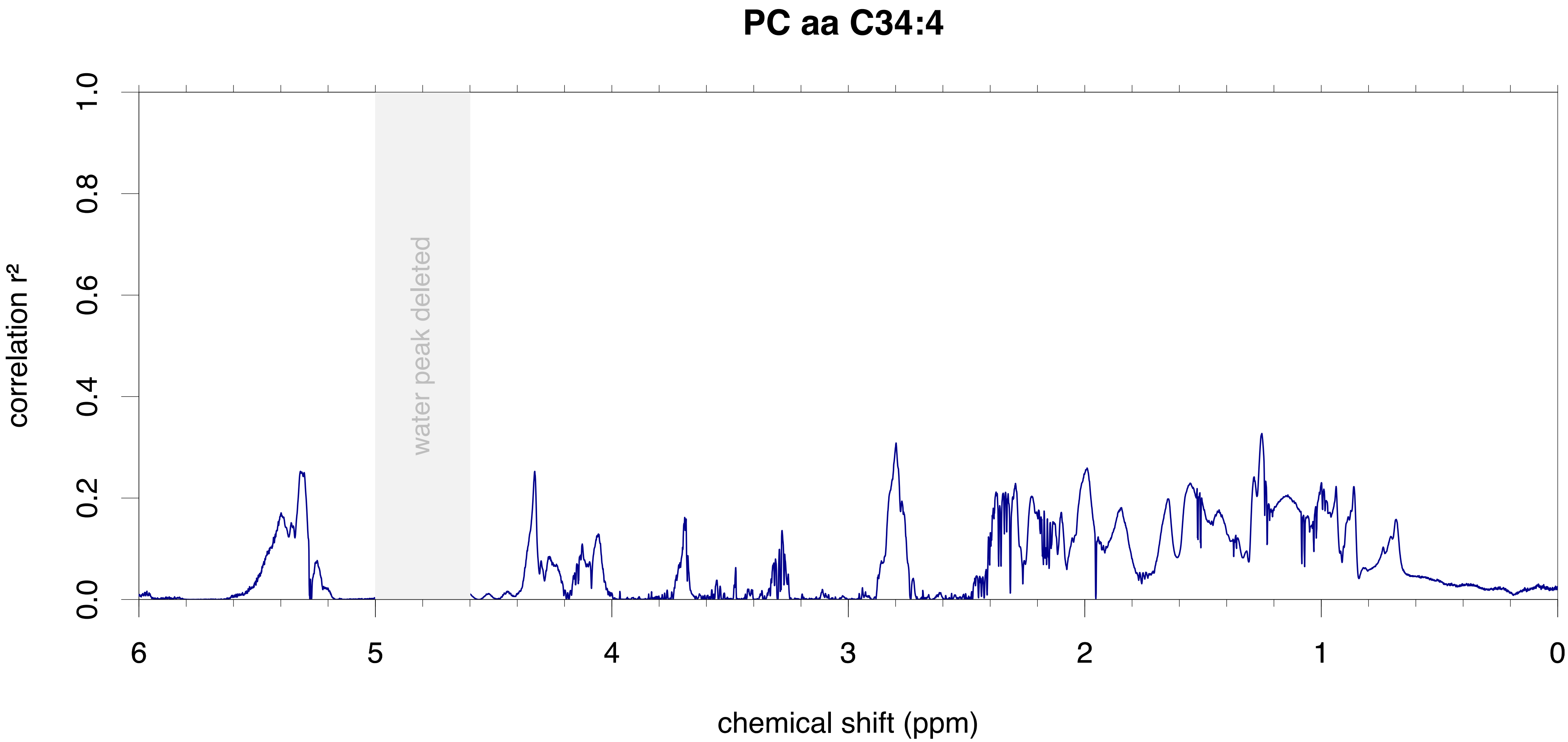
PC aa C34:4
correlation r²
1.0
0.8
0.6
0.4
0.2
0.0
water peak deleted
6
5
4
3
2
1
0
chemical shift (ppm)

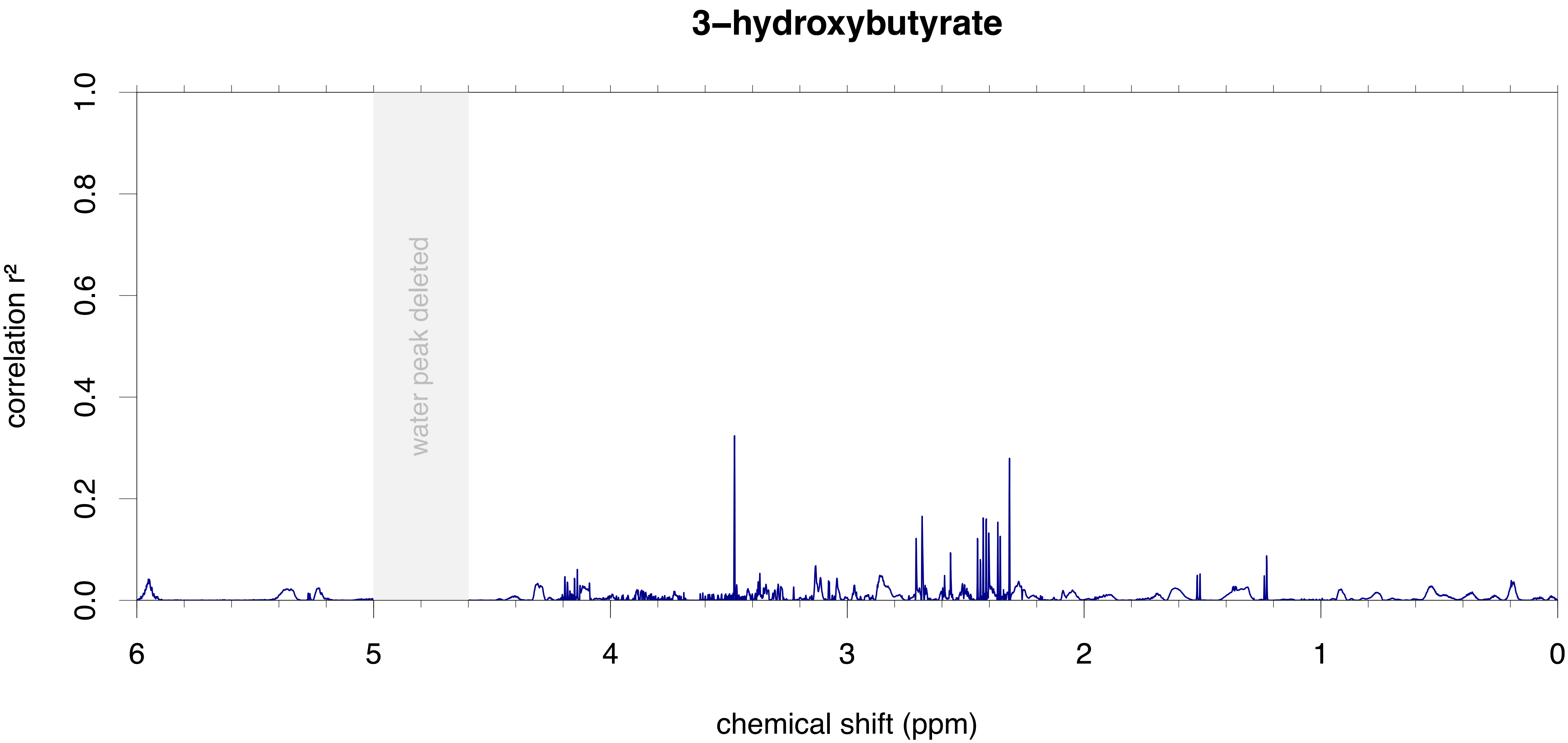

3−hydroxybutyrate
correlation r²
1.0
0.8
0.6
0.4
0.2
0.0
water peak deleted
6
5
4
3
2
1
0
chemical shift (ppm)

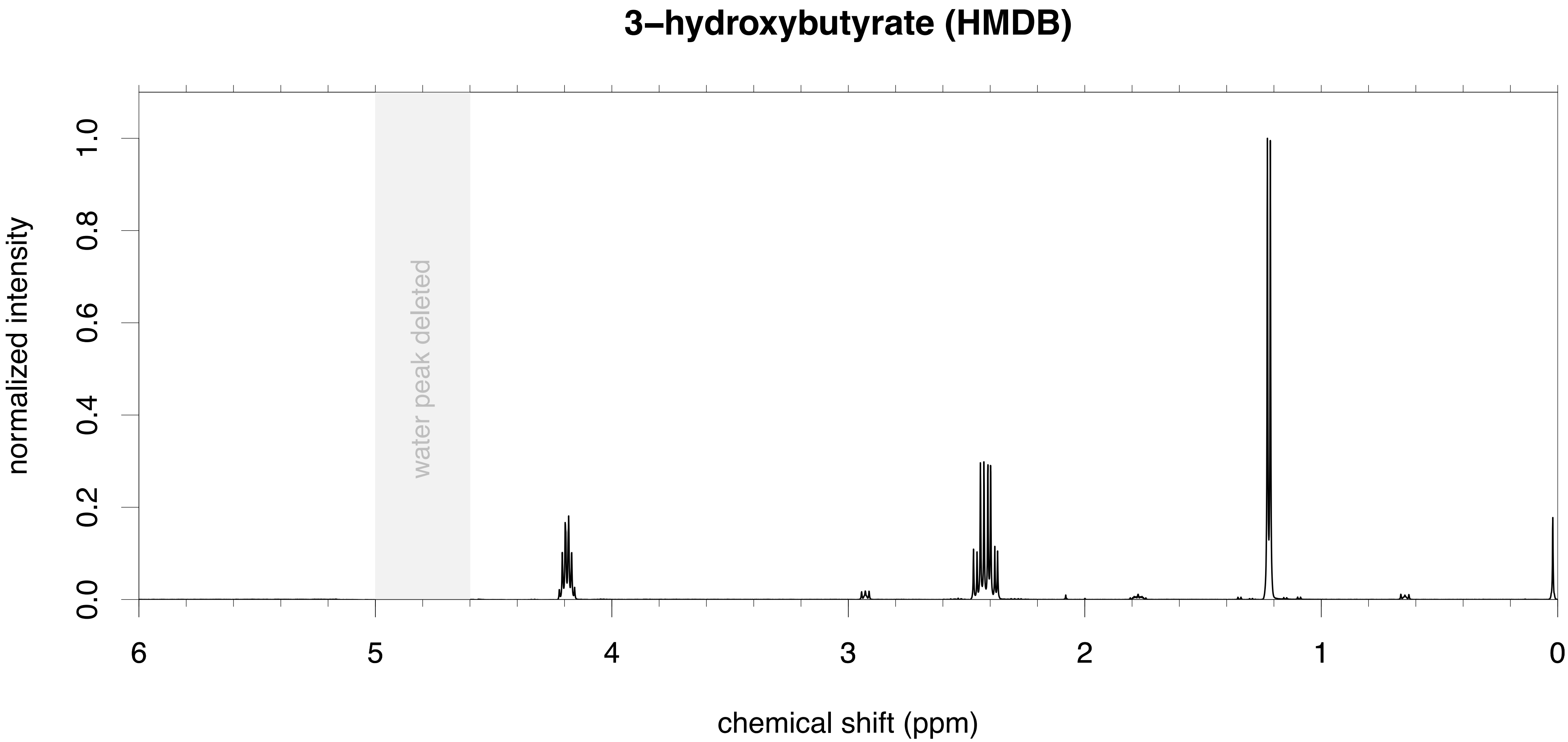

3–hydroxybutyrate (HMDB)
normalized intensity
0.0
0.2
0.4
0.6
0.8
1.0
water peak deleted
6
5
4
3
2
1
0
chemical shift (ppm)

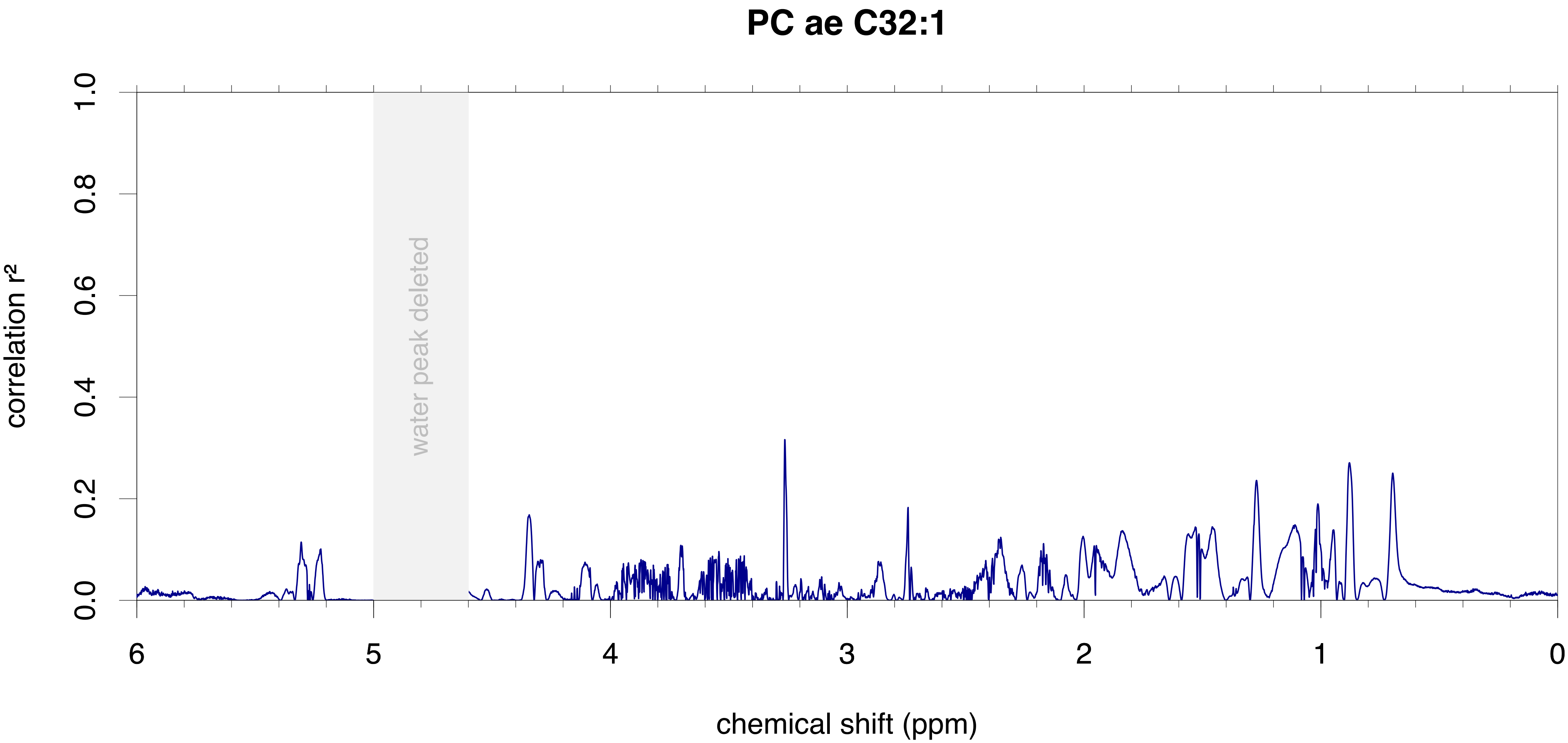

PC ae C32:1
water peak deleted
correlation r²
0.0
0.2
0.4
0.6
0.8
1.0
6
5
4
3
2
1
0
chemical shift (ppm)

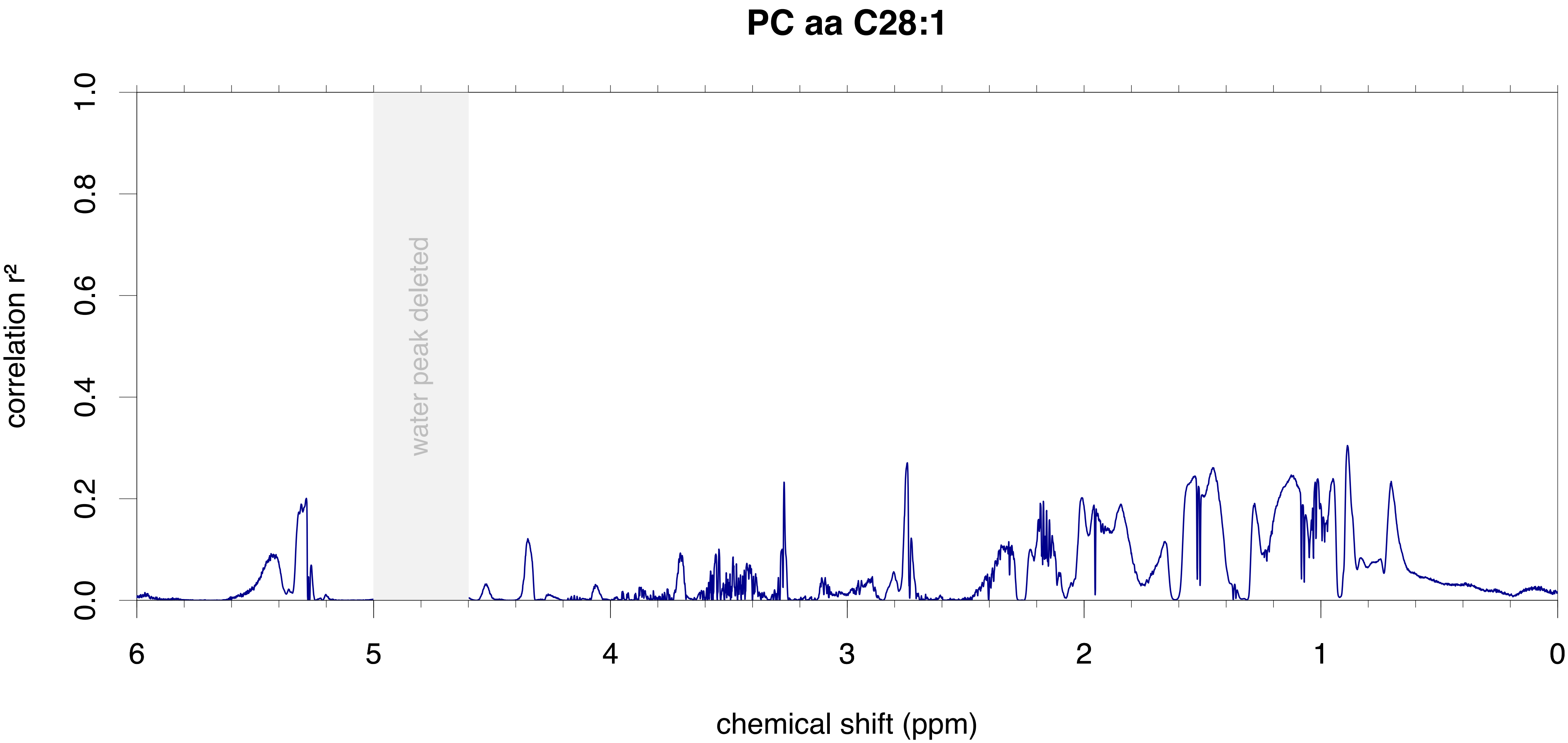

PC aa C28:1
correlation r²
0.0
0.2
0.4
0.6
0.8
1.0
water peak deleted
6
5
4
3
2
1
0
chemical shift (ppm)

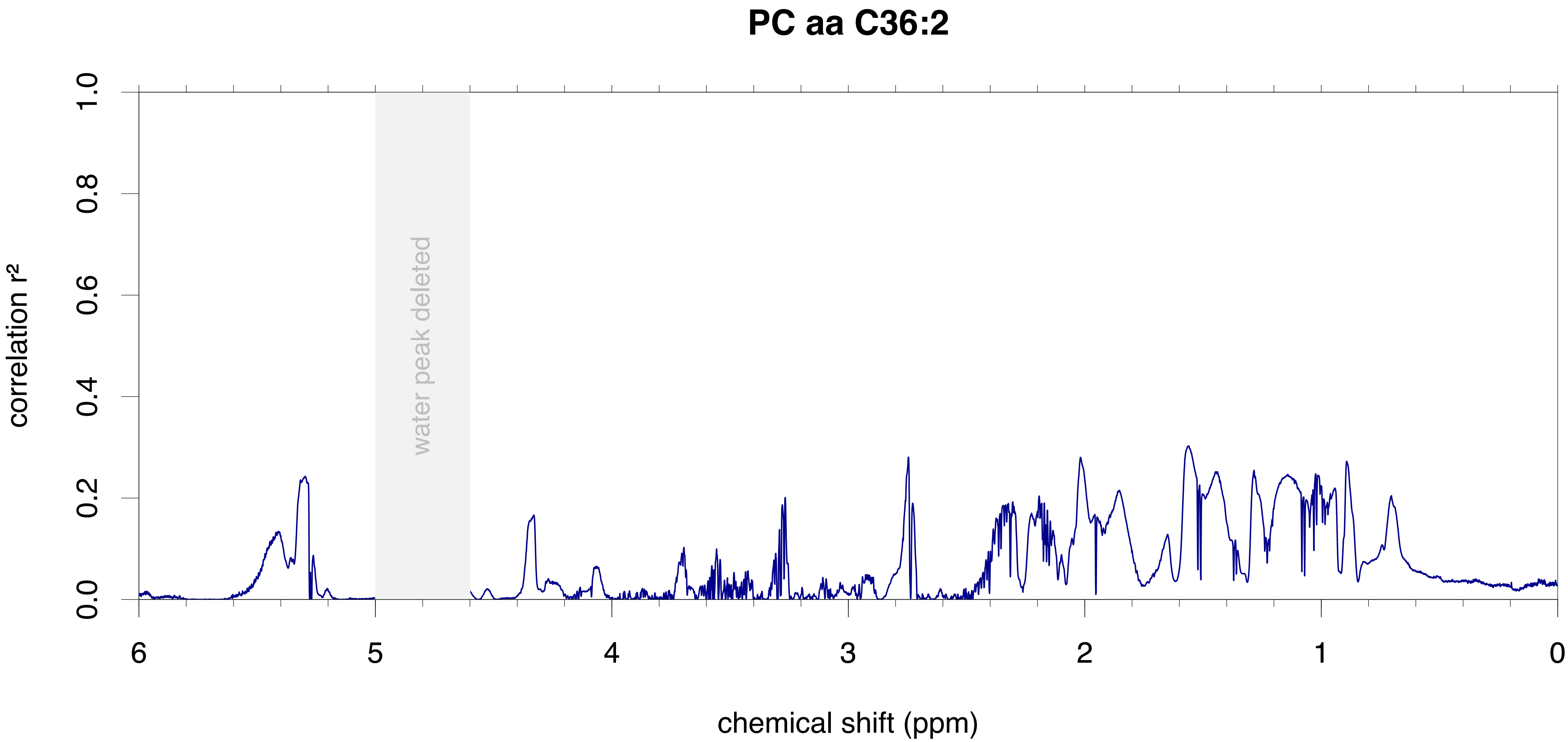
PC aa C36:2
correlation r²
1.0
0.8
0.6
0.4
0.2
0.0
water peak deleted
6
5
4
3
2
1
0
chemical shift (ppm)

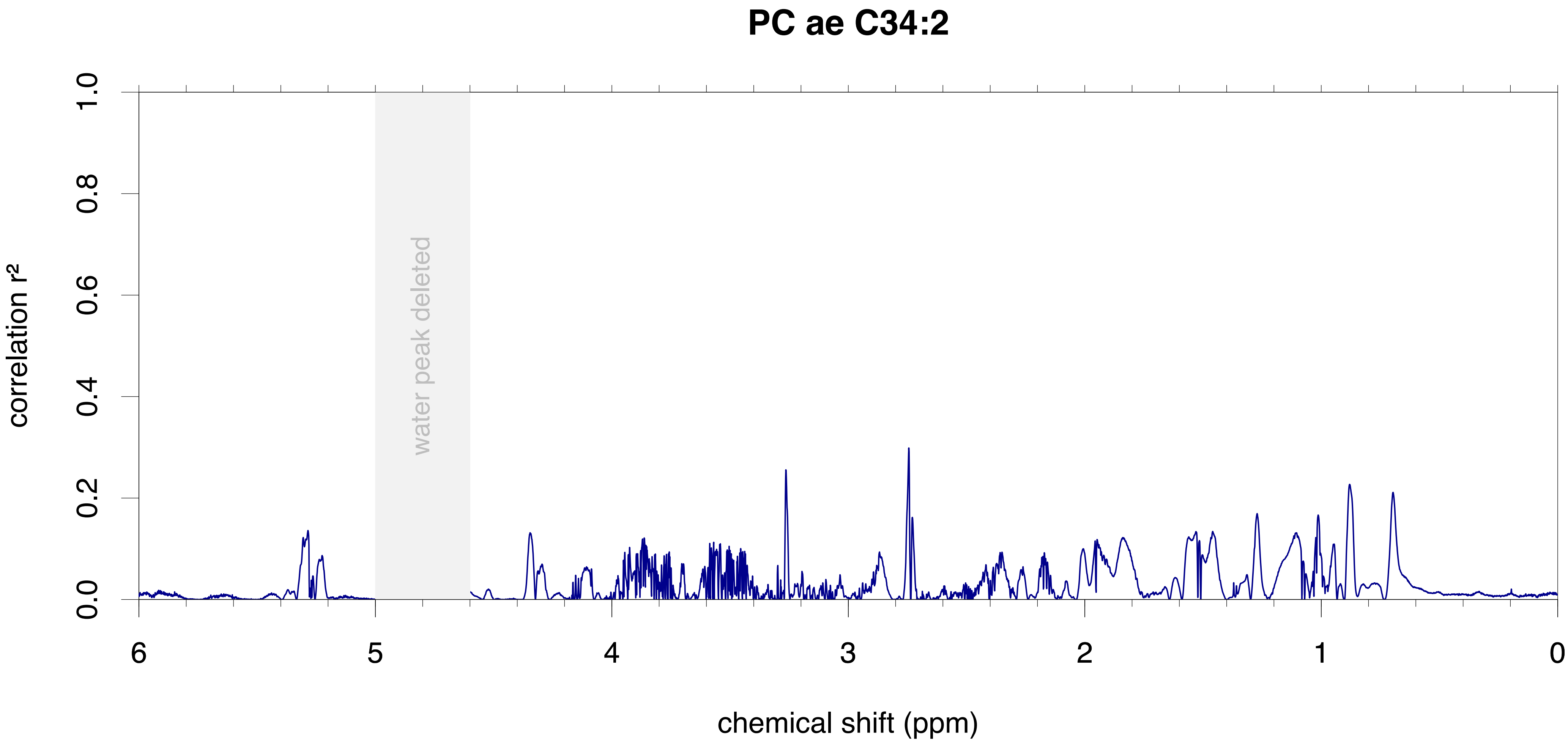

PC ae C34:2
water peak deleted
correlation r²
1.0
0.8
0.6
0.4
0.2
0.0
6
5
4
3
2
1
0
chemical shift (ppm)

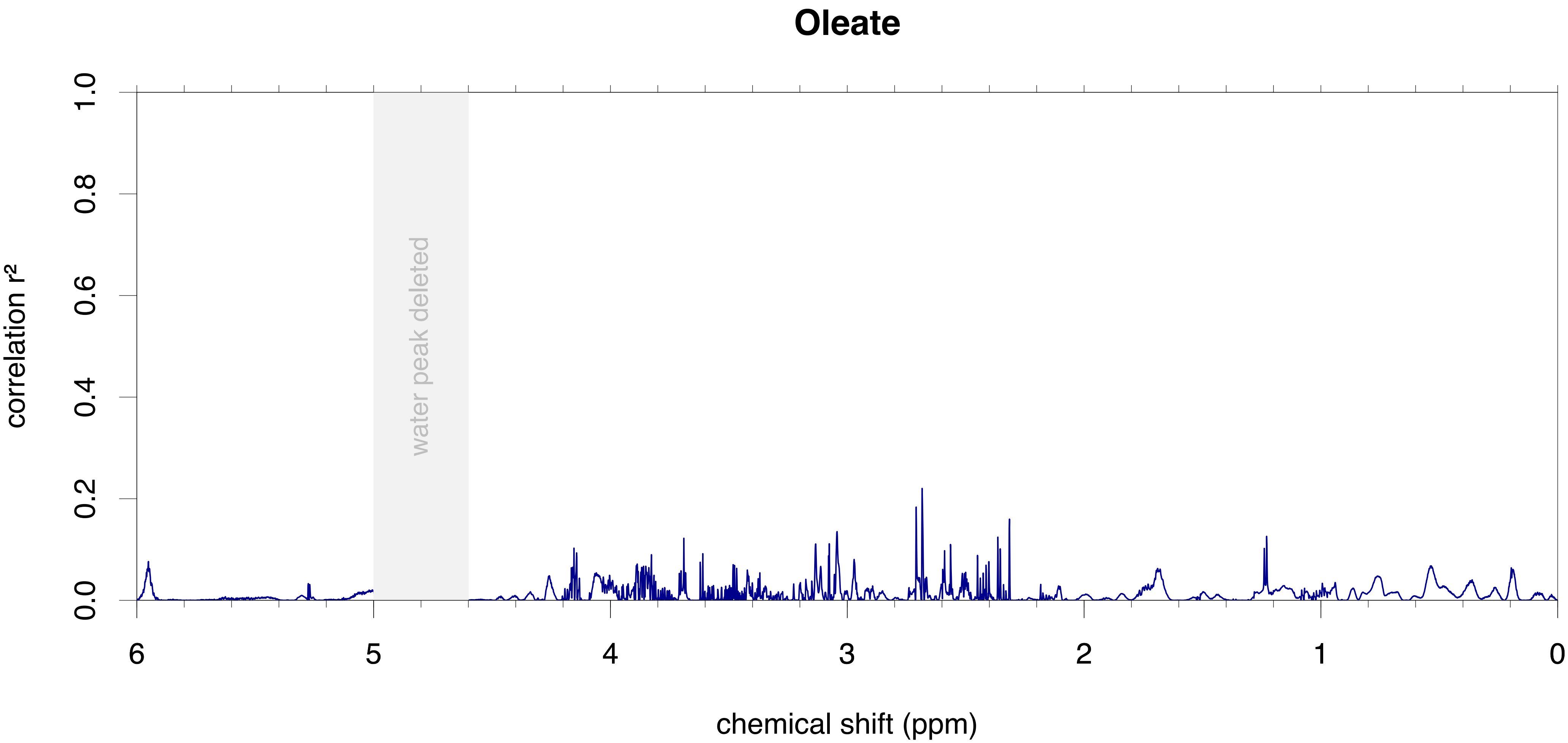

Oleate
correlation r²
1.0
0.8
0.6
0.4
0.2
0.0
water peak deleted
6
5
4
3
2
1
0
chemical shift (ppm)

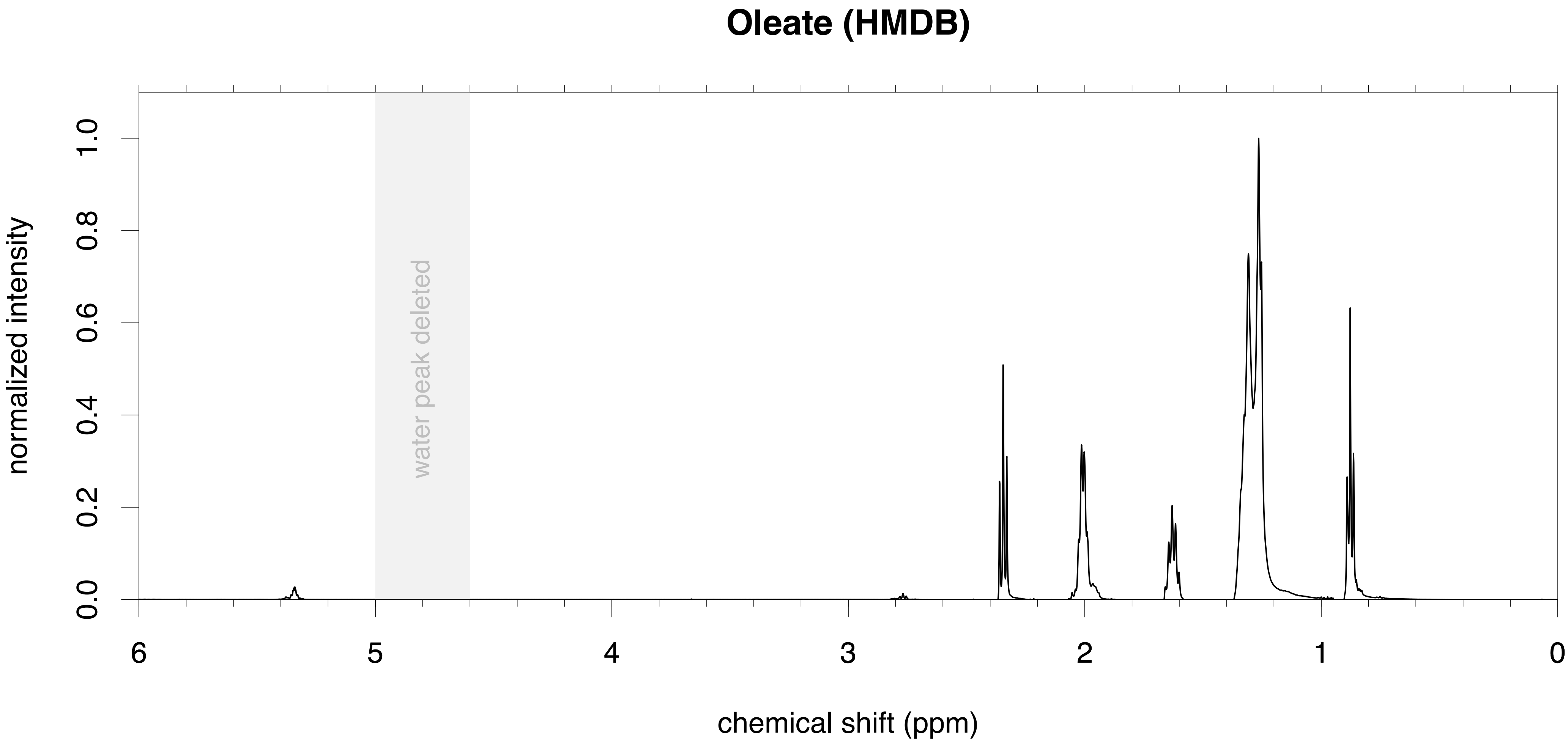

Oleate (HMDB)
normalized intensity
0.0
0.2
0.4
0.6
0.8
1.0
water peak deleted
6
5
4
3
2
1
0
chemical shift (ppm)

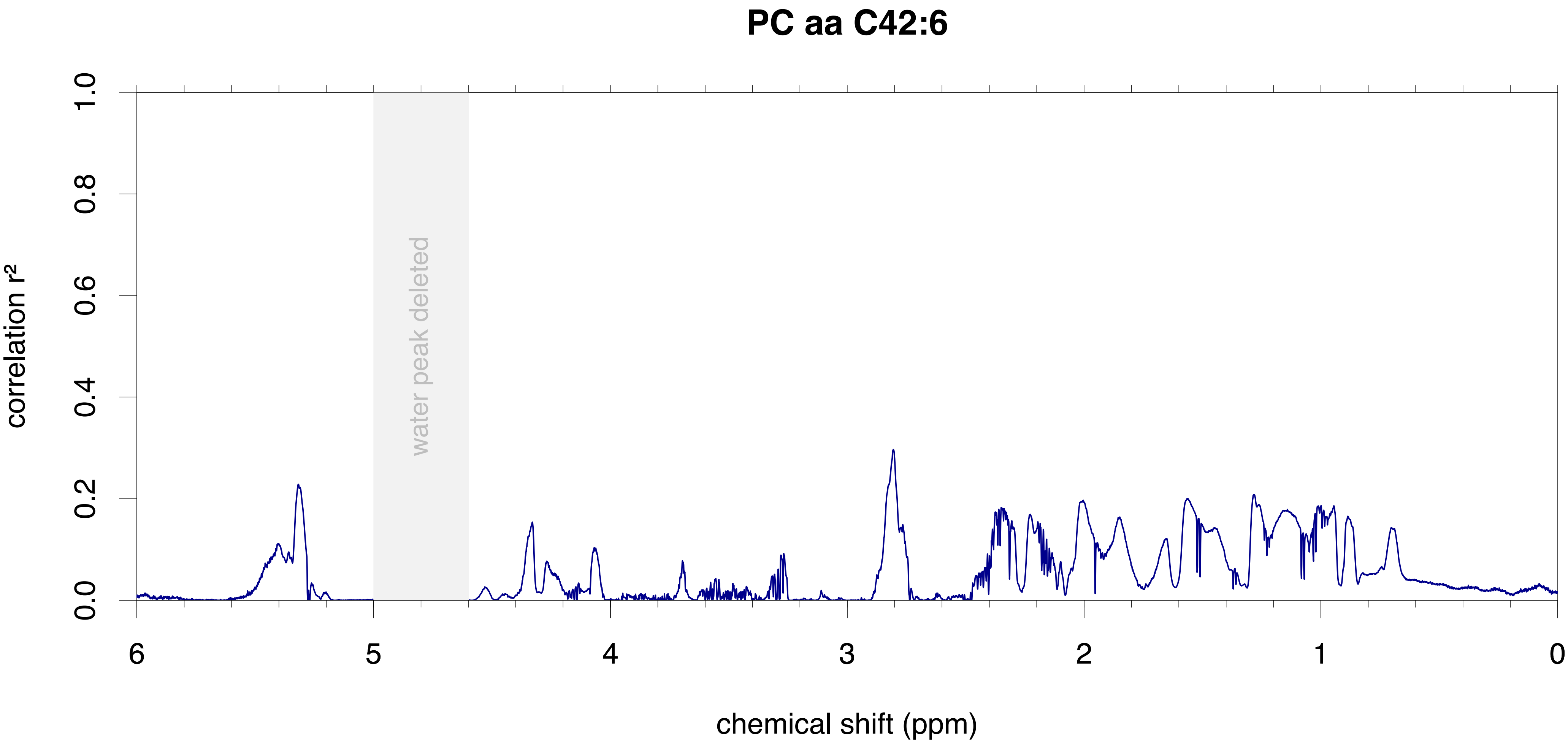
PC aa C42:6
correlation r²
1.0
0.8
0.6
0.4
0.2
0.0
water peak deleted
6
5
4
3
2
1
0
chemical shift (ppm)

PC ae C42:1

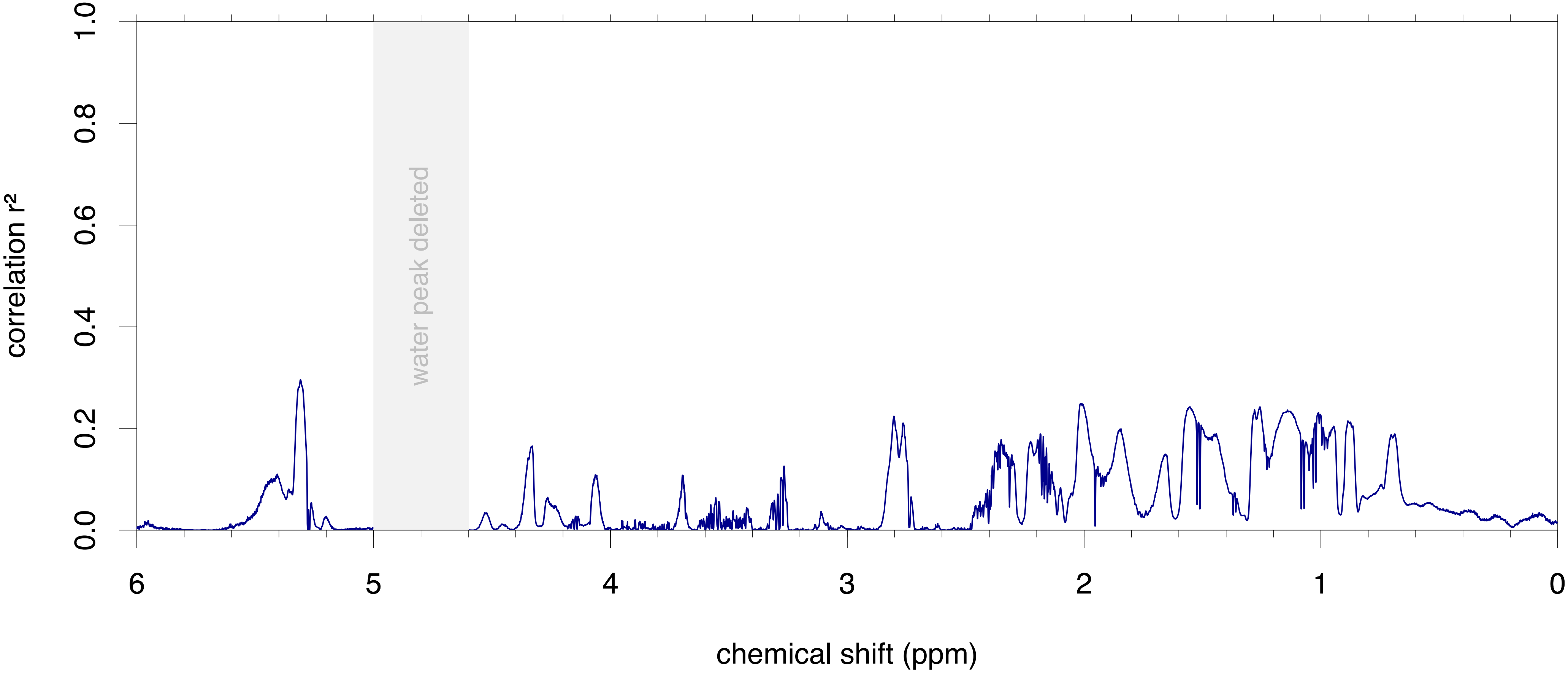

water peak deleted
correlation r²
0.0
0.2
0.4
0.6
0.8
1.0
6
5
4
3
2
1
0
chemical shift (ppm)

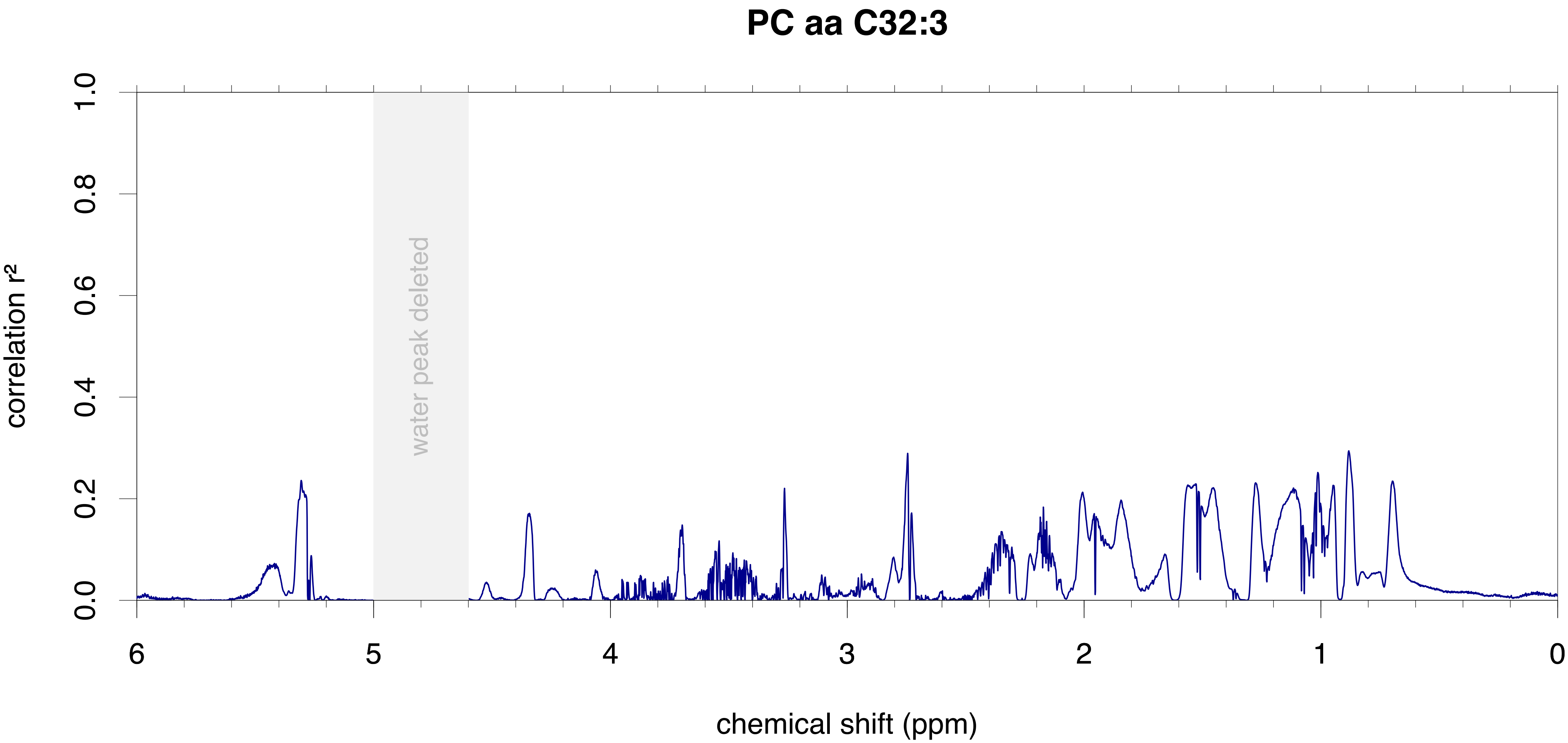

PC aa C32:3
correlation r²
1.0
0.8
0.6
0.4
0.2
0.0
water peak deleted
6
5
4
3
2
1
0
chemical shift (ppm)

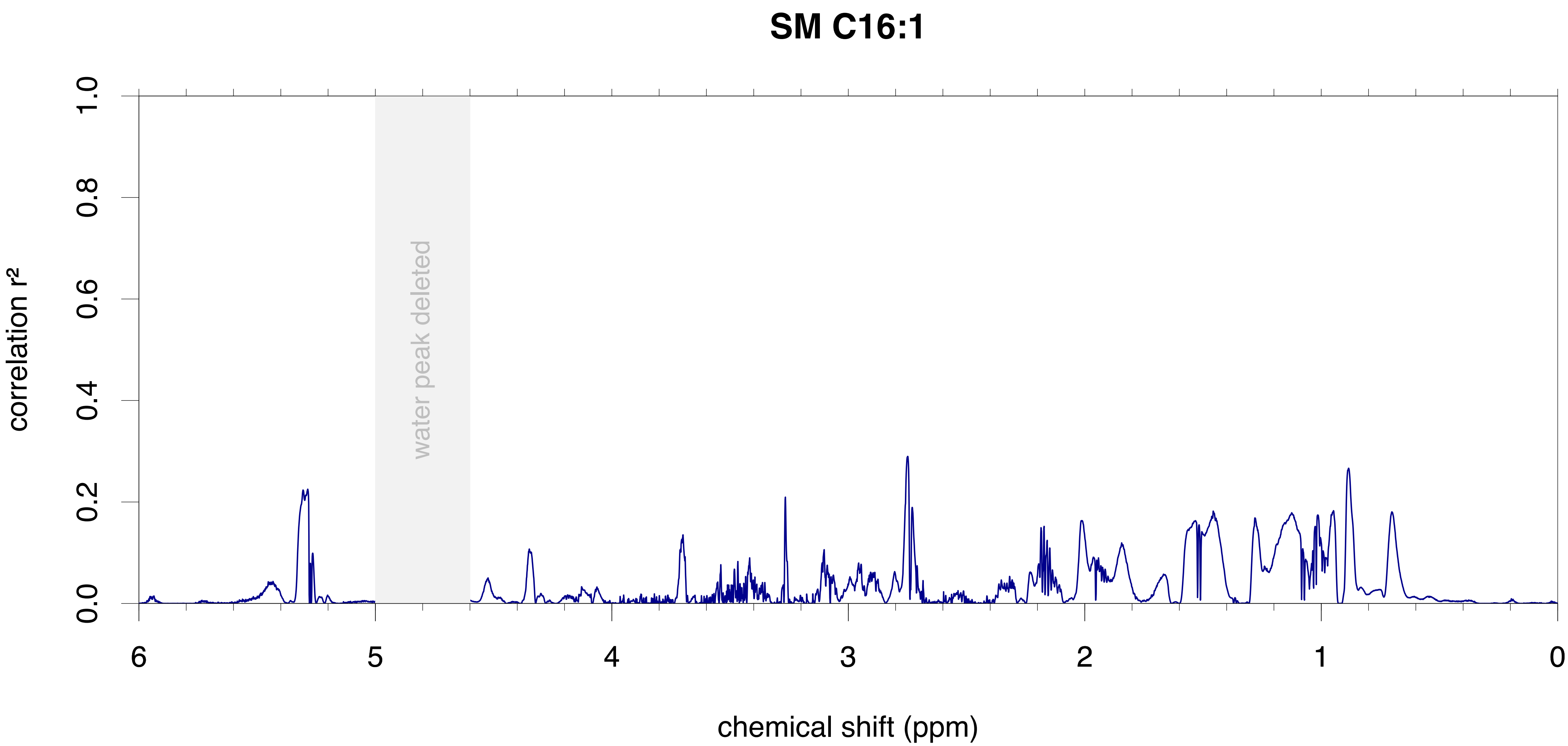

SM C16:1
correlation r²
1.0
0.8
0.6
0.4
0.2
0.0
water peak deleted
6
5
4
3
2
1
0
chemical shift (ppm)

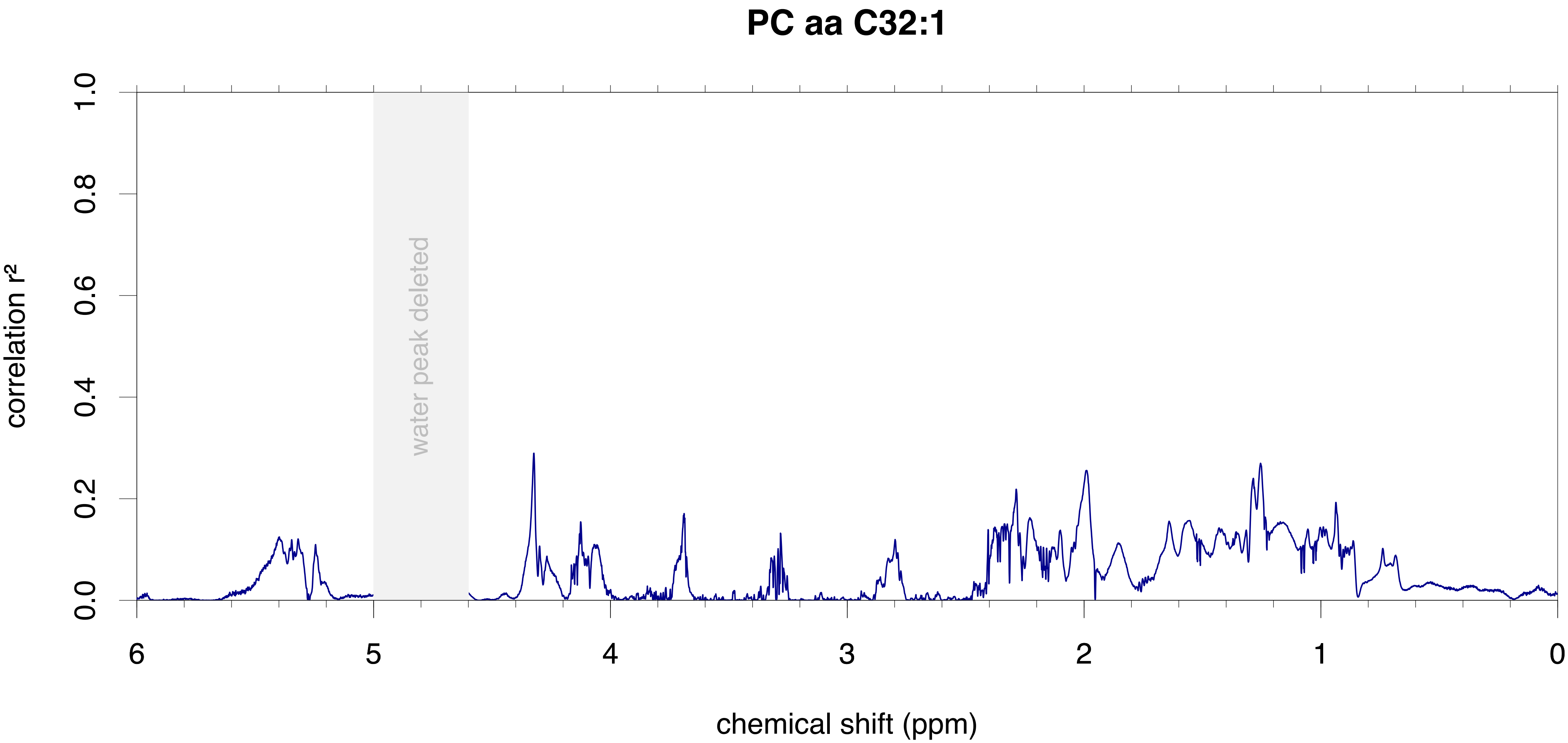
PC aa C32:1
correlation r²
0.0
0.2
0.4
0.6
0.8
1.0
water peak deleted
6
5
4
3
2
1
0
chemical shift (ppm)

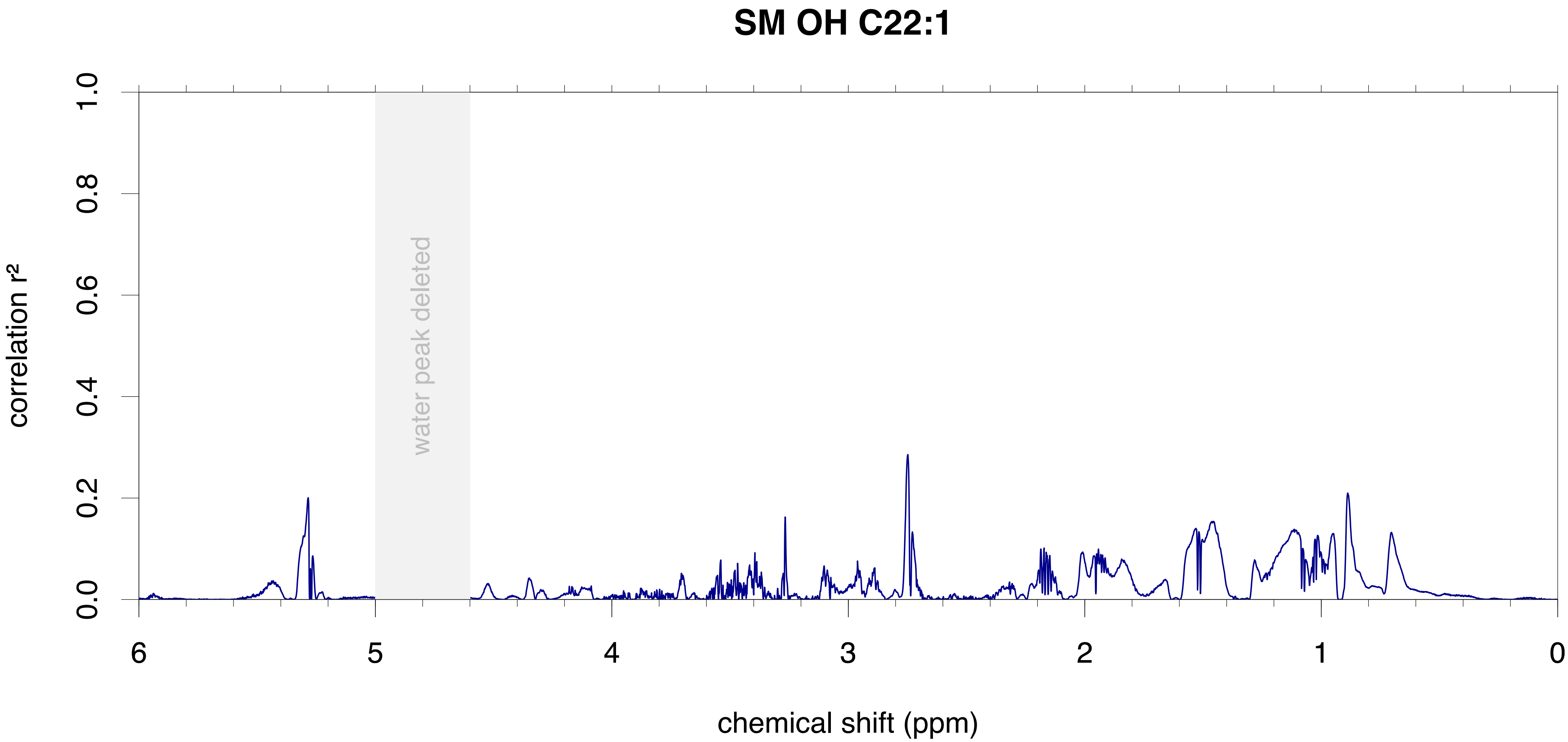

SM OH C22:1
correlation r²
0.0
0.2
0.4
0.6
0.8
1.0
water peak deleted
6
5
4
3
2
1
0
chemical shift (ppm)

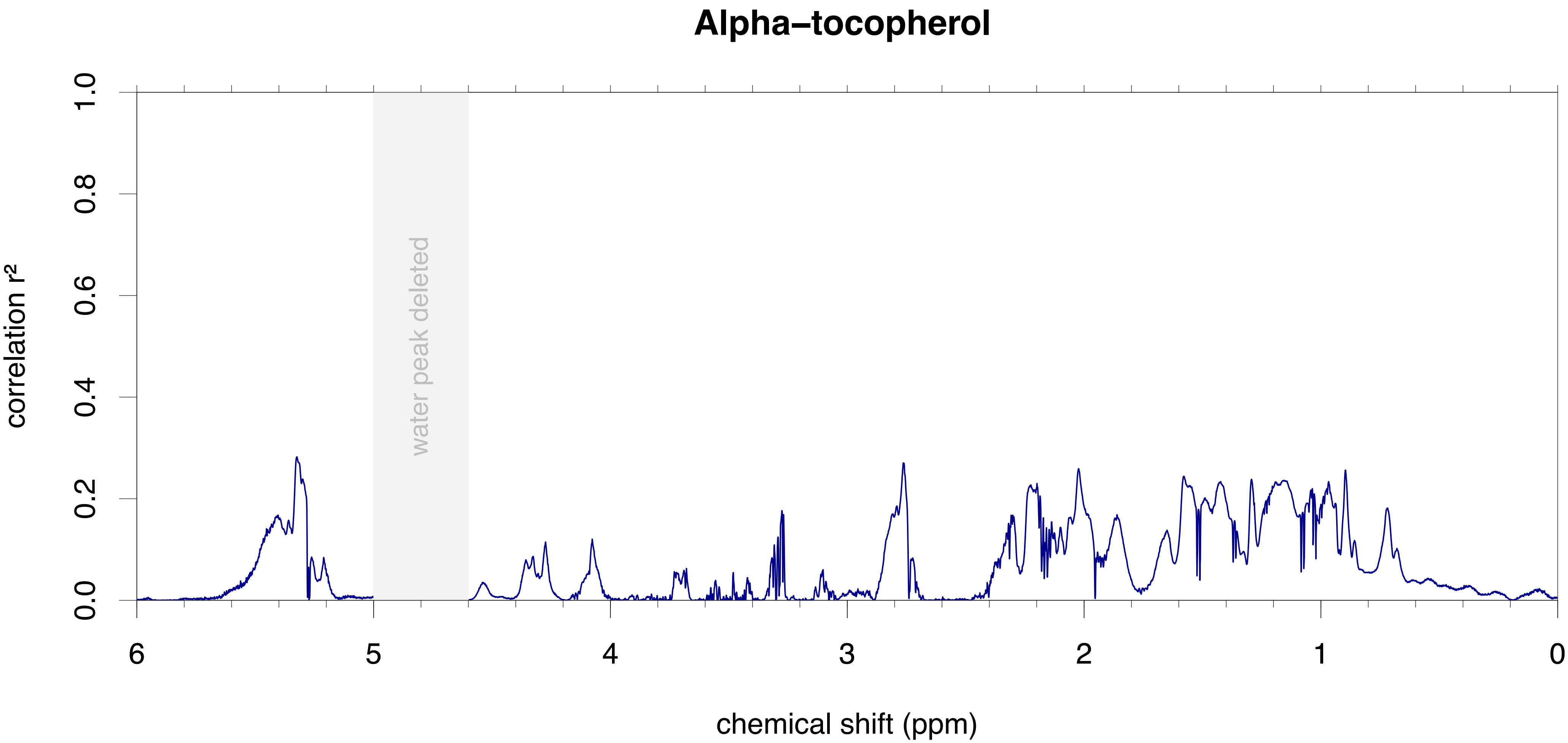

Alpha–tocopherol
correlation r²
0.0
0.2
0.4
0.6
0.8
1.0
water peak deleted
6
5
4
3
2
1
0
chemical shift (ppm)

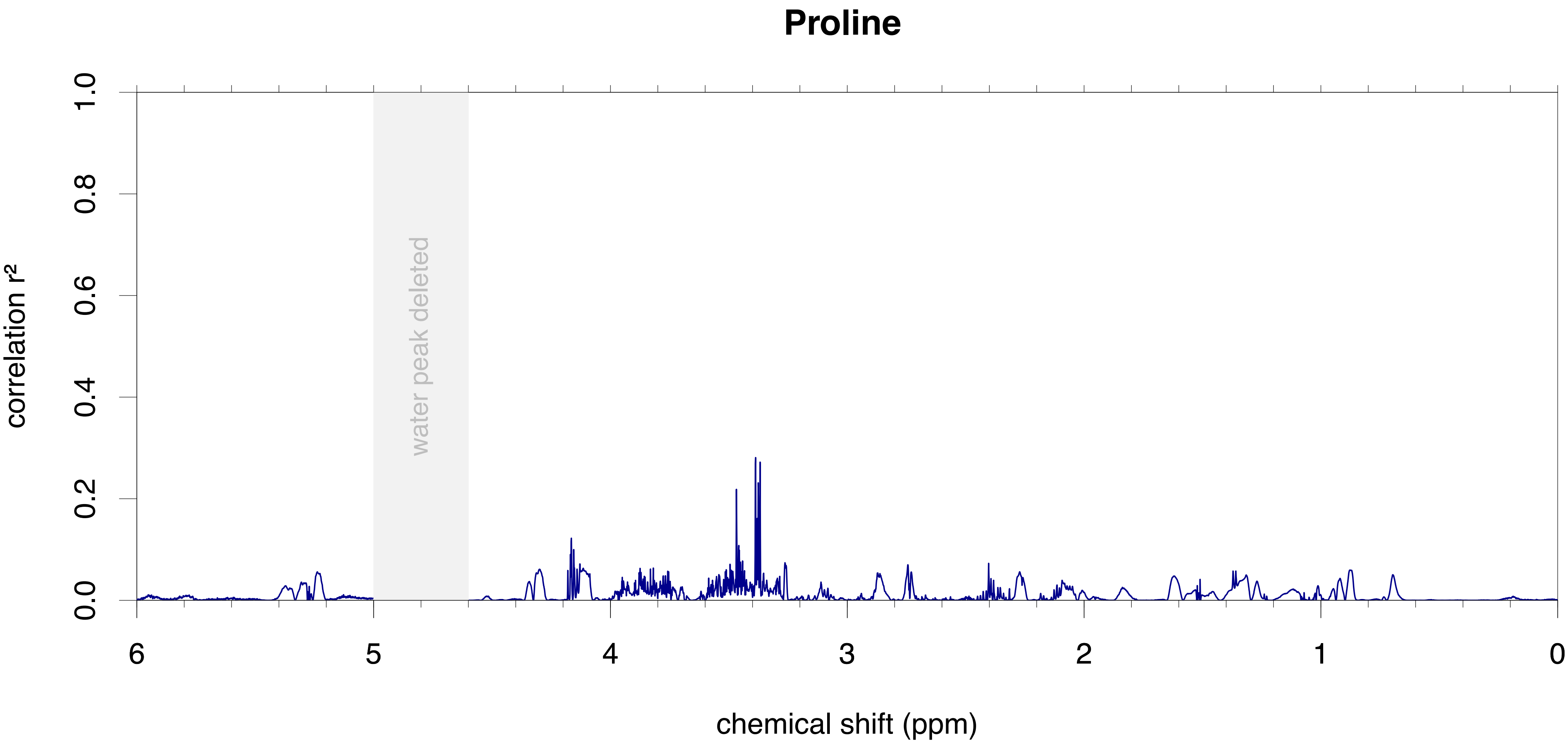

Proline
1.0
0.8
0.6
0.4
0.2
0.0
correlation r²
water peak deleted
6
5
4
3
2
1
0
chemical shift (ppm)

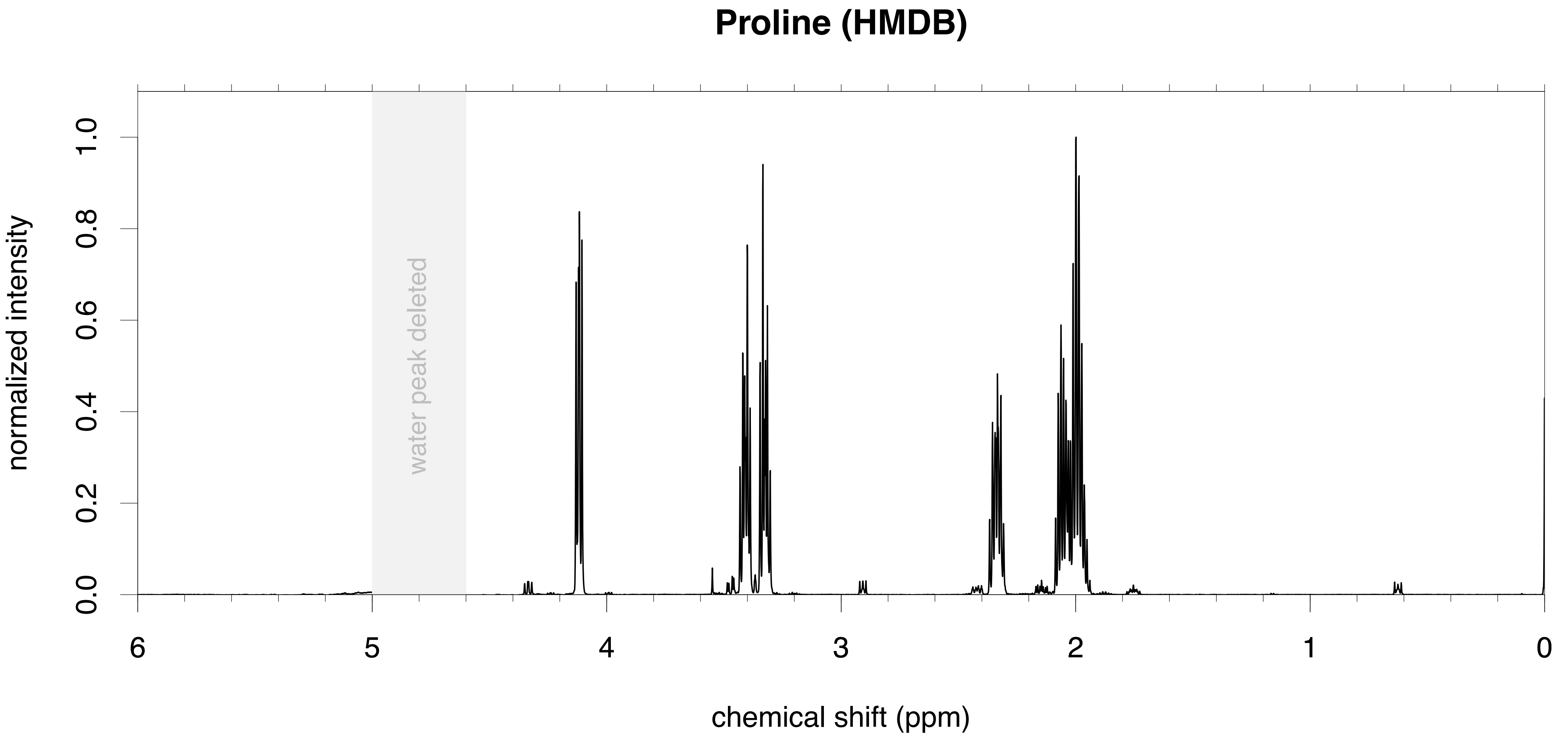

Proline (HMDB)
normalized intensity
0.0
0.2
0.4
0.6
0.8
1.0
water peak deleted
6
5
4
3
2
1
0
chemical shift (ppm)

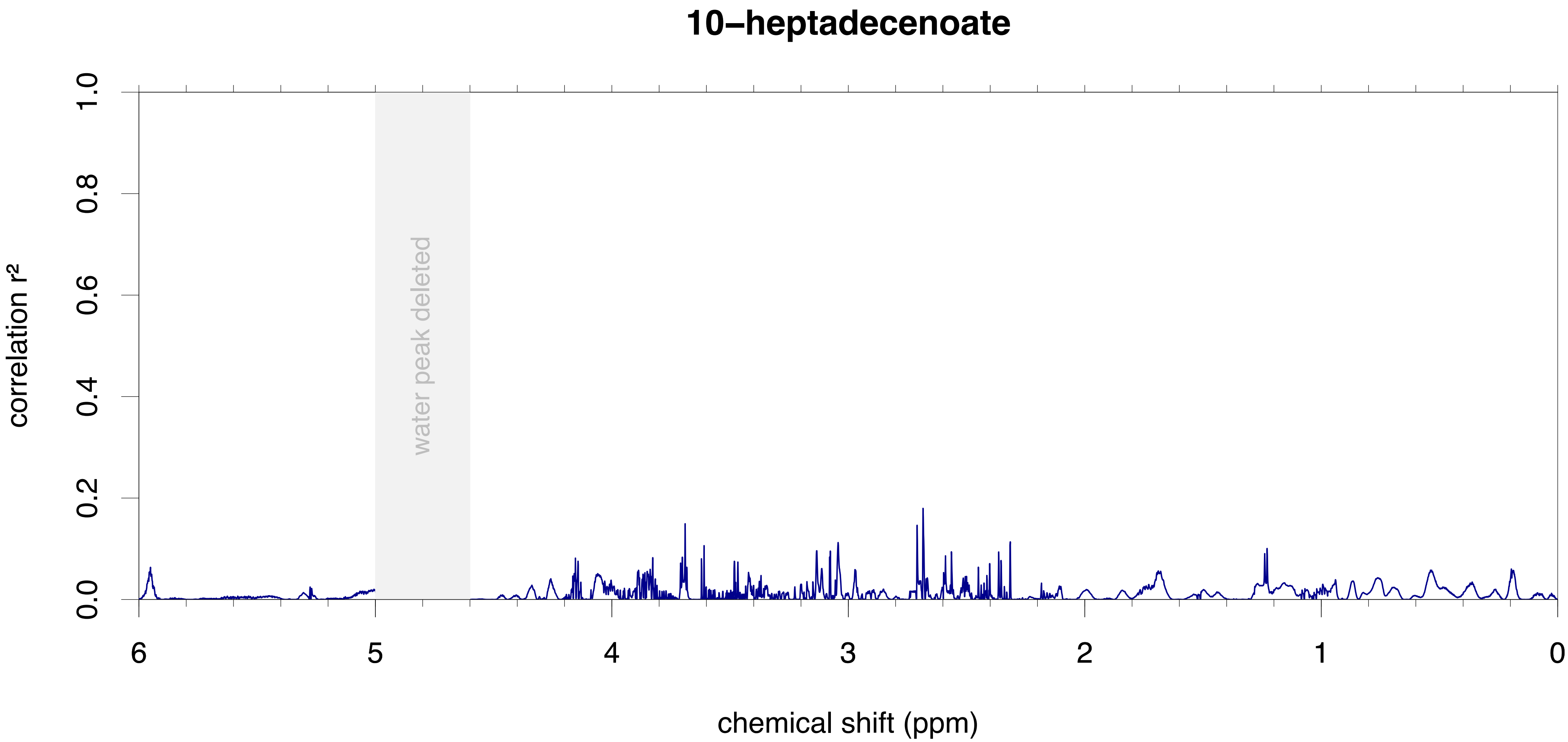

10−heptadecenoate
correlation r²
water peak deleted
chemical shift (ppm)
0.0
0.2
0.4
0.6
0.8
1.0
6
5
4
3
2
1
0

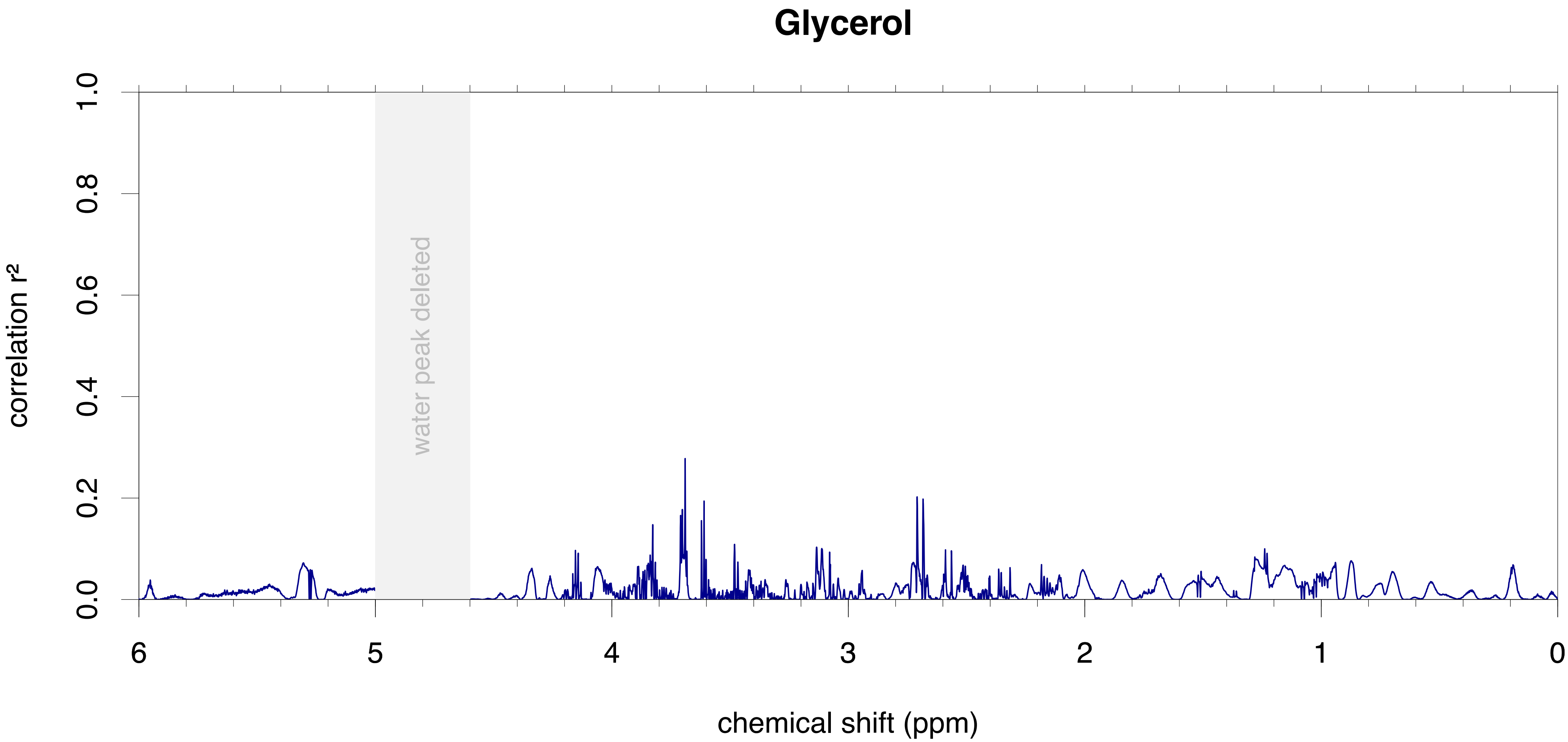
Glycerol
correlation r²
0.0
0.2
0.4
0.6
0.8
1.0
water peak deleted
6
5
4
3
2
1
0
chemical shift (ppm)

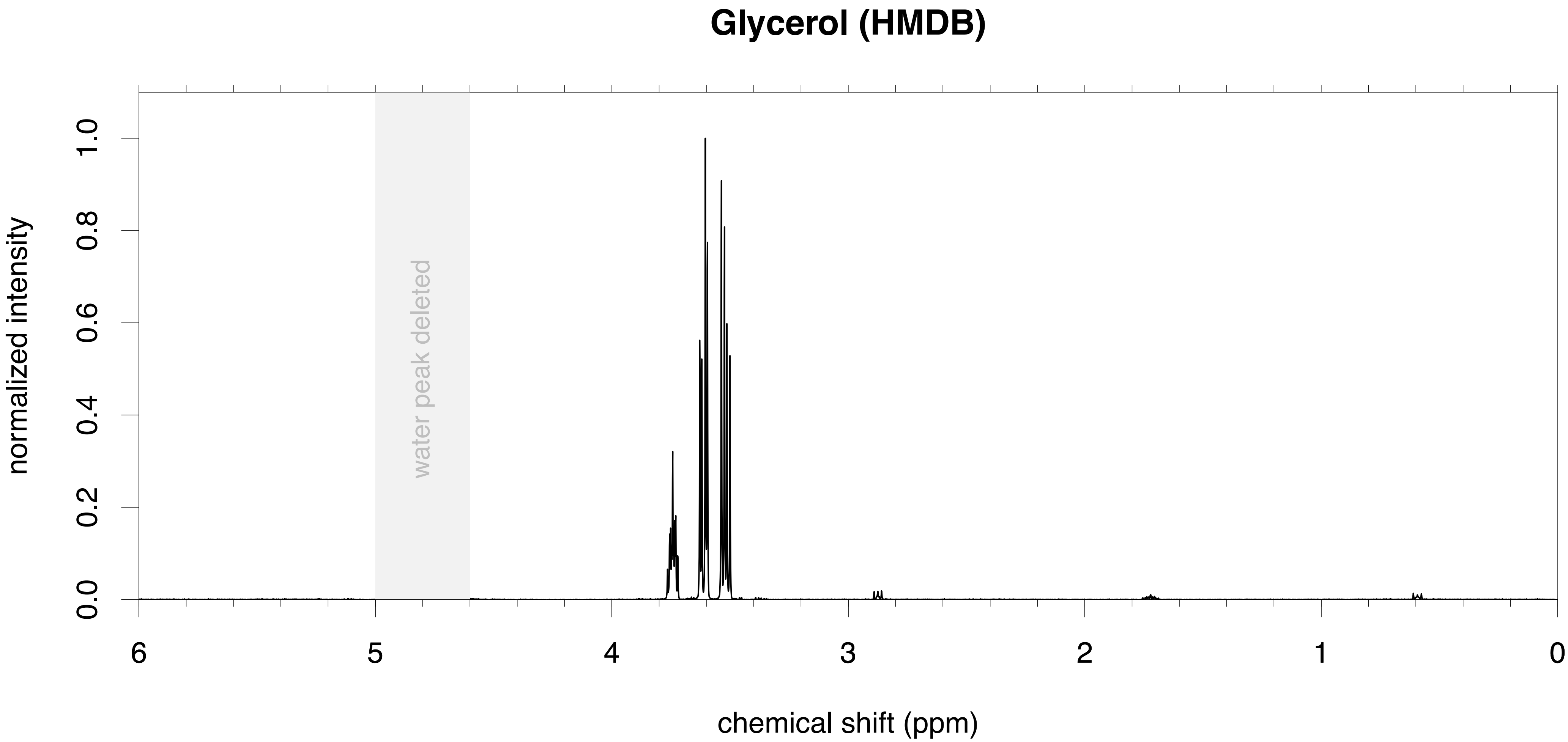

Glycerol (HMDB)
normalized intensity
0.0
0.2
0.4
0.6
0.8
1.0
water peak deleted
6
5
4
3
2
1
0
chemical shift (ppm)

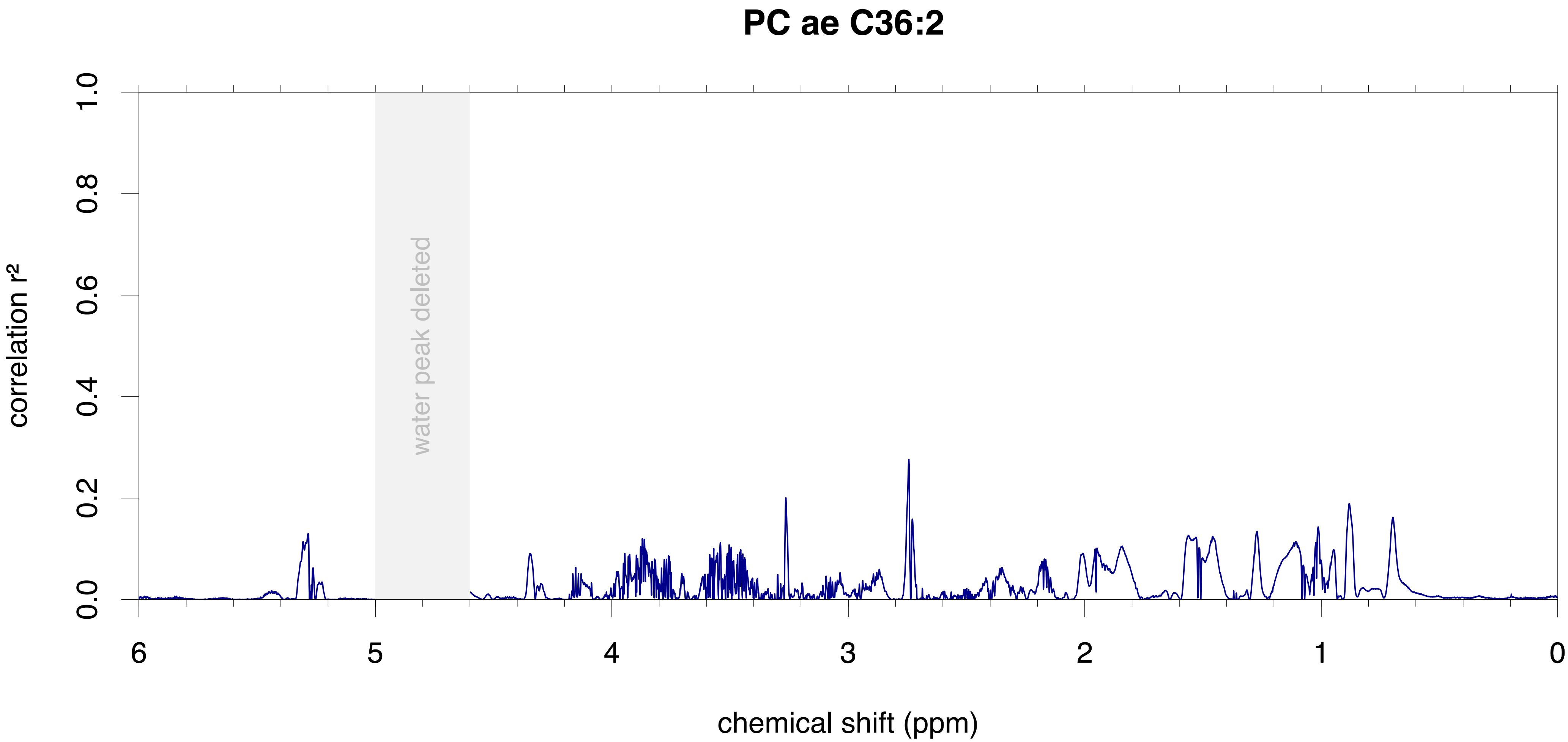
PC ae C36:2
correlation r²
1.0
0.8
0.6
0.4
0.2
0.0
water peak deleted
6
5
4
3
2
1
0
chemical shift (ppm)

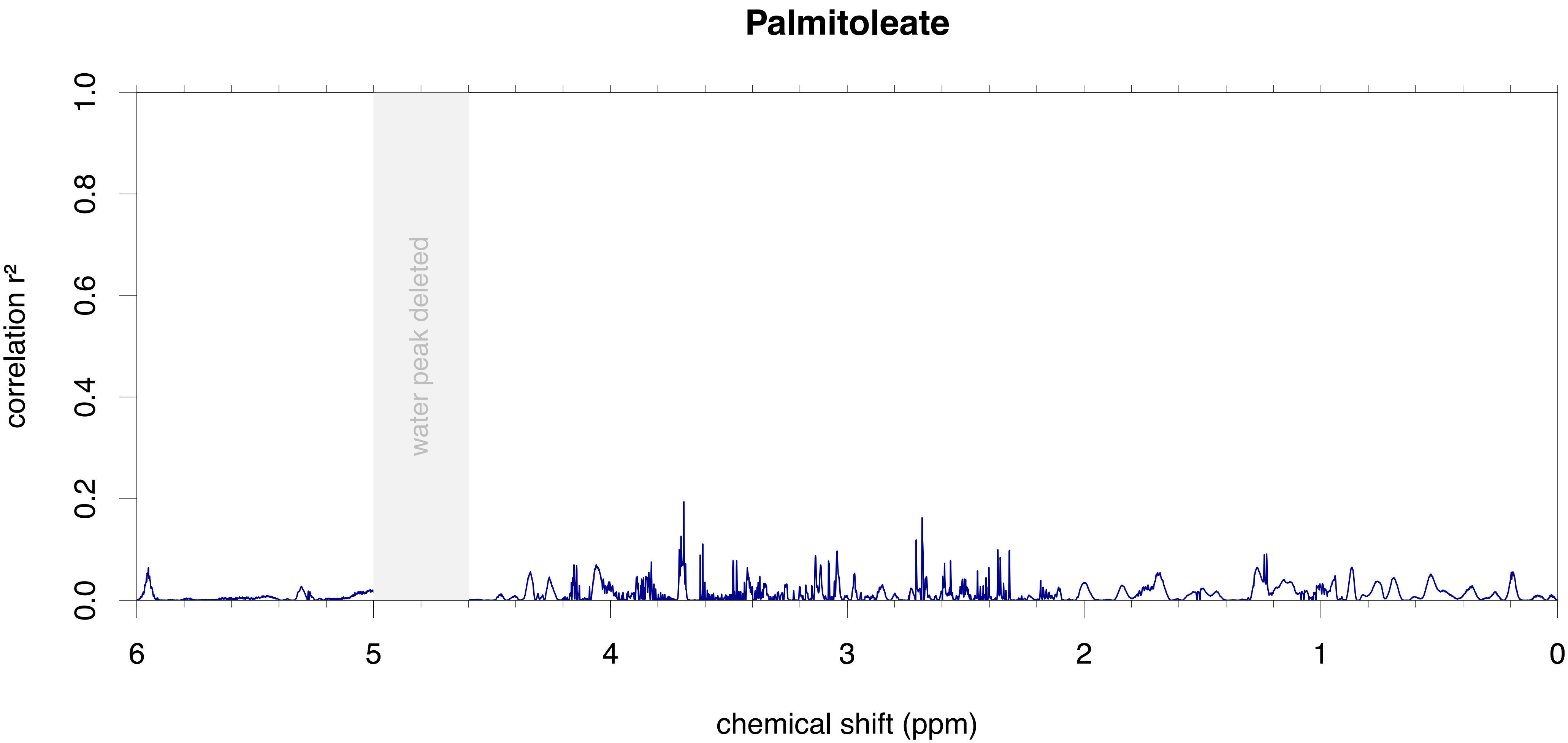

Palmitoleate
correlation r²
1.0
0.8
0.6
0.4
0.2
0.0
water peak deleted
6
5
4
3
2
1
0
chemical shift (ppm)

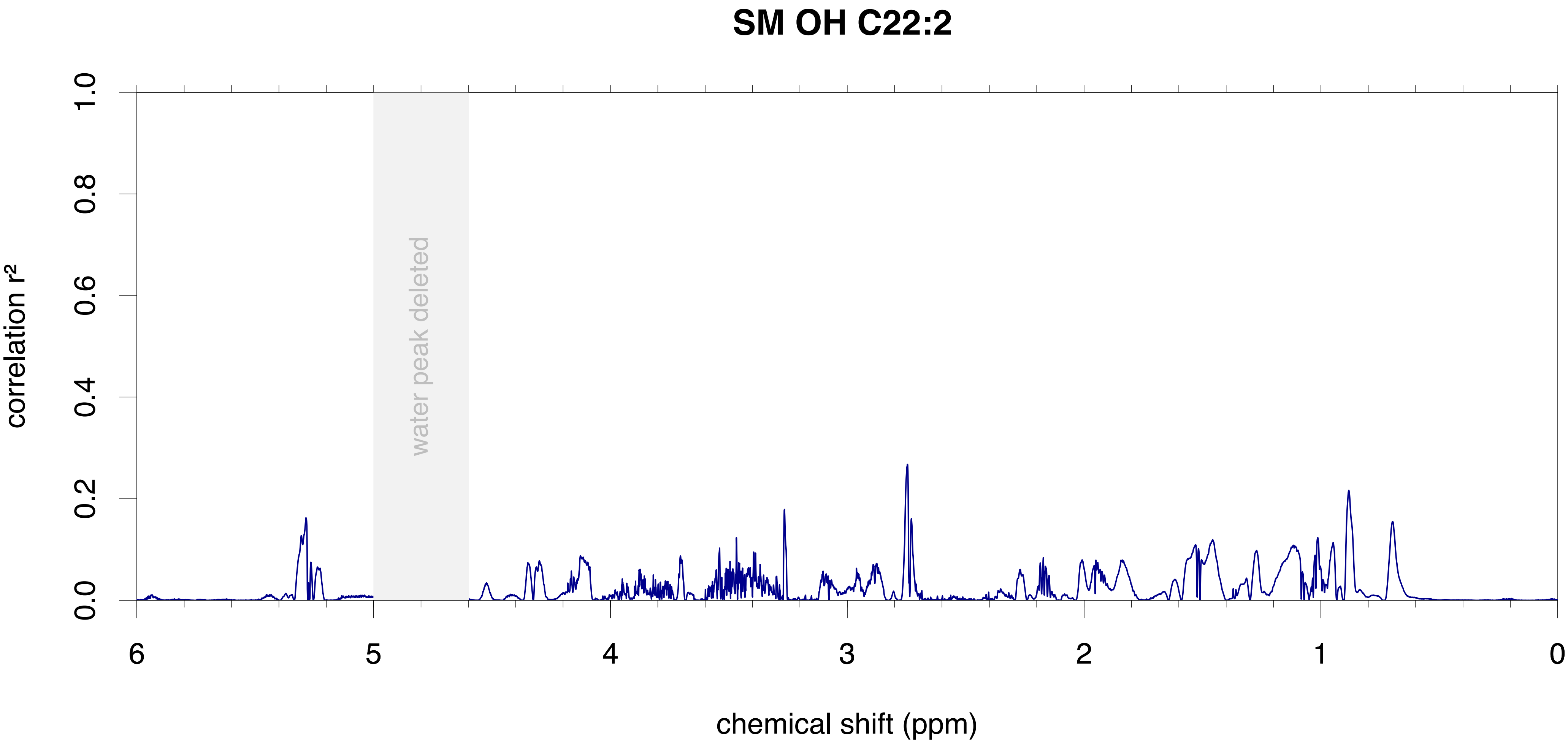
SM OH C22:2
1.0
0.8
0.6
0.4
0.2
0.0
correlation r²
water peak deleted
6
5
4
3
2
1
0
chemical shift (ppm)

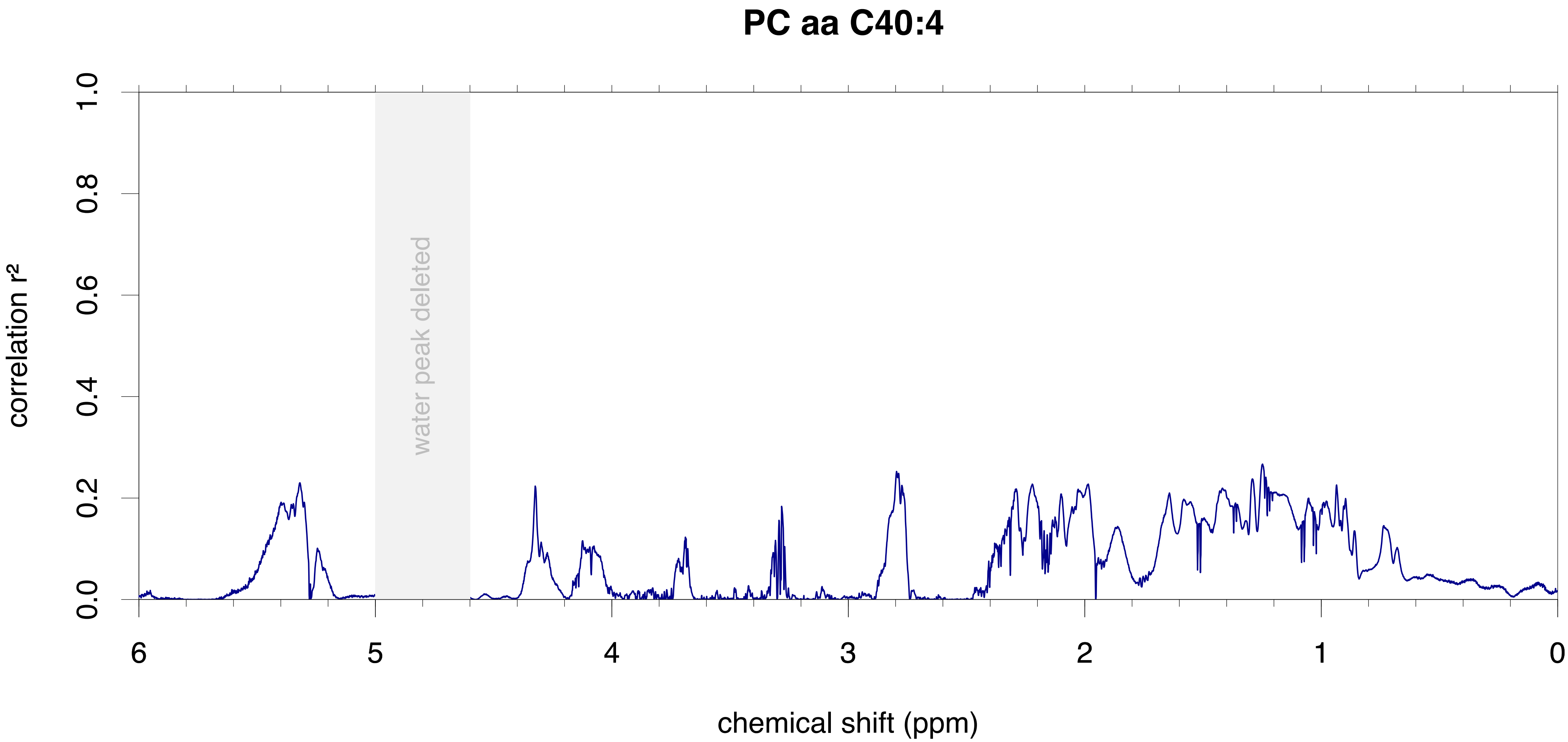
PC aa C40:4
correlation r²
0.0
0.2
0.4
0.6
0.8
1.0
water peak deleted
6
5
4
3
2
1
0
chemical shift (ppm)

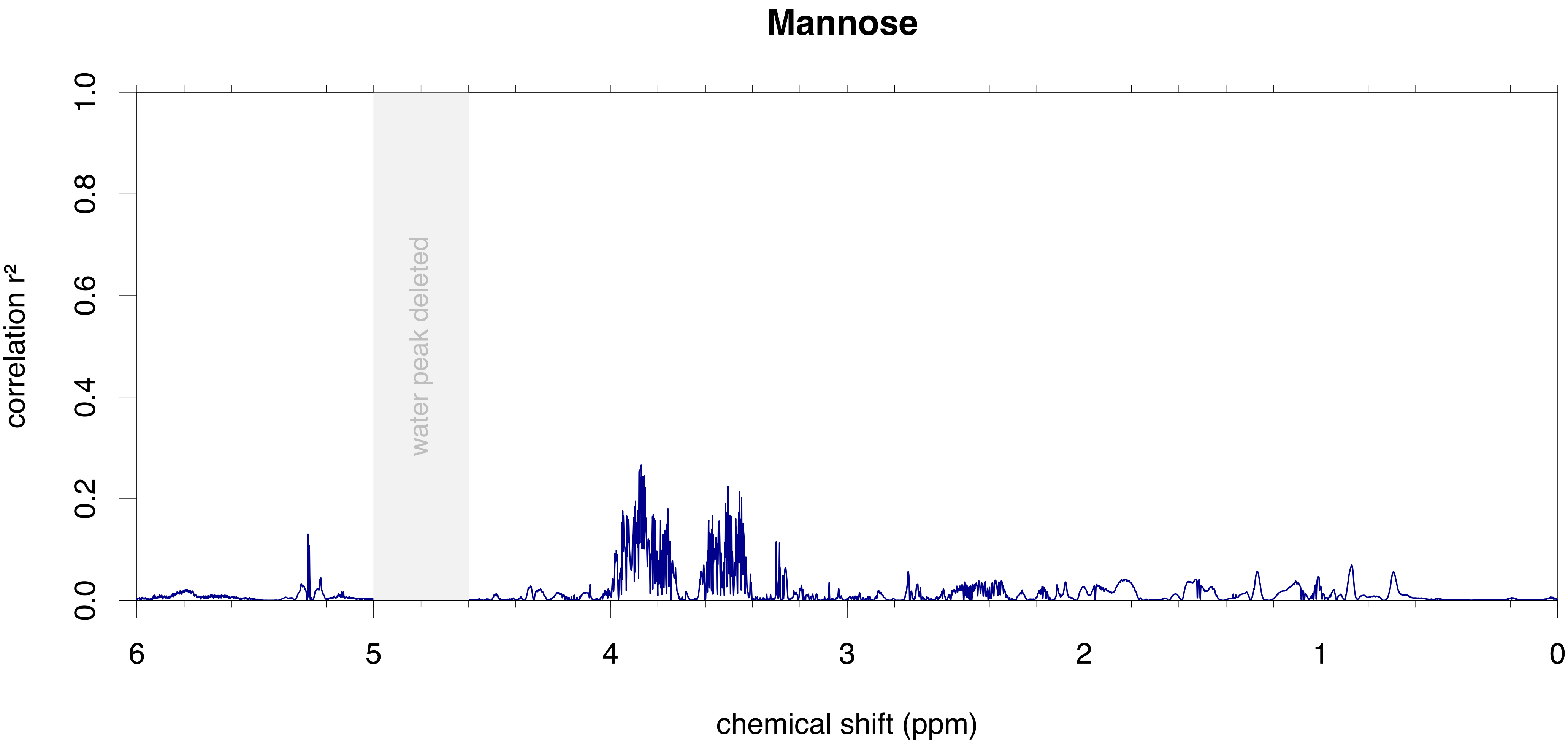

Mannose
correlation r²
1.0
0.8
0.6
0.4
0.2
0.0
water peak deleted
6
5
4
3
2
1
0
chemical shift (ppm)

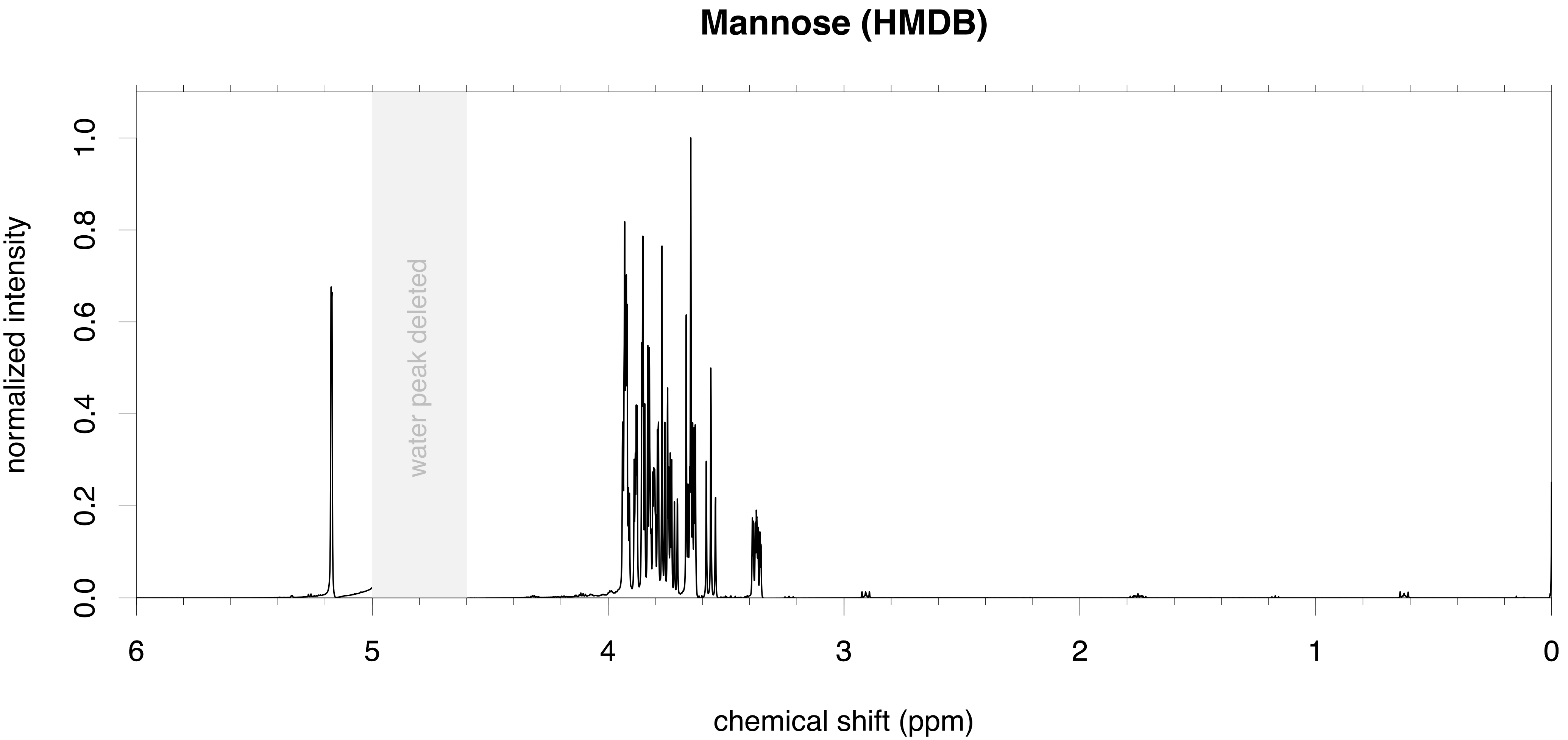

Mannose (HMDB)
normalized intensity
0.0
0.2
0.4
0.6
0.8
1.0
water peak deleted
6
5
4
3
2
1
0
chemical shift (ppm)

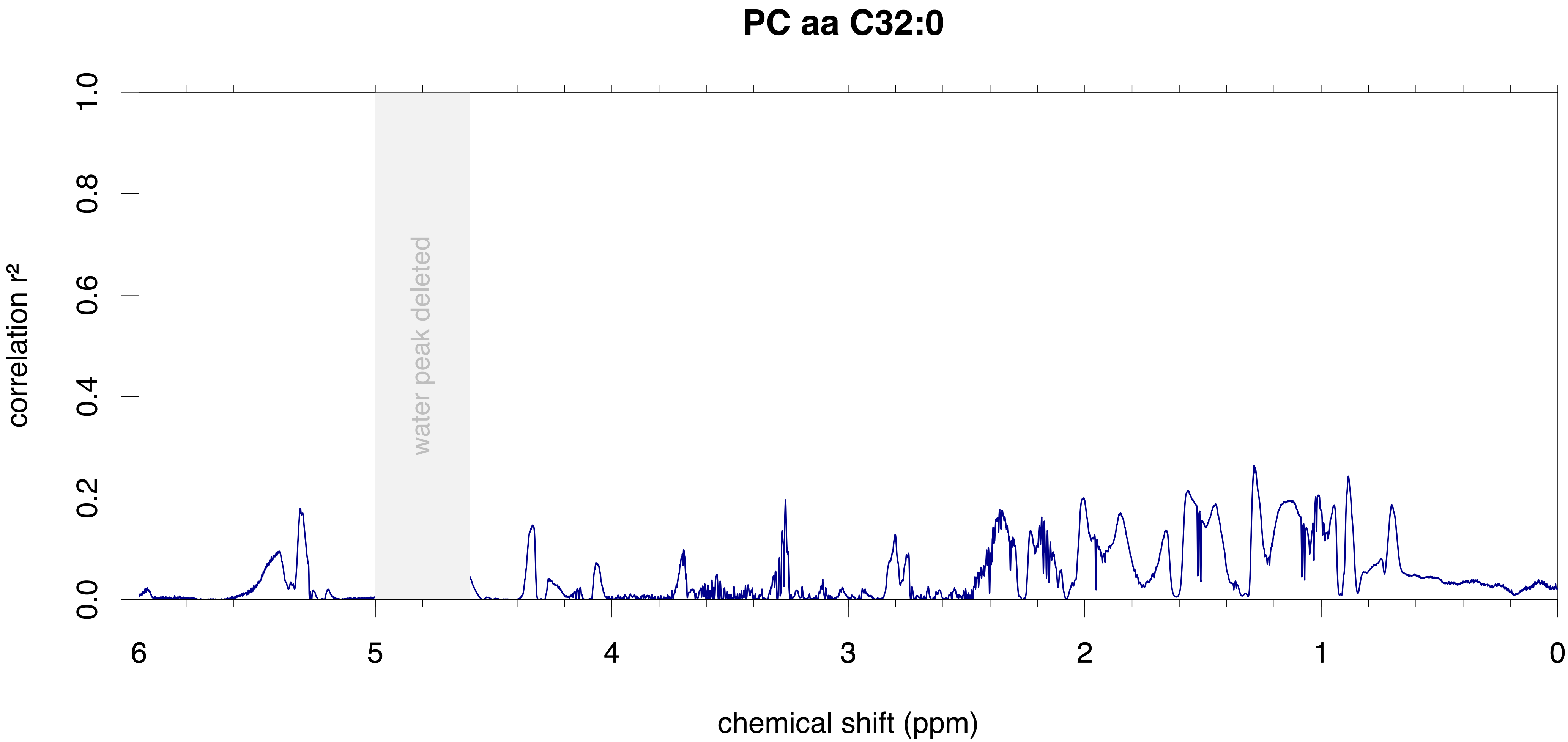
PC aa C32:0
correlation r²
1.0
0.8
0.6
0.4
0.2
0.0
water peak deleted
6
5
4
3
2
1
0
chemical shift (ppm)

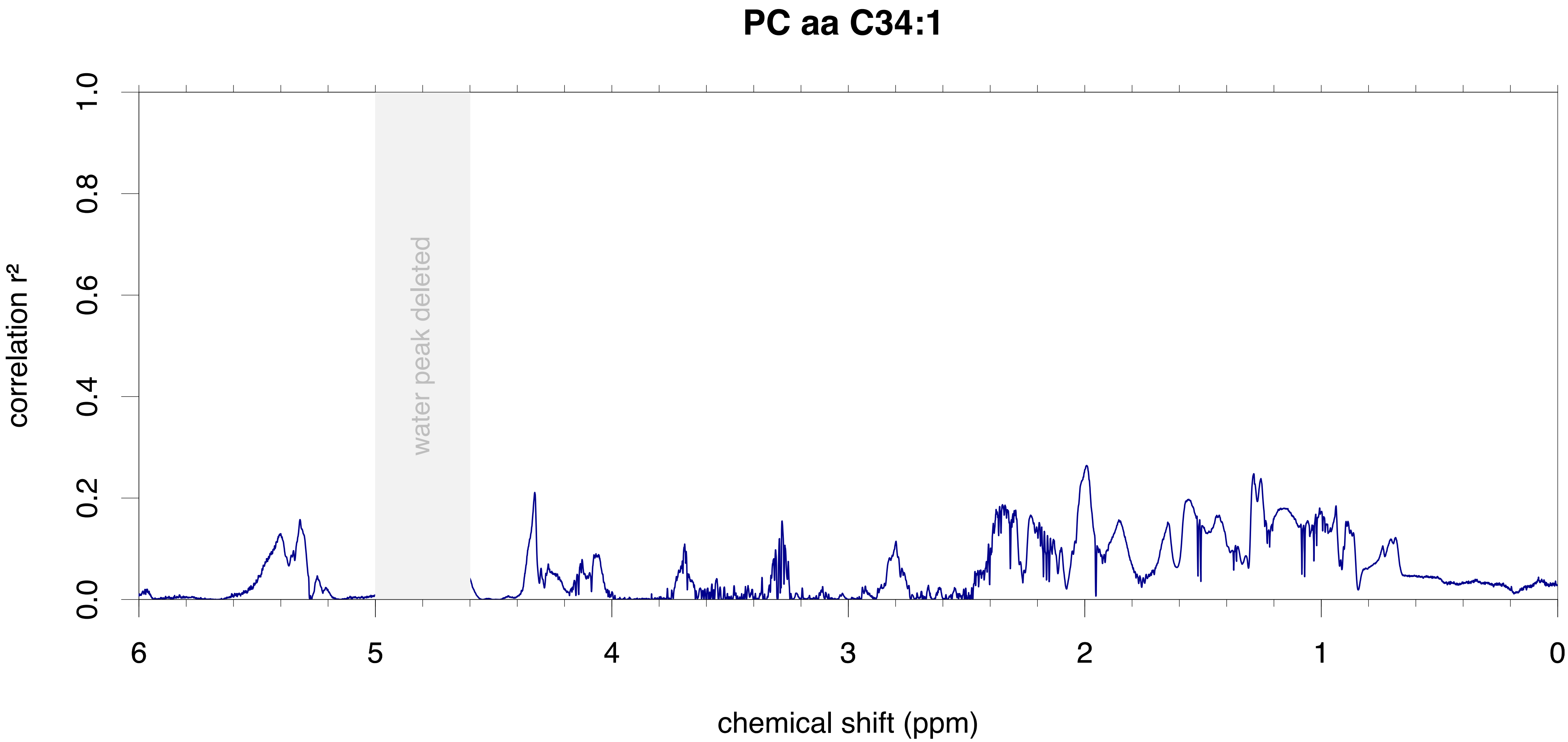
PC aa C34:1
correlation r²
1.0
0.8
0.6
0.4
0.2
0.0
water peak deleted
6
5
4
3
2
1
0
chemical shift (ppm)

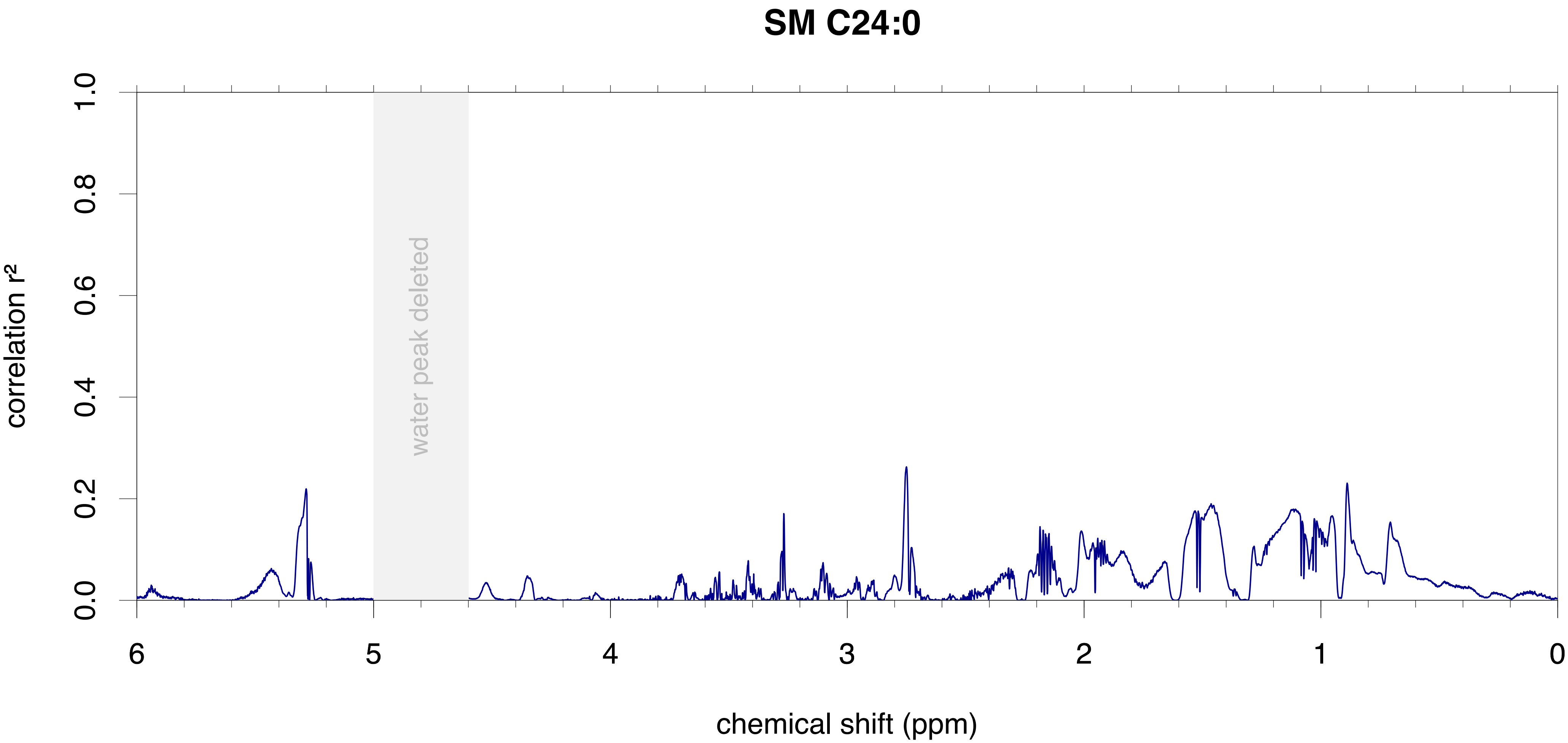

SM C24:0
correlation r²
1.0
0.8
0.6
0.4
0.2
0.0
water peak deleted
6
5
4
3
2
1
0
chemical shift (ppm)

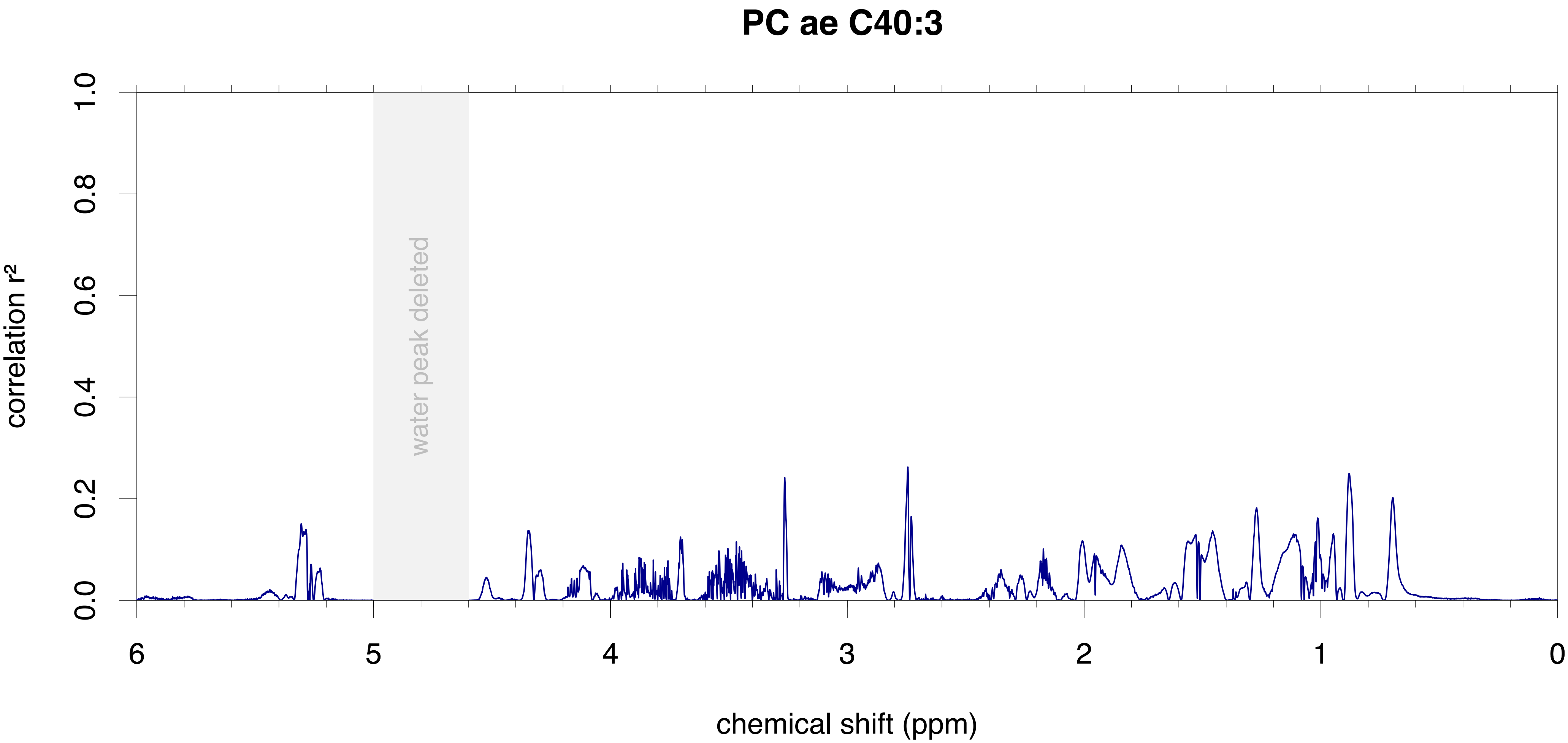
PC ae C40:3
correlation r²
1.0
0.8
0.6
0.4
0.2
0.0
water peak deleted
6
5
4
3
2
1
0
chemical shift (ppm)

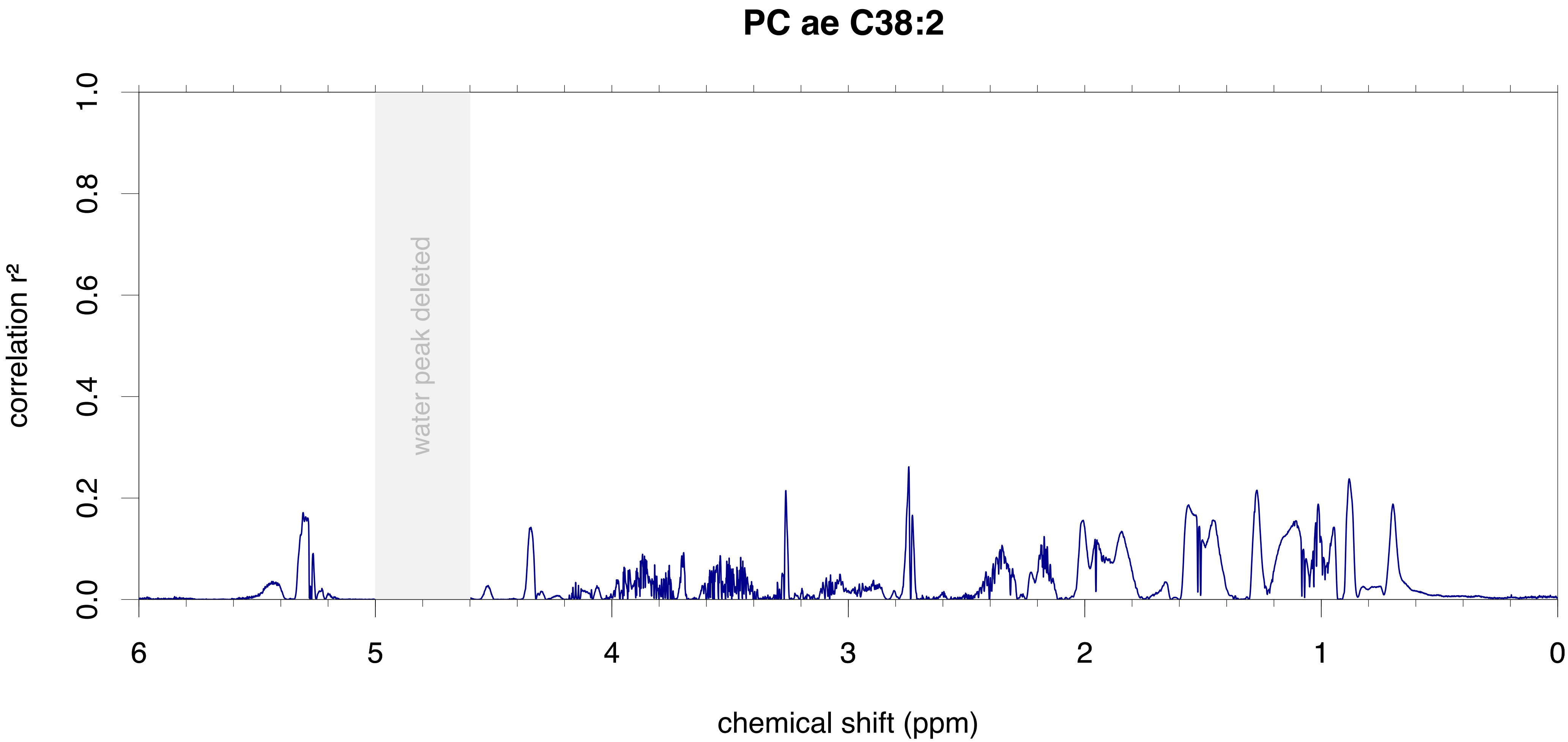

PC ae C38:2
correlation r²
1.0
0.8
0.6
0.4
0.2
0.0
water peak deleted
6
5
4
3
2
1
0
chemical shift (ppm)

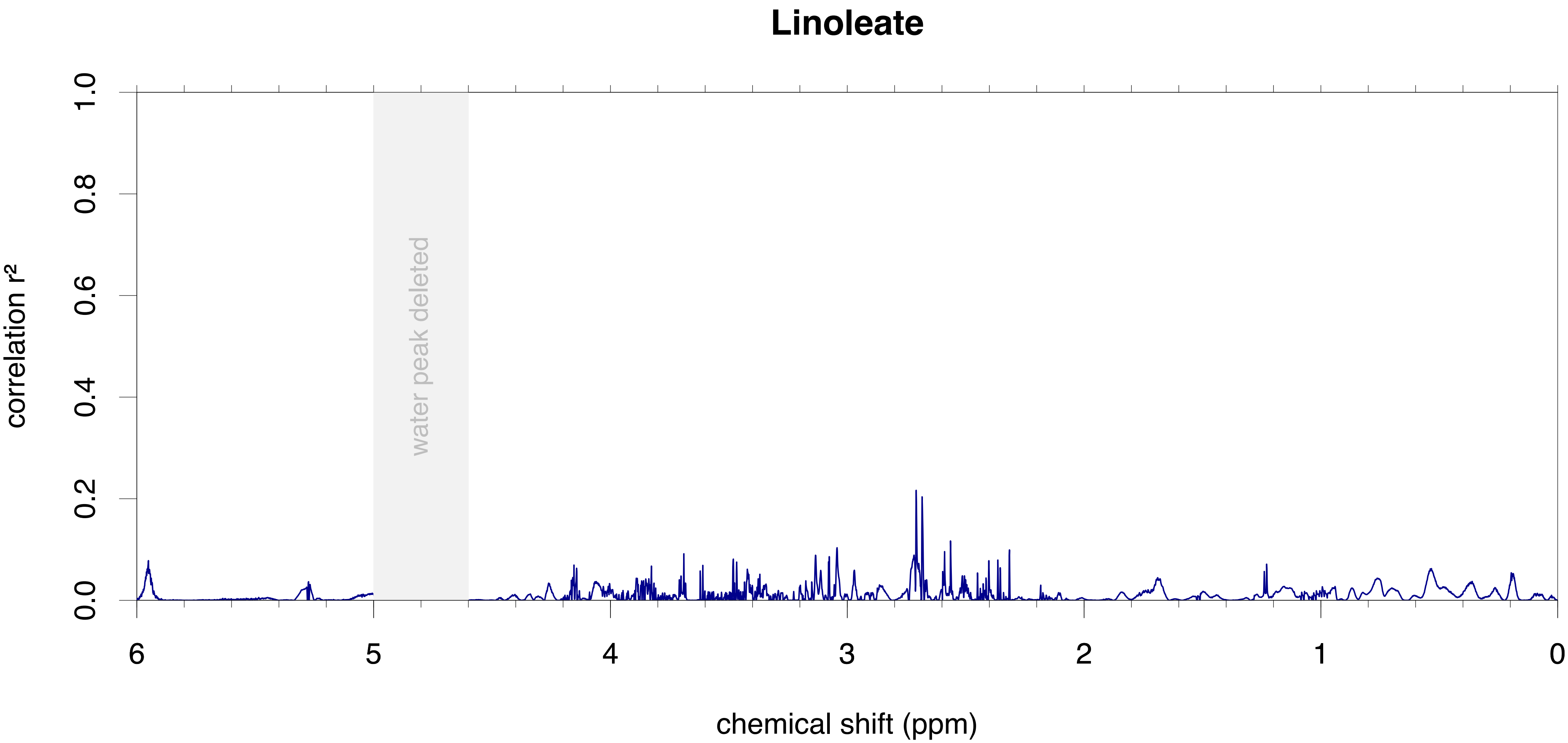

Linoleate
correlation r²
1.0
0.8
0.6
0.4
0.2
0.0
water peak deleted
6
5
4
3
2
1
0
chemical shift (ppm)

Palmitate

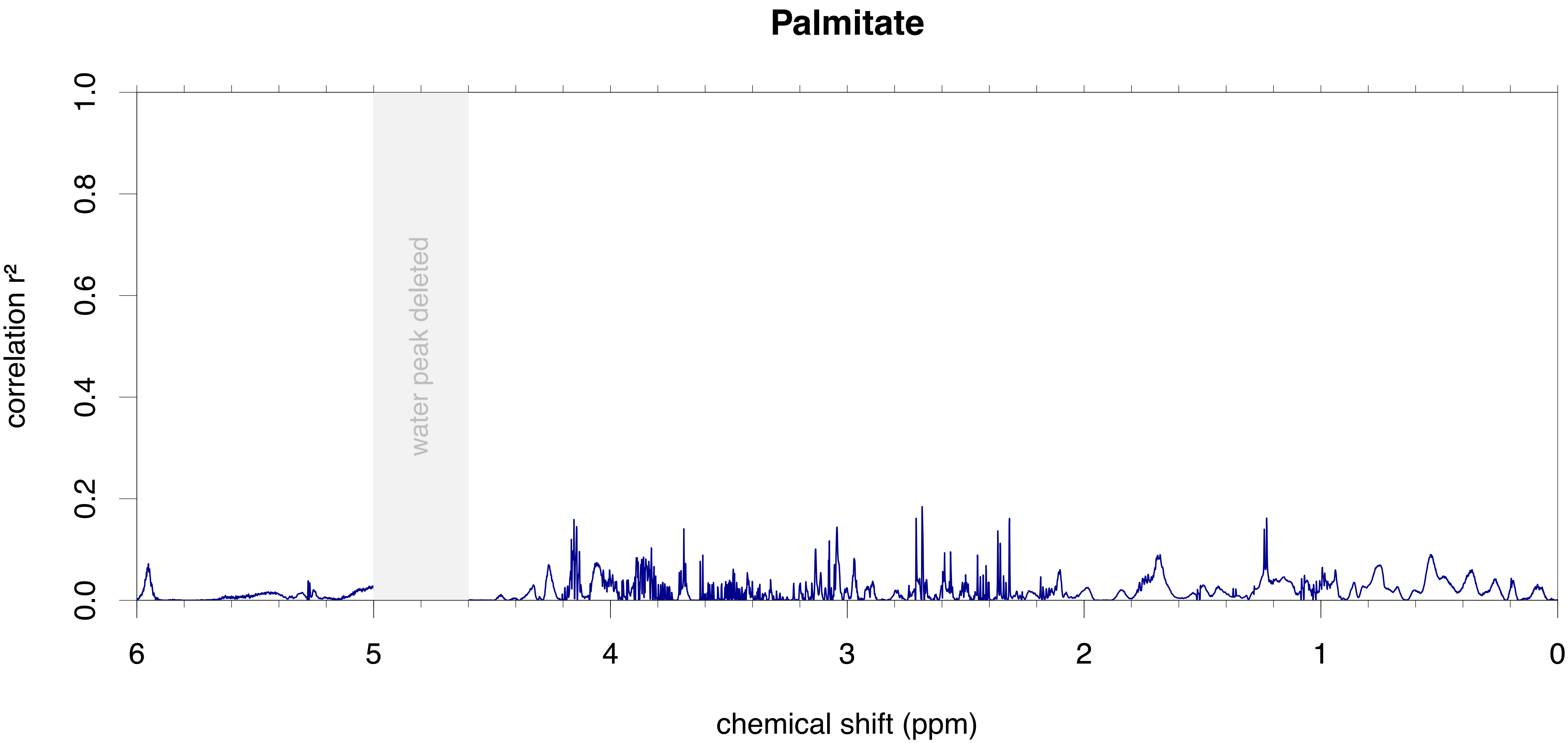

Palmitate
correlation r²
1.0
0.8
0.6
0.4
0.2
0.0
water peak deleted
6
5
4
3
2
1
0
chemical shift (ppm)

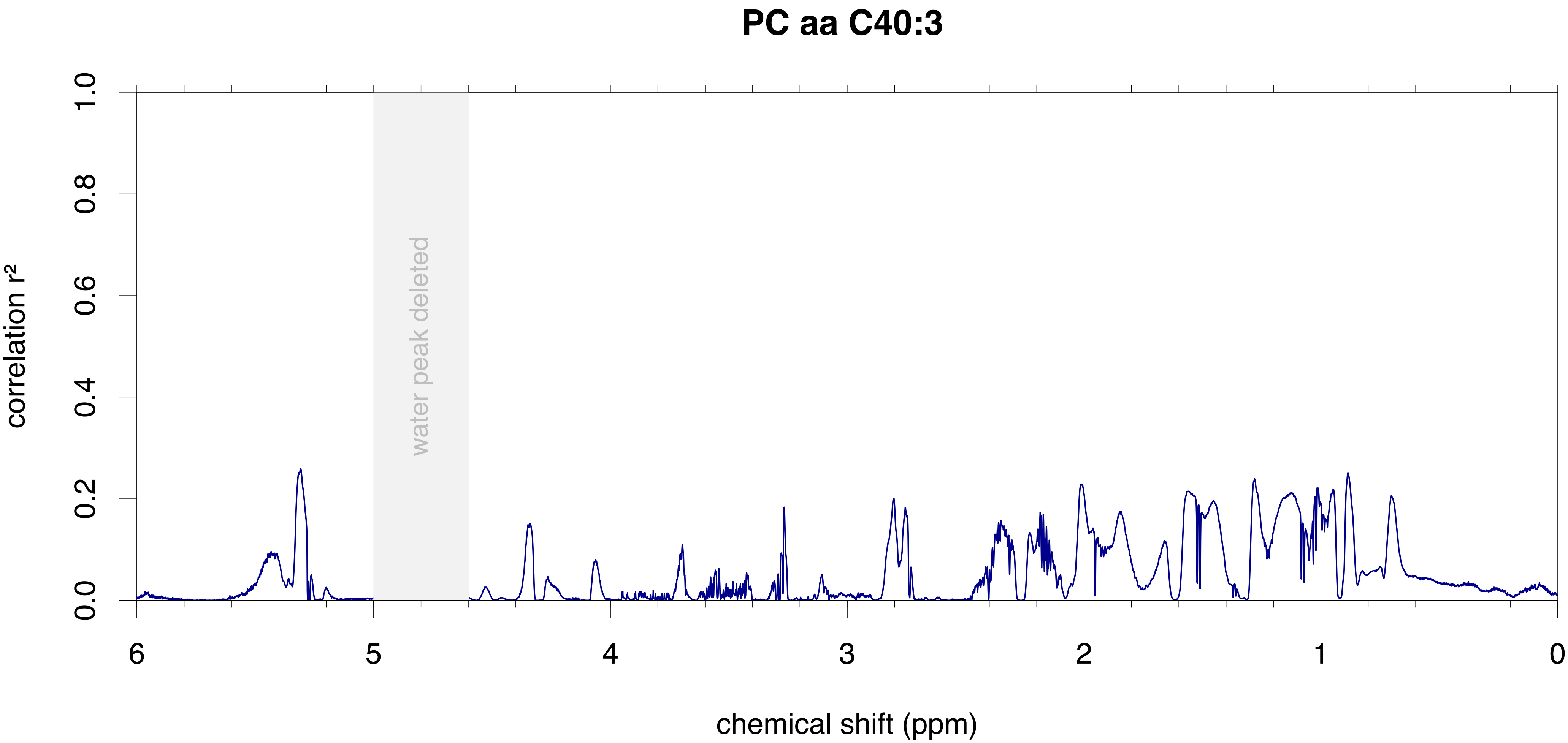

PC aa C40:3
correlation r²
1.0
0.8
0.6
0.4
0.2
0.0
water peak deleted
6
5
4
3
2
1
0
chemical shift (ppm)

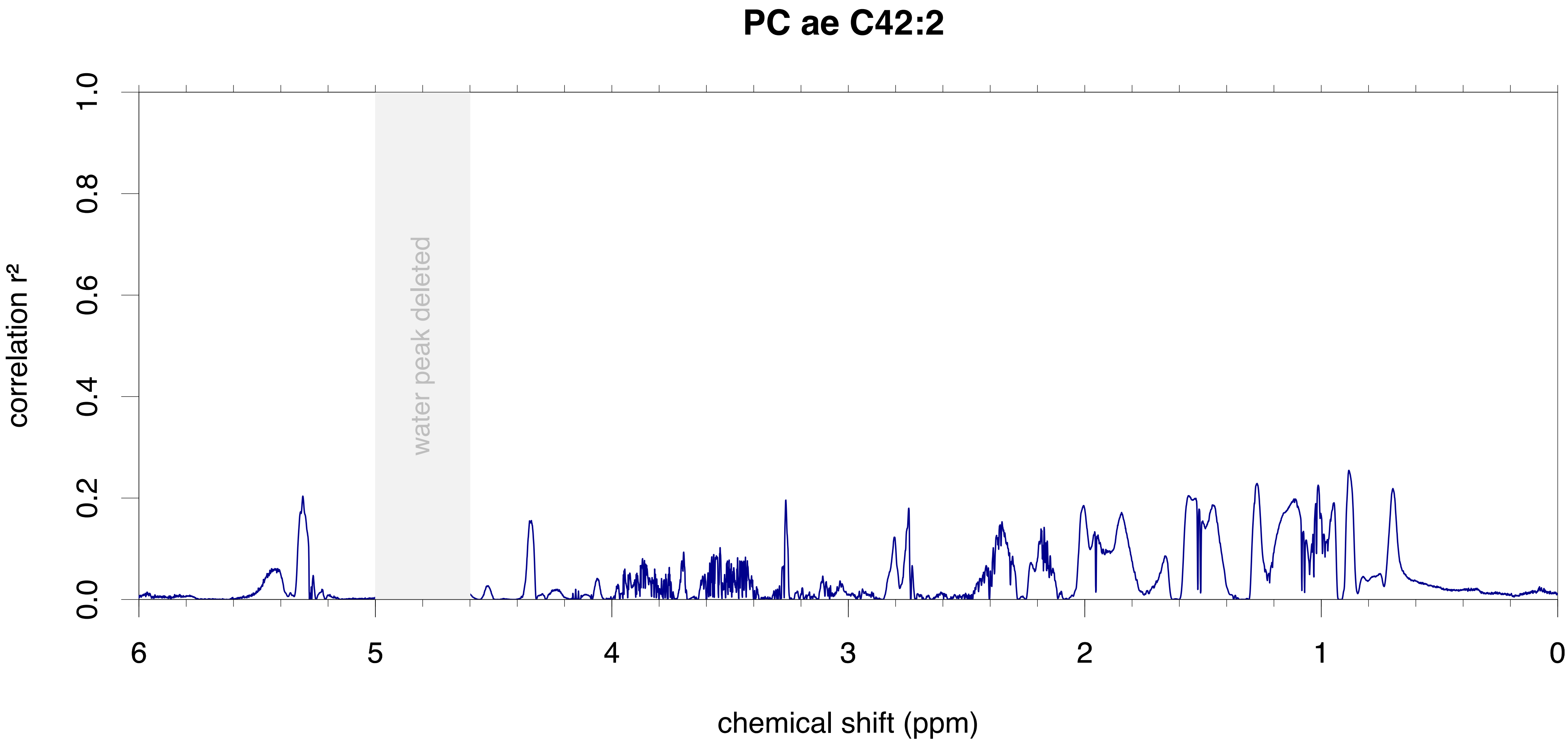
PC ae C42:2
correlation r²
chemical shift (ppm)
water peak deleted
0.0
0.2
0.4
0.6
0.8
1.0
6
5
4
3
2
1
0

PC ae C36:5

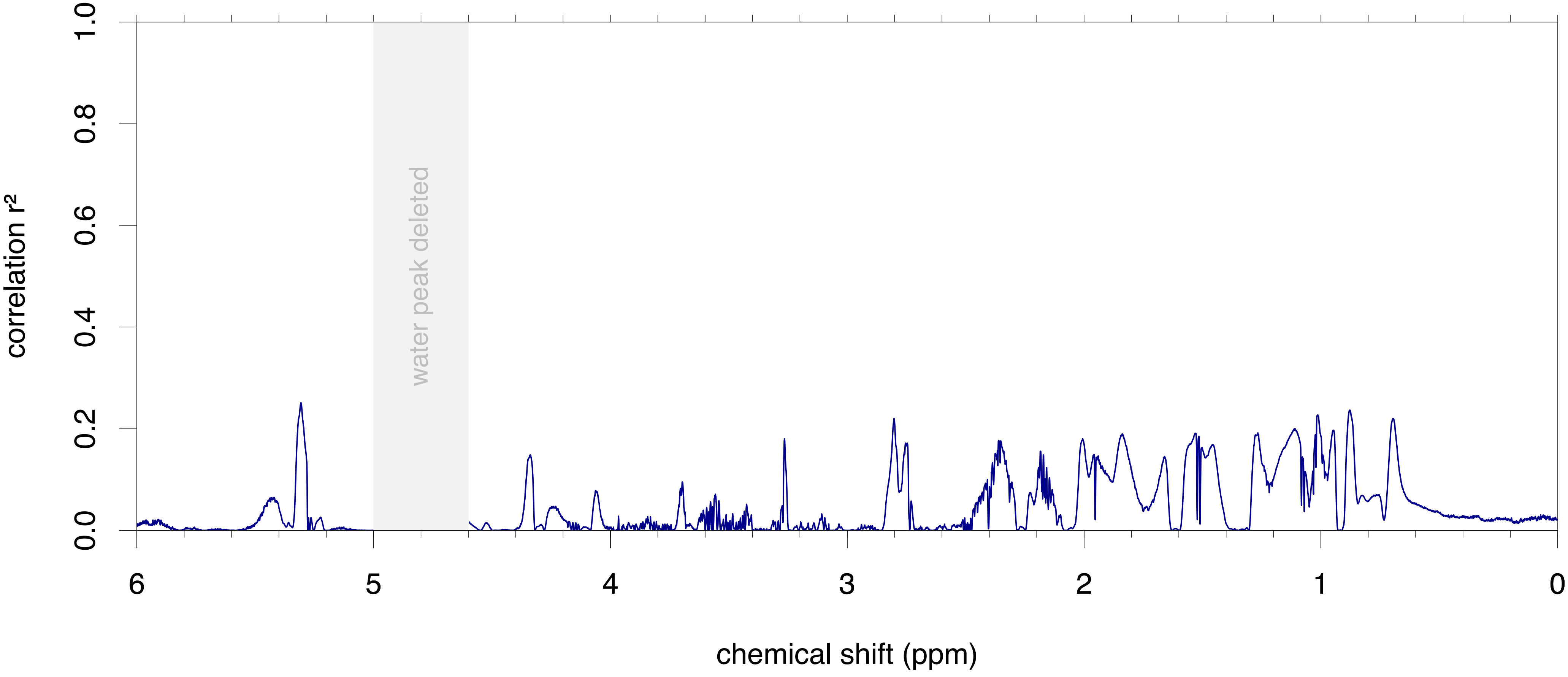

correlation r²
1.0
0.8
0.6
0.4
0.2
0.0
water peak deleted
6
5
4
3
2
1
0
chemical shift (ppm)

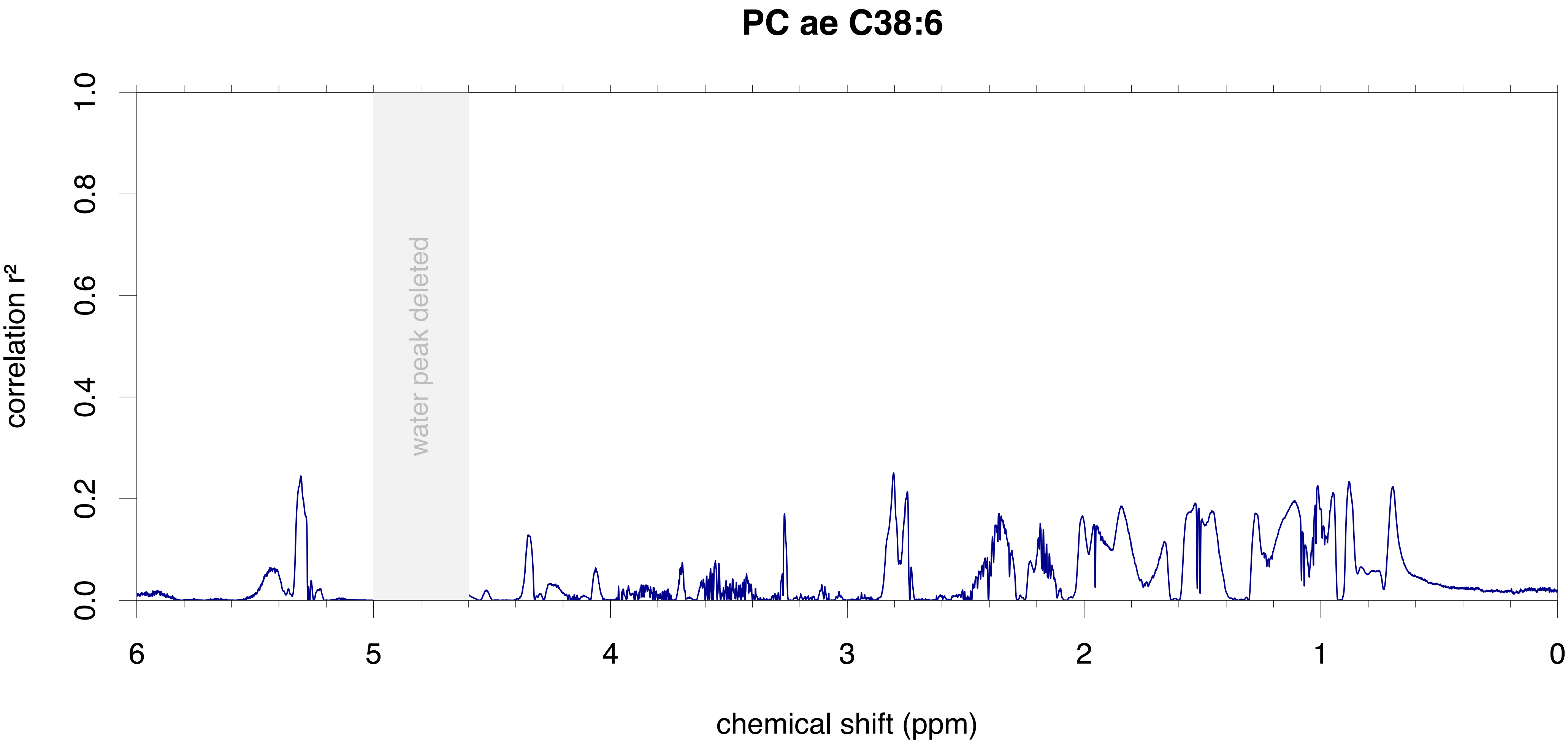

PC ae C38:6
correlation r²
water peak deleted
0.0
0.2
0.4
0.6
0.8
1.0
6
5
4
3
2
1
0
chemical shift (ppm)

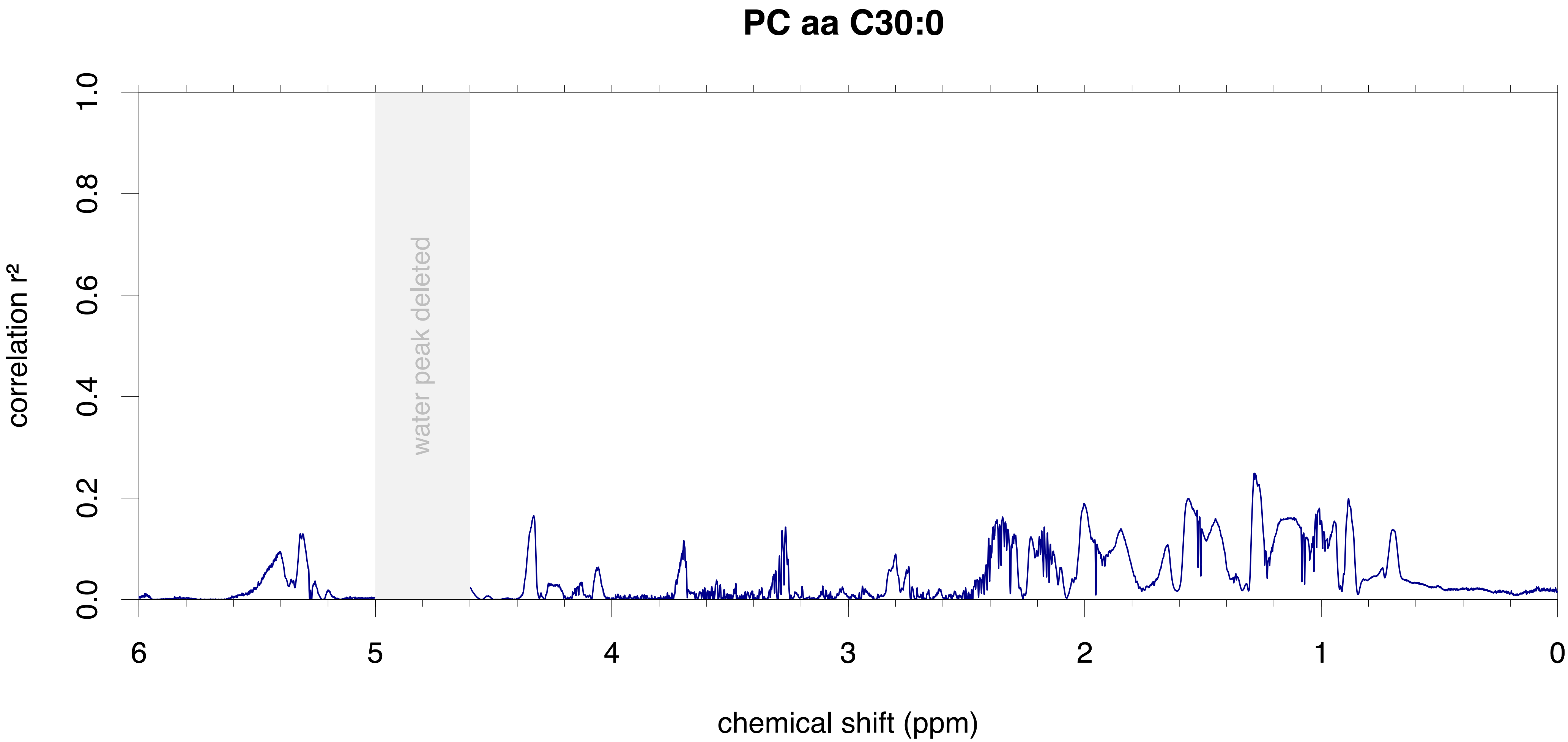

PC aa C30:0
correlation r²
1.0
0.8
0.6
0.4
0.2
0.0
water peak deleted
6
5
4
3
2
1
0
chemical shift (ppm)

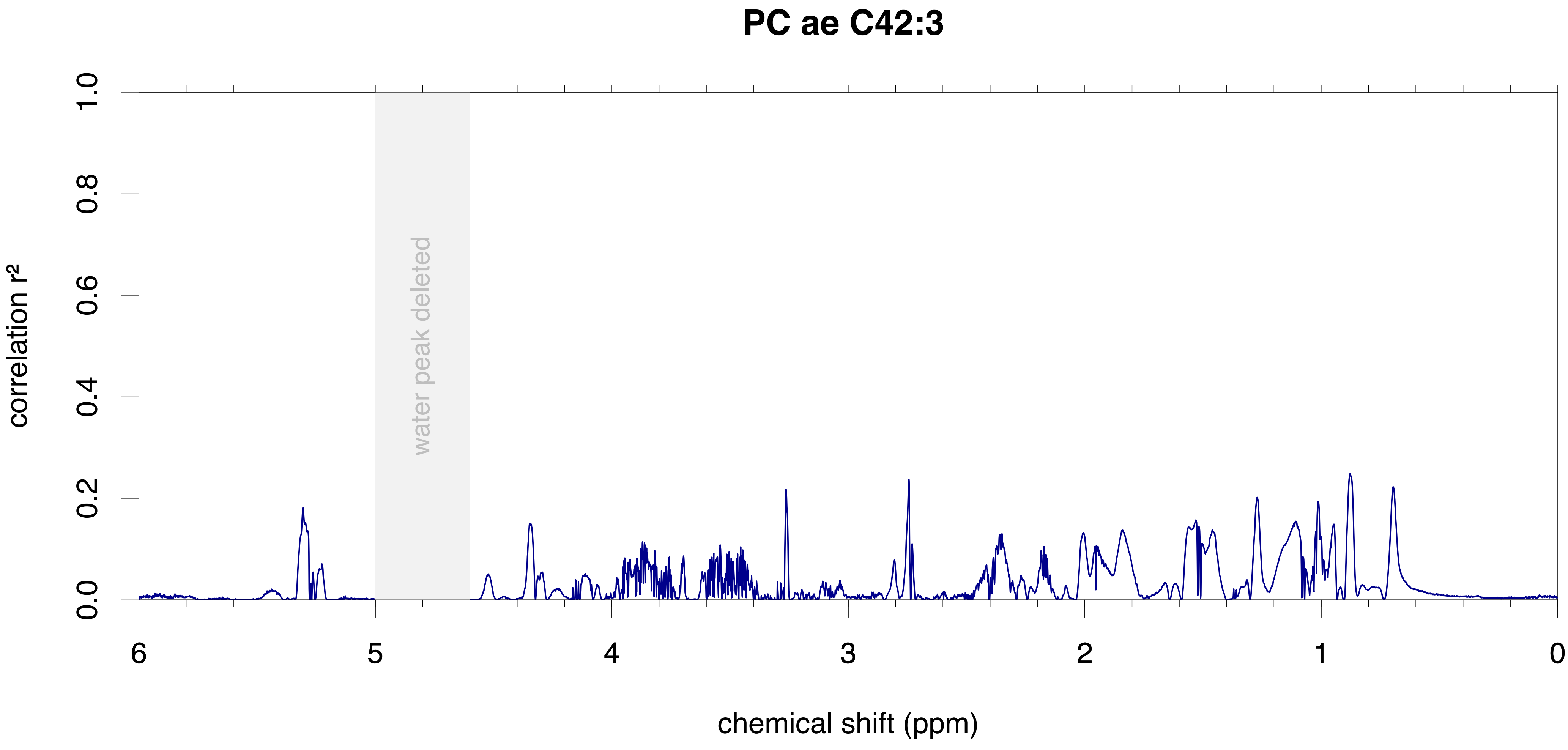

PC ae C42:3
correlation r²
1.0
0.8
0.6
0.4
0.2
0.0
water peak deleted
6
5
4
3
2
1
0
chemical shift (ppm)

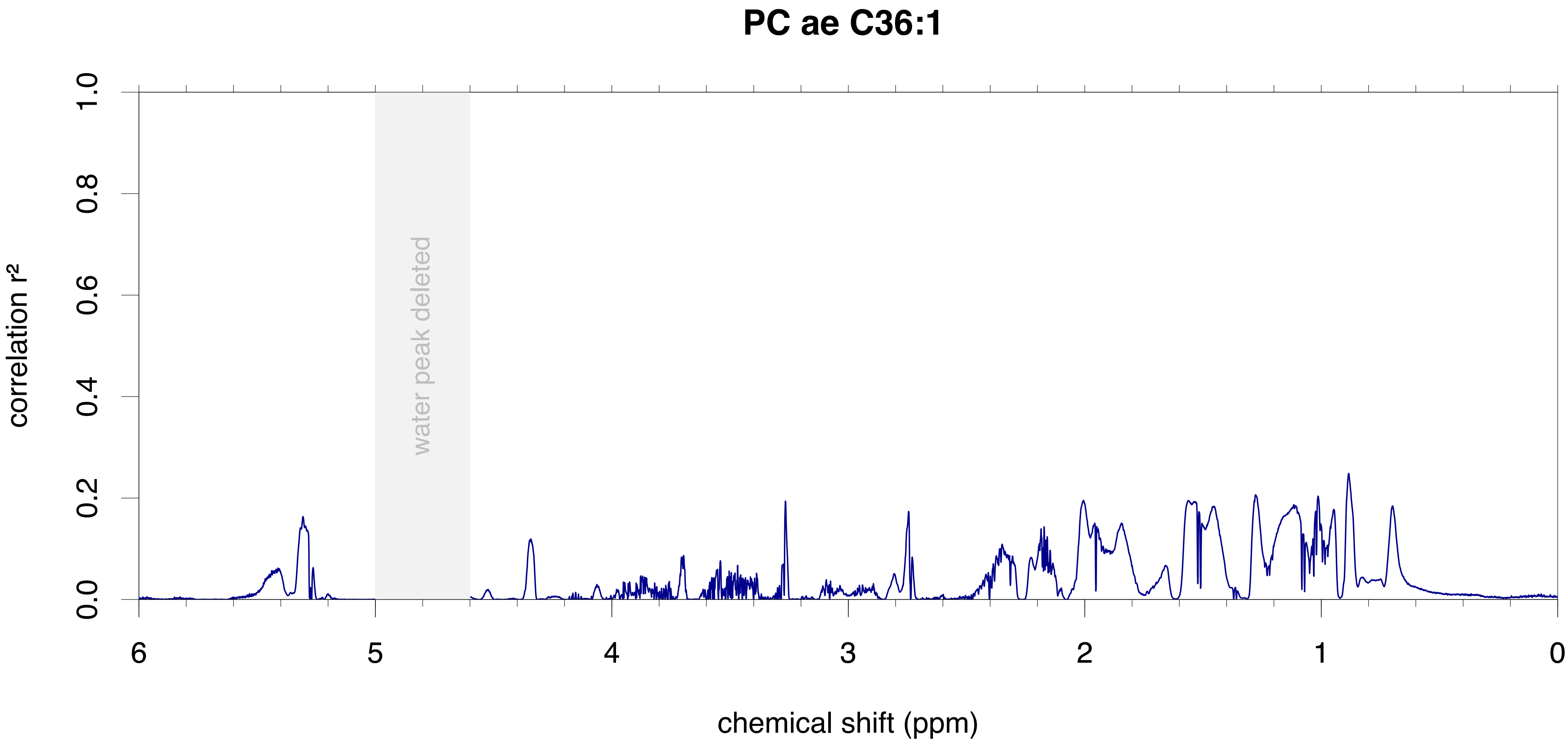
PC ae C36:1
correlation r²
chemical shift (ppm)
water peak deleted
0.0
0.2
0.4
0.6
0.8
1.0
6
5
4
3
2
1
0

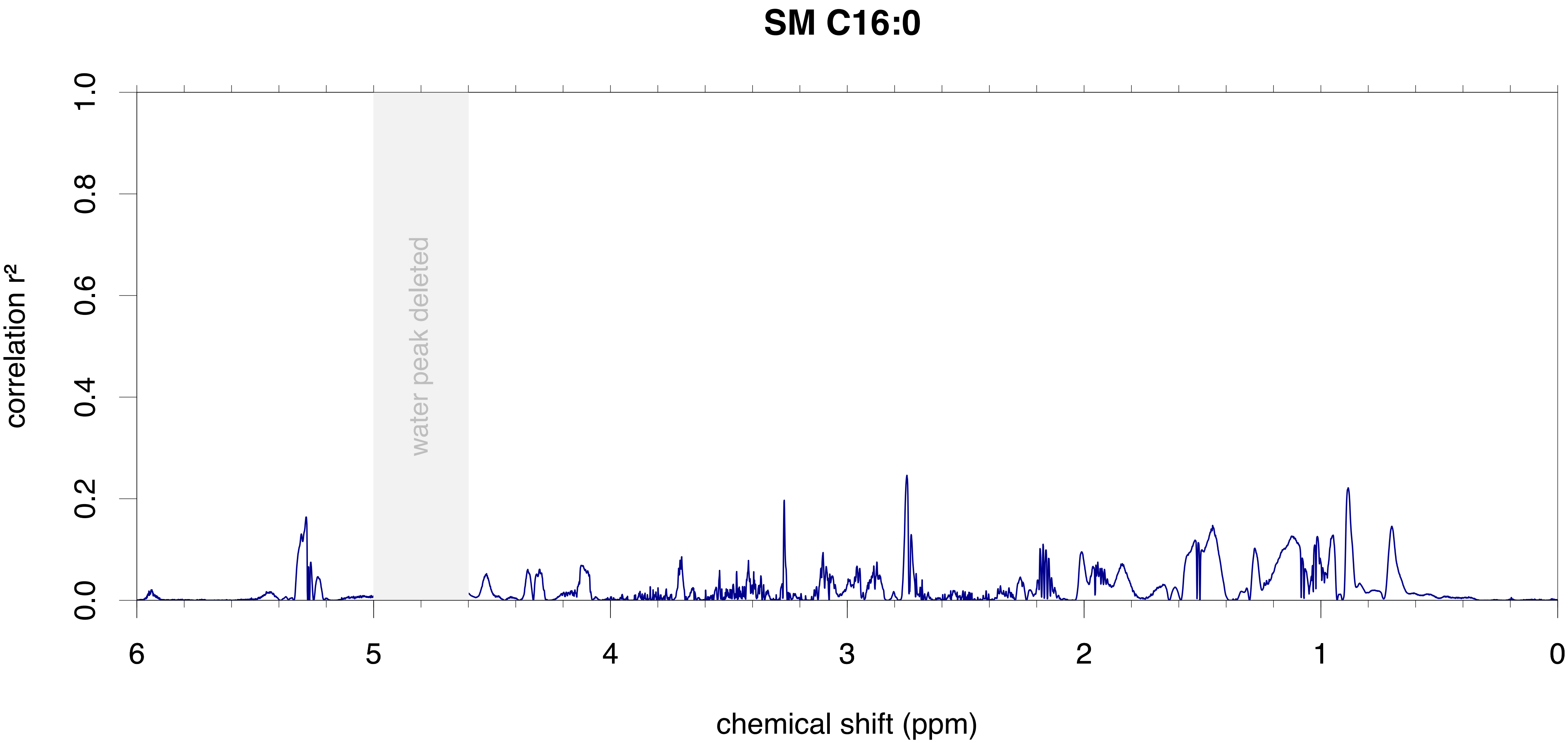
SM C16:0
correlation r²
1.0
0.8
0.6
0.4
0.2
0.0
water peak deleted
6
5
4
3
2
1
0
chemical shift (ppm)

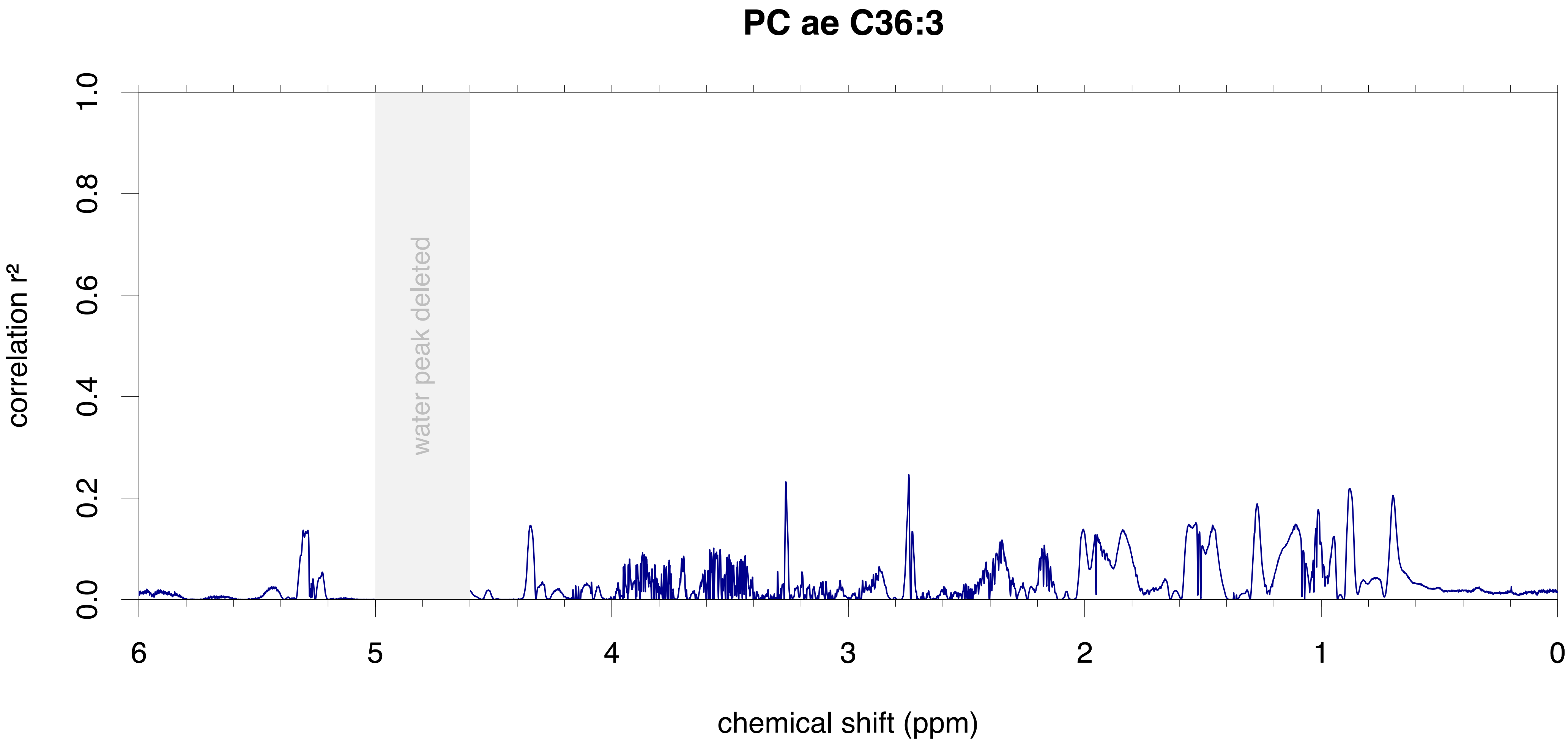
PC ae C36:3
correlation r²
0.0
0.2
0.4
0.6
0.8
1.0
water peak deleted
6
5
4
3
2
1
0
chemical shift (ppm)

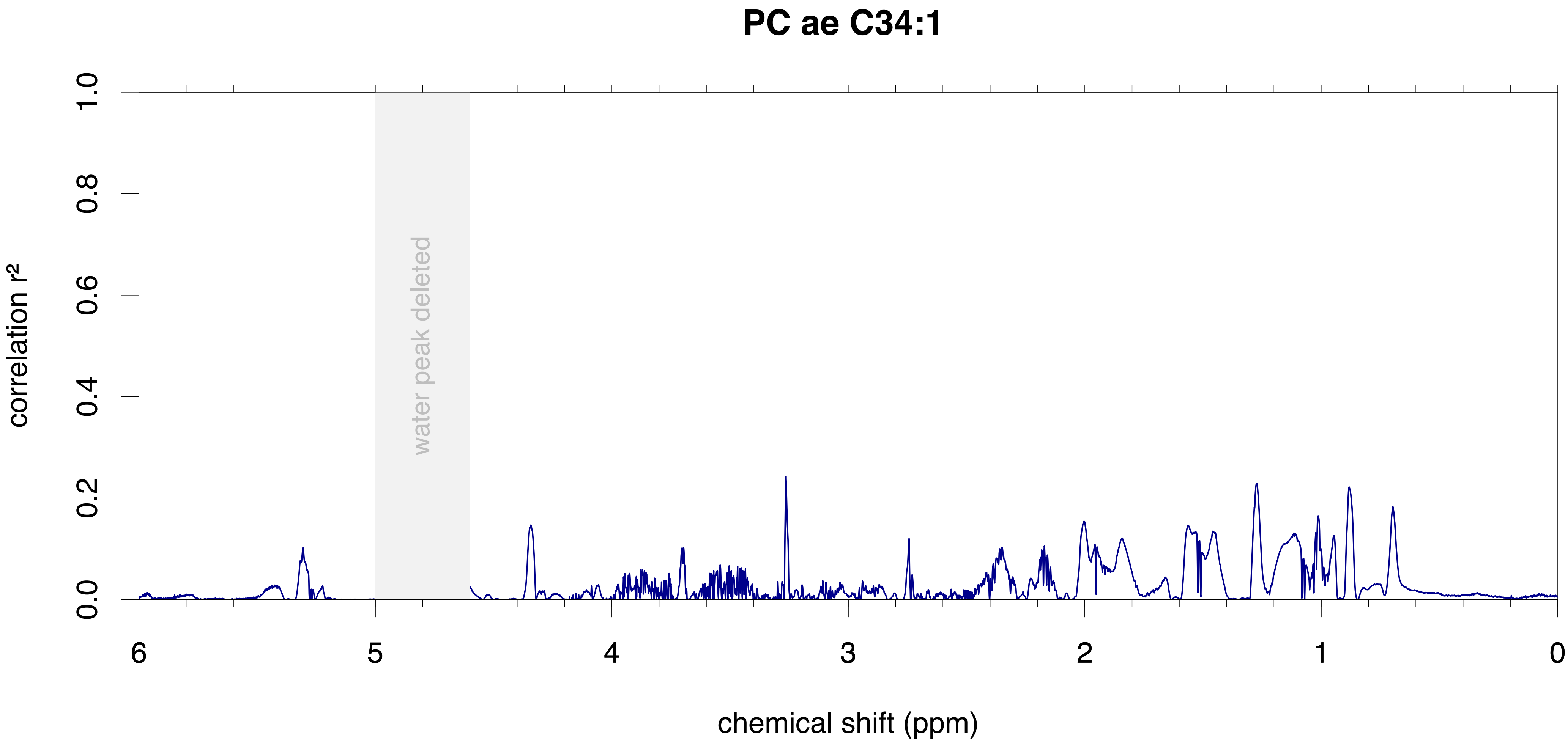
PC ae C34:1
correlation r²
1.0
0.8
0.6
0.4
0.2
0.0
water peak deleted
6
5
4
3
2
1
0
chemical shift (ppm)

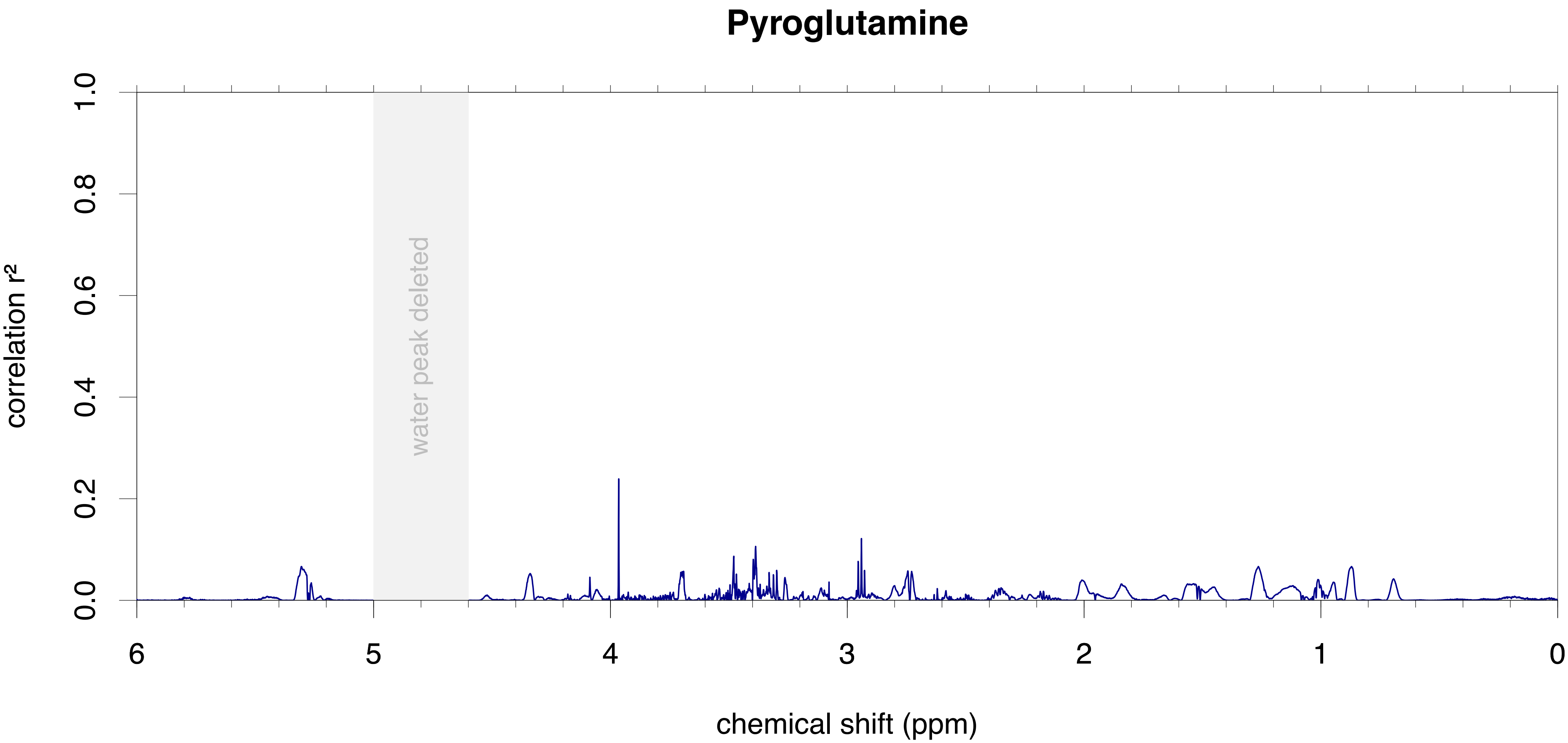

Pyroglutamine
correlation r²
1.0
0.8
0.6
0.4
0.2
0.0
water peak deleted
6
5
4
3
2
1
0
chemical shift (ppm)

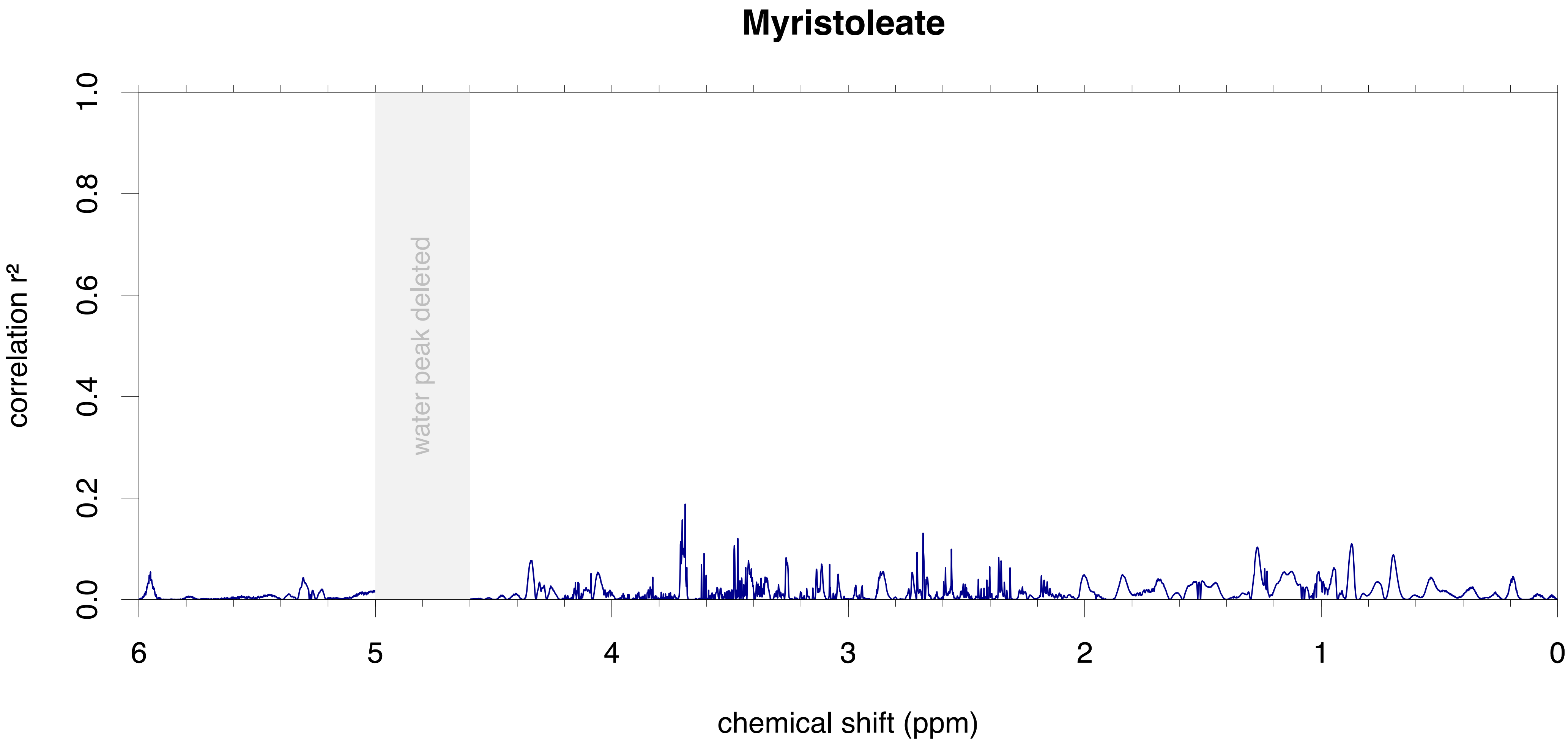

Myristoleate
correlation r²
1.0
0.8
0.6
0.4
0.2
0.0
water peak deleted
6
5
4
3
2
1
0
chemical shift (ppm)

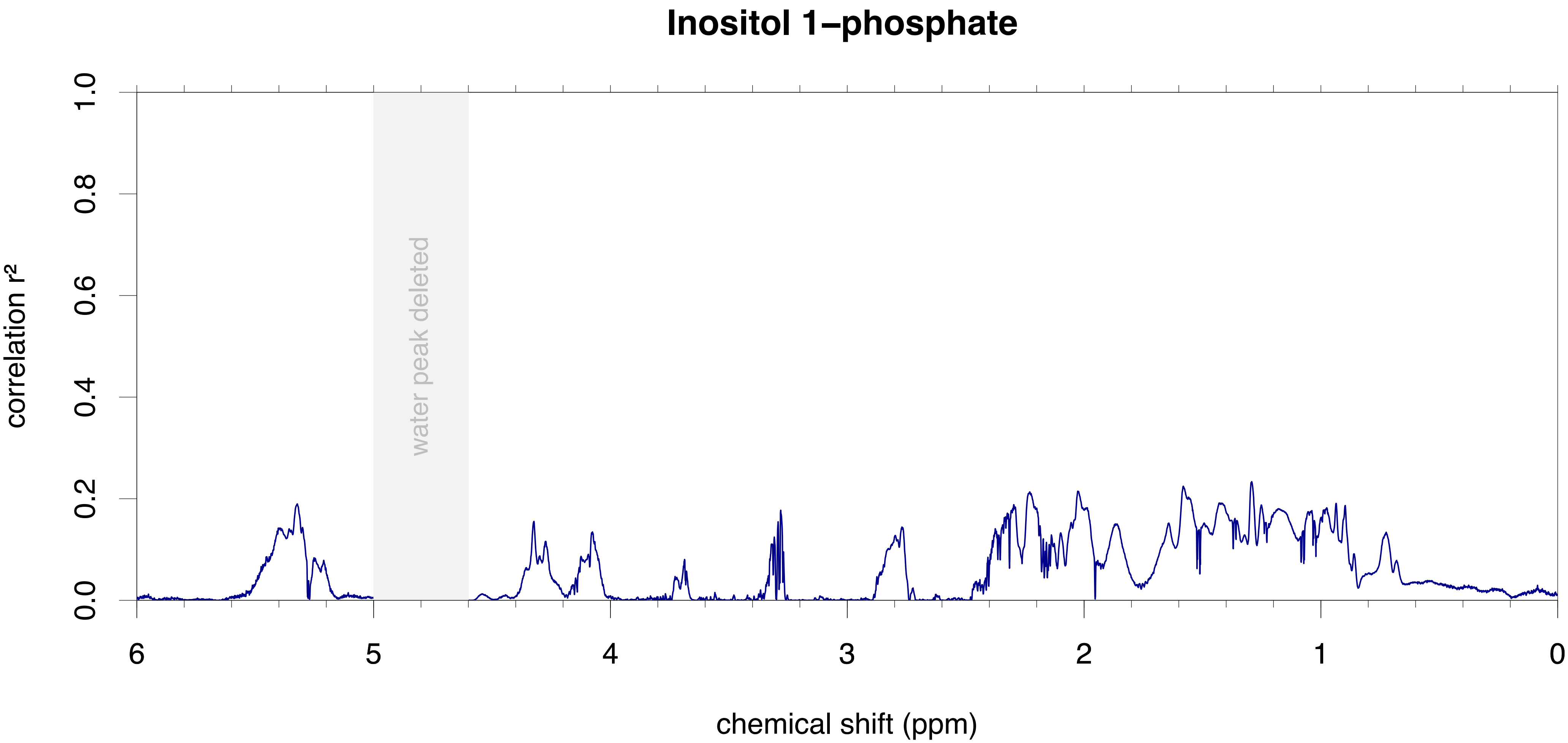
Inositol 1−phosphate
water peak deleted
correlation r²
chemical shift (ppm)
0.0
0.2
0.4
0.6
0.8
1.0
6
5
4
3
2
1
0

1–oleoylglycerol

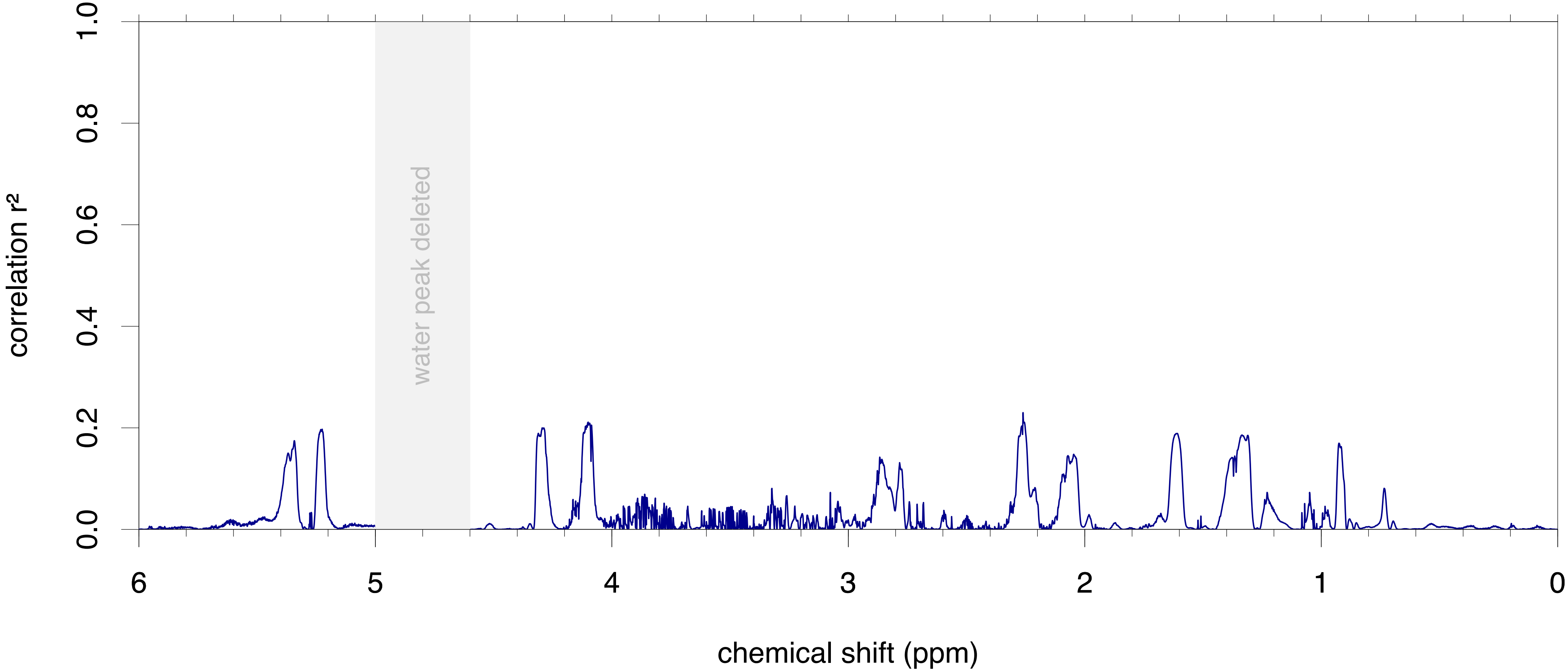

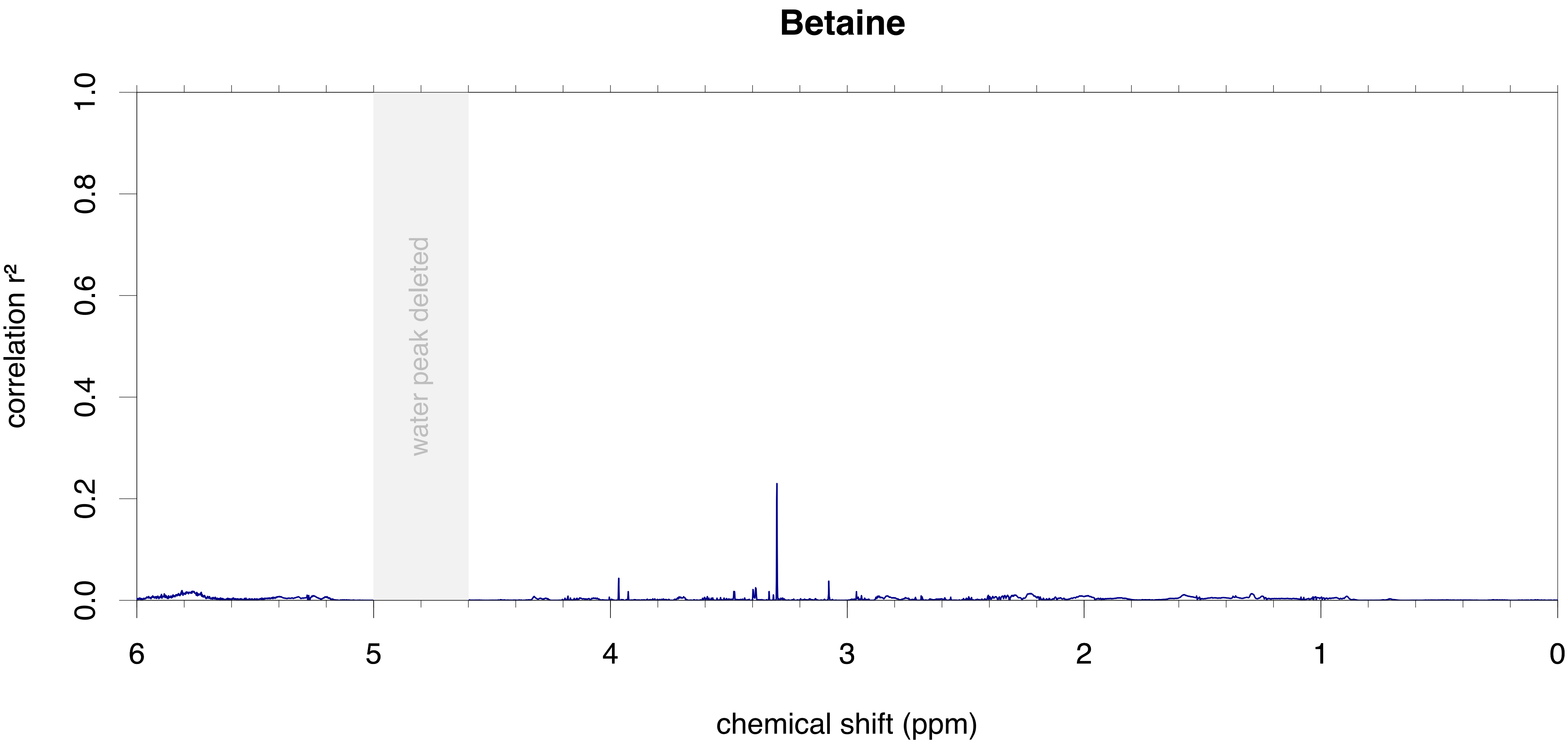

Betaine
correlation r²
1.0
0.8
0.6
0.4
0.2
0.0
water peak deleted
6
5
4
3
2
1
0
chemical shift (ppm)

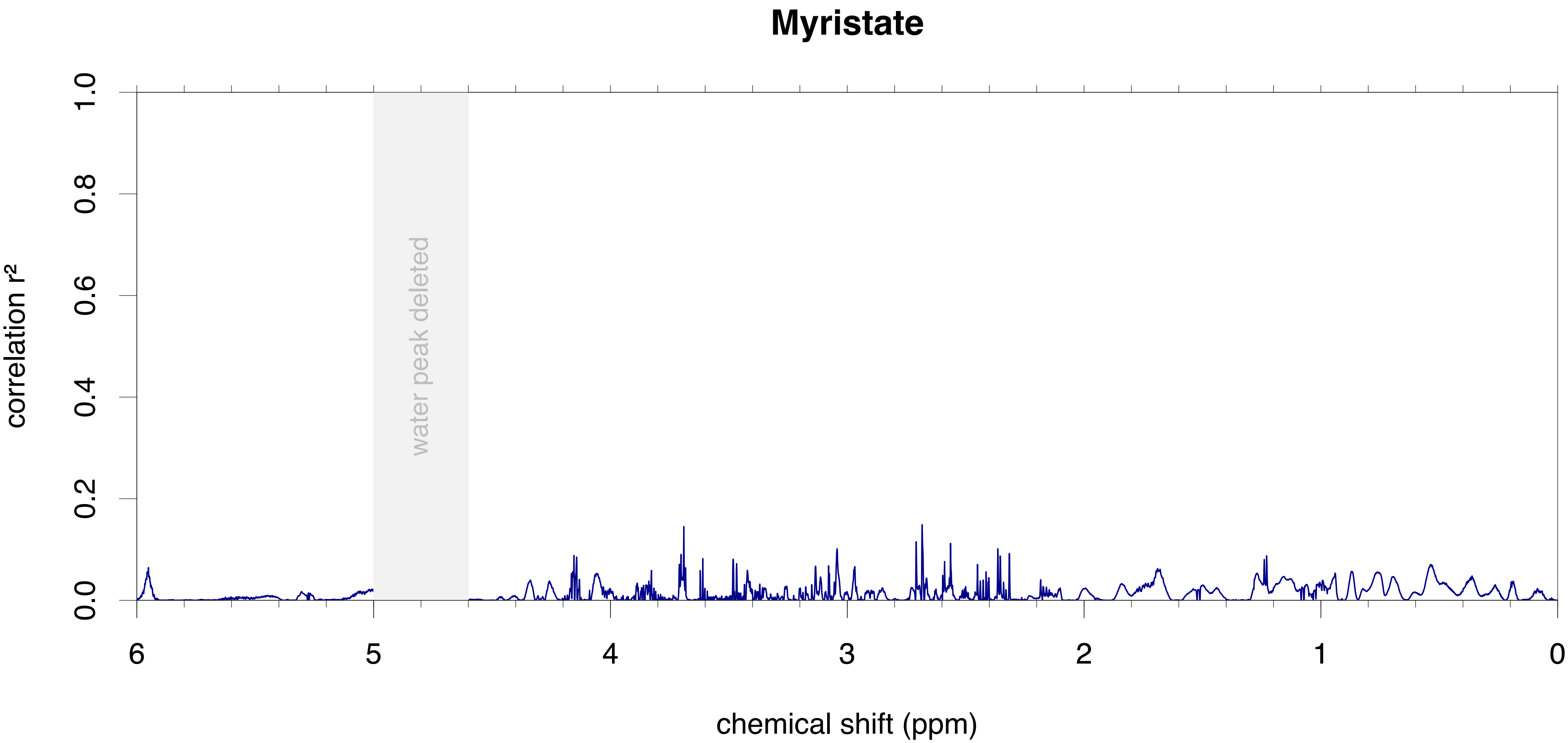
Myristate
correlation r²
1.0
0.8
0.6
0.4
0.2
0.0
water peak deleted
6
5
4
3
2
1
0
chemical shift (ppm)

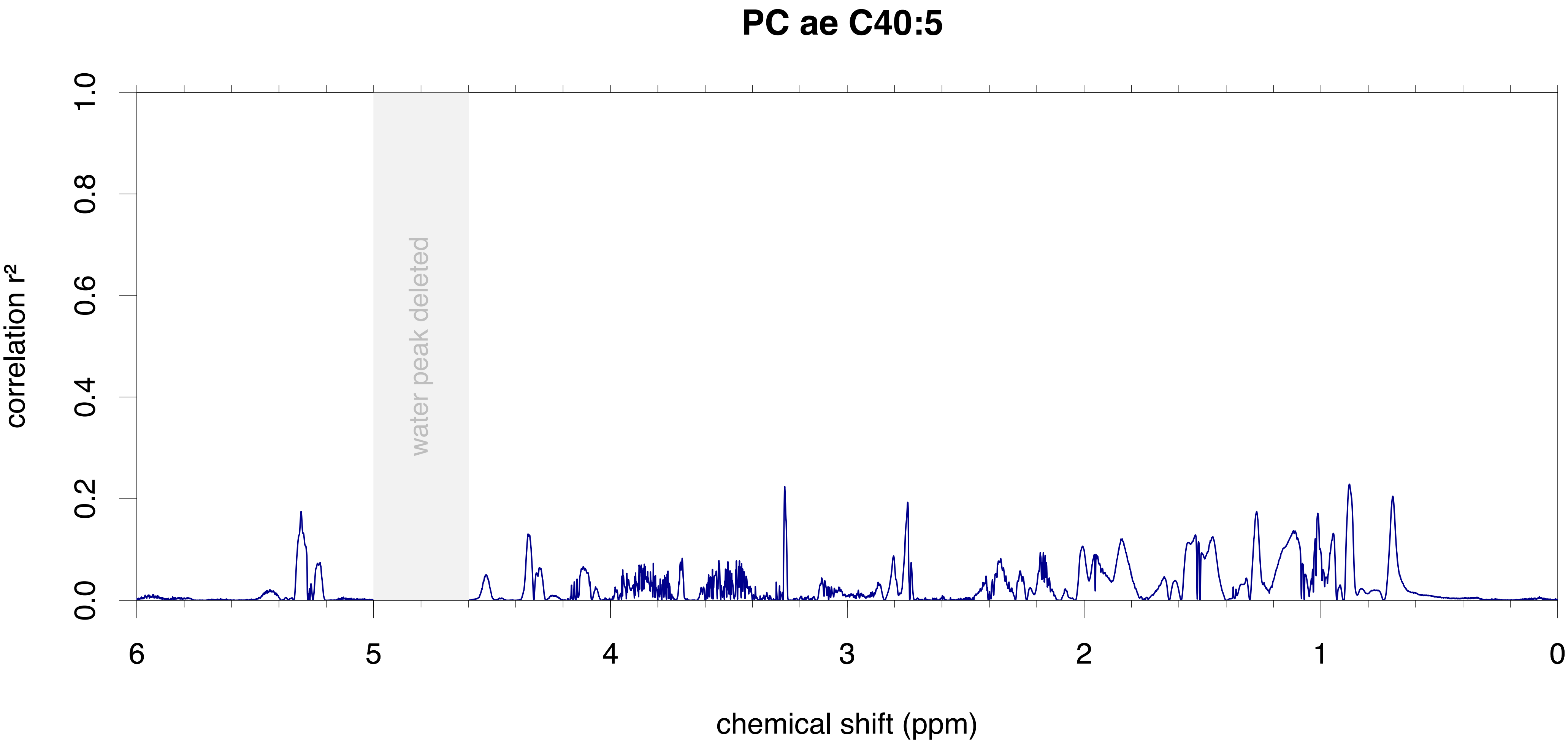
PC ae C40:5
correlation r²
1.0
0.8
0.6
0.4
0.2
0.0
water peak deleted
6
5
4
3
2
1
0
chemical shift (ppm)

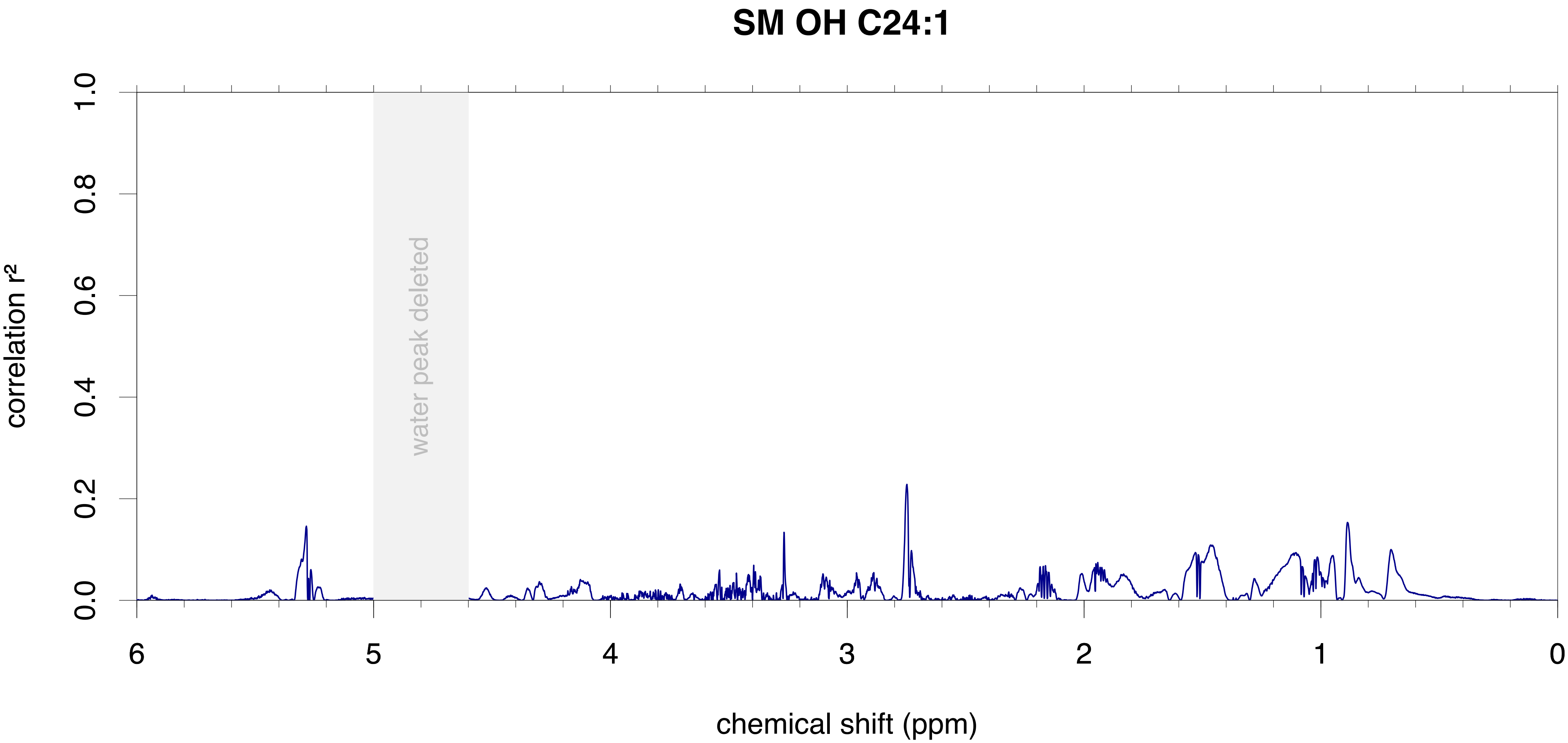

SM OH C24:1
correlation r²
1.0
0.8
0.6
0.4
0.2
0.0
water peak deleted
6
5
4
3
2
1
0
chemical shift (ppm)

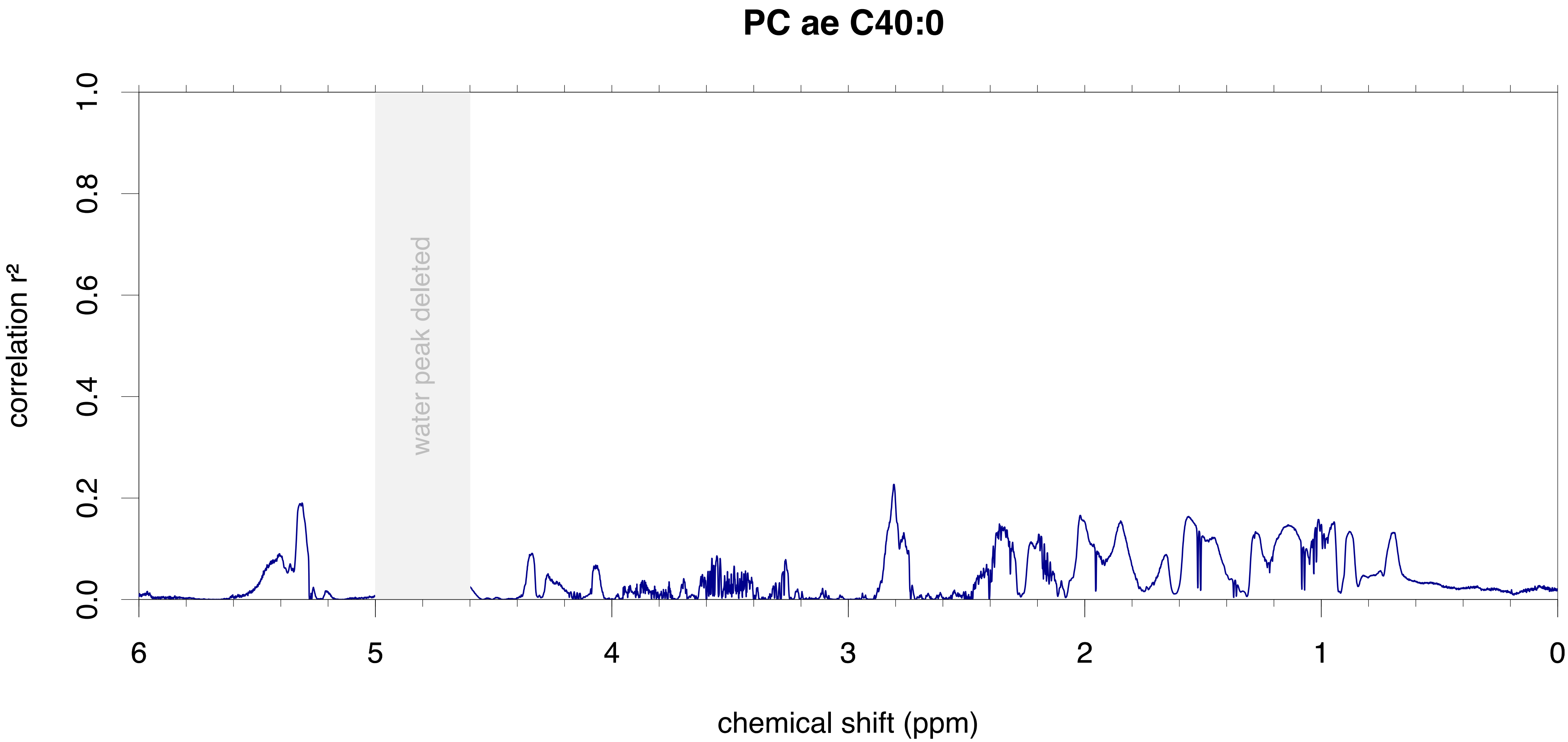
PC ae C40:0
correlation r²
1.0
0.8
0.6
0.4
0.2
0.0
water peak deleted
6
5
4
3
2
1
0
chemical shift (ppm)

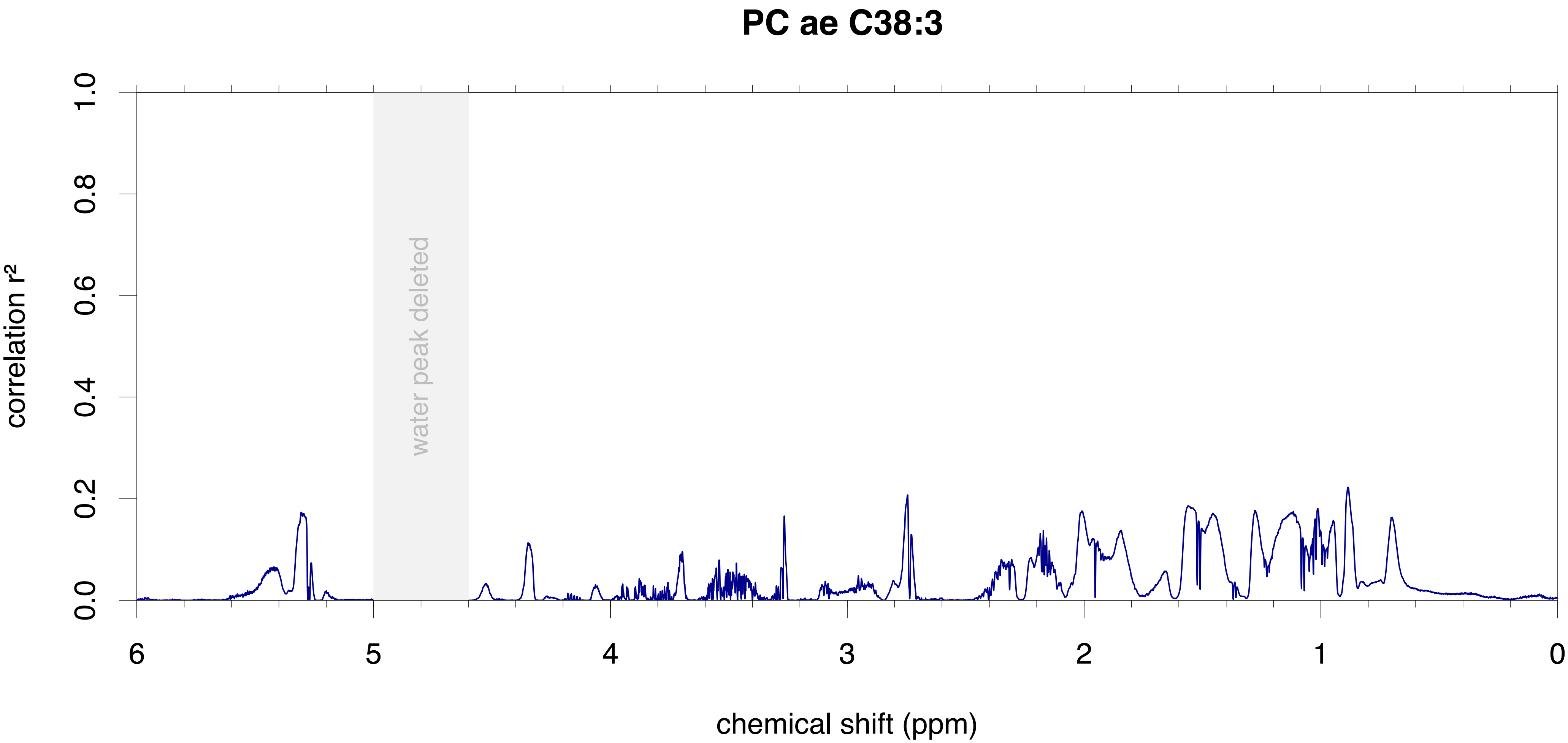

PC ae C38:3
1.0
0.8
0.6
0.4
0.2
0.0
correlation r²
water peak deleted
6
5
4
3
2
1
0
chemical shift (ppm)

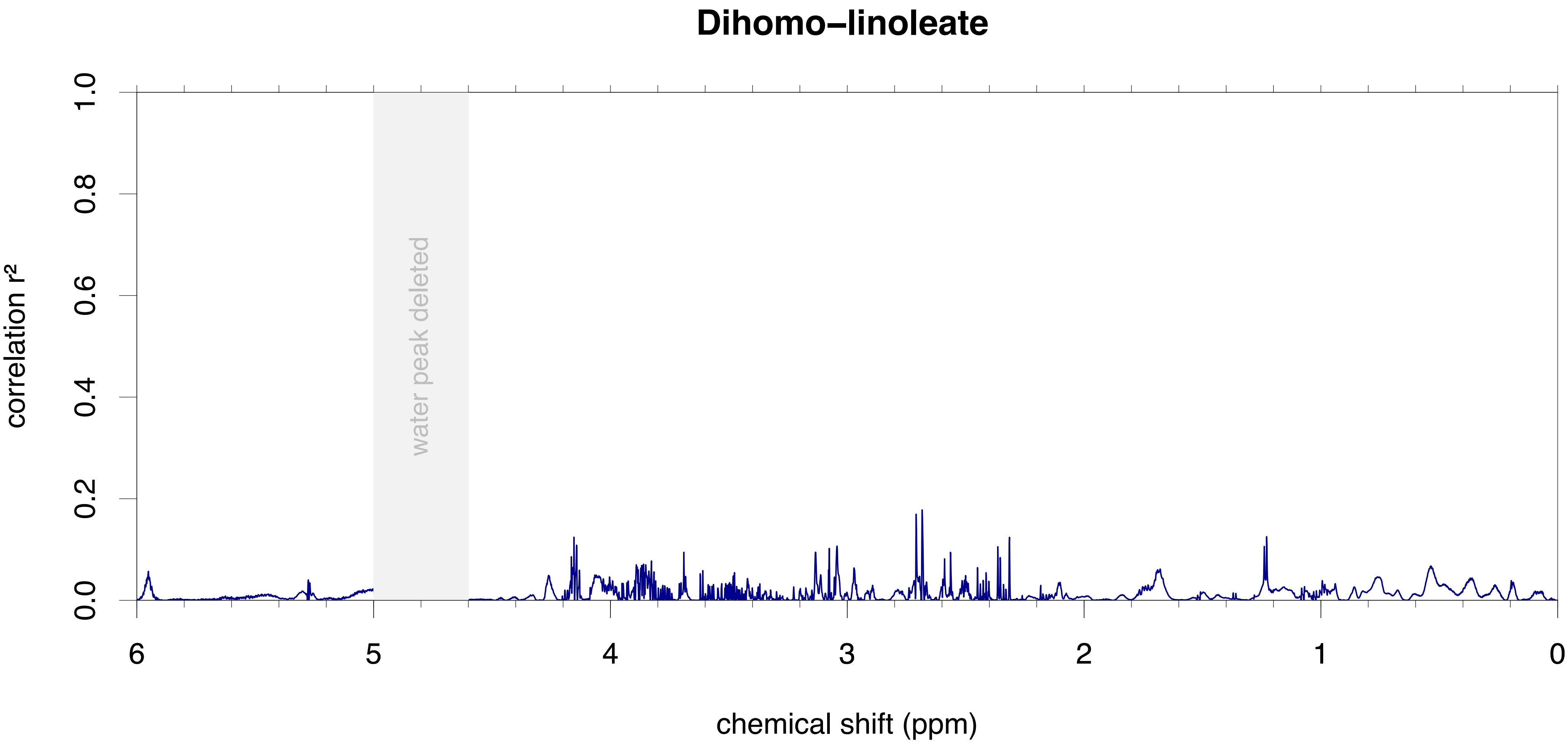

Dihomo–linoleate
correlation r²
1.0
0.8
0.6
0.4
0.2
0.0
water peak deleted
6
5
4
3
2
1
0
chemical shift (ppm)

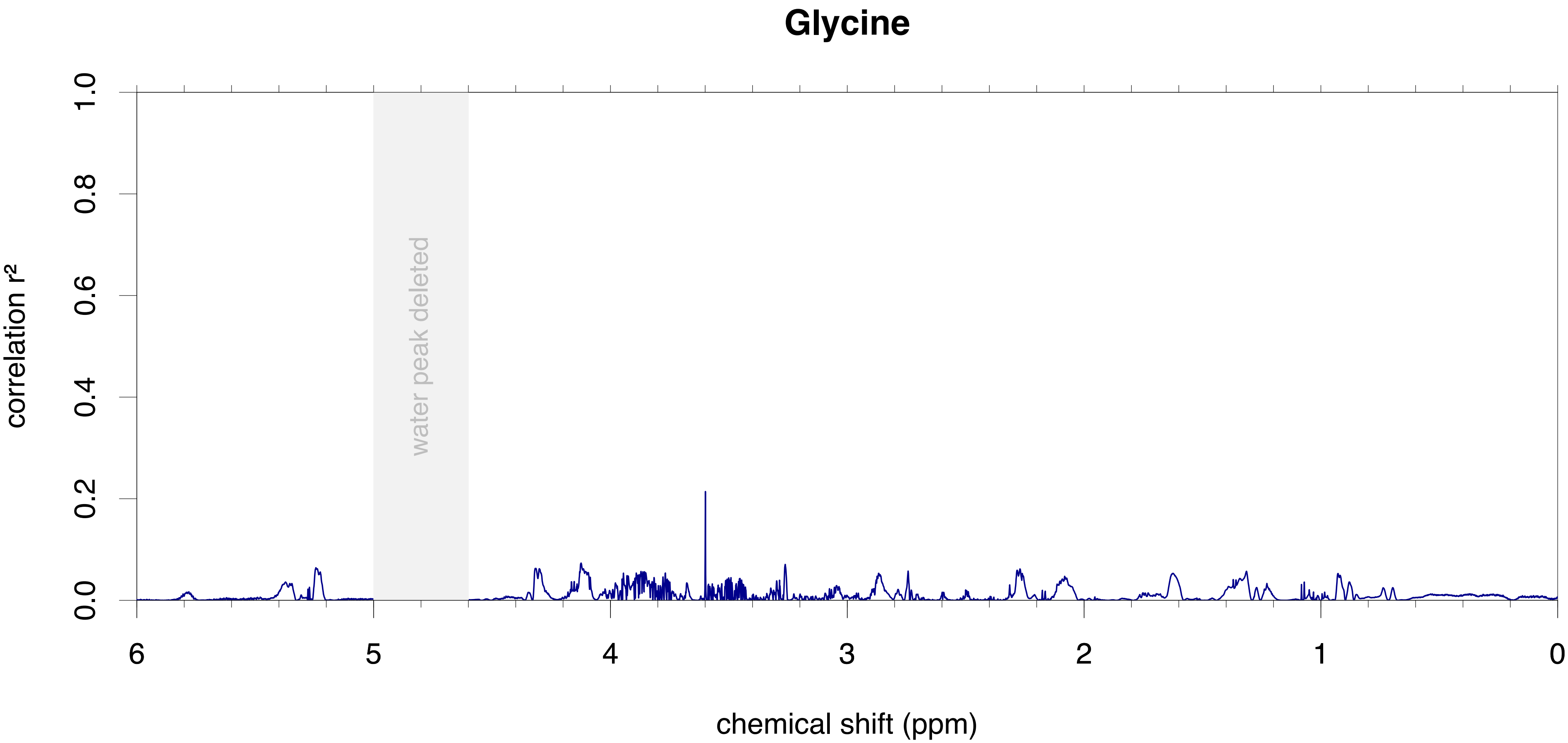

Glycine
correlation r²
1.0
0.8
0.6
0.4
0.2
0.0
water peak deleted
6
5
4
3
2
1
0
chemical shift (ppm)

Glycine (HMDB)

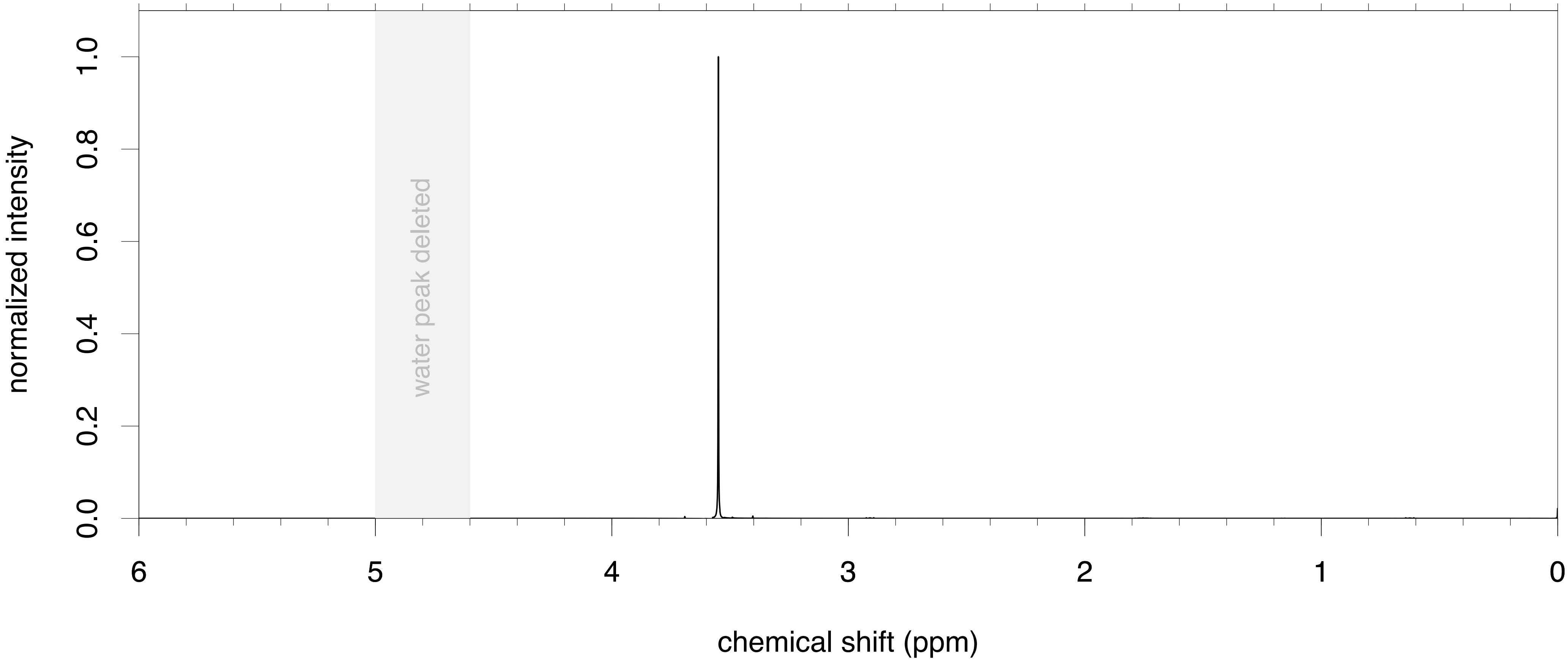

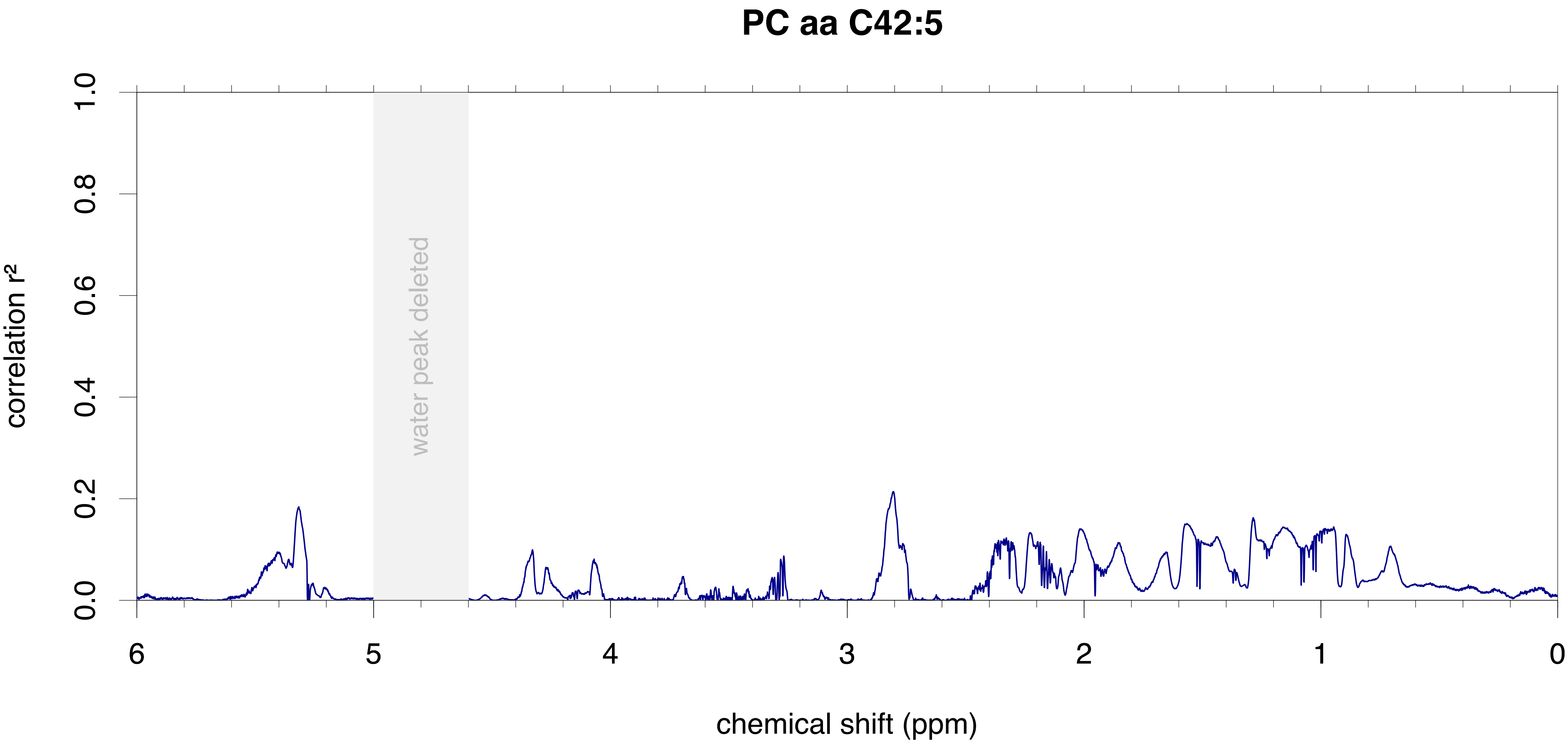
PC aa C42:5
correlation r²
1.0
0.8
0.6
0.4
0.2
0.0
water peak deleted
6
5
4
3
2
1
0
chemical shift (ppm)

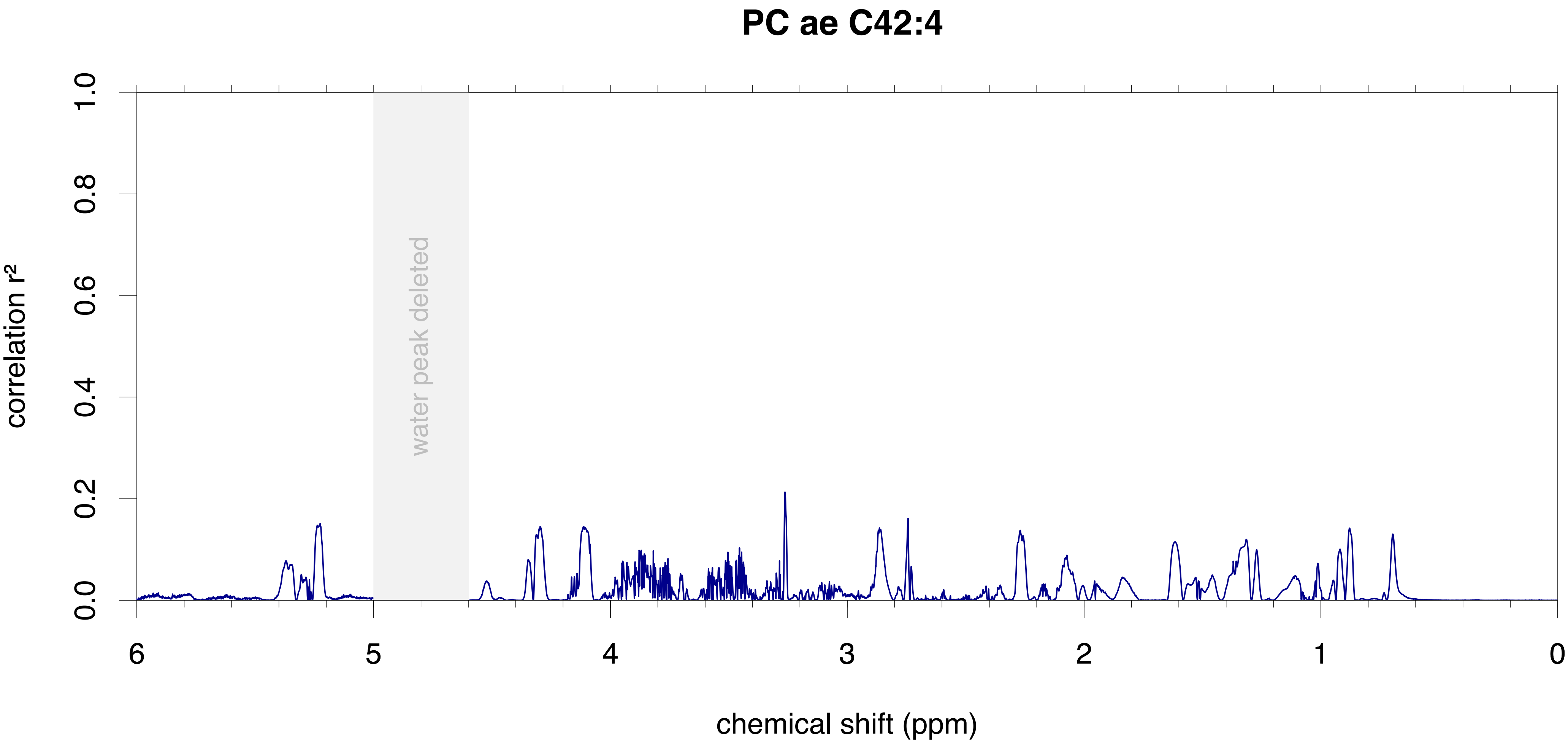

PC ae C42:4
correlation r²
1.0
0.8
0.6
0.4
0.2
0.0
water peak deleted
6
5
4
3
2
1
0
chemical shift (ppm)

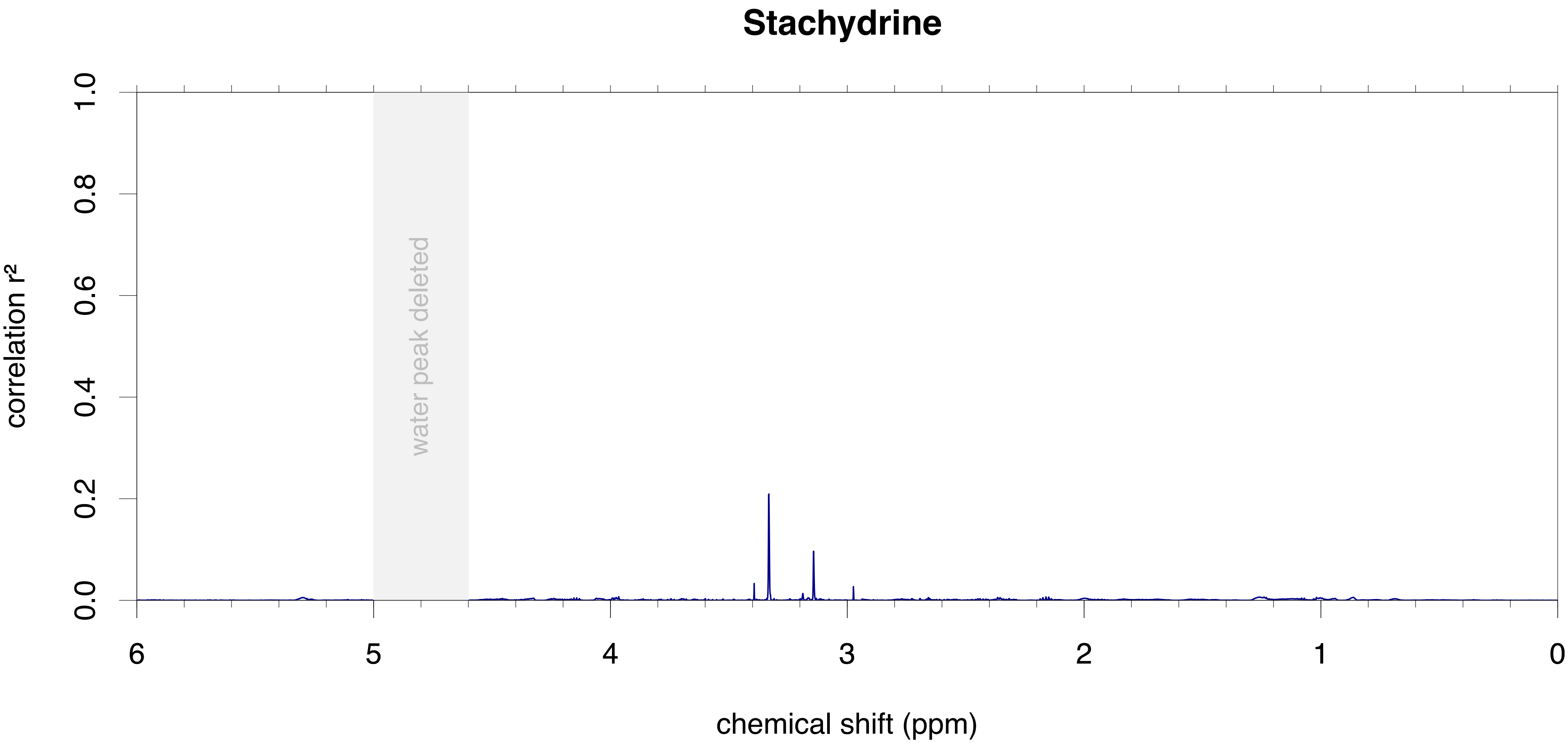

Stachydrine
correlation r²
1.0
0.8
0.6
0.4
0.2
0.0
water peak deleted
6
5
4
3
2
1
0
chemical shift (ppm)

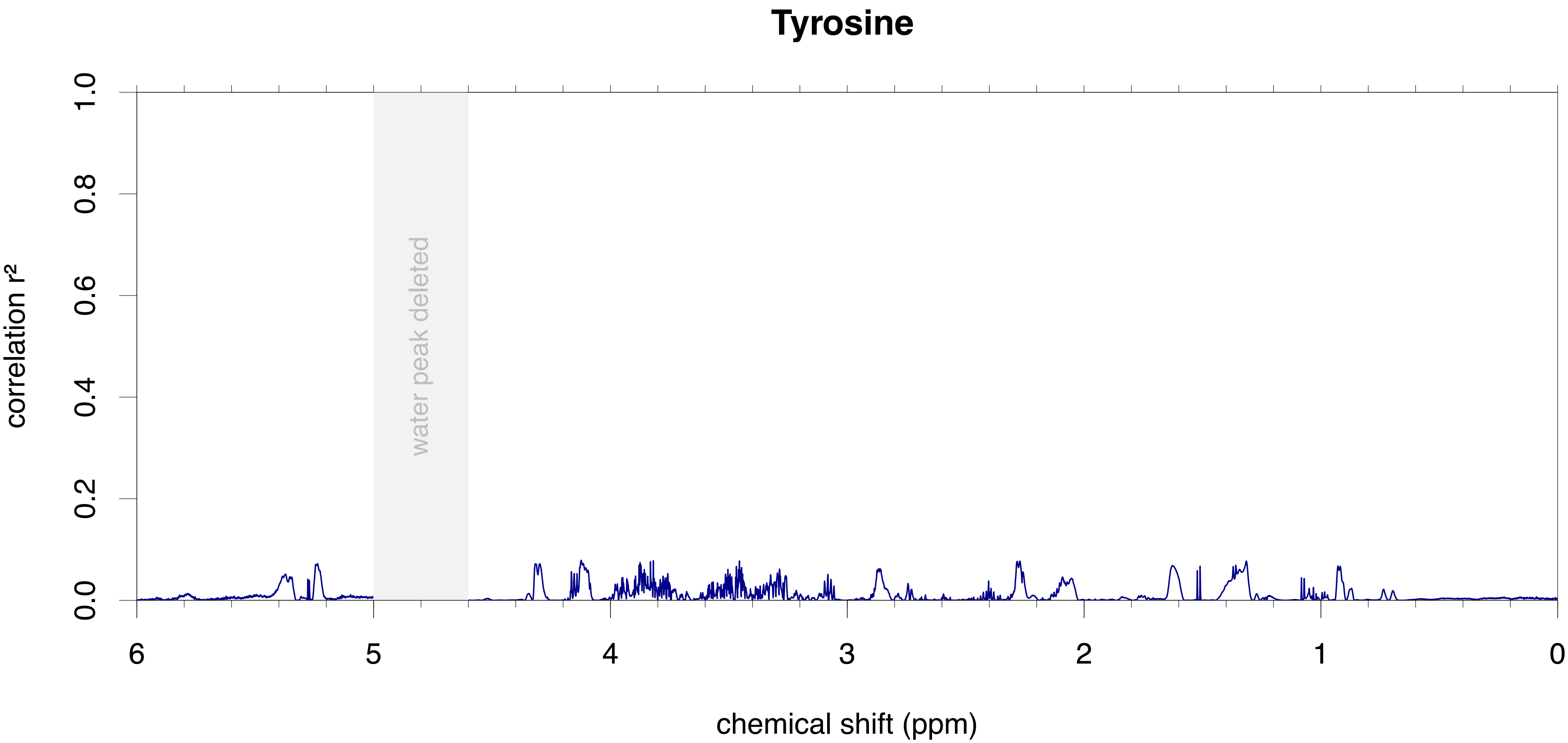

Tyrosine
1.0
0.8
0.6
0.4
0.2
0.0
correlation r²
water peak deleted
6
5
4
3
2
1
0
chemical shift (ppm)

Tyrosine (HMDB)

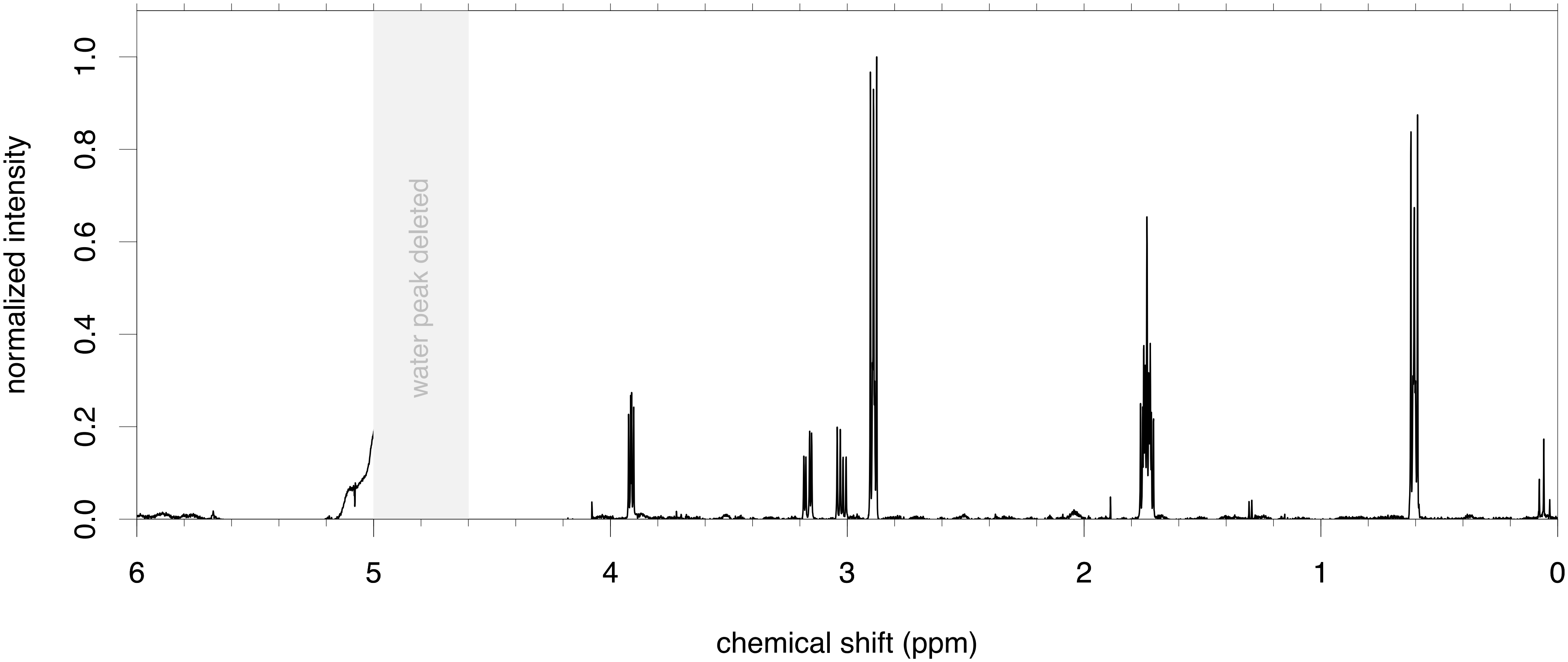

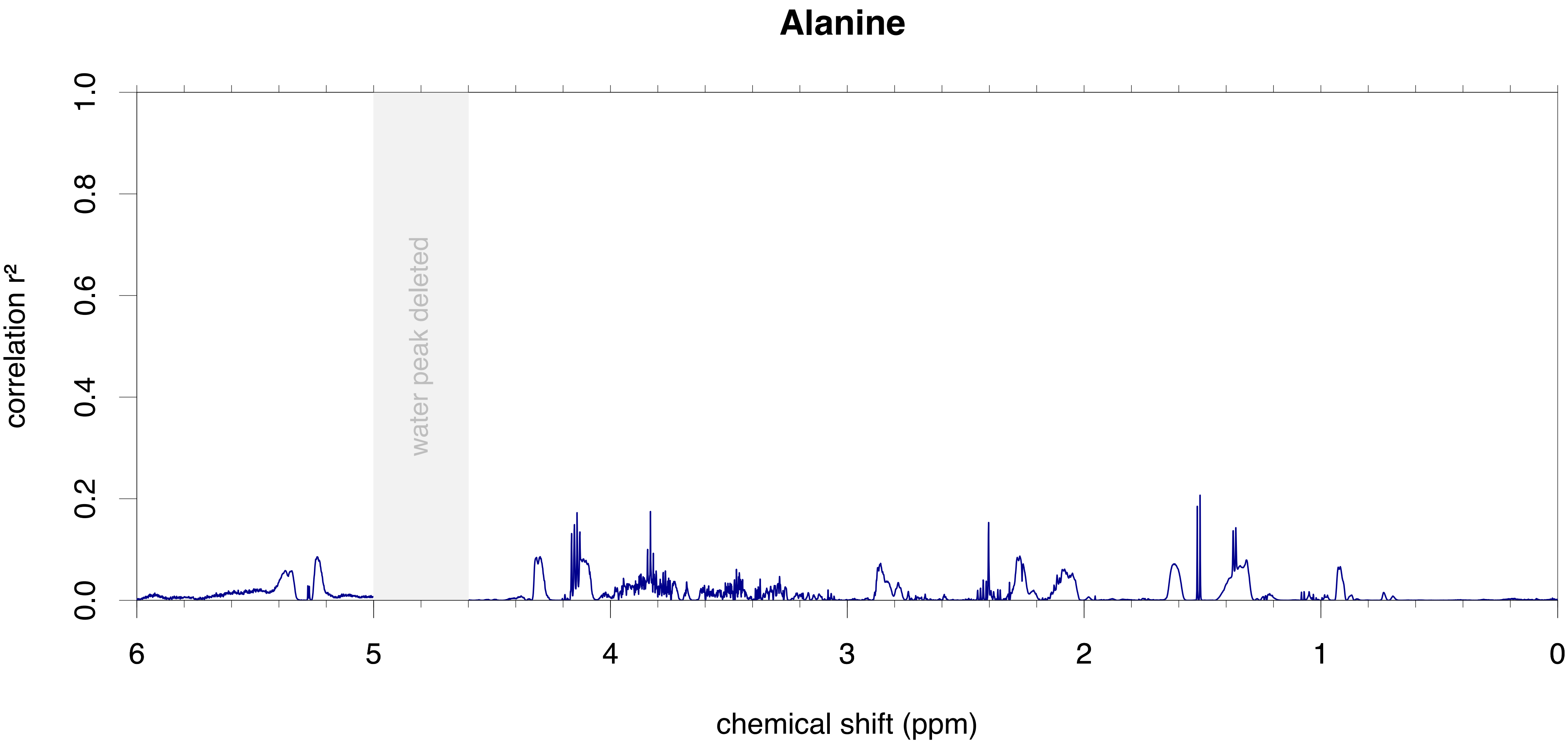

Alanine
water peak deleted
correlation r²
1.0
0.8
0.6
0.4
0.2
0.0
6
5
4
3
2
1
0
chemical shift (ppm)

Alanine (HMDB)

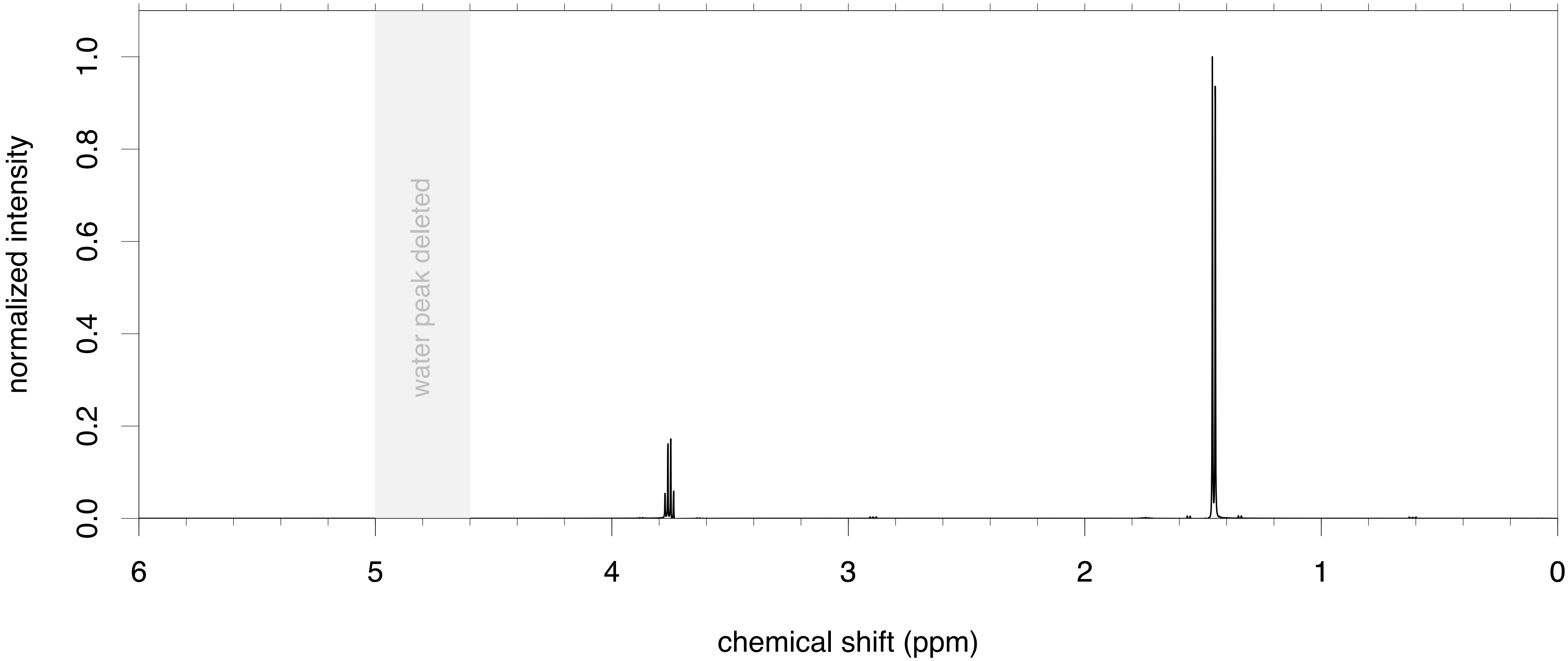

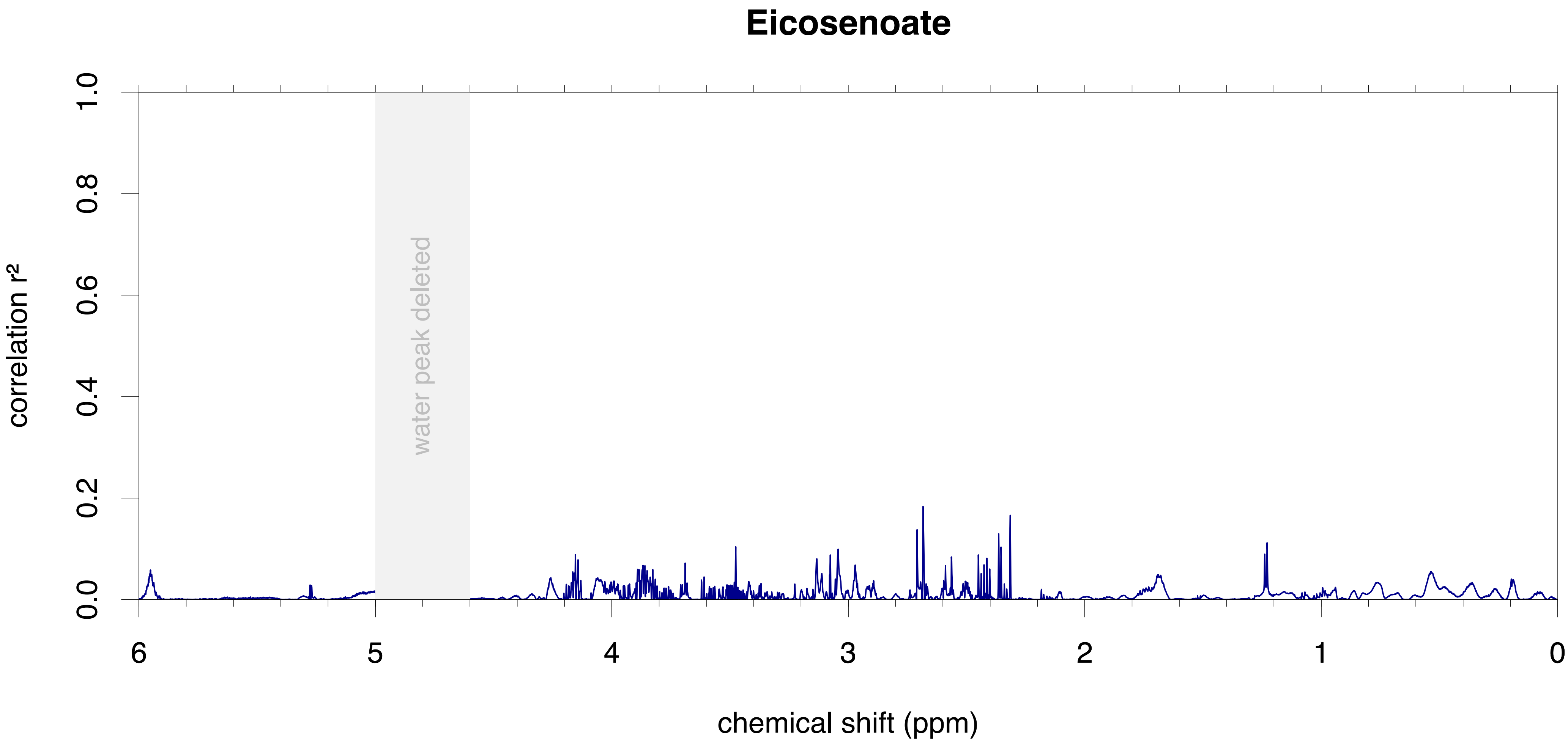

Eicosenoate
correlation r²
1.0
0.8
0.6
0.4
0.2
0.0
water peak deleted
6
5
4
3
2
1
0
chemical shift (ppm)

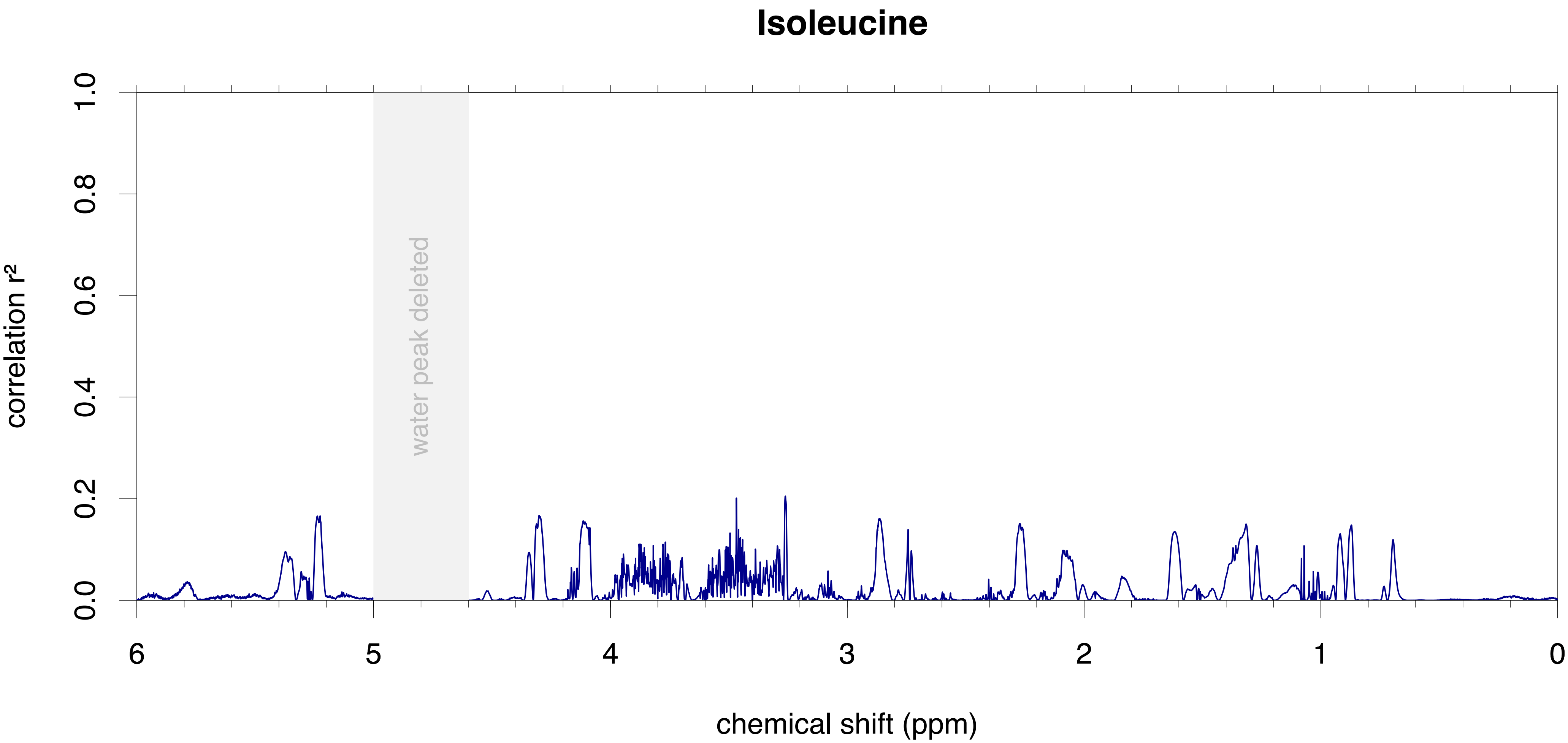

Isoleucine
correlation r²
1.0
0.8
0.6
0.4
0.2
0.0
water peak deleted
6
5
4
3
2
1
0
chemical shift (ppm)

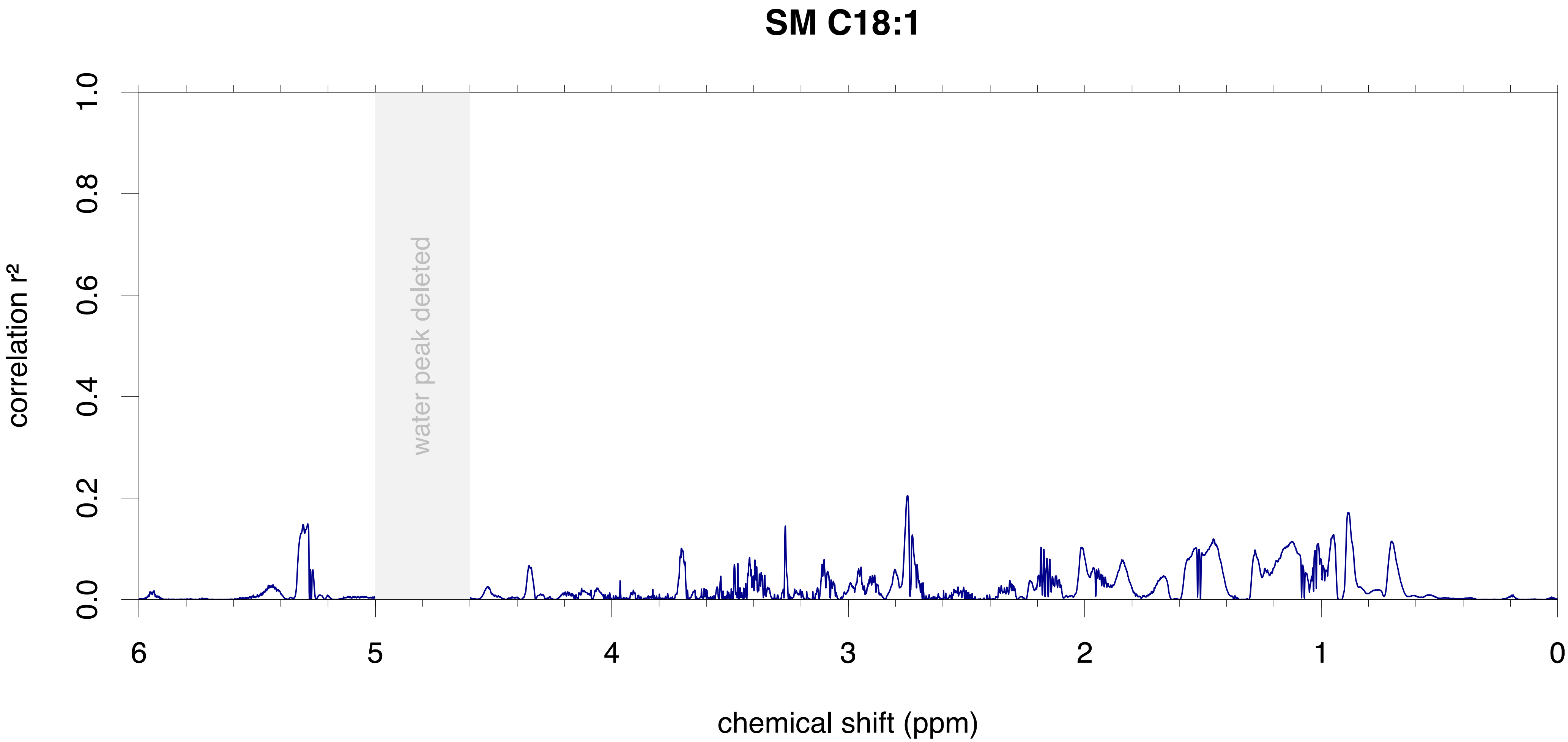

SM C18:1
correlation r²
1.0
0.8
0.6
0.4
0.2
0.0
water peak deleted
6
5
4
3
2
1
0
chemical shift (ppm)

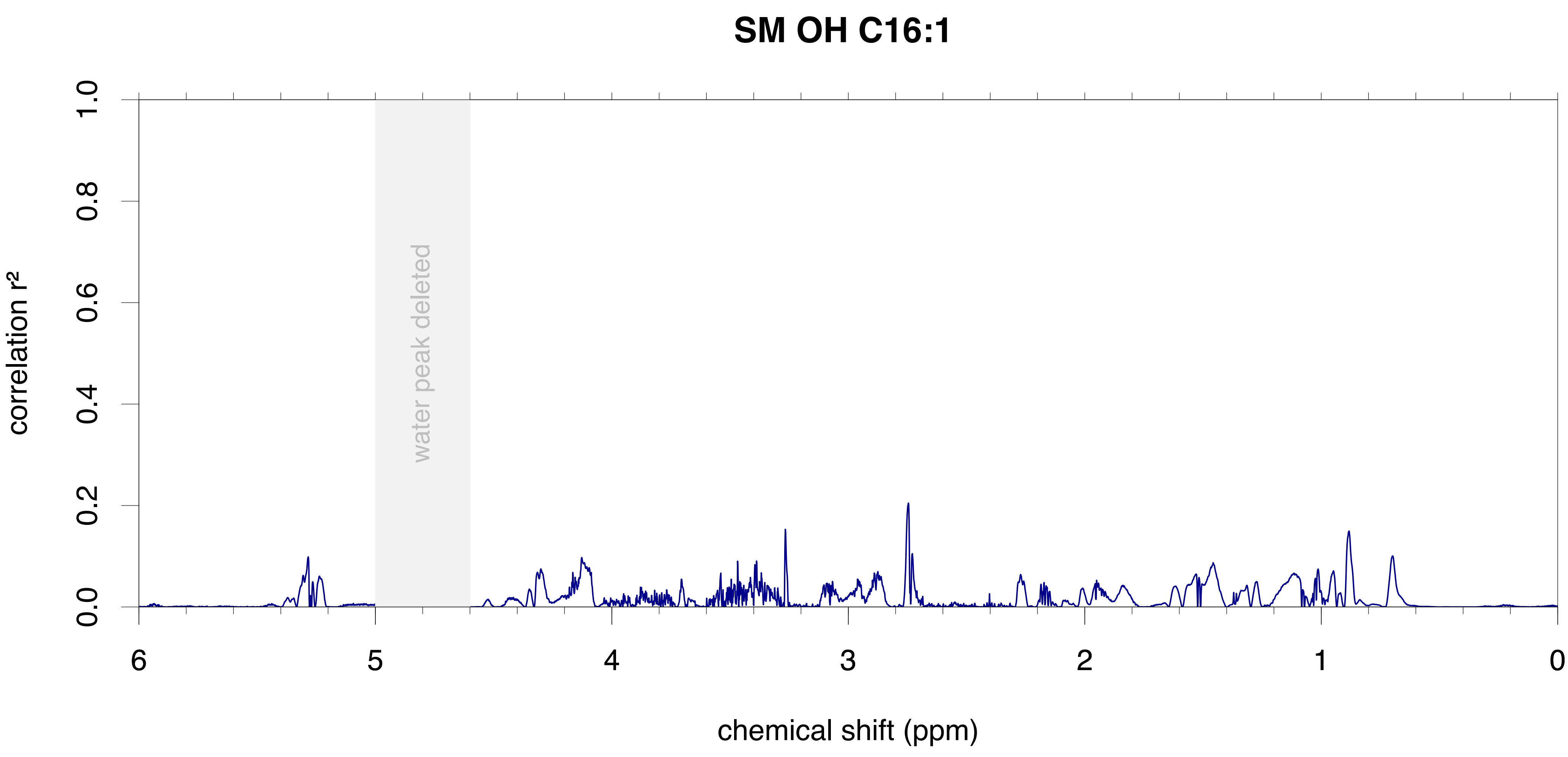

SM OH C16:1
correlation r²
1.0
0.8
0.6
0.4
0.2
0.0
water peak deleted
6
5
4
3
2
1
0
chemical shift (ppm)

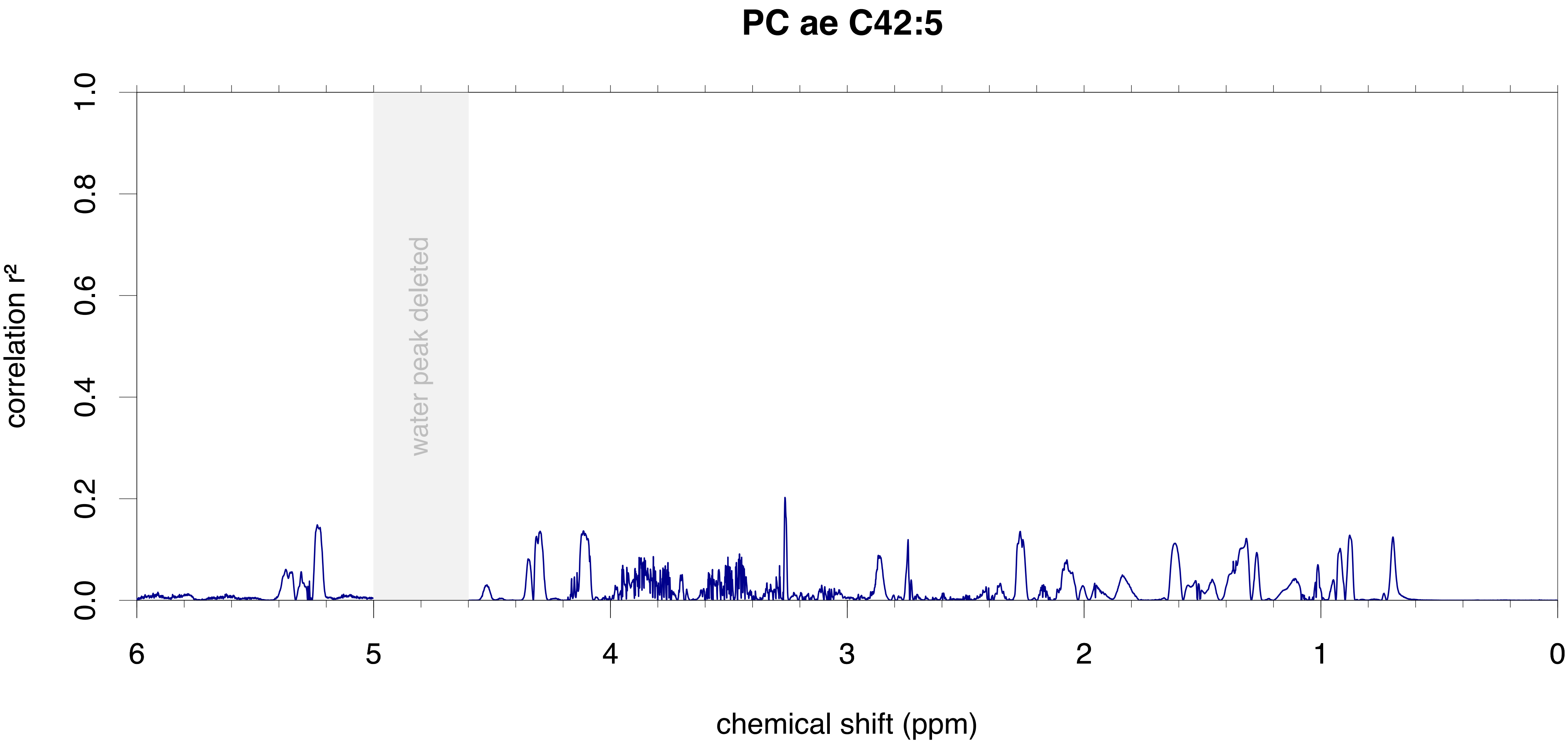
PC ae C42:5
correlation r²
1.0
0.8
0.6
0.4
0.2
0.0
water peak deleted
6
5
4
3
2
1
0
chemical shift (ppm)

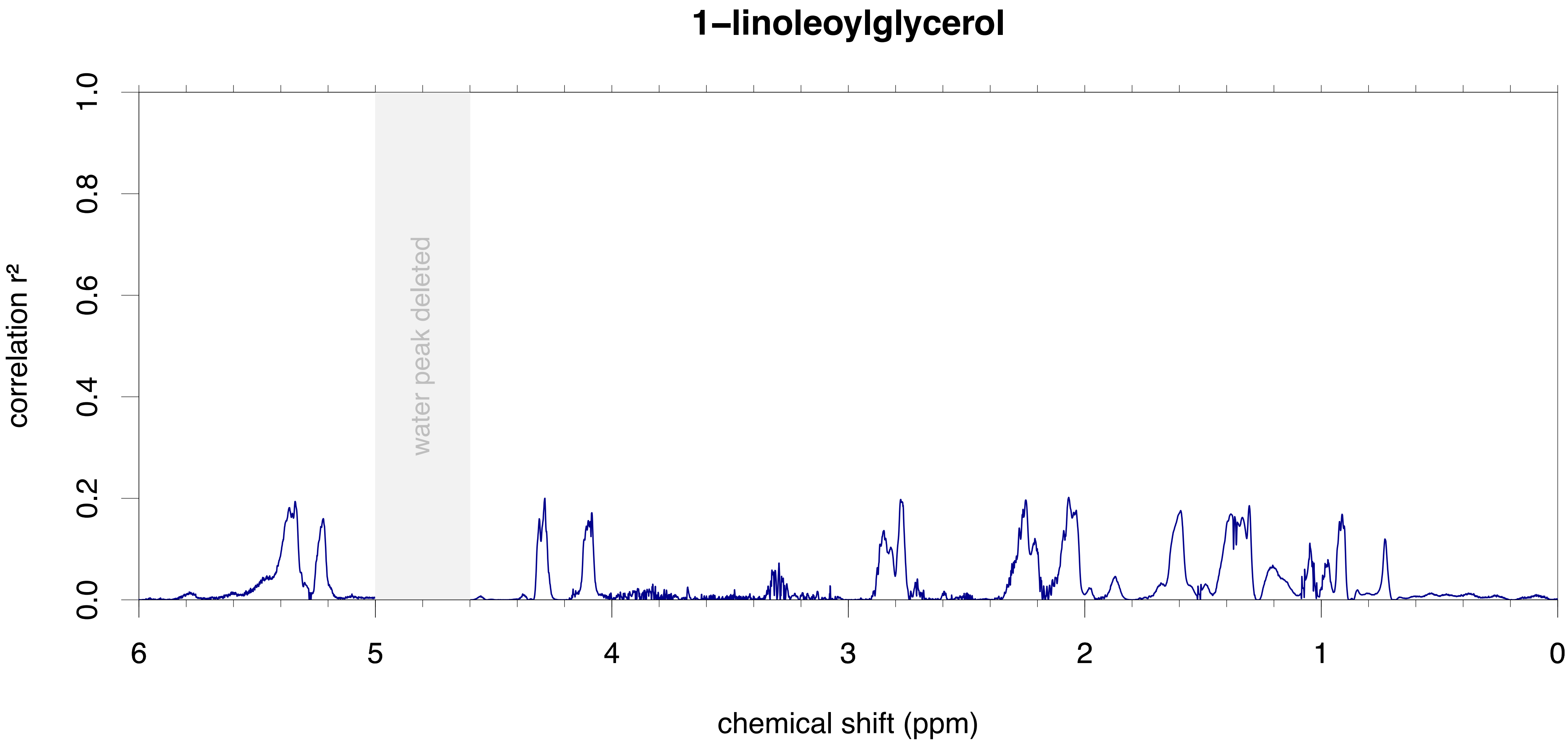

1−linoleoylglycerol
correlation r²
0.0
0.2
0.4
0.6
0.8
1.0
water peak deleted
6
5
4
3
2
1
0
chemical shift (ppm)

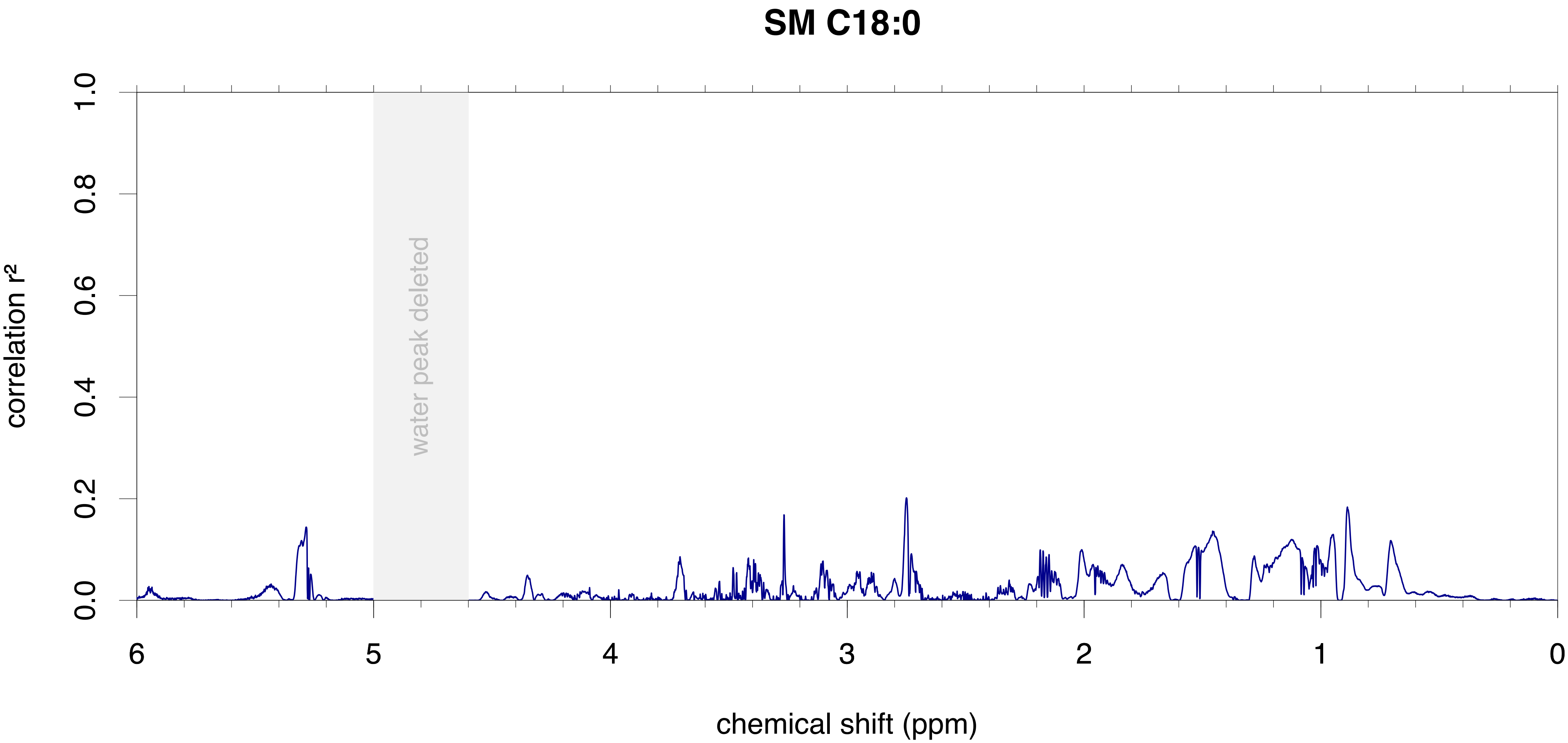
SM C18:0
correlation r²
chemical shift (ppm)
water peak deleted
0.0
0.2
0.4
0.6
0.8
1.0
6
5
4
3
2
1
0

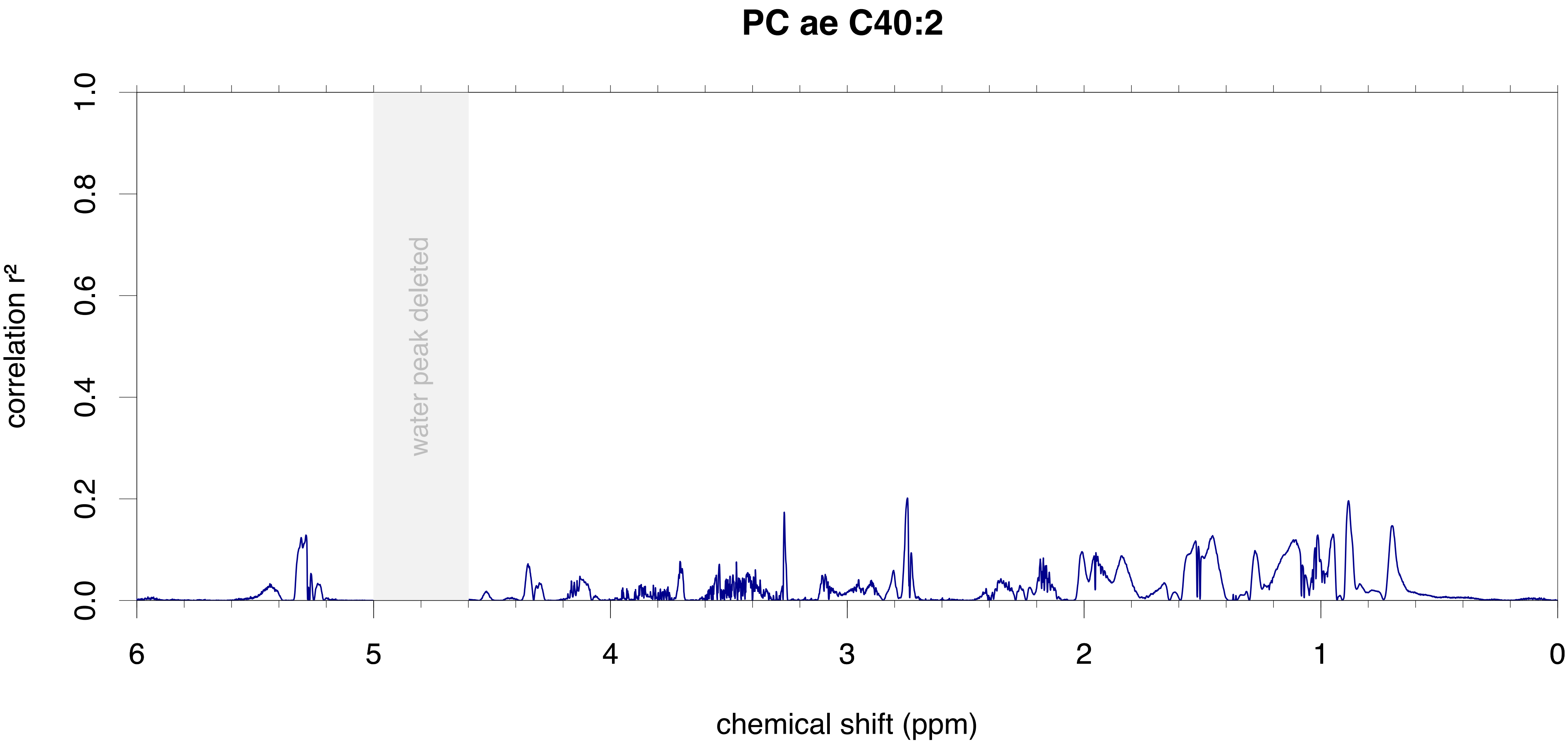
PC ae C40:2
correlation r²
1.0
0.8
0.6
0.4
0.2
0.0
water peak deleted
6
5
4
3
2
1
0
chemical shift (ppm)

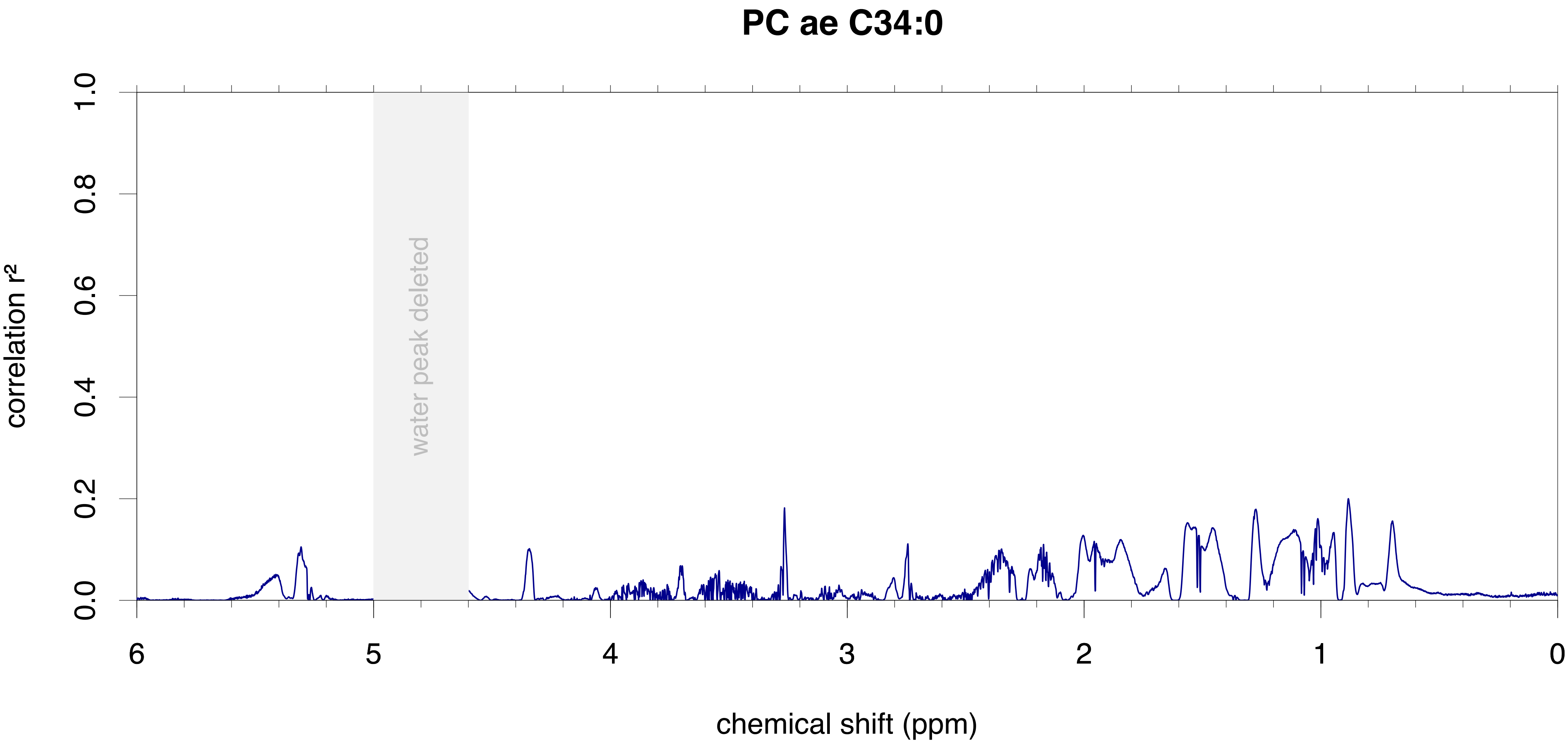
PC ae C34:0
correlation r²
1.0
0.8
0.6
0.4
0.2
0.0
water peak deleted
6
5
4
3
2
1
0
chemical shift (ppm)
