# Supplementary material for: Identification and MS-assisted interpretation of genetically influenced NMR signals in human plasma
Source: Genome Med. 2013 Feb 15;5(2):13. doi: 10.1186/gm417 (PMC3706909; doi:10.1186/gm417)
Supplement: Additional file 3 — Table S1. Spearman correlations between NMR intensities and metabolite concentrations measured on different platforms. The 'Chemical Shift' column lists the position of the signal intensities that show the best correlation (for rs2≥0.20) with the chemical compound noted in the 'Metabolite' column. '▲' 'indicates positive correlation, '▼' anticorrelation. 'N' is the number of samples used for calculating the correlations where valid NMR data points and metabolite concentrations were jointly available.'CV' gives the coefficient of variation in the quality control samples, where available. Chemical shifts are reported in parts per million (ppm).Table S2. Non-parametric tests for genetic associations with NMR bins and NMR ratios. All associations listed in Table 1 were tested separately using a non-parametric test. To test for associations between a SNP and an NMR trait (individual chemical shift or ratio between intensities at two different chemical shifts), Spearman's rho statistic was used; the resulting P values are given as ps. For comparison, the P values of the age- and gender-corrected linear models are given as plm. Chemical shifts are reported in parts per million (ppm). See Table 1 for details about the SNPs and the number of tested traits for each variant. [file gm417-S3.PDF]

Table S1. Spearman correlations between NMR intensities and metabolite concentrations measured on different platforms. The "Chemical Shift" column lists the position of the signal intensities that show the best correlation (for $r_s^2 \geq 0.20$) with the chemical compound noted in the "Metabolite" column. "▲" indicates positive correlation, "▼ " anticorrelation. "N" is the number of samples used for calculating the correlations where valid NMR data points and metabolite concentrations were jointly available. "CV" gives the coefficient of variation in the quality control samples, where available. Chemical shifts are reported in parts per million (ppm).

Chemical shift	r_s^2		Metabolite	Platform	N	CV
2.257	0.94	▲	Triglyceride	Clinical Biochemistry	1,726	
0.892	0.82	▲	Total cholesterol	Clinical Biochemistry	1,742	
0.870	0.69	▲	HDL cholesterol	Clinical Biochemistry	1,743	
3.386	0.61	▲	1,5-anhydroglucitol	Non-targeted, semi-quant. MS	1,749	5.6%
3.276	0.56	▲	LDL Cholesterol	Clinical Biochemistry	1,742	
3.965	0.55	▲	Creatine	Non-targeted, semi-quant. MS	1,740	5.4%
5.783	0.51	▲	Urea	Non-targeted, semi-quant. MS	1,737	10.1%
3.875	0.51	▲	Glucose	Clinical Biochemistry	1,718	
4.141	0.50	▲	Lactate	Non-targeted, semi-quant. MS	1,725	8.6%
2.802	0.48	▲	PC aa C38:5	Targeted, quantitive MS	1,732	5.6%
2.794	0.48	▲	PC aa C38:4	Targeted, quantitive MS	1,728	5.3%
1.285	0.42	▲	PC aa C36:1	Targeted, quantitive MS	1,724	5.2%
0.886	0.39	▲	Phosphate	Non-targeted, semi-quant. MS	1,748	7.8%
2.812	0.39	▲	PC aa C40:6	Targeted, quantitive MS	1,728	5.0%
0.879	0.38	▲	PC ae C32:2	Targeted, quantitive MS	1,733	13.4%
2.796	0.38	▲	PC aa C40:5	Targeted, quantitive MS	1,723	5.2%
2.744	0.38	▲	PC ae C34:3	Targeted, quantitive MS	1,735	5.7%
0.886	0.37	▲	Palmitoyl-sphingomyelin	Non-targeted, semi-quant. MS	1,741	
2.685	0.37	▲	Citrate	Non-targeted, semi-quant. MS	1,737	9.2%
5.307	0.37	▲	PC ae C40:1	Targeted, quantitive MS	1,734	9.3%
2.806	0.36	▲	PC aa C38:6	Targeted, quantitive MS	1,728	5.3%
2.803	0.36	▲	PC aa C36:5	Targeted, quantitive MS	1,715	6.4%
1.419	0.36	▲	PC aa C38:3	Targeted, quantitive MS	1,732	4.8%
2.807	0.35	▲	PC aa C36:6	Targeted, quantitive MS	1,726	10.6%
2.806	0.34	▲	PC ae C38:0	Targeted, quantitive MS	1,728	11.9%
2.018	0.34	▲	PC aa C36:3	Targeted, quantitive MS	1,729	5.5%
1.261	0.33	▲	PC aa C34:3	Targeted, quantitive MS	1,730	6.5%
1.295	0.33	▲	1-palmitoylglycerol	Non-targeted, semi-quant. MS	1,589	19.9%
2.798	0.33	▲	PC aa C36:4	Targeted, quantitive MS	1,727	6.3%
1.251	0.33	▲	PC aa C34:4	Targeted, quantitive MS	1,727	6.1%
3.476	0.32	▲	3-hydroxybutyrate	Non-targeted, semi-quant. MS	1,726	8.0%
3.264	0.32	▲	PC ae C32:1	Targeted, quantitive MS	1,733	7.0%
0.888	0.30	▲	PC aa C28:1	Targeted, quantitive MS	1,732	10.9%
1.560	0.30	▲	PC aa C36:2	Targeted, quantitive MS	1,726	5.7%
2.744	0.30	▲	PC ae C34:2	Targeted, quantitive MS	1,734	5.8%
6.961	0.30	▼	Oleate	Non-targeted, semi-quant. MS	1,750	6.7%

2.805	0.30	▲	PC aa C42:6	Targeted, quantitive MS	1,724	13.3%
5.309	0.30	▲	PC ae C42:1	Targeted, quantitive MS	1,730	16.2%
0.882	0.29	▲	PC aa C32:3	Targeted, quantitive MS	1,733	12.6%
2.749	0.29	▲	SM C16:1	Targeted, quantitive MS	1,732	7.2%
4.323	0.29	▲	PC aa C32:1	Targeted, quantitive MS	1,712	5.8%
2.748	0.29	▲	SM(OH) C22:1	Targeted, quantitive MS	1,737	8.3%
5.324	0.28	▲	Alpha-tocopherol	Non-targeted, semi-quant. MS	1,726	19.3%
3.387	0.28	▲	Proline	Non-targeted, semi-quant. MS	1,736	5.9%
6.961	0.28	▼	10-heptadecenoate	Non-targeted, semi-quant. MS	1,746	10.2%
3.690	0.28	▲	Glycerol	Non-targeted, semi-quant. MS	1,733	8.9%
2.744	0.28	▲	PC ae C36:2	Targeted, quantitive MS	1,735	6.0%
6.961	0.27	▼	Palmitoleate	Non-targeted, semi-quant. MS	1,740	7.5%
2.746	0.27	▲	SM(OH) C22:2	Targeted, quantitive MS	1,736	7.8%
1.248	0.27	▲	PC aa C40:4	Targeted, quantitive MS	1,711	5.1%
3.871	0.27	▲	Mannose	Non-targeted, semi-quant. MS	1,735	17.1%
1.284	0.26	▲	PC aa C32:0	Targeted, quantitive MS	1,729	6.1%
1.992	0.26	▲	PC aa C34:1	Targeted, quantitive MS	1,726	6.7%
2.750	0.26	▲	SM C24:0	Targeted, quantitive MS	1,735	8.4%
2.744	0.26	▲	PC ae C40:3	Targeted, quantitive MS	1,736	15.1%
2.744	0.26	▲	PC ae C38:2	Targeted, quantitive MS	1,730	6.2%
6.960	0.26	▼	Linoleate	Non-targeted, semi-quant. MS	1,745	6.1%
6.961	0.26	▼	Palmitate	Non-targeted, semi-quant. MS	1,751	7.8%
5.308	0.26	▲	PC aa C40:3	Targeted, quantitive MS	1,719	7.3%
0.883	0.25	▲	PC ae C42:2	Targeted, quantitive MS	1,739	8.4%
5.307	0.25	▲	PC ae C36:5	Targeted, quantitive MS	1,733	5.7%
2.804	0.25	▲	PC ae C38:6	Targeted, quantitive MS	1,731	6.4%
1.283	0.25	▲	PC aa C30:0	Targeted, quantitive MS	1,729	6.2%
0.878	0.25	▲	PC ae C42:3	Targeted, quantitive MS	1,733	8.6%
0.884	0.25	▲	PC ae C36:1	Targeted, quantitive MS	1,729	10.1%
2.748	0.25	▲	SM C16:0	Targeted, quantitive MS	1,741	7.2%
2.744	0.25	▲	PC ae C36:3	Targeted, quantitive MS	1,732	5.9%
3.264	0.24	▲	PC ae C34:1	Targeted, quantitive MS	1,733	6.2%
3.965	0.24	▼	Pyroglutamine	Non-targeted, semi-quant. MS	1,730	9.3%
6.960	0.23	▼	Myristoleate	Non-targeted, semi-quant. MS	1,733	7.8%
1.292	0.23	▲	Inositol 1-phosphate	Non-targeted, semi-quant. MS	1,357	
2.261	0.23	▲	1-oleoylglycerol	Non-targeted, semi-quant. MS	1,653	64.5%
3.297	0.23	▲	Betaine	Non-targeted, semi-quant. MS	1,733	9.0%
6.960	0.23	▼	Myristate	Non-targeted, semi-quant. MS	1,745	7.1%
0.880	0.23	▲	PC ae C40:5	Targeted, quantitive MS	1,732	6.0%
2.748	0.23	▲	SM(OH) C24:1	Targeted, quantitive MS	1,730	10.1%
2.808	0.23	▲	PC ae C40:0	Targeted, quantitive MS	1,733	3.3%
0.886	0.22	▲	PC ae C38:3	Targeted, quantitive MS	1,731	8.6%
6.961	0.22	▼	Dihomo-linoleate	Non-targeted, semi-quant. MS	1,731	11.1%
3.599	0.21	▲	Glycine	Targeted, quantitive MS	1,718	6.9%
2.805	0.21	▲	PC aa C42:5	Targeted, quantitive MS	1,718	15.1%
3.263	0.21	▲	PC ae C42:4	Targeted, quantitive MS	1,730	6.6%
3.331	0.21	▲	Stachydrine	Non-targeted, semi-quant. MS	1,725	6.4%
6.925	0.21	▲	Tyrosine	Non-targeted, semi-quant. MS	1,739	6.1%
1.510	0.21	▲	Alanine	Non-targeted, semi-quant. MS	1,745	15.1%
6.961	0.21	▼	Eicosenoate	Non-targeted, semi-quant. MS	1,734	12.9%
3.261	0.21	▼	Isoleucine	Non-targeted, semi-quant. MS	1,737	6.0%

2.749	0.20	▲	SM C18:1	Targeted, quantitive MS	1,736	6.6%
2.746	0.20	▲	SM(OH) C16:1	Targeted, quantitive MS	1,740	7.8%
3.263	0.20	▲	PC ae C42:5	Targeted, quantitive MS	1,728	4.7%
2.068	0.20	▲	1-linoleoylglycerol	Non-targeted, semi-quant. MS	1,721	75.0%
2.750	0.20	▲	SM C18:0	Targeted, quantitive MS	1,736	6.8%
2.746	0.20	▲	PC ae C40:2	Targeted, quantitive MS	1,734	7.0%
0.884	0.20	▲	PC ae C34:0	Targeted, quantitive MS	1,728	7.6%

Table S2. Non-parametric tests for genetic associations with NMR bins and NMR ratios. All associations listed in Table 1 were tested separately using a non-parametric test. To test for associations between a SNP and an NMR trait (individual chemical shift or ratio between intensities at two different chemical shifts), Spearman's rho statistic was used; the resulting p-values are given as p_s. For comparison, the p-values of the age- and gender-corrected linear models are given as p_{lm}. Chemical shifts are reported in parts per million (ppm). See Table 1 for details about the SNPs and the number of tested traits for each variant.

**Locus**	**SNP**	**Chemical shift**	p_s	p_{lm}	**Chemical shifts for ratios**	p_s	p_{lm}
GCKR	rs780094	1.370	$9.31x10^{-10}$	$1.21x10^{-10}$	3.286 / 1.370	$2.35x10^{-15}$	$2.8x10^{-15}$
CPS1	rs2216405	3.599	$5.14x10^{-14}$	$4.46x10^{-14}$	3.599 / 2.475	$1.57x10^{-18}$	$1.8x10^{-19}$
PYROXD2	rs4488133	2.757	$2.87x10^{-11}$	$7.34x10^{-12}$	2.757 / 2.755	$1.13x10^{-109}$	$2.9x10^{-94}$
FADS1	rs174547	2.801	$3.24x10^{-32}$	$3.96x10^{-35}$	2.801 / 2.017	$3.02x10^{-88}$	$1.1x10^{-94}$
APOA1	rs3741298	2.038	$1.61x10^{-10}$	$8.35x10^{-11}$	4.162 / 4.082	$1.21x10^{-13}$	$1.8x10^{-14}$
LIPC	rs4775041	1.283	$2.84x10^{-08}$	$1.44x10^{-10}$	1.068 / 1.029	$2.25x10^{-16}$	$3.6x10^{-21}$
CETP	rs247617	3.259	$1.15x10^{-12}$	$7.61x10^{-15}$	2.211 / 2.011	$2.64x10^{-15}$	$1.1x10^{-18}$
